# Parasitic contamination in vegetables for human consumption: a systematic review and meta-analysis

**DOI:** 10.1590/S1984-29612024040

**Published:** 2024-09-06

**Authors:** Rafael Alves Santomauro, Fernanda Pinto-Ferreira, Nathália Miasato Pimont, Mariana da Silva Marques, Maria Clara Soares Lemos, Winni Alves Ladeia, Letícia Santos Balbino, Italmar Teodorico Navarro

**Affiliations:** 1 Departamento de Medicina Veterinária Preventiva, Universidade Estadual de Londrina – UEL, Londrina, PR, Brasil

**Keywords:** Lettucce, helminths, kitchen gardens, meta-analyses, parasites, protozoa, Alface, helmintos, hortas, meta-análise, parasitos, protozoários

## Abstract

The study conducted a review of the parasitological profile of vegetables from 2001 to 2021, considering the type, consumption, and cultivation, globally. The databases searched included MEDLINE, SciELO, Web of Science, Science Direct, and Scopus using the terms "Detection OR Prevalence OR Incidence OR occurrence OR contamination AND vegetable OR fruit AND Helminth OR egg OR Parasite OR cysts OR protozoa". A total of 16,600 articles were found, 117 of which were reviewed. Of the 391,291 samples, 3.85% (15,095) were contaminated by parasites. Among those positive, 30.10% (4,543/15,095) contained enteroparasites commonly of human origin and 58.78% (8,873/15,095) came from markets. Few articles mentioned the cultivation type, but among those, conventional cultivation showed more contamination (42.34%; 224/529). Herbaceous vegetables were the most contaminated (56.84%; 8,580/15,095. *Ascaris lumbricoides* was found in 10.16% (1,535/15,095) of the samples. Lettuce was the most contaminated (20.43%; 3,084/15,095).

## Resumo

O estudo realizou uma revisão do perfil parasitológico de vegetais, de 2001 a 2021, considerando o tipo, consumo e cultivo, global. Foram pesquisadas as bases MEDLINE, SciELO, Web of Science, Science Direct e Scopus com os termos "*Detection OR Prevalence OR Incidence OR occurrence OR contamination AND vegetable OR fruit AND Helminth OR egg OR Parasite OR cysts OR protozoa*". Foram encontrados 16.600 artigos, 117 dos quais foram revisados. Das 391.291 amostras, 3,85% (15.095) estavam contaminadas por parasitos. Entre as positivas, 30,10% (4.543/15.095) continham enteroparasitos comumente de origem humana e 58,78% (8.873/15.095) eram originadas de mercados. Poucos artigos mencionaram o tipo de cultivo, mas entre esses, o convencional apresentou maior contaminação (42,34%; 224/529). Vegetais herbáceos foram os mais contaminados (56,84%; 8.580/15.095). *Ascaris lumbricoides* foi encontrado em 10,16% (1.535/15.095) das amostras. Alface foi o vegetal mais contaminado (20,43%; 3.084/15.095).

## Introduction

Vegetables are important components of a healthy diet. They have few calories, lipids, or proteins, but are rich in fiber, carbohydrates, minerals, and vitamins, as well as functional compounds such as antioxidants, which prevent the synthesis of inflammatory substances and other components related to the prevention and inhibition of cancers and tumors (Favell, 1998; Carvalho et al., 2006; Wynn et al., 2010).

Gastrointestinal parasites, which are common across the globe, are caused by helminths and protozoa and are endemic in underdeveloped countries, representing an important public health problem that is directly related to poor sanitation and low socioeconomic conditions (Brasil, 2005; Saraiva et al., 2005). These organisms can cause malabsorption of nutrients by the intestines, resulting in diarrhea, malnutrition, and abdominal pain, especially in children (Saraiva et al., 2005).

Vegetables that are consumed raw can provide important parasite transmission routes (Vollkopf et al., 2006; Pinto-Ferreira et al., 2019a), for its cultivation conditions, including the quality of the water destined for irrigation, soil quality, and type of fertilizer used, as well as the harvesting means, transport, and storage, are associated with contamination by different parasitic forms: eggs, larvae, and cysts (Simões et al., 2001; Beuchat, 2002; Ferreira et al., 2018; Pinto-Ferreira et al., 2019b; Al Nahhas & Aboualchamat, 2020).

Thus, the main aim of this study was to identify the parasitological profile of vegetables for consumption, as well as to associate contamination with different types of vegetables, types of cultivation and consumption, countries socioeconomic conditions where the vegetables were originally grown, and their zoonotic potential. Parasites were found through a systematic review covering the years 2001 to 2021.

## Material and Methods

The review process followed the protocol suggested by the Preferred Reporting Items for Systematic Reviews and Meta-Analyses (PRISMA) (Page et al., 2021). During March 2021, studies were consulted and selected from the MEDLINE (via PubMed), SciELO, Web of Science, Science Direct, and Scopus databases. The search terms were “(((Detection OR Prevalence OR Incidence OR occurrence OR contamination) AND vegetable OR fruit) AND Helminth OR egg OR Parasite OR cysts OR protozoa)”.

The initial selection by titles and abstracts was performed independently by three researchers, who also evaluated the full texts of all potentially relevant studies. Selected articles were manually searched for possible eligible literature. In the case of articles that were difficult to access, the authors were contacted to request the studies.

The review examined studies that provided data on vegetable contamination by parasites over the last twenty years (2001-2021), which included information on the prevalence of contamination and plant type, and were written in Portuguese, English, or Spanish. However, studies that were published more than 20 years ago, and those that did not have complete results, reported contamination from other foods, were presented at conferences without full text or were duplicated, were excluded.

After a careful evaluation of the articles selected, those were organized in Mendeley software (Mendeley, London, UK) and the following data were extracted: first author surname, year of publication and study conclusion, country, total number and contaminated samples, parasites found and their respective biological forms, vegetables evaluated in the study, cultivation type, and vegetable origin. Data were obtained separately and entered an Excel spreadsheet (Microsoft, Redmond, Washington, USA) in which they were organized and categorized. The taxonomic class of organisms and their zoonotic potential were included, as well as their ability to parasitize humans, animals, or both, the vegetable type (herbaceous, fruit, or tuberous), its consumption type (raw, cooked, or both), and the Human Development Index (HDI) of the related country (UNDP, 2020). Studies in which there were no total number of samples, or the total of positive samples were not considered or where their deduction was not possible, were excluded.

R software (R Core Team, 2022) was used for both descriptive statistics and meta-analysis. A confidence interval of 0.95 was considered for meta-analysis interpretation. Pooled parasite frequency in vegetables and heterogeneity were verified by Cochran's Q test and measured according to inconsistency (I^2^) (meta and metafor packages (Viechtbauer, 2010); metaprop function, maximum likelihood, and inverse-variance weight model). The fixed effects model was primarily used to quantify heterogeneity; in the case of a high I^2^ (> 50%), the random effects model was applied using the variables that presented the greatest variability in descriptive analysis for grouping (Higgins & Thompson, 2002; Higgins et al., 2009). To identify the contribution of the study to heterogeneity, Baujat statistics were used (Baujat et al., 2002). To evaluate bias, a funnel plot was viewed to verify error tendencies, and Egger’s test was used to verify the significance of bias (Sterne & Egger, 2001).

Vegetable parasites, such as mites and aphids, and their respective biological forms, as well as free-living organisms and bacteria, were disregarded from these analyses.

## Results

**Figure 1:**
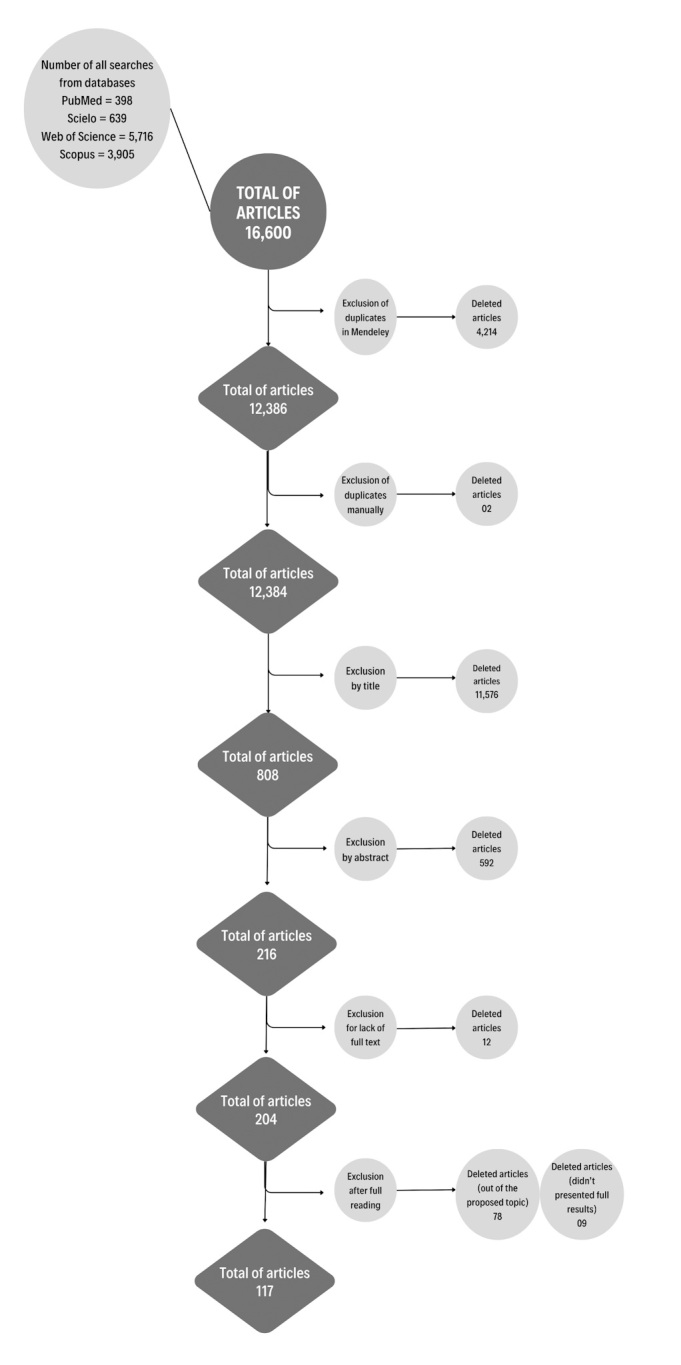
Flowchart of articles included in this systematic review, of years 2001 to 2021.

Between 2001 and 2021, 16,600 articles were found in the databases, and after careful screening of these studies, 117 were selected for statistical analysis (Figure 1). The studies included in this review can be consulted on spreadsheet S1 and on References S1, in the Supplementary Material.

Overall, 391,291 foods were analyzed, with 3.85% (15,095) of the samples contaminated by at least one parasitic species. Among the positive samples, parasites originated from humans represented 30.10% (4,543/15,095), zoonotics 28.59% (4,316/15,095), and animals 3.06% (463/15,095), while in 38.24% (5,773/15,095), the authors did not report the organism species, only the genus, family, or class; therefore, these could not be included in any category of origin and their zoonotic character could not be assessed.

As for the contaminated food origin, 58.78% (8,873/15,095) were from markets, 21.08% (3,182/15,095), vegetable gardens, 2.19% (330/15,095) fairs, and 17.83% (2,692 /15,095) originated from other places or were not indicated by the authors; vegetables from restaurants represented 0.12% (18/15,095).

Only 3.50% (529/15,095) of the contaminated foods evaluated contained information on the cultivation type, in which 21.17% (112/529) hydroponic, 36.48% (193/529) organic, and 42.34% (224/529) conventional.

Vegetables preferably consumed raw or cooked (mixed consumption) represented 70.33% (10,617/15,095) of the analyses performed for positive samples, whereas raw constituted 24.94% (3,766/15,095) and cooked comprised 4.71% (712/15,095).

Considering the different vegetable types, 56.84% (8,580/15,095) of herbs, 16.73% (2,526/15,095) of tuberous plants and 10.33% (1,560/15,095) of fruits showed contamination, while 16.09% (2,429/15,095) were considered “inconclusive”, as it were ready-to-eat foods, commonly sold as “salads”.

Of all positive samples, *Ascaris lumbricoides* represented 10.16% (1,535/15,095), *Giardia lamblia* 6.77% (1,022/15,095), hookworms 5.52% (833/15,095), *Entamoeba coli* 4.62% (697/15,095), and *Strongyloides stercoralis* 4.60% (695/15,095). A list of other parasites detected is shown in Table 1.

**Table 1:** List of parasites found in vegetable samples between the years 2001 to 2021, globally.

Parasite	Positive	Total of analysis	Positives/analyses	Positives/total of positives
*Ascaris lumbricoides*	1535	26186	5.86%	10.17%
*Giardia lamblia*	1022	23069	4.43%	6.77%
Hookworms	833	11452	7.27%	5.52%
*Entamoeba* spp.	746	3323	22.45%	4.94%
*Ascaris* spp.	697	7705	9.05%	4.62%
*Entamoeba coli*	697	19849	3.51%	4.62%
*Strongyloides stercoralis*	695	20744	3.35%	4.60%
*Cryptosporidium* spp.	658	8263	7.96%	4.36%
*Entamoeba histolytica*	657	16517	3.98%	4.35%
*Fasciola hepatica*	568	16674	3.41%	3.76%
*Taenia* spp.	533	20700	2.57%	3.53%
*Toxocara* spp.	483	17406	2.77%	3.20%
*Hymenolepis nana*	420	23025	1.82%	2.78%
*Giardia* spp.	380	5822	6.53%	2.52%
*Toxocara cati*	355	3111	11.41%	2.35%
*Trichuris trichiura*	348	19611	1.77%	2.31%
*Toxocara canis*	316	4151	7.61%	2.09%
*Blastocystis hominis*	281	14662	1.92%	1.86%
*Cryptosporidium parvum*	279	17227	1.62%	1.85%
*Dicrocoelium dendriticum*	272	2873	9.47%	1.80%
*Enterobius vermicularis*	266	3556	7.48%	1.76%
*Balantidium coli*	261	13284	1.96%	1.73%
*Endolimax nana*	214	1851	11.56%	1.42%
*Cyclospora cayetanensis*	197	15556	1.27%	1.31%
Nematodes	180	996	18.07%	1.19%
*Trichomonas hominis*	162	578	28.03%	1.07%
*Ancylostoma duodenale*	150	13753	1.09%	0.99%
*Toxoplasma gondii*	146	5147	2.84%	0.97%
*Entamoeba histolytica/dispar*	141	1816	7.76%	0.93%
*Trichostrongylus* spp.	120	3404	3.53%	0.79%
*Strongyloides* spp.	119	2190	5.43%	0.79%
*Microsporidia* spp.	116	519	22.35%	0.77%
*Trichuris* spp.	105	1942	5.41%	0.70%
*Cystoisospora* spp.	100	12969	0.77%	0.66%
*Fasciola* spp.	94	1815	5.18%	0.62%
*Ancylostoma* spp.	94	3364	2.79%	0.62%
*Taenia/Echinococcus*	85	704	12.07%	0.56%
*Hymenolepis diminuta*	78	900	8.67%	0.52%
Others^*^	692	24577	2.82%	4.58%

* *Toxascaris leonina, Dicrocoelium spp.,* nematode larvae, *Cyclospora* spp., ascarids, unsporulated oocyst, *Enterocytozoon bieneusi, Blastocystis* spp., *Hymenolepis* spp*., Ascaridia gali, Cystoisospora belli*, helminths*, Iodamoeba butschlii, Taenia saginata,* Trichostrongylidae, *Echinococcus multilocularis, Echinococcus* spp., *Entamoeba complex, Hydatigera taeniformis, Paramphistomum* spp., *Eimeria* spp., *Enterocytozoom* spp., *Enterobius* spp., *Haemonchus contortus, Taenia hydatigena, cestodes*, coccidia, *Cryptosporidium andersoni, Iodamoeba* spp., Oxyuridae, *Iodamoeba* spp., Amoeba, *Dipylidium caninum*, *Echinococcus granulosus, Trichuris ovis, Ascaris suum, Diphyllobothrium latum, Physaloptera* spp. *Schistosoma haematobium, Schistosoma mansoni, Strongylida, Taenia polyacantha, Trichostrongylus colubriformes, Acanthamoeba* spp*., Dipylidium* spp., *Heterophyes heterophyes, Schistosoma japonicum, Taenia crassiceps, Toxocara vitulorum, Necator americanus.*

The most frequently contaminated vegetables were lettuce varieties, with 20.43% (3,084/15,095), followed by watercress (5.72%; 864/15,095), leeks (5.68%; 858/15,095), parsley (3.99%; 603/15,095), and cabbage (3.94%; 595/15,095). The complete list of the analyzed foods is shown in Table 2.

**Table 2:** List of vegetables, in their popular nomenclature, analyzed for parasitic contamination in the period from 2001 to 2021, globally.

Food	Positives	Total of analyzes	(Positives/analyzes)	Positivos/ total of Positives
Lettuce	3084	45135	6.83%	20.43%
Cress	864	24583	3.51%	5.72%
Leek	858	24027	3.57%	5.68%
Parsley	603	23782	2.54%	3.99%
Cabbage	595	7415	8.02%	3.94%
Arugula	495	4498	11.00%	3.28%
Carrot	432	7965	5.42%	2.86%
Tomato	429	22852	1.88%	2.84%
Radish	382	25917	1.47%	2.53%
Scallion	348	7561	4.60%	2.31%
Mint	331	9029	3.67%	2.19%
Cucumber	329	4931	6.67%	2.18%
Celery	297	17950	1.65%	1.97%
Cilantro	293	5875	4.99%	1.94%
Basil	236	6332	3.73%	1.56%
Spinach	229	5570	4.11%	1.52%
Roots	225	1749	12.86%	1.49%
Fennel	215	17100	1.26%	1.42%
Strawberry	203	804	25.25%	1.34%
Turnip	203	16545	1.23%	1.34%
Beet	184	17389	1.06%	1.22%
Potato	182	17134	1.06%	1.21%
Green Cabbage	180	16768	1.07%	1.19%
Pumpkin	119	1400	8.50%	0.79%
Dill	92	2756	3.34%	0.61%
Multiple vegetables^*^	3074	39622	7.76%	20.36%
Others^**^	613	16602	3.69%	4.06%

* Multiples vegetables are foods that could not be classified separately for parasitic analyses, as wel as “pumpkin leaves”, “asparagus leaves”, “beet leaves”, “green leaves”, ready-to-eat foods and “corn husks”;

** Others includes the following vegetables: bell pepper, chicory, onion, purslane, parsley, savory, spark, white jute, avocado, pepper, sprouts, pea, Orange, tarragon, vietnamese balm, eggplant, mango, shallot, gongroneme, jute mallow, rhubarb, basil, grape, yam, thyme, garlic, blackberry, cashew, apple, pear, cherry tomato, mustard, banana, bluberry, perilla, milkweed, amaranth, plumosa crest, beans, raspberry, okra, broccoli, chinese yoghurt, marjoram, swiss chard, chrysanthemum, dandelion, endive, ginger, watermelon, plum, blackberry, boldo, canton, cauliflower, turmeric, guava, melon, nectarine, oregano, peach, schizonepeta, green beans and vinegar.

Both the fixed effects and random effects models showed high heterogeneity (I^2^ > 99%), as shown in the forest plot, Figure S1, in the Supplementary Material for comprehensive image analysis. The variables country, HDI, and sample origins showed high variability in the descriptive analysis; therefore, a random effects model was applied subgrouping these variables. The frequency of parasites in vegetables in the random effects model relative to all studies and subgroups is shown in Table 3. Regarding subgroups, the statistical significance was observed among Country and the Sample origin subgroups.

**Table 3:** Pooled frequency of parasites in vegetables according to Random Effects Model of meta-analysis of 117 studies from 2001 to 2021.

Parameter	n	Pooled frequency	CI (0.95)		Heterogeneity (I^2^)	p-value
POOLED FREQUENCY	391291	0.08	0.06	0.09	99%	<0.01
COUNTRY						
Iran	76419	0.05	0.03	0.09	99%	<0.01
Brazil	19158	0.11	0.08	0.14	95%	<0.01
Iraq	205115	0.06	0.02	0.19	100%	<0.01
Saudi Arabia	6989	0.07	0.03	0.16	99%	<0.01
Nigeria	16432	0.07	0.03	0.16	99%	<0.01
Libya	630	0.29	0.26	0.33	not applied	not calculated
Turkey	1550	0.13	0.03	0.52	98%	<0.01
Syria	1444	0.06	0.04	0.10	80%	0.02
Ethiopia	9796	0.08	0.05	0.13	97%	<0.01
United Arab Emirates	574	0.06	0.04	0.08	not applied	not calculated
Spain	76	0.58	0.48	0.70	not applied	not calculated
Cambodia	144	0.23	0.17	0.31	not applied	not calculated
Nepal	1013	0.01	0.01	0.02	not applied	not calculated
India	1312	0.11	0.03	0.43	99%	<0.01
Morocco	304	0.11	0.08	0.15	not applied	not calculated
Portugal and Spain	700	0	0	0.01	not applied	not calculated
Thailand	2340	0.18	0.05	0.64	100%	<0.01
Venezuela	1062	0.04	0.03	0.05	not applied	not calculated
Egypt	4791	0.11	0.06	0.20	98%	<0.01
Palestine	600	0.09	0.07	0.12	not applied	not calculated
Canada	5145	0.01	0	0.10	98%	<0.01
Philippines	1274	0.22	0.20	0.24	0%	0.52
Greece	144	0.01	0	0.06	not applied	not calculated
Poland	1102	0.09	0.05	0.16	83%	<0.01
European countries	1692	0.02	0.01	0.03	not applied	not calculated
Pakistan	13906	0.04	0.02	0.10	99%	<0.01
Korea	832	0.07	0.02	0.17	70%	0.07
Ghana	1440	0.17	0.15	0.19	not applied	not calculated
China	4297	0.03	0	0.22	99%	<0.01
Costa Rica	500	0.06	0.04	0.08	not applied	not calculated
Sudan	1554	0.02	0.01	0.03	not applied	not calculated
Cuba	200	0.02	0.01	0.05	not applied	not calculated
Norway	1188	0.02	0.02	0.03	not applied	not calculated
Vietnam	1963	0.12	0.09	0.17	64%	0.06
Eritrean	56	0.27	0.17	0.41	not applied	not calculated
Italy	3888	0.01	0	0.01	not applied	not calculated
Czech Republic	1246	0.02	0.02	0.03	not applied	not calculated
Jordan	415	0.39	0.34	0.44		
*among groups*						<0.01
HDI						
Low: 1	27838	0.07	0.04	0.13	98%	<0.01
Medium: 2	224678	0.07	0.04	0.13	100%	<0.01
High: 3	112929	0.09	0.07	0.12	99%	<0.01
Very high: 4	25846	0.06	0.03	0.10	98%	<0.01
*among groups*						0.45
SAMPLE ORIGIN						
Vegetable garden	10318	0.14	0.09	0.21	98%	<0.01
Market and garden	41134	0.07	0.04	0.11	97%	<0.01
Market	284640	0.07	0.05	0.10	100%	<0.01
Street fair	2450	0.03	0.02	0.06	94%	<0.01
Multiple locals	31930	0.05	0.02	0.10	99%	<0.01
Not informed	144	0.01	0	0.05	not applied	not calculated
Forest and garden	78	0.19	0.12	0.29	not applied	not calculated
Market and street fair	19802	0.07	0.01	0.28	100%	<0.01
Street fair and garden	480	0.06	0.04	0.09	54%	0.14
Restaurants	315	0.06	0.04	0.09	0%	0.72
*among groups*						<0.01

"n": Number of events, "CI" Confidence interval, "HDI": Human Development Index.

**Figure 2:**
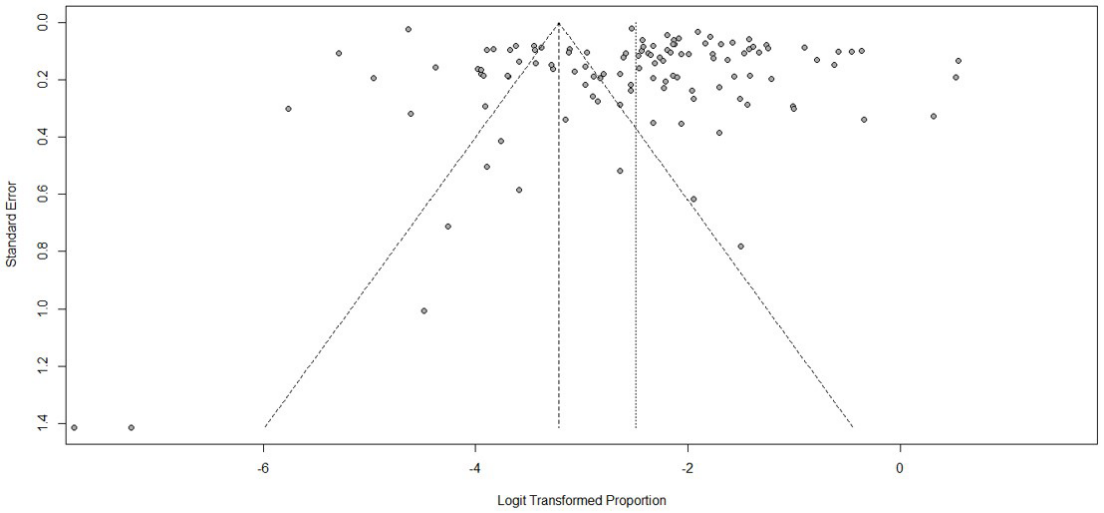
Funnel plot for viewing possible bias among 117 studies regarding parasite frequency in vegetables published from 2001 to 2021.

All models tested showed high I^2^ (> 95%) values, and two studies (Mohameed et al., 2021 and Isazadeh et al., 2020) showed atypical behavior regarding the contribution to bias. The presence of bias among studies was statistically significant in Egger's test (p-value: 0.04). The bias tendency of those studies can be observed in the funnel plot (Figure 2).

The pooled frequency of parasites in vegetables in Random Effects Model regarding all studies and subgroups are visualized in Table 3.

## Discussion

The results of this study showed that for every 100 foods evaluated, four were contaminated by at least one parasite species and most of these had the ability to infect humans, either as their natural host (30.10%) or due to the zoonotic character (28.59%). This is a highly relevant piece of data, as it indicates that at some stage of the production processes of these foods, they were contaminated by human and/or animal feces (Santana et al., 2006), posing a risk to consumer health. On the other hand, in 38.24% (5,773/15,095) of the analyses, the authors of the original study did not report the species of the organism, only the genus, family, or class. This lack of information limited the present study, as some genera, families, or classes may contain different species with zoonotic potential, which are of public health interest.

Regarding the contaminated vegetables origin, 58.78% (8,873/15,095) came from markets. Food contamination in these establishments can occur due to hygienic failures of sellers and customers who handle these vegetables, or during transport and storage to the sales points (Gregório et al., 2012). The vegetable gardens represented 21.08% (3,182/15,095) of the positive samples, and the probable contamination causes of these vegetables are through crop irrigation, as water contaminated by fecal waste is often used, as well as soils containing organic origin fertilizers and animal manure without adequate composting (Gregório et al., 2012). The fairs showed a positivity of only 2.19% (330/15,095), and this result is possibly a reflection of the reduced sample numbers analyzed by the authors. In contrast, food from restaurants revealed 0.12% (18/15,095) positivity, and although this appears low, it demonstrates a high infection risk for consumers, since these foods are ready for consumption.

Vegetables grown in a conventional system had the highest parasitic contamination (42.34%; 224/529), followed by organic (36.48%; 193/529), and hydroponic (21.17%; 112/529). In conventional cultivation, phytosanitary control is conducted through synthetic substances, commonly known as "agricultural pesticides." Although these products are used, vegetable contamination by parasites is not controlled. Similar to organic farming, conventional farming often uses animal manure, which, if not composted for at least 60 days, is considered an important contamination route (Abreu et al., 2016). Hydroponic cultivation is less prone to human and animal contamination due to its management form; however, with unsatisfactory hygienic-sanitary conditions of the water used for vegetable irrigation and/or rinsing, parasitic contamination may occur (Santana et al., 2006; Neres et al., 2011; Pinto-Ferreira et al., 2020; Pacifico et al., 2013).

Among the analyses, 95.28% (14,383/15,095) of the contaminated vegetables were usually consumed raw or raw and cooked, which is relevant in terms of public health, since cooking is one of the main methods to inactivate parasite forms. These foods, when ingested “in natura,” are important transmission routes of pathogenic enteroparasites (Vollkopf et al., 2006; Esteves & Figueirôa 2009; Abreu et al., 2016); therefore, hygiene is extremely important for parasite reduction in these cases.

Herbaceous plants are vegetables whose edible portions develop above the ground, which include lettuce and cabbage (leaves), asparagus and celery (stalks), and broccoli and cauliflower (flowers and inflorescences). In the present review, 56.84% (8,580/15,095) of the vegetables included in this category showed parasitic contamination. The large number of contaminated samples is possibly enhanced by these plants' conformation, which are capable of harboring different parasitic forms from contaminated irrigation water and greater contact with polluted soil (Santarém et al., 2012; Silva et al., 2019; Sá et al., 2020). Vegetables such as lettuce, watercress, parsley, chives, and arugula were the most contaminated, which is an important result as these are frequently consumed foods.

Tuberous vegetables, whose parts used for consumption develop in the soil, such as garlic (bulbs), carrots, and yams (roots), presented contamination by parasites in 16.73% (2,526/15,095) of the samples, which may be indicative of direct and consistent contact with contaminated soil (Vollkopf et al., 2006). Fruits such as watermelon, pea, pumpkin, and cucumber were positive in 10.33% (1,560/15,095) of the analyses. This type of plant presents great diversity in growth terms, which can be creeping, growing close to the ground, or climbing and developing into trees, and its contamination is directly linked to management practices, especially the use of low-quality water. The most critical were ready-to-eat foods, in which 16.09% (2,429/15,095) contained parasites, indicating insufficient hygiene during handling, processing, and storage of these vegetables (Silva et al., 2019).

Among the parasites identified in the analyses, those causing gastroenteric disorders predominated. *Ascaris lumbricoides* affects humans through the ingestion of eggs containing the infective L3 larvae in water and food, which can lead to abdominal discomfort, cramps, and weight loss, among other symptoms (Moura et al., 2015). *Giardia lamblia* is also pathogenic and causes chronic and acute diarrhea, nausea, and colic (Sá et al. 2020). *Entamoeba histolytica* is a protozoan acquired from the ingestion of food containing contaminating cysts, which causes inflammation of the intestinal mucosa, diarrhea, and abdominal pain (Jung et al., 2017). *Strongyloides stercoralis* affects the host intestine, with perforations reported, and causes hypochromic anemia, especially in immunocompromised patients (Maia et al., 2006). *Fasciola hepatica* is a zoonotic parasite that affects humans and herbivorous animals’ livers through the ingestion of plants containing metacercariae. Clinical signs of infection include jaundice, general malaise, fever, and right upper quadrant pain (Fairweather, 2005; Gil et al., 2014). *Toxocara canis* and *T. cati* are parasites whose hosts are canids and felids; however, it can cause toxocariasis in humans from egg ingestion, leading to visceral and ocular problems by *larva migrans* (Gawor et al., 2008; Mattia et al., 2012). *Entamoeba coli* is considered a pathogenic organism in humans as it inhabits the human intestinal microbiota. However, its identification in plant samples indicates food exposure to fecal contamination (Moura et al., 2015). The occurrence of these parasites suggests insufficient sanitary conditions in the different vegetable production stages, indicating contact of these foods with human and animal waste. This foments human infection by pathogenic agents, since the main decontaminating solutions used for washing vegetables, such as sodium hypochlorite and acetic acid, act only on bacteria (Karapinar & Segun, 2007).

As observed in the funnel plot, there was a tendency of agglomeration in parasite’s frequency among the studies because a huge part of the results is concentrated out-right of the confidence interval of meta-analysis. This publication bias may have been caused by higher frequencies in studies with small n (n<100) and high standard deviation. The lack of standard methodology for seeking parasites in vegetables is also a contributor to this frequency variation and might be an important factor for heterogeneity value presented by the meta-analysis, as countries with higher HDI can search and publish more. These error factors make comparison among studies not feasible.

Although studies were highly heterogeneous and presented publication bias, differences in people's habits and agriculture might explain these frequency variations among studies, as the random effects model demonstrated significance in country and sample origin subgroups. It might happen because there are different sanitary legislations among countries and in some sample origins such as vegetable gardens, there are no specific standards of growing food or verification of quality and safety in these products. Probably, these two variables, country, and sample origin, also contributed to frequency variation which was found in this review.

## Conclusion

The consumption of vegetables, especially when raw, represents a great epidemiological importance for the transmission of pathogenic enteroparasites to humans. Parasitic contamination was observed, globally, in various cultivation and vegetable types, as well as in different places of origin, indicating that hygiene is deficient in all production process stages, whether in the field or in sales establishments.

## Supplementary Material

Supplementary material accompanies this paper.

Spreadsheet S1 Studies included in the systematic review.

Figure S1 Fixed and Random Effects Models applied to systematic review studies.

References S1 References used in the systematic review and meta-analysis.

This material is available as part of the online article from https://doi.org/10.1590/S1984-29612024040
