# Supplementary material for: Parasitic contamination in vegetables for human consumption: a systematic review and meta-analysis
Source: Rev Bras Parasitol Vet. 2024 Sep 6;33(3):e002824. doi: 10.1590/S1984-29612024040 (PMC11452068; doi:10.1590/S1984-29612024040)
Supplement: Spreadsheet S1 [file rbpv-33-3-e002824-Suppl-S01.pdf]

Spreadsheet S1: Studies included in the systematic review.

AUTHORS	YEAR OF PUBLICATION
A. Bafghi et al	2020
A. Bafghi et al	2020
A. Bafghi et al	2020
A. Bafghi et al	2020
A. Bafghi et al	2020
A. Bafghi et al	2020
A. Bafghi et al	2020
A. Bafghi et al	2020
A. Bafghi et al	2020
A. Daryani et al.	2008
A. Daryani et al.	2008
A. Daryani et al.	2008
A. Daryani et al.	2008
A. Daryani et al.	2008
A. Daryani et al.	2008
A. Daryani et al.	2008
A. Daryani et al.	2008
A. Daryani et al.	2008
A. Daryani et al.	2008
A. Daryani et al.	2008
A. Daryani et al.	2008
A. Daryani et al.	2008
A. Daryani et al.	2008
A. Fallah et al	2016
A. Fallah et al	2016
A. Fallah et al	2016
A. Fallah et al	2016
A. Fallah et al	2016
A. Fallah et al	2016
A. Fallah et al	2016
A. Fallah et al	2016
A. Fallah et al	2016
A. Fallah et al	2016
A. Fallah et al	2016
A. Fallah et al	2016
A. Fallah et al	2016
A. Fallah et al	2016
A. Fallah et al	2016
A. Fallah et al	2016
A. Fallah et al	2016
A. Fallah et al	2016
A. Fallah et al	2016
A. Fallah et al	2016
A. Fallah et al	2016
A. Fallah et al	2016
A. Fallah et al	2016
A. Fallah et al	2016
A. Fallah et al	2016
A. Fallah et al	2016

A. Fallah et al	2016
A. Fallah et al	2016
A. Fallah et al	2016
A. Fallah et al	2016
A. Fallah et al	2016
A. Fallah et al	2016
A. Fallah et al	2016
A. Fallah et al	2016
A. Fallah et al	2016
A. Fallah et al	2016
A. Fallah et al	2016
A. Fallah et al	2016
A. Fallah et al	2016
A. Fallah et al	2016
A. Fallah et al	2016
A. Fallah et al	2016
A. Fallah et al	2016
A. Fallah et al	2016
A. Fallah et al	2016
A. Fallah et al	2016
A. Fallah et al	2016
A. Fallah et al	2016
A. Fallah et al	2016
A. Fallah et al	2016
A. Fallah et al	2016
A. Fallah et al	2016
A. Fallah et al	2016
A. Fallah et al	2016
A. Fallah et al	2016
A. Fallah et al	2016
A. Fallah et al	2016
A. Fallah et al	2016
A. Fallah et al	2016
A. Fallah et al	2016
A. Fallah et al	2016
A. Fallah et al	2016
A. Fallah et al	2016
A. Fallah et al	2016
A. Fallah et al	2016
A. Fallah et al	2016
A. Fallah et al	2016
A. Fallah et al	2016
A. Fallah et al	2016
A. Fallah et al	2016
A. Fallah et al	2016
A. Fallah et al	2016
A. Fallah et al	2016
A. Fallah et al	2016
A. Fallah et al	2016
A. Fallah et al	2016
A. Fallah et al	2016
A. Fallah et al	2016
A. Fallah et al	2016
A. Fallah et al	2016
A. Fallah et al	2016
A. Fallah et al	2016
A. Fallah et al	2016
A. Fallah et al	2016
A. Fallah et al	2016

A. Fallah et al	2016
A. Fallah et al	2016
A. Fallah et al	2016
A. Fallah et al	2016
A. Fallah et al	2016
A. Fallah et al	2016
A. Fallah et al	2016
A. Fallah et al	2016
A. Fallah et al	2016
A. Fallah et al	2016
A. Fallah et al	2016
A. Fallah et al	2016
A. Filho et al.	2018
A. Filho et al.	2018
A. Filho et al.	2018
A. Filho et al.	2018
A. Filho et al.	2018
A. Filho et al.	2018
A. Filho et al.	2018
A. Filho et al.	2018
A. Filho et al.	2018
A. Filho et al.	2018
A. Filho et al.	2018
A. Filho et al.	2018
A. Mohemeed et al	2021
A. Mohemeed et al	2021
A. Mohemeed et al	2021
A. Mohemeed et al	2021
A. Mohemeed et al	2021
A. Mohemeed et al	2021
A. Mohemeed et al	2021
A. Mohemeed et al	2021
A. Mohemeed et al	2021
A. Mohemeed et al	2021
A. Mohemeed et al	2021
A. Mohemeed et al	2021
A. Mohemeed et al	2021
A. Mohemeed et al	2021
A. Mohemeed et al	2021
A. Mohemeed et al	2021
A. Mohemeed et al	2021
A. Mohemeed et al	2021
A. Mohemeed et al	2021
A. Mohemeed et al	2021
A. Mohemeed et al	2021
A. Mohemeed et al	2021
A. Mohemeed et al	2021
A. Mohemeed et al	2021
A. Mohemeed et al	2021
A. Mohemeed et al	2021
A. Mohemeed et al	2021
A. Mohemeed et al	2021
A. Mohemeed et al	2021
A. Mohemeed et al	2021
A. Mohemeed et al	2021
A. Mohemeed et al	2021
A. Mohemeed et al	2021
A. Mohemeed et al	2021
A. Mohemeed et al	2021
A. Mohemeed et al	2021

A. Mohemeed et al	2021
A. Mohemeed et al	2021
A. Mohemeed et al	2021
A. Mohemeed et al	2021
A. Mohemeed et al	2021
A. Mohemeed et al	2021
A. Mohemeed et al	2021
A. Mohemeed et al	2021
A. Mohemeed et al	2021
A. Mohemeed et al	2021
A. Mohemeed et al	2021
A. Mohemeed et al	2021
A. Mohemeed et al	2021
A. Mohemeed et al	2021
A. Mohemeed et al	2021
A. Mohemeed et al	2021
A. Mohemeed et al	2021
A. Mohemeed et al	2021
A. Mohemeed et al	2021
A. Mohemeed et al	2021
A. Mohemeed et al	2021
A. Mohemeed et al	2021
A. Mohemeed et al	2021
A. Mohemeed et al	2021
A. Mohemeed et al	2021
A. Mohemeed et al	2021
A. Mohemeed et al	2021
A. Mohemeed et al	2021
A. Mohemeed et al	2021
A. Mohemeed et al	2021
A. Mohemeed et al	2021
A. Mohemeed et al	2021
A. Mohemeed et al	2021
A. Mohemeed et al	2021
A. Mohemeed et al	2021
A. Mohemeed et al	2021
A. Mohemeed et al	2021
A. Mohemeed et al	2021
A. Mohemeed et al	2021
A. Mohemeed et al	2021
A. Mohemeed et al	2021
A. Mohemeed et al	2021
A. Mohemeed et al	2021
A. Mohemeed et al	2021
A. Mohemeed et al	2021
A. Mohemeed et al	2021
A. Mohemeed et al	2021
A. Mohemeed et al	2021
A. Mohemeed et al	2021
A. Mohemeed et al	2021
A. Mohemeed et al	2021
A. Mohemeed et al	2021
A. Mohemeed et al	2021

A. Mohemeed et al	2021
A. Mohemeed et al	2021
A. Mohemeed et al	2021
A. Mohemeed et al	2021
A. Mohemeed et al	2021
A. Mohemeed et al	2021
A. Mohemeed et al	2021
A. Mohemeed et al	2021
A. Mohemeed et al	2021
A. Mohemeed et al	2021
A. Mohemeed et al	2021
A. Mohemeed et al	2021
A. Mohemeed et al	2021
A. Mohemeed et al	2021
A. Mohemeed et al	2021
A. Mohemeed et al	2021
A. Mohemeed et al	2021
A. Mohemeed et al	2021
A. Mohemeed et al	2021
A. Mohemeed et al	2021
A. Mohemeed et al	2021
A. Mohemeed et al	2021
A. Mohemeed et al	2021
A. Mohemeed et al	2021
A. Mohemeed et al	2021
A. Mohemeed et al	2021
A. Mohemeed et al	2021
A. Mohemeed et al	2021
A. Mohemeed et al	2021
A. Mohemeed et al	2021
A. Mohemeed et al	2021
A. Mohemeed et al	2021
A. Mohemeed et al	2021
A. Mohemeed et al	2021
A. Mohemeed et al	2021
A. Mohemeed et al	2021
A. Mohemeed et al	2021
A. Mohemeed et al	2021
A. Mohemeed et al	2021
A. Mohemeed et al	2021
A. Mohemeed et al	2021
A. Mohemeed et al	2021
A. Mohemeed et al	2021
A. Mohemeed et al	2021
A. Mohemeed et al	2021
A. Mohemeed et al	2021
A. Mohemeed et al	2021
A. Mohemeed et al	2021
A. Mohemeed et al	2021
A. Mohemeed et al	2021
A. Mohemeed et al	2021
A. Mohemeed et al	2021

A. Mohemeed et al	2021
A. Mohemeed et al	2021
A. Mohemeed et al	2021
A. Mohemeed et al	2021
A. Mohemeed et al	2021
A. Mohemeed et al	2021
A. Mohemeed et al	2021
A. Mohemeed et al	2021
A. Mohemeed et al	2021
A. Mohemeed et al	2021
A. Mohemeed et al	2021
A. Mohemeed et al	2021
A. Mohemeed et al	2021
A. Mohemeed et al	2021
A. Mohemeed et al	2021
A. Mohemeed et al	2021
A. Mohemeed et al	2021
A. Mohemeed et al	2021
A. Mohemeed et al	2021
A. Mohemeed et al	2021
A. Mohemeed et al	2021
A. Mohemeed et al	2021
A. Mohemeed et al	2021
A. Mohemeed et al	2021
A. Mohemeed et al	2021
A. Mohemeed et al	2021
A. Mohemeed et al	2021
A. Mohemeed et al	2021
A. Mohemeed et al	2021
A. Mohemeed et al	2021
A. Mohemeed et al	2021
A. Mohemeed et al	2021
A. Mohemeed et al	2021
A. Mohemeed et al	2021
A. Mohemeed et al	2021
A. Mohemeed et al	2021
A. Mohemeed et al	2021
A. Mohemeed et al	2021
A. Mohemeed et al	2021
A. Mohemeed et al	2021
A. Mohemeed et al	2021
A. Mohemeed et al	2021
A. Mohemeed et al	2021
A. Mohemeed et al	2021
A. Mohemeed et al	2021
A. Mohemeed et al	2021
A. Mohemeed et al	2021
A. Mohemeed et al	2021
A. Mohemeed et al	2021
A. Mohemeed et al	2021
A. Mohemeed et al	2021
A. Rostami et al	2016

A. Rostami et al	2016
A. Rostami et al	2016
A. Rostami et al	2016
A. Rostami et al	2016
A. Rostami et al	2016
A. Rostami et al	2016
A. Rostami et al	2016
A. Rostami et al	2016
A. Rostami et al	2016
A. Rostami et al	2016
A. Rostami et al	2016
A. Rostami et al	2016
A. Rostami et al	2016
A. Rostami et al	2016
A. Rostami et al	2016
A. Rostami et al	2016
A. Rostami et al	2016
A. Rostami et al	2016
A. Rostami et al	2016
A. Rostami et al	2016
A. Rostami et al	2016
A. Rostami et al	2016
A. Rostami et al	2016
A. Rostami et al	2016
A. Rostami et al	2016
A. Rostami et al	2016
A. Rostami et al	2016
A. Rostami et al	2016
A. Rostami et al	2016
A. Rostami et al	2016
A. Rostami et al	2016
A. Rostami et al	2016
A. Rostami et al	2016
A. Rostami et al	2016
A. Rostami et al	2016
A. Rostami et al	2016
A. Rostami et al	2016
A. Rostami et al	2016
A. Rostami et al	2016
A. Rostami et al	2016
A. Rostami et al	2016
A. Rostami et al	2016
A. Rostami et al	2016
A. Rostami et al	2016
A. Rostami et al	2016
A. Rostami et al	2016
A. Rostami et al	2016
A. Rostami et al	2016
Abdalla, M.A et al.	2013
Abdalla, M.A et al.	2013
Abdalla, M.A et al.	2013

Abdalla, M.A et al.	2013
Abdalla, M.A et al.	2013
Abdalla, M.A et al.	2013
Abdalla, M.A et al.	2013
Abdalla, M.A et al.	2013
Abdalla, M.A et al.	2013
Abdalla, M.A et al.	2013
Abdalla, M.A et al.	2013
Abdalla, M.A et al.	2013
Abdalla, M.A et al.	2013
Abdalla, M.A et al.	2013
Abe, E M et al.	2016
Abe, E M et al.	2016
Abe, E M et al.	2016
Abe, E M et al.	2016
Abe, E M et al.	2016
Abe, E M et al.	2016
Abe, E M et al.	2016
Abe, E M et al.	2016
Abe, E M et al.	2016
Abe, E M et al.	2016
Abe, E M et al.	2016
Abe, E M et al.	2016
Abe, E M et al.	2016
Abe, E M et al.	2016
Abe, E M et al.	2016
Abe, E M et al.	2016
Abe, E M et al.	2016
Abe, E M et al.	2016
Abe, E M et al.	2016
Abe, E M et al.	2016
Abe, E M et al.	2016
Abe, E M et al.	2016
Abe, E M et al.	2016
Abe, E M et al.	2016
Abe, E M et al.	2016
Abe, E M et al.	2016
Abe, E M et al.	2016
Abe, E M et al.	2016
Abe, E M et al.	2016
Abe, E M et al.	2016
Abe, E M et al.	2016
Abe, E M et al.	2016
Abe, E M et al.	2016
Abe, E M et al.	2016
Abe, E M et al.	2016
Abe, E M et al.	2016
Abe, E M et al.	2016
Abe, E M et al.	2016
Abe, E M et al.	2016

Abe, E M et al.	2016
Abougrain, A K et al.	2010
Abougrain, A K et al.	2010
Abougrain, A K et al.	2010
Abougrain, A K et al.	2010
Abougrain, A K et al.	2010
Abougrain, A K et al.	2010
Abougrain, A K et al.	2010
Abougrain, A K et al.	2010
Abougrain, A K et al.	2010
Abougrain, A K et al.	2010
Abougrain, A K et al.	2010
Abougrain, A K et al.	2010
Abougrain, A K et al.	2010
Abougrain, A K et al.	2010
Abougrain, A K et al.	2010
Abougrain, A K et al.	2010
Abougrain, A K et al.	2010
Abougrain, A K et al.	2010
Abougrain, A K et al.	2010
Abougrain, A K et al.	2010
Abougrain, A K et al.	2010
Abougrain, A K et al.	2010
Abougrain, A K et al.	2010
Abougrain, A K et al.	2010
Abougrain, A K et al.	2010
Abougrain, A K et al.	2010
Abougrain, A K et al.	2010
Abougrain, A K et al.	2010
Abougrain, A K et al.	2010
Abougrain, A K et al.	2010
Abougrain, A K et al.	2010
Abougrain, A K et al.	2010
Abougrain, A K et al.	2010
Abougrain, A K et al.	2010
Abougrain, A K et al.	2010
Abougrain, A K et al.	2010
Abougrain, A K et al.	2010
Abougrain, A K et al.	2010
Abougrain, A K et al.	2010
Abougrain, A K et al.	2010
Adamu N. B. et al	2012
Adamu N. B. et al	2012
Adamu N. B. et al	2012
Adamu N. B. et al	2012
Adamu N. B. et al	2012
Adamu N. B. et al	2012
Adamu N. B. et al	2012
Adamu N. B. et al	2012
Adamu N. B. et al	2012

Adanir, Ramazan; Tasci, Fulya	2013
Adanir, Ramazan; Tasci, Fulya	2013
Adanir, Ramazan; Tasci, Fulya	2013
Adanir, Ramazan; Tasci, Fulya	2013
Adanir, Ramazan; Tasci, Fulya	2013
Adanir, Ramazan; Tasci, Fulya	2013
Adanir, Ramazan; Tasci, Fulya	2013
Adanir, Ramazan; Tasci, Fulya	2013
Adanir, Ramazan; Tasci, Fulya	2013
Al Nahhas, Samar et al.	2020
Al Nahhas, Samar et al.	2020
Al Nahhas, Samar et al.	2020
Al Nahhas, Samar et al.	2020
Al Nahhas, Samar et al.	2020
Al Nahhas, Samar et al.	2020
Al Nahhas, Samar et al.	2020
Al Nahhas, Samar et al.	2020
Al Nahhas, Samar et al.	2020
Al Nahhas, Samar et al.	2020
Al Nahhas, Samar et al.	2020
Al Nahhas, Samar et al.	2020
Al Nahhas, Samar et al.	2020
Al Nahhas, Samar et al.	2020
Al Nahhas, Samar et al.	2020
Al Nahhas, Samar et al.	2020
Al Nahhas, Samar et al.	2020
Al Nahhas, Samar et al.	2020
Al Nahhas, Samar et al.	2020
Al Nahhas, Samar et al.	2020
Al Nahhas, Samar et al.	2020
Al Nahhas, Samar et al.	2020
Al Nahhas, Samar et al.	2020
Al Nahhas, Samar et al.	2020
Al Nahhas, Samar et al.	2020
Al Nahhas, Samar et al.	2020
Al Nahhas, Samar et al.	2020
Al Nahhas, Samar et al.	2020
Al Nahhas, Samar et al.	2020
Al Nahhas, Samar et al.	2020
Al Nahhas, Samar et al.	2020
Al Nahhas, Samar et al.	2020
Al Nahhas, Samar et al.	2020
Al Nahhas, Samar et al.	2020
Al Nahhas, Samar et al.	2020
Al Nahhas, Samar et al.	2020
Al Nahhas, Samar et al.	2020
Al Nahhas, Samar et al.	2020
Al Nahhas, Samar et al.	2020
Al Nahhas, Samar et al.	2020

Al Nahhas, Samar et al.	2020
Al Nahhas, Samar et al.	2020
Al Nahhas, Samar et al.	2020
Al Nahhas, Samar et al.	2020
Al Nahhas, Samar et al.	2020
Al Nahhas, Samar et al.	2020
Al Nahhas, Samar et al.	2020
Al Nahhas, Samar et al.	2020
Al Nahhas, Samar et al.	2020
Al Nahhas, Samar et al.	2020
Al Nahhas, Samar et al.	2020
Al Nahhas, Samar et al.	2020
Al Nahhas, Samar et al.	2020
Al Nahhas, Samar et al.	2020
Al Nahhas, Samar et al.	2020
Al-Binali et al	2006
Al-Binali et al	2006
Al-Binali et al	2006
Al-Binali et al	2006
Al-Binali et al	2006
Al-Binali et al	2006
Al-Binali et al	2006
Al-Binali et al	2006
Al-Binali et al	2006
Al-Binali et al	2006
Al-Binali et al	2006
Al-Binali et al	2006
Al-Binali et al	2006
Al-Binali et al	2006
Al-Binali et al	2006
Al-Binali et al	2006
Al-Binali et al	2006
Al-Binali et al	2006
Al-Binali et al	2006
Al-Binali et al	2006
Al-Binali et al	2006
Al-Binali et al	2006
Al-Binali et al	2006
Al-Binali et al	2006
Al-Binali et al	2006
Al-Binali et al	2006
Al-Binali et al	2006
Al-Binali et al	2006
Al-Binali et al	2006
Al-Binali et al	2006
Al-Binali et al	2006
Al-Binali et al	2006
Alemu, Getaneh et al.	2019
Alemu, Getaneh et al.	2019
Alemu, Getaneh et al.	2019

Alemu, Getaneh et al.	2019
Alemu, Getaneh et al.	2019
Alemu, Getaneh et al.	2019
Alemu, Getaneh et al.	2019
Alemu, Getaneh et al.	2019
Alemu, Getaneh et al.	2019
Alemu, Getaneh et al.	2019
Alemu, Getaneh et al.	2019
Alemu, Getaneh et al.	2019
Alemu, Getaneh et al.	2019
Alemu, Getaneh et al.	2019
Alemu, Getaneh et al.	2019
Alemu, Getaneh et al.	2019
Alemu, Getaneh et al.	2019
Alemu, Getaneh et al.	2019
Alemu, Getaneh et al.	2019
Alemu, Getaneh et al.	2019
Alemu, Getaneh et al.	2019
Alemu, Getaneh et al.	2019
Alemu, Getaneh et al.	2019
Alemu, Getaneh et al.	2019
Alemu, Getaneh et al.	2019
Alemu, Getaneh et al.	2019
Alemu, Getaneh et al.	2019
Alemu, Getaneh et al.	2019
Alemu, Getaneh et al.	2019
Alemu, Getaneh et al.	2019
Alemu, Getaneh et al.	2019
Alemu, Getaneh et al.	2019
Alemu, Getaneh et al.	2019
Alemu, Getaneh et al.	2019
Alemu, Getaneh et al.	2019
Alemu, Getaneh et al.	2019
Alemu, Getaneh et al.	2019
Alemu, Getaneh et al.	2019
Alemu, Getaneh et al.	2019
Alemu, Getaneh et al.	2019
Alhabbal, A T	2015
Alhabbal, A T	2015
Alhabbal, A T	2015
Alhabbal, A T	2015
Alhabbal, A T	2015
Alhabbal, A T	2015
Alhabbal, A T	2015
Alhabbal, A T	2015
Alhabbal, A T	2015
Alhabbal, A T	2015
Alhabbal, A T	2015
Alhabbal, A T	2015
Alhabbal, A T	2015

Alhabbal, A T	2015
Alhabbal, A T	2015
Alhabbal, A T	2015
Alhabbal, A T	2015
Alhabbal, A T	2015
Alhabbal, A T	2015
Alhabbal, A T	2015
Ali El Bakri, et al.	2020
Ali El Bakri, et al.	2020
Ali El Bakri, et al.	2020
Ali El Bakri, et al.	2020
Ali El Bakri, et al.	2020
Ali El Bakri, et al.	2020
Ali El Bakri, et al.	2020
Ali El Bakri, et al.	2020
Ali El Bakri, et al.	2020
Ali El Bakri, et al.	2020
Ali El Bakri, et al.	2020
Ali El Bakri, et al.	2020
Ali El Bakri, et al.	2020
Ali El Bakri, et al.	2020
Ali El Bakri, et al.	2020
Ali El Bakri, et al.	2020
Ali El Bakri, et al.	2020
Ali El Bakri, et al.	2020
Ali El Bakri, et al.	2020
Ali El Bakri, et al.	2020
Ali El Bakri, et al.	2020
Ali El Bakri, et al.	2020
Ali El Bakri, et al.	2020
Ali El Bakri, et al.	2020
Ali El Bakri, et al.	2020
Ali El Bakri, et al.	2020
Ali El Bakri, et al.	2020
Al-Megrin, W. A. I.	2010
Al-Megrin, W. A. I.	2010
Al-Megrin, W. A. I.	2010
Al-Megrin, W. A. I.	2010
Al-Megrin, W. A. I.	2010
Al-Megrin, W. A. I.	2010
Al-Megrin, W. A. I.	2010
Al-Megrin, W. A. I.	2010
Al-Megrin, W. A. I.	2010
Al-Megrin, W. A. I.	2010
Al-Megrin, W. A. I.	2010
Ambrozim, et al	2017
Ambrozim, et al	2017
Ambrozim, et al	2017
Ambrozim, et al	2017
Ambrozim, et al	2017

Ambrozim, et al	2017
Ambrozim, et al	2017
Ambrozim, et al	2017
Ambrozim, et al	2017
Ambrozim, et al	2017
Ambrozim, et al	2017
Ambrozim, et al	2017
Ambrozim, et al	2017
Ambrozim, et al	2017
Ambrozim, et al	2017
Ambrozim, et al	2017
Ambrozim, et al	2017
Ambrozim, et al	2017
Ambrozim, et al	2017
Ambrozim, et al	2017
Ambrozim, et al	2017
Ambrozim, et al	2017
Ambrozim, et al	2017
Amoros, I et al.	2010
Amoros, I et al.	2010
Amoros, I et al.	2010
Amoros, I et al.	2010
Amoros, I et al.	2010
Amoros, I et al.	2010
Amuta et al.	2017
Amuta et al.	2017
Amuta et al.	2017
Amuta et al.	2017
Amuta et al.	2017
Amuta et al.	2017
Amuta et al.	2017
Amuta et al.	2017
Amuta et al.	2017
Amuta et al.	2017
Amuta et al.	2017
Amuta et al.	2017
Amuta et al.	2017
Amuta et al.	2017
Anh, V T et al.	2007
Anh, V T et al.	2007
Anh, V T et al.	2007
Anh, V T et al.	2007
Arbos, K A et al.	2010
Arbos, K A et al.	2010
Arbos, K A et al.	2010
Arbos, K A et al.	2010
Arbos, K A et al.	2010
Arbos, K A et al.	2010
Arbos, K A et al.	2010
Arbos, K A et al.	2010

Arbos, K A et al.	2010
Arbos, K A et al.	2010
Arbos, K A et al.	2010
Arbos, K A et al.	2010
Arbos, K A et al.	2010
Arbos, K A et al.	2010
Arbos, K A et al.	2010
Ashrafi, K et al.	2004
Ashrafi, K et al.	2004
Ashrafi, K et al.	2004
Ashrafi, K et al.	2004
Ashrafi, K et al.	2004
Ashrafi, K et al.	2004
Ashrafi, K et al.	2004
Ashrafi, K et al.	2004
Avazpoor et al.	2015
Avazpoor et al.	2015
Avazpoor et al.	2015
Avazpoor et al.	2015
B. Ejike et al	2018
B. Ejike et al	2018
B. Ejike et al	2018
B. Ejike et al	2018
B. Ejike et al	2018
B. Ejike et al	2018
B. Ejike et al	2018
B. Ejike et al	2018
B. Ejike et al	2018
B. Ejike et al	2018
B. Ejike et al	2018
B. Ejike et al	2018
B. Ejike et al	2018
B. Ejike et al	2018
B. Ejike et al	2018
B. Ejike et al	2018
B. Ejike et al	2018
B. Ejike et al	2018
B. Ejike et al	2018
B. Ejike et al	2018
B. Ejike et al	2018
B. Ejike et al	2018
B. Ejike et al	2018
B. Ejike et al	2018
B. Ejike et al	2018
B. Ejike et al	2018
B. Ejike et al	2018
B. Ejike et al	2018
B. Ejike et al	2018
B. Ejike et al	2018
B. Ejike et al	2018

B. Ejike et al	2018
B. Ejike et al	2018
B. Ejike et al	2018
B. Ejike et al	2018
B. Ejike et al	2018
B. Ejike et al	2018
B. Ejike et al	2018
B. Ejike et al	2018
B. Ejike et al	2018
B. Maikai et al	2012
B. Maikai et al	2012
B. Maikai et al	2012
B. Maikai et al	2012
B. Maikai et al	2012
B. Maikai et al	2012
B.Sunil et al.	2014
B.Sunil et al.	2014
B.Sunil et al.	2014
B.Sunil et al.	2014
B.Sunil et al.	2014
B.Sunil et al.	2014
B.Sunil et al.	2014
B.Sunil et al.	2014
B.Sunil et al.	2014
B.Sunil et al.	2014
Bekele, F at al.	2017
Bekele, F at al.	2017
Bekele, F at al.	2017
Bekele, F at al.	2017
Bekele, F at al.	2017
Bekele, F at al.	2017
Bekele, F at al.	2017
Bekele, F at al.	2017
Bekele, F at al.	2017
Bekele, Fitsum et al.	2019
Bekele, Fitsum et al.	2019
Bekele, Fitsum et al.	2019
Bekele, Fitsum et al.	2019
Bekele, Fitsum et al.	2019
Bekele, Fitsum et al.	2019
Bekele, Fitsum et al.	2019
Bekele, Fitsum et al.	2019
Bekele, Fitsum et al.	2019
Bekele, Fitsum et al.	2019
Bekele, Fitsum et al.	2019
Bekele, Fitsum et al.	2019
Bekele, Fitsum et al.	2019
Bekele, Fitsum et al.	2019
Bekele, Fitsum et al.	2019
Bekele, Fitsum et al.	2019
Bekele, Fitsum et al.	2019
Bekele, Fitsum et al.	2019
Bekele, Fitsum et al.	2019
Bekele, Fitsum et al.	2019
Bekele, Fitsum et al.	2019
Bekele, Fitsum et al.	2019

Bekele, Fitsum et al.	2019
Bekele, Fitsum et al.	2019
Bekele, Fitsum et al.	2019
Bekele, Fitsum et al.	2019
Bekele, Fitsum et al.	2019
Bekele, Fitsum et al.	2019
Bekele, Fitsum et al.	2019
Bekele, Fitsum et al.	2019
Bekele, Fitsum et al.	2019
Bekele, Fitsum et al.	2019
Bekele, Fitsum et al.	2019
Bekele, Fitsum et al.	2019
Bekele, Fitsum et al.	2019
Bekele, Fitsum et al.	2019
Bekele, Fitsum et al.	2019
Bekele, Fitsum et al.	2019
Bekele, Fitsum et al.	2019
Bekele, Fitsum et al.	2019
Bekele, Fitsum et al.	2019
Bekele, Fitsum et al.	2019
Berrouch, S et al.	2020
Berrouch, S et al.	2020
Berrouch, S et al.	2020
Berrouch, S et al.	2020
Berrouch, S et al.	2020
Berrouch, S et al.	2020
Berrouch, S et al.	2020
Berrouch, S et al.	2020
Berrouch, S et al.	2020
Berrouch, S et al.	2020
Berrouch, S et al.	2020
C. Marques et al.	2020
C. Marques et al.	2020
C. Marques et al.	2020
C. Marques et al.	2020
C. Marques et al.	2020
C. Marques et al.	2020
C. Marques et al.	2020
C. Marques et al.	2020
C. Marques et al.	2020
C. Marques et al.	2020
C. Marques et al.	2020
C. Marques et al.	2020
C. Marques et al.	2020
C. Marques et al.	2020
C. Marques et al.	2020
C. Marques et al.	2020
C. Marques et al.	2020
C. Marques et al.	2020
C. Marques et al.	2020
C. Marques et al.	2020

C. Punsawad et al.	2019
C. Punsawad et al.	2019
C. Punsawad et al.	2019
C. Punsawad et al.	2019
C. Punsawad et al.	2019
C. Punsawad et al.	2019
C. Punsawad et al.	2019
C. Punsawad et al.	2019
C. Punsawad et al.	2019
C. Punsawad et al.	2019
C. Punsawad et al.	2019
C. Punsawad et al.	2019
C. Punsawad et al.	2019
C. Punsawad et al.	2019
C. Punsawad et al.	2019
C. Punsawad et al.	2019
C. Punsawad et al.	2019
C. Punsawad et al.	2019
C. Punsawad et al.	2019
C. Punsawad et al.	2019
C. Punsawad et al.	2019
C. Punsawad et al.	2019
C. Punsawad et al.	2019
C. Punsawad et al.	2019
C. Punsawad et al.	2019
C. Punsawad et al.	2019
C. Punsawad et al.	2019
C. Punsawad et al.	2019
C. Punsawad et al.	2019
Carzola et al	2009
Carzola et al	2009
Carzola et al	2009
Carzola et al	2009
Carzola et al	2009
Carzola et al	2009
Carzola et al	2009
Carzola et al	2009
Carzola et al	2009
Carzola et al	2009
Carzola et al	2009
Carzola et al	2009
Carzola et al	2009
Carzola et al	2009
Carzola et al	2009
Carzola et al	2009
Carzola et al	2009
Carzola et al	2009
Carzola et al	2009
Carzola et al	2009
Carzola et al	2009

Carzola et al	2009
Carzola et al	2009
Carzola et al	2009
Carzola et al	2009
Carzola et al	2009
Carzola et al	2009
Carzola et al	2009
Carzola et al	2009
Carzola et al	2009
Carzola et al	2009
Carzola et al	2009
Carzola et al	2009
Carzola et al	2009
Carzola et al	2009
Carzola et al	2009
Carzola et al	2009
Carzola et al	2009
Carzola et al	2009
Carzola et al	2009
Carzola et al	2009
Carzola et al	2009
Carzola et al	2009
Carzola et al	2009
Carzola et al	2009
Carzola et al	2009
Carzola et al	2009
Carzola et al	2009
Carzola et al	2009
Carzola et al	2009
Carzola et al	2009
Carzola et al	2009
Carzola et al	2009
Carzola et al	2009
Carzola et al	2009
Carzola et al	2009
Carzola et al	2009
Carzola et al	2009
Carzola et al	2009
Carzola et al	2009
Carzola et al	2009
Carzola et al	2009
Carzola et al	2009
Carzola et al	2009
Carzola et al	2009
Carzola et al	2009
Carzola et al	2009
Carzola et al	2009
Carzola et al	2009
Carzola et al	2009
Carzola et al	2009
Carzola et al	2009
Carzola et al	2009

Carzola et al	2009
Carzola et al	2009
Carzola et al	2009
Carzola et al	2009
Carzola et al	2009
Carzola et al	2009
Carzola et al	2009
Carzola et al	2009
Carzola et al	2009
Carzola et al	2009
Carzola et al	2009
Carzola et al	2009
Carzola et al	2009
Carzola et al	2009
Carzola et al	2009
Carzola et al	2009
Carzola et al	2009
Colli et al.	2015
Colli et al.	2015
D. El Said Said et al.	2012
D. El Said Said et al.	2012
D. El Said Said et al.	2012
D. El Said Said et al.	2012
D. El Said Said et al.	2012
D. El Said Said et al.	2012
D. El Said Said et al.	2012
D. El Said Said et al.	2012
D. El Said Said et al.	2012
D. El Said Said et al.	2012
D. El Said Said et al.	2012
D. El Said Said et al.	2012
D. El Said Said et al.	2012
D. El Said Said et al.	2012
D. El Said Said et al.	2012
D. El Said Said et al.	2012
D. El Said Said et al.	2012
D. El Said Said et al.	2012
D. El Said Said et al.	2012
D. El Said Said et al.	2012
D. El Said Said et al.	2012
D. El Said Said et al.	2012
D. El Said Said et al.	2012
D. El Said Said et al.	2012
D. El Said Said et al.	2012
D. El Said Said et al.	2012
D. El Said Said et al.	2012
D. El Said Said et al.	2012
D. El Said Said et al.	2012
D. El Said Said et al.	2012
D. El Said Said et al.	2012
D. El Said Said et al.	2012
Da Silva et al.	2007

Dardona, Z et al.	2021
Dardona, Z et al.	2021
Dardona, Z et al.	2021
Dardona, Z et al.	2021
Dardona, Z et al.	2021
Dardona, Z et al.	2021
Dardona, Z et al.	2021
Dardona, Z et al.	2021
Dardona, Z et al.	2021
Dardona, Z et al.	2021
Dardona, Z et al.	2021
Dardona, Z et al.	2021
Dixon et al.	2012
Dixon et al.	2012
Dixon et al.	2012
Eraky, Maysa Ahmad et al.	2014
Eraky, Maysa Ahmad et al.	2014
Eraky, Maysa Ahmad et al.	2014
Eraky, Maysa Ahmad et al.	2014
Eraky, Maysa Ahmad et al.	2014
Eraky, Maysa Ahmad et al.	2014
Eraky, Maysa Ahmad et al.	2014
Eraky, Maysa Ahmad et al.	2014
Eraky, Maysa Ahmad et al.	2014
Eraky, Maysa Ahmad et al.	2014
Eraky, Maysa Ahmad et al.	2014
Eraky, Maysa Ahmad et al.	2014
Eraky, Maysa Ahmad et al.	2014
Eraky, Maysa Ahmad et al.	2014
Eraky, Maysa Ahmad et al.	2014
Eraky, Maysa Ahmad et al.	2014
Eraky, Maysa Ahmad et al.	2014
Eraky, Maysa Ahmad et al.	2014
Eraky, Maysa Chmad et al.	2014
Eraky, Maysa Chmad et al.	2014
Eraky, Maysa Chmad et al.	2014
Eraky, Maysa Chmad et al.	2014
Eraky, Maysa Chmad et al.	2014
Eraky, Maysa Chmad et al.	2014
Eraky, Maysa Shmad et al.	2014
Eraky, Maysa Shmad et al.	2014
Eraky, Maysa Shmad et al.	2014
Eraky, Maysa Shmad et al.	2014
Eraky, Maysa Shmad et al.	2014
Eraky, Maysa Shmad et al.	2014
Erdogrul, O et al.	2005
Erdogrul, O et al.	2005
Erdogrul, O et al.	2005
Erdogrul, O et al.	2005
Erdogrul, O et al.	2005
Erdogrul, O et al.	2005
Erdogrul, O et al.	2005
Erdogrul, O et al.	2005

Erdogrul, O et al.	2005
Erdogrul, O et al.	2005
Erdogrul, O et al.	2005
Erdogrul, O et al.	2005
Erdogrul, O et al.	2005
Erdogrul, O et al.	2005
Erdogrul, O et al.	2005
Erdogrul, O et al.	2005
Erdogrul, O et al.	2005
Erdogrul, O et al.	2005
Erdogrul, O et al.	2005
Erdogrul, O et al.	2005
Ezatpour, B et al.	2013
Ezatpour, B et al.	2013
Ezatpour, B et al.	2013
Ezatpour, B et al.	2013
Ezatpour, B et al.	2013
Ezatpour, B et al.	2013
Ezatpour, B et al.	2013
Ezatpour, B et al.	2013
Ezatpour, B et al.	2013
Ezatpour, B et al.	2013
Ezatpour, B et al.	2013
Ezatpour, B et al.	2013
Ezatpour, B et al.	2013
Ezatpour, B et al.	2013
Ezatpour, B et al.	2013
Ezatpour, B et al.	2013
Ezatpour, B et al.	2013
Ezatpour, B et al.	2013
Ezatpour, B et al.	2013
Ezatpour, B et al.	2013
Ezatpour, B et al.	2013
Ezatpour, B et al.	2013
Ezatpour, B et al.	2013
Ezatpour, B et al.	2013
Ezatpour, B et al.	2013
Ezatpour, B et al.	2013
Ezatpour, B et al.	2013
Ezatpour, B et al.	2013
Ezatpour, B et al.	2013
Ezatpour, B et al.	2013
Ezatpour, B et al.	2013
Ezatpour, B et al.	2013
Ezatpour, B et al.	2013
Ezatpour, B et al.	2013
Ezatpour, B et al.	2013
Ezatpour, B et al.	2013
Ezatpour, B et al.	2013
Ezatpour, B et al.	2013
Ezatpour, B et al.	2013
Ezatpour, B et al.	2013
F. Bekele et al.	2020

F. Bekele et al.	2020
F. Bekele et al.	2020
F. Bekele et al.	2020
F. Bekele et al.	2020
F. Bekele et al.	2020
F. Bekele et al.	2020
Fallah, A A et al.	2012
Fallah, A A et al.	2012
Fallah, A A et al.	2012
Fallah, A A et al.	2012
Fallah, A A et al.	2012
Fallah, A A et al.	2012
Fallah, A A et al.	2012
Fallah, A A et al.	2012
Fallah, A A et al.	2012
Fallah, A A et al.	2012
Fallah, A A et al.	2012
Fallah, A A et al.	2012
Fallah, A A et al.	2012
Fallah, A A et al.	2012
Fallah, A A et al.	2012
Fallah, A A et al.	2012
Fallah, A A et al.	2012
Fallah, A A et al.	2012
Fallah, A A et al.	2012
Fallah, A A et al.	2012
Fallah, A A et al.	2012
Fallah, A A et al.	2012
Fallah, A A et al.	2012
Fallah, A A et al.	2012
Fallah, A A et al.	2012
Fallah, A A et al.	2012
Fallah, A A et al.	2012
Fallah, A A et al.	2012
Fallah, A A et al.	2012
Fallah, A A et al.	2012
Fallah, A A et al.	2012
Fallah, A A et al.	2012
Fallah, A A et al.	2012
Fallah, A A et al.	2012
Fallah, A A et al.	2012
Fallah, A A et al.	2012
Fallah, A A et al.	2012
Fallah, A A et al.	2012
Fallah, A A et al.	2012
Fallah, A A et al.	2012
Fallah, A A et al.	2012
Fallah, A A et al.	2012
Fallah, A A et al.	2012
Fallah, A A et al.	2012
Fallah, A A et al.	2012
Fallah, A A et al.	2012
Fallah, A A et al.	2012
Fallah, A A et al.	2012

Fallah, A A et al.	2012
Fallah, A A et al.	2012
Fallah, A A et al.	2012
Fallah, A A et al.	2012
Fallah, A A et al.	2012
Fallah, A A et al.	2012
Fallah, A A et al.	2012
Fallah, A A et al.	2012
Fallah, A A et al.	2012
Fallah, A A et al.	2012
Fallah, A A et al.	2012
Fallah, A A et al.	2012
Fallah, A A et al.	2012
Fallah, A A et al.	2012
Fallah, A A et al.	2012
Fallah, A A et al.	2012
Fallah, A A et al.	2012
Fallah, A A et al.	2012
Ferrelran et al.,	2018
Ferrelran et al.,	2018
Ferrelran et al.,	2018
Ferrelran et al.,	2018
Ferrelran et al.,	2018
Ferrelran et al.,	2018
Ferrelran et al.,	2018
Ferrelran et al.,	2018
Ferrelran et al.,	2018
Ferrelran et al.,	2018
Gabre and Shakir	2016
Gabre and Shakir	2016
Gabre and Shakir	2016
Gabre and Shakir	2016
Gabre and Shakir	2016
Gabre and Shakir	2016
Gabre and Shakir	2016
Gabre and Shakir	2016
Gabre and Shakir	2016
Gabre and Shakir	2016
Gabre and Shakir	2016
Gabre and Shakir	2016
Gabre and Shakir	2016
Gabre and Shakir	2016
Gabre and Shakir	2016
Gabre and Shakir	2016
Gabre and Shakir	2016
Gabre and Shakir	2016
Gabre and Shakir	2016
Gabre and Shakir	2016
Gabre and Shakir	2016
Gabre and Shakir	2016
Gabre and Shakir	2016
Gabre and Shakir	2016
Gabre and Shakir	2016
Gabre and Shakir	2016
Gabre and Shakir	2016
Gabre and Shakir	2016
Gabre and Shakir	2016
Gabre and Shakir	2016

Gad, M A et al.	2020
Gad, M A et al.	2020
Gad, M A et al.	2020
Gad, M A et al.	2020
Gad, M A et al.	2020
Gad, M A et al.	2020
Gad, M A et al.	2020
Gad, M A et al.	2020
Gad, M A et al.	2020
Gad, M A et al.	2020
Gad, M A et al.	2020
Gad, M A et al.	2020
Gad, M A et al.	2020
Gad, M A et al.	2020
Gad, M A et al.	2020
Gad, M A et al.	2020
Gad, M A et al.	2020
Gad, M A et al.	2020
Gad, M A et al.	2020
Gad, M A et al.	2020
Gad, M A et al.	2020
Gad, M A et al.	2020
Gad, M A et al.	2020
Gad, M A et al.	2020
Gad, M A et al.	2020
Gad, M A et al.	2020
Gad, M A et al.	2020
Gad, M A et al.	2020
Gad, M A et al.	2020
Gad, M A et al.	2020
Gad, M A et al.	2020
Gad, M A et al.	2020
Gad, M A et al.	2020
Gad, M A et al.	2020
Gad, M A et al.	2020
Gad, M A et al.	2020
Gad, M A et al.	2020
Gad, M A et al.	2020
Gad, M A et al.	2020
Gad, M A et al.	2020
Gad, M A et al.	2020
Gad, M A et al.	2020
Gad, M A et al.	2020
Gad, M A et al.	2020
Gad, M A et al.	2020
Gad, M A et al.	2020
Gad, M A et al.	2020
Gad, M A et al.	2020
Gad, M A et al.	2020
Gad, M A et al.	2020
Gad, M A et al.	2020
Gad, M A et al.	2020
Gad, M A et al.	2020
Gad, M A et al.	2020
Glenn L Sai Su et al.	2012
Glenn L Sai Su et al.	2012
Glenn L Sai Su et al.	2012

Glenn L Sai Su et al.	2012
Glenn L Sai Su et al.	2012
Glenn L Sai Su et al.	2012
Glenn L Sai Su et al.	2012
Glenn L Sai Su et al.	2012
Glenn L Sai Su et al.	2012
Glenn L Sai Su et al.	2012
Glenn L Sai Su et al.	2012
Glenn L Sai Su et al.	2012
Glenn L Sai Su et al.	2012
Glenn L Sai Su et al.	2012
Gomes Neto, et al	2012
Gomes Neto, et al	2012
Gomes Neto, et al	2012
Gomes Neto, et al	2012
Gomes Neto, et al	2012
Gomes Neto, et al	2012
Gomes Neto, et al	2012
Gomes Neto, et al	2012
Gomes Neto, et al	2012
Gomes Neto, et al	2012
Gomes Neto, et al	2012
Gomes Neto, et al	2012
Gomes Neto, et al	2012
Gomes Neto, et al	2012
Gomes Neto, et al	2012
Gomes Neto, et al	2012
Gomes Neto, et al	2012
Gomes Neto, et al	2012
Gomes Neto, et al	2012
Gomes Neto, et al	2012
Gomes Neto, et al	2012
Gomes Neto, et al	2012
Gomes Neto, et al	2012
Gomes Neto, et al	2012
Gomes Neto, et al	2012
Gomes Neto, et al	2012
Gomes Neto, et al	2012
Gomes Neto, et al	2012
Gomes Neto, et al	2012
Gomes Neto, et al	2012
Guimarães M. A. et al	2003
Guimarães M. A. et al	2003
Guimarães M. A. et al	2003
Guimarães M. A. et al	2003
Guimarães M. A. et al	2003
Guimarães M. A. et al	2003
Guimarães M. A. et al	2003
Guimarães M. A. et al	2003
Guimarães M. A. et al	2003
Guimarães M. A. et al	2003
Guimarães M. A. et al	2003
Guimarães M. A. et al	2003
Guimarães M. A. et al	2003
Guimarães M. A. et al	2003
Guimarães M. A. et al	2003
Guimarães M. A. et al	2003
Guimarães M. A. et al	2003

Guimarães M. A. et al	2003
Guimarães M. A. et al	2003
Guimarães M. A. et al	2003
Guimarães M. A. et al	2003
Guimarães M. A. et al	2003
Guimarães M. A. et al	2003
H. Al-Mozan et al	2019
H. Al-Mozan et al	2019
H. Al-Mozan et al	2019
H. Al-Mozan et al	2019
H. Al-Mozan et al	2019
H. Al-Mozan et al	2019
H. Al-Mozan et al	2019
H. Al-Mozan et al	2019
H. Al-Mozan et al	2019
H. Al-Mozan et al	2019
H. Al-Mozan et al	2019
H. Al-Mozan et al	2019
H. Al-Mozan et al	2019
H. Al-Mozan et al	2019
H. Al-Mozan et al	2019
H. Al-Mozan et al	2019
H. Al-Mozan et al	2019
H. Al-Mozan et al	2019
H. Al-Mozan et al	2019
H. Al-Mozan et al	2019
H. Al-Mozan et al	2019
H. Khalil et al	2019
H. Khalil et al	2019
H. Khalil et al	2019
H. Khalil et al	2019
H. Khalil et al	2019
H. Khalil et al	2019
H. Khalil et al	2019
H. Khalil et al	2019
H. Khalil et al	2019
H. Khalil et al	2019
H. Khalil et al	2019
H. Khalil et al	2019
H. Khalil et al	2019
H. Khalil et al	2019
H. Khalil et al	2019
H. Khalil et al	2019
H. Khalil et al	2019
H. Khalil et al	2019
H. Khalil et al	2019
H. Khalil et al	2019
H. Khalil et al	2019
H. Khalil et al	2019
H. Khalil et al	2019
H. Khalil et al	2019
H. Khalil et al	2019
H. Khalil et al	2019
H. Khalil et al	2019
H. Khalil et al	2019
H. Khalil et al	2019
H. Khalil et al	2019
H. Khalil et al	2019

H. Khalil et al	2019
H. Khalil et al	2019
H. Khalil et al	2019
H. Khalil et al	2019
H. Khalil et al	2019
H. Khalil et al	2019
H. Khalil et al	2019
H. Khalil et al	2019
H. Khalil et al	2019
H. Khalil et al	2019
H. Khalil et al	2019
H. Khalil et al	2019
H. Khalil et al	2019
H. Khalil et al	2019
H. Khalil et al	2019
H. Khalil et al	2019
H. Khalil et al	2019
H. Khalil et al	2019
H. Khalil et al	2019
H. Khalil et al	2019
H. Khalil et al	2019
H. Khalil et al	2019
H. Khalil et al	2019
H. Khalil et al	2019
H. Khalil et al	2019
H. Khalil et al	2019
H. Khalil et al	2019
H. Khalil et al	2019
H. Khalil et al	2019
H. Sakkas et al	2020
H. Sakkas et al	2020
H. Sakkas et al	2020
H. Sakkas et al	2020
H. Sakkas et al	2020
H. Sakkas et al	2020
H. Sakkas et al	2020
H. Sakkas et al	2020
H. Sakkas et al	2020
H. Sakkas et al	2020
H. Sakkas et al	2020
H. Sakkas et al	2020
H. Sakkas et al	2020
H. Sakkas et al	2020
H. Sakkas et al	2020
H. Sakkas et al	2020
H. Sakkas et al	2020
H. Sakkas et al	2020
H. Sakkas et al	2020
H. Sakkas et al	2020
H. Sakkas et al	2020
H. Sakkas et al	2020
H. Sakkas et al	2020
H. Sakkas et al	2020
H. Sakkas et al	2020
H. Sakkas et al	2020
H. Sakkas et al	2020
H. Sakkas et al	2020
H. Sakkas et al	2020

H. Sakkas et al	2020
H. Sakkas et al	2020
H. Sakkas et al	2020
H. Sakkas et al	2020
H. Sakkas et al	2020
H. Sakkas et al	2020
H. Sakkas et al	2020
H. Sakkas et al	2020
H. Sakkas et al	2020
H. Sakkas et al	2020
H. Sakkas et al	2020
H. Sakkas et al	2020
H. Sakkas et al	2020
H. Sakkas et al	2020
H. Sakkas et al	2020
H. Sakkas et al	2020
H. Sakkas et al	2020
H. Sakkas et al	2020
H. Sakkas et al	2020
H. Sakkas et al	2020
H. Sakkas et al	2020
H. Sakkas et al	2020
H. Sakkas et al	2020
H. Sakkas et al	2020
H. Sakkas et al	2020
Hajipour, N et al.	2020
Hajipour, N et al.	2020
Hajipour, N et al.	2020
Hajipour, N et al.	2020
Hajipour, N et al.	2020
Hajipour, N et al.	2020
Hajipour, N et al.	2020
Hajipour, N et al.	2020
Hajipour, N et al.	2020
Hajipour, N et al.	2020
Hajipour, N et al.	2020
Hajipour, N et al.	2020
Hajipour, N et al.	2020
Hajipour, N et al.	2020
Hajipour, N et al.	2020
Hajipour, N et al.	2020
Hajipour, N et al.	2020
Hajipour, N et al.	2020
Hajipour, N et al.	2020
Hajipour, N et al.	2020
Hajipour, N et al.	2020
Hajipour, N et al.	2020
Hajipour, N et al.	2020
Hajipour, N et al.	2020
Hajipour, N et al.	2020
Hajipour, N et al.	2020
Hajipour, N et al.	2020
Hajipour, N et al.	2020
Hajipour, N et al.	2020
Hajipour, N et al.	2020
Hajipour, N et al.	2020
Hajipour, N et al.	2020
Hajipour, N et al.	2020

Hajipour, N et al.	2020
Hajipour, N et al.	2020
Hajipour, N et al.	2020
Hajipour, N et al.	2020
Hajipour, N et al.	2020
Hajipour, N et al.	2020
Hajipour, N et al.	2020
Hajipour, N et al.	2020
Hajipour, N et al.	2020
Hajipour, N et al.	2020
Hajipour, N et al.	2020
Hajipour, N et al.	2020
Hajipour, N et al.	2020
Hajipour, N et al.	2020
Hajipour, N et al.	2020
Hajipour, N et al.	2020
Hajipour, N et al.	2020
Hajipour, N et al.	2020
Hajipour, N et al.	2020
Hajipour, N et al.	2020
Hajipour, N et al.	2020
Hajipour, N et al.	2020
Hajipour, N et al.	2020
Hajipour, N et al.	2020
Hajipour, N et al.	2020
Hajipour, N et al.	2020
Hajipour, N et al.	2020
Hajipour, N et al.	2020
Hajipour, N et al.	2020
Hajipour, N et al.	2020
Hajipour, N et al.	2020
Hajipour, N et al.	2020
Hajipour, N et al.	2020
Hajipour, N et al.	2020
Hajipour, N et al.	2020
Hajipour, N et al.	2020
Hajipour, N et al.	2020
Hajipour, N et al.	2020
Hajipour, N et al.	2020
Hajipour, N et al.	2020
Hajipour, N et al.	2020
Hajipour, N et al.	2020
Hajipour, N et al.	2020
Hajipour, N et al.	2020
Hajipour, N et al.	2020
Hajipour, N et al.	2020
Hajipour, N et al.	2020
Hajipour, N et al.	2020
Hajipour, N et al.	2020
Hajipour, N et al.	2020
Hajipour, N et al.	2020
Hajipour, N et al.	2020
Hajipour, N et al.	2020
Hajipour, N et al.	2020
Hajipour, N et al.	2020
Hajipour, N et al.	2020
Hajipour, N et al.	2020
Hajipour, N et al.	2020

Hajipour, N et al.	2020
Hajipour, N et al.	2020
Hajipour, N et al.	2020
Hajipour, N et al.	2020
Hajipour, N et al.	2020
Hajipour, N et al.	2020
Hajipour, N et al.	2020
Hajipour, N et al.	2020
Hajipour, N et al.	2020
Hajipour, N et al.	2020
Hajipour, N et al.	2020
Hajipour, N et al.	2020
Hajipour, N et al.	2020
Hajipour, N et al.	2020
Hajipour, N et al.	2020
Hajipour, N et al.	2020
Hajipour, N et al.	2020
Hajipour, N et al.	2020
Hajipour, N et al.	2020
Hajipour, N et al.	2020
Hajipour, N et al.	2020
Hajipour, N et al.	2020
Hajipour, N et al.	2020
Hajipour, N et al.	2020
Hajipour, N et al.	2020
Hajipour, N et al.	2020
Hajipour, N et al.	2020
Hajipour, N et al.	2020
Hajipour, N et al.	2020
Hajipour, N et al.	2020
Hajipour, N et al.	2020
Hajipour, N et al.	2020
Hajipour, N et al.	2020
Hajipour, N et al.	2020
Hajipour, N et al.	2020
Hajipour, N et al.	2020
Hajipour, N et al.	2020
Hajipour, N et al.	2020
Hajipour, N et al.	2020
Hajipour, N et al.	2020
Hajipour, N et al.	2020
Hajipour, N et al.	2020
Hajipour, N et al.	2020
Hajipour, N et al.	2020
Hajipour, N et al.	2020
Hajipour, N et al.	2020
Hajipour, N et al.	2020
Hajipour, N et al.	2020
Hajipour, N et al.	2020
Hajipour, N et al.	2020
Hajipour, N et al.	2020
Hajipour, N et al.	2020
Hajipour, N et al.	2020

Hajipour, N et al.	2020
Hajipour, N et al.	2020
Hajipour, N et al.	2020
Hajipour, N et al.	2020
Hajipour, N et al.	2020
Hajipour, N et al.	2020
Hajipour, N et al.	2020
Hajipour, N et al.	2020
Hajipour, N et al.	2020
Hajipour, N et al.	2020
Hajipour, N et al.	2020
Hajipour, N et al.	2020
Hajipour, N et al.	2020
Hajipour, N et al.	2020
Hajipour, N et al.	2020
Hajipour, N et al.	2020
Hajipour, N et al.	2020
Hajipour, N et al.	2020
Hajipour, N et al.	2020
Hajipour, N et al.	2020
Hajipour, N et al.	2020
Hajipour, N et al.	2020
Hajipour, N et al.	2020
Hajipour, N et al.	2020
Hajipour, N et al.	2020
Hajipour, N et al.	2020
Hajipour, N et al.	2020
Hajipour, N et al.	2020
Hajipour, N et al.	2020
Hajipour, N et al.	2020
Hajipour, N et al.	2020
Hajipour, N et al.	2020
Hajipour, N et al.	2020
Hajipour, N et al.	2020
Hajipour, N et al.	2020
Hajipour, N et al.	2020
Hajipour, N et al.	2020
Hajipour, N et al.	2020
Hajipour, N et al.	2020
Hajipour, N et al.	2020
Hajipour, N et al.	2020
Hajipour, N et al.	2020
Hajipour, N et al.	2020
Hajipour, N et al.	2020
Hajipour, N et al.	2020
Hajipour, N et al.	2020
Hajipour, N et al.	2020
Hajipour, N et al.	2020
Hajipour, N et al.	2020
Hajipour, N et al.	2020
Hajipour, N et al.	2020
Hajipour, N et al.	2020
Hajipour, N et al.	2020

Hajipour, N et al.	2020
Hajipour, N et al.	2020
Hajipour, N et al.	2020
Hajipour, N et al.	2020
Hajipour, N et al.	2020
Hajipour, N et al.	2020
Hajipour, N et al.	2020
Hajipour, N et al.	2020
Hajipour, N et al.	2020
Hajipour, N et al.	2020
Hajipour, N et al.	2020
Hajipour, N et al.	2020
Hajipour, N et al.	2020
Hajipour, N et al.	2020
Hajipour, N et al.	2020
Hajipour, N et al.	2020
Hajipour, N et al.	2020
Hajipour, N et al.	2020
Hajipour, N et al.	2020
Hajipour, N et al.	2020
Hajipour, N et al.	2020
Hajipour, N et al.	2020
Hajipour, N et al.	2020
Hajipour, N et al.	2020
Hajipour, N et al.	2020
Hajipour, N et al.	2020
Hajipour, N et al.	2020
Hajipour, N et al.	2020
Hajipour, N et al.	2020
Hajipour, N et al.	2020
Hajipour, N et al.	2020
Hajipour, N et al.	2020
Hajipour, N et al.	2020
Hajipour, N et al.	2020
Hajipour, N et al.	2020
Hajipour, N et al.	2020
Hajipour, N et al.	2020
Hajipour, N et al.	2020
Hajipour, N et al.	2020
Hajipour, N et al.	2020
Hajipour, N et al.	2020
Hajipour, N et al.	2020
Hajipour, N et al.	2020
Hajipour, N et al.	2020
Hajipour, N et al.	2020
Hajipour, N et al.	2020
Hajipour, N et al.	2020
Hajipour, N et al.	2020
Hajipour, N et al.	2020
Hajipour, N et al.	2020
Hajipour, N et al.	2020

Hajipour, N et al.	2020
Hajipour, N et al.	2020
Hajipour, N et al.	2020
Hajipour, N et al.	2020
Hajipour, N et al.	2020
Hajipour, N et al.	2020
Hajipour, N et al.	2020
Hajipour, N et al.	2020
Hajipour, N et al.	2020
Hajipour, N et al.	2020
Hajipour, N et al.	2020
Hajipour, N et al.	2020
Hajipour, N et al.	2020
Hajipour, N et al.	2020
Hajipour, N et al.	2020
Hajipour, N et al.	2020
Hajipour, N et al.	2020
Hajipour, N et al.	2020
Hajipour, N et al.	2020
Hajipour, N et al.	2020
Hajipour, N et al.	2020
Hajipour, N et al.	2020
Hajipour, N et al.	2020
Hajipour, N et al.	2020
Hajipour, N et al.	2020
Hajipour, N et al.	2020
Hajipour, N et al.	2020
Hajipour, N et al.	2020
Hajipour, N et al.	2020
Hajipour, N et al.	2020
Hajipour, N et al.	2020
Hajipour, N et al.	2020
Hajipour, N et al.	2020
Hajipour, N et al.	2020
Hajipour, N et al.	2020
Hajipour, N et al.	2020
Hajipour, N et al.	2020
Hajipour, N et al.	2020
Hajipour, N et al.	2020
Hajipour, N et al.	2020
Hajipour, N et al.	2020
Hajipour, N et al.	2020
Hajipour, N et al.	2020
Hajipour, N et al.	2020
Hajipour, N et al.	2020
Hajipour, N et al.	2020
Hajipour, N et al.	2020
Hajipour, N et al.	2020
Hajipour, N et al.	2020
Hajipour, N et al.	2020
Hajipour, N et al.	2020

Hajipour, N et al.	2020
Hassan, A et al.	2012
Hassan, A et al.	2012
Hassan, A et al.	2012
Hassan, A et al.	2012
Hassan, A et al.	2012
Hassan, A et al.	2012
Hassan, A et al.	2012
Hassan, A et al.	2012
Hassan, A et al.	2012
Hassan, A et al.	2012
Hassan, A et al.	2012
Hassan, A et al.	2012
Hassan, A et al.	2012
Hassan, A et al.	2012
Hassan, A et al.	2012
Hassan, A et al.	2012
Hassan, A et al.	2012
Hassan, A et al.	2012
Hassan, A et al.	2012
Hassan, A et al.	2012
Hassan, A et al.	2012
Hassan, A et al.	2012
Hassan, A et al.	2012
Hassan, A et al.	2012
Hassan, A et al.	2012
Hassan, A et al.	2012
Hassan, A et al.	2012
Hassan, A et al.	2012
Hassan, A et al.	2012
Hassan, A et al.	2012
I. Amorós et al.	2010
I. Amorós et al.	2010
I. Amorós et al.	2010
I. Amorós et al.	2010
I. Amorós et al.	2010
I. Amorós et al.	2010
J. Damen et al	2007
J. Damen et al	2007
J. Damen et al	2007
J. Damen et al	2007
J. Damen et al	2007
J. Damen et al	2007
J. Damen et al	2007
Jedrzejewski, S et al.	2007
Jedrzejewski, S et al.	2007
Jedrzejewski, S et al.	2007
K. Federer et al.	2016
K. Federer et al.	2016
K. Federer et al.	2016

K. Federer et al.	2016
K. Federer et al.	2016
K. Federer et al.	2016
K. Federer et al.	2016
K. Federer et al.	2016
K. Federer et al.	2016
K. Federer et al.	2016
K. Federer et al.	2016
K. Federer et al.	2016
Khan W. et al.	2021
Khan W. et al.	2021
Khan W. et al.	2021
Khan W. et al.	2021
Khan W. et al.	2021
Khan W. et al.	2021
Khan W. et al.	2021
Khan W. et al.	2021
Khan W. et al.	2021
Kim, K et al.	2014
Kim, K et al.	2014
Kim, K et al.	2014
Kim, K et al.	2014
Kim, K et al.	2014
Kim, K et al.	2014
Kim, K et al.	2014
Kim, K et al.	2014
Kłapeć, T	2009
Kłapeć, T	2009
Kłapeć, T	2009
Kłapeć, T	2009
Kłapeć, T	2009
Kłapeć, T	2009
Kłapeć, T	2009
Kłapeć, T	2009
Kłapeć, T	2009
Kłapeć, T	2009
Kłapeć, T	2009
Kłapeć, T	2009
Kłapeć, T	2009
Kłapeć, T	2009
Kłapeć, T	2009
Kłapeć, T	2009
Kłapeć, T	2009
Kłapeć, T	2009
Kłapeć, T	2009
Kłapeć, T	2009
Kłapeć, T	2009
Kłapeć, T	2009
Kłapeć, T	2009
Kłapeć, T	2009
Kłapeć, T	2009

Kłapeć, T	2009
Kłapeć, T	2009
Kłapeć, T	2009
Kłapeć, T	2009
Kłapeć, T	2009
Kłapeć, T	2009
Kłapeć, T	2009
Kłapeć, T	2009
Kłapeć, T	2009
Kłapeć, T	2009
Kłapeć, T	2009
Kłapeć, T	2009
Kłapeć, T	2009
Kłapeć, T	2009
Kłapeć, T	2009
Kłapeć, T	2009
Kłapeć, T	2009
Kłapeć, T	2009
Kłapeć, T	2009
Kłapeć, T	2009
Kłapeć, T	2009
Kłapeć, T	2009
Kłapeć, T	2009
Kłapeć, T	2009
Kłapeć, T	2009
Kłapeć, T	2009
Kłapeć, T	2009
Kłapeć, T	2009
Kłapeć, T	2009
Kłapeć, T	2009
Kłapeć, T	2009
Kłapeć, T	2009
Kłapeć, T	2009
Kłapeć, T	2009
Kłapeć, T	2009
Kłapeć, T	2009
Kłapeć, T	2009
Kłapeć, T	2009
Kłapeć, T	2009
Kłapeć, T	2009
Kłapeć, T	2009
Kłapeć, T	2009
Kłapeć, T	2009
Kłapeć, T	2009
Kłapeć, T	2009
Kłapeć, T	2009
Kłapeć, T	2009
Kłapeć, T	2009
Kłapeć, T	2009
Kłapeć, T	2009
Kłapeć, T	2009

Kłapeć, T	2009
Kłapeć, T	2009
Kłapeć, T	2009
Kłapeć, T	2009
Kłapeć, T	2009
Kłapeć, T	2009
Kłapeć, T	2009
Kłapeć, T	2009
Kłapeć, T	2009
Kłapeć, T	2009
Kłapeć, T	2009
Kłapeć, T	2009
Kłapeć, T	2009
Kłapeć, T	2009
Kłapeć, T	2009
Kłapeć, T	2009
Kłapeć, T	2009
Kłapeć, T	2009
Kłapeć, T	2009
Kłapeć, T	2009
Kłapeć, T	2009
Kłapeć, T	2009
Kłapeć, T	2009
Kłapeć, T	2009
Kłapeć, T	2009
Kłapeć, T	2009
Kłapeć, T	2009
Kłapeć, T	2009
Kłapeć, T	2009
Kłapeć, T	2009
Kłapeć, T	2009
Kłapeć, T	2009
Kłapeć, T	2009
Kłapeć, T	2009
Kłapeć, T	2009
Kłapeć, T	2009
Kłapeć, T	2009
Kłapeć, T	2009
Kłapeć, T	2009
Kłapeć, T	2009
Kłapeć, T	2009
Kłapeć, T	2009
Kłapeć, T	2009
Kłapeć, T	2009
Kozan, E et al.	2005
Kozan, E et al.	2005
Kozan, E et al.	2005
Kozan, E et al.	2005
Kozan, E et al.	2005
Kozan, E et al.	2005

Kozan, E et al.	2005
Kozan, E et al.	2005
Kozan, E et al.	2005
Kozan, E et al.	2005
Kozan, E et al.	2005
Kozan, E et al.	2005
Kozan, E et al.	2005
Kozan, E et al.	2005
Kozan, E et al.	2005
Kozan, E et al.	2005
Kozan, E et al.	2005
Kozan, E et al.	2005
Kozan, E et al.	2005
Kozan, E et al.	2005
Kozan, E et al.	2005
Kozan, E et al.	2005
Kozan, E et al.	2005
Kozan, E et al.	2005
Kozan, E et al.	2005
Kozan, E et al.	2005
Kozan, E et al.	2005
Kudah, Catherine et al.	2018
Kudah, Catherine et al.	2018
Kudah, Catherine et al.	2018
Kudah, Catherine et al.	2018
Kudah, Catherine et al.	2018
Kudah, Catherine et al.	2018
Kudah, Catherine et al.	2018
Kudah, Catherine et al.	2018
Kudah, Catherine et al.	2018
Kudah, Catherine et al.	2018
Kudah, Catherine et al.	2018
Kudah, Catherine et al.	2018
Kudah, Catherine et al.	2018
Kudah, Catherine et al.	2018
Kudah, Catherine et al.	2018
Kudah, Catherine et al.	2018
Kudah, Catherine et al.	2018
Kudah, Catherine et al.	2018
Kudah, Catherine et al.	2018
Kudah, Catherine et al.	2018
Lalonde, L F et al.	2016
Lalonde, L F et al.	2016
Lalonde, L F et al.	2016
Lalonde, L F et al.	2016
Lalonde, L F et al.	2016
Lalonde, L F et al.	2016
Lalonde, L F et al.	2016
Lalonde, L F et al.	2016
Lalonde, L F et al.	2016

Lalonde, L F et al.	2016
Lalonde, L F et al.	2016
Lalonde, L F et al.	2016
Lalonde, L F et al.	2016
Lalonde, L F et al.	2016
Lalonde, L F et al.	2016
Lalonde, L F et al.	2016
Lalonde, L F et al.	2016
Lalonde, L F et al.	2016
Lalonde, L F et al.	2016
Lalonde, L F et al.	2016
Lalonde, L F et al.	2016
Lalonde, L F et al.	2016
Lalonde, L F et al.	2016
Lalonde, L F et al.	2016
Lalonde, L F et al.	2016
Lalonde, L F et al.	2016
Lalonde, L F et al.	2016
Lass et al.	2011
Lass et al.	2011
Lass et al.	2011
Lass et al.	2011
Lass et al.	2011
Lass et al.	2011
Lass et al.	2011
Lass et al.	2011
Lass, A et al.	2015
Lass, A et al.	2015
Lass, A et al.	2015
Li, J et al.	2019
Li, J et al.	2019
Li, J et al.	2019
Li, J et al.	2019
Li, J et al.	2019
Li, J et al.	2019
Li, J et al.	2019
Li, J et al.	2019
Li, J et al.	2019
Li, J et al.	2019
Li, J et al.	2019
Li, J et al.	2019
Li, J et al.	2019
Li, J et al.	2019
Li, J et al.	2019
Li, J et al.	2019
Li, J et al.	2019
Li, J et al.	2019
Li, J et al.	2019
Li, J et al.	2019
Li, J et al.	2019

Li, J et al.	2019
Li, J et al.	2019
Li, J et al.	2019
Li, J et al.	2019
Li, J et al.	2019
Li, J et al.	2019
Li, J et al.	2019
Li, J et al.	2019
Li, J et al.	2019
Li, J et al.	2019
Li, J et al.	2019
Li, J et al.	2019
Li, J et al.	2019
Li, J et al.	2019
Li, J et al.	2019
Li, J et al.	2019
Li, J et al.	2019
Li, J et al.	2019
Li, J et al.	2019
Li, J et al.	2019
Li, J et al.	2019
Li, J et al.	2019
Li, J et al.	2019
Li, J et al.	2019
Li, J et al.	2019
Li, J et al.	2019
Li, J et al.	2019
Li, J et al.	2019
Li, J et al.	2019
Li, J et al.	2019
Li, J et al.	2019
Li, J et al.	2019
Li, J et al.	2019
Li, J et al.	2019
Li, J et al.	2019
Li, J et al.	2019
Li, J et al.	2019
Li, J et al.	2019
Li, J et al.	2019
Li, J et al.	2019
Li, J et al.	2019
Li, J et al.	2019
LUZ et al.	2017
LUZ et al.	2017
LUZ et al.	2017
LUZ et al.	2017
LUZ et al.	2017
LUZ et al.	2017
LUZ et al.	2017
LUZ et al.	2017
LUZ et al.	2017

LUZ et al.	2017
LUZ et al.	2017
LUZ et al.	2017
LUZ et al.	2017
LUZ et al.	2017
LUZ et al.	2017
LUZ et al.	2017
LUZ et al.	2017
LUZ et al.	2017
LUZ et al.	2017
LUZ et al.	2017
LUZ et al.	2017
LUZ et al.	2017
LUZ et al.	2017
LUZ et al.	2017
LUZ et al.	2017
LUZ et al.	2017
LUZ et al.	2017
LUZ et al.	2017
LUZ et al.	2017
LUZ et al.	2017
LUZ et al.	2017
LUZ et al.	2017
LUZ et al.	2017
LUZ et al.	2017
LUZ et al.	2017
LUZ et al.	2017
LUZ et al.	2017
LUZ et al.	2017
LUZ et al.	2017
LUZ et al.	2017
LUZ et al.	2017
LUZ et al.	2017
LUZ et al.	2017
LUZ et al.	2017
LUZ et al.	2017
LUZ et al.	2017
LUZ et al.	2017
LUZ et al.	2017
LUZ et al.	2017
LUZ et al.	2017
LUZ et al.	2017
LUZ et al.	2017
LUZ et al.	2017
LUZ et al.	2017
LUZ et al.	2017
LUZ et al.	2017
LUZ et al.	2017
LUZ et al.	2017
LUZ et al.	2017
LUZ et al.	2017

LUZ et al.	2017
LUZ et al.	2017
LUZ et al.	2017
LUZ et al.	2017
LUZ et al.	2017
LUZ et al.	2017
LUZ et al.	2017
LUZ et al.	2017
LUZ et al.	2017
LUZ et al.	2017
LUZ et al.	2017
LUZ et al.	2017
LUZ et al.	2017
LUZ et al.	2017
LUZ et al.	2017
LUZ et al.	2017
LUZ et al.	2017
LUZ et al.	2017
LUZ et al.	2017
LUZ et al.	2017
LUZ et al.	2017
LUZ et al.	2017
LUZ et al.	2017
LUZ et al.	2017
LUZ et al.	2017
LUZ et al.	2017
LUZ et al.	2017
LUZ et al.	2017
LUZ et al.	2017
LUZ et al.	2017
M. Calvo et al	2004
M. Calvo et al	2004
M. Calvo et al	2004
M. Calvo et al	2004
M. Calvo et al	2004
M. Calvo et al	2004
M. Calvo et al	2004
M. Calvo et al	2004
M. Calvo et al	2004
M. Calvo et al	2004
M. Isazadeh et al.	2020
M. Isazadeh et al.	2020
M. Isazadeh et al.	2020
M. Isazadeh et al.	2020
M. Isazadeh et al.	2020
M. Isazadeh et al.	2020
M. Isazadeh et al.	2020
M. Isazadeh et al.	2020
M. Isazadeh et al.	2020
M. Isazadeh et al.	2020

M. Isazadeh et al.	2020
M. Isazadeh et al.	2020
M. Isazadeh et al.	2020
M. Isazadeh et al.	2020
M. Isazadeh et al.	2020
M. Isazadeh et al.	2020
M. Isazadeh et al.	2020
M. Isazadeh et al.	2020
M. Isazadeh et al.	2020
M. Isazadeh et al.	2020
M. Isazadeh et al.	2020
M. Isazadeh et al.	2020
M. Isazadeh et al.	2020
M. Isazadeh et al.	2020
M. Isazadeh et al.	2020
M. Isazadeh et al.	2020
M. Isazadeh et al.	2020
M. Isazadeh et al.	2020
M. Isazadeh et al.	2020
M. Isazadeh et al.	2020
M. Isazadeh et al.	2020
M. Isazadeh et al.	2020
M. Isazadeh et al.	2020
M. Isazadeh et al.	2020
M. Isazadeh et al.	2020
M. Isazadeh et al.	2020
M. Isazadeh et al.	2020
M. Isazadeh et al.	2020
M. Isazadeh et al.	2020
M. Isazadeh et al.	2020
M. Isazadeh et al.	2020
M. Isazadeh et al.	2020
M. Isazadeh et al.	2020
M. Isazadeh et al.	2020
M. Isazadeh et al.	2020
M. Isazadeh et al.	2020
M. Isazadeh et al.	2020
M. Isazadeh et al.	2020
M. Isazadeh et al.	2020
M. Isazadeh et al.	2020
M. Isazadeh et al.	2020
M. Isazadeh et al.	2020
M. Isazadeh et al.	2020
M. Isazadeh et al.	2020
M. Isazadeh et al.	2020
M. Isazadeh et al.	2020
M. Isazadeh et al.	2020
M. Isazadeh et al.	2020
M. Isazadeh et al.	2020
M. Isazadeh et al.	2020

M. Isazadeh et al.	2020
M. Isazadeh et al.	2020
M. Isazadeh et al.	2020
M. Isazadeh et al.	2020
M. Isazadeh et al.	2020
M. Isazadeh et al.	2020
M. Isazadeh et al.	2020
M. Isazadeh et al.	2020
M. Isazadeh et al.	2020
M. Isazadeh et al.	2020
M. Isazadeh et al.	2020
M. Isazadeh et al.	2020
M. Marilyn et al	2009
M. Marilyn et al	2009
M. Marilyn et al	2009
M. Marilyn et al	2009
M. Marilyn et al	2009
M. Marilyn et al	2009
M.Nisha et al	2018
M.Nisha et al	2018
M.Nisha et al	2018
M.Nisha et al	2018
Maikai, B V et al.	2013
Maikai, B V et al.	2013
Maikai, B V et al.	2013
Maikai, B V et al.	2013
Maikai, B V et al.	2013
Maikai, B V et al.	2013
Maikai, B V et al.	2013
Maikai, B V et al.	2013
Marchioro et al	2016
Marchioro et al	2016
Marchioro et al	2016
Marchioro et al	2016
Mohamed, Mona Ali et al.	2018
Mohamed, Mona Ali et al.	2018
Mohamed, Mona Ali et al.	2018
Mohamed, Mona Ali et al.	2018
Mohamed, Mona Ali et al.	2018
Mohamed, Mona Ali et al.	2018
Mohamed, Mona Ali et al.	2018
Mohamed, Mona Ali et al.	2018
Mohamed, Mona Ali et al.	2018
Mohamed, Mona Ali et al.	2018
Mohamed, Mona Ali et al.	2018
Mohamed, Mona Ali et al.	2018
Mohamed, Mona Ali et al.	2018
Mohamed, Mona Ali et al.	2018
Mohamed, Mona Ali et al.	2018
Mohamed, Mona Ali et al.	2018

Mohamed, Mona Ali et al.	2018
Mohamed, Mona Ali et al.	2018
Mohamed, Mona Ali et al.	2018
Mohamed, Mona Ali et al.	2018
Mohamed, Mona Ali et al.	2018
Mohamed, Mona Ali et al.	2018
Mohamed, Mona Ali et al.	2018
Mohamed, Mona Ali et al.	2018
Mohamed, Mona Ali et al.	2018
Mohamed, Mona Ali et al.	2018
Mohamed, Mona Ali et al.	2018
Mohamed, Mona Ali et al.	2018
Mohamed, Mona Ali et al.	2018
Mohamed, Mona Ali et al.	2018
Mohamed, Mona Ali et al.	2018
Mohamed, Mona Ali et al.	2018
Mohamed, Mona Ali et al.	2018
Mohamed, Mona Ali et al.	2018
Mohamed, Mona Ali et al.	2018
Mohamed, Mona Ali et al.	2018
Mohamed, Mona Ali et al.	2018
Mohamed, Mona Ali et al.	2018
Mohamed, Mona Ali et al.	2018
Mohamed, Mona Ali et al.	2018
Mohamed, Mona Ali et al.	2018
Mohamed, Mona Ali et al.	2018
Mohamed, Mona Ali et al.	2018
Mohamed, Mona Ali et al.	2018
Mohamed, Mona Ali et al.	2018
Mohamed, Mona Ali et al.	2018
Mohamed, Mona Ali et al.	2018
Mohamed, Mona Ali et al.	2018
Mohamed, Mona Ali et al.	2018
Mohamed, Mona Ali et al.	2018
Mohamed, Mona Ali et al.	2018
Mohamed, Mona Ali et al.	2018
Mohamed, Mona Ali et al.	2018
Mohamed, Mona Ali et al.	2018
Mohamed, Mona Ali et al.	2018
Mohamed, Mona Ali et al.	2018
Mohamed, Mona Ali et al.	2018
Mohamed, Mona Ali et al.	2018
Mohamed, Mona Ali et al.	2018
Mohamed, Mona Ali et al.	2018
Mohamed, Mona Ali et al.	2018
Mohamed, Mona Ali et al.	2018
Mohamed, Mona Ali et al.	2018
Mohamed, Mona Ali et al.	2018
Mohamed, Mona Ali et al.	2018
Mohamed, Mona Ali et al.	2018

Muniswamappa et al.	2012
N. Gupta et al	2009
N. Gupta et al	2009
N. Gupta et al	2009
N. Gupta et al	2009
N. Gupta et al	2009
N. Gupta et al	2009
N. Gupta et al	2009
N. Gupta et al	2009
N. Gupta et al	2009
N. Gupta et al	2009
N. Gupta et al	2009
N. Gupta et al	2009
N. Gupta et al	2009
N. Gupta et al	2009
N. Gupta et al	2009
N. Gupta et al	2009
N. Gupta et al	2009
N. Gupta et al	2009
Neres et al	2011
Neres et al	2011
Neres et al	2011
Neres et al	2011
Neres et al	2011
Neres et al	2011
Neres et al	2011
Neres et al	2011
Neres et al	2011
Neres et al	2011
Neres et al	2011
Neres et al	2011
Neres et al	2011
Neres et al	2011
Neres et al	2011
Neres et al	2011
Neres et al	2011
Neres et al	2011
Neres et al	2011
Neres et al	2011
Neres et al	2011
Neres et al	2011
Neres et al	2011
Neres et al	2011
Neres et al	2011
Neres et al	2011
Neres et al	2011
Neres et al	2011
Neres et al	2011
Neres et al	2011
Neres et al	2011
Neres et al	2011

Neres et al	2011
Neres et al	2011
Neres et al	2011
Neres et al	2011
Neres et al	2011
Neres et al	2011
Neres et al	2011
Neres et al	2011
Neres et al	2011
Neres et al	2011
Neres et al	2011
Neres et al	2011
Neres et al	2011
Neres et al	2011
Neres et al	2011
Neres et al	2011
Neres et al	2011
Neres et al	2011
Neres et al	2011
Neres et al	2011
O. Erdogrul et al	2004
O. Erdogrul et al	2004
O. Erdogrul et al	2004
O. Erdogrul et al	2004
O. Erdogrul et al	2004
O. Erdogrul et al	2004
O. Erdogrul et al	2004
O. Erdogrul et al	2004
O. Erdogrul et al	2004
O. Erdogrul et al	2004
O. Erdogrul et al	2004
O. Erdogrul et al	2004
O. Erdogrul et al	2004
O. Erdogrul et al	2004
O. Erdogrul et al	2004
O. Erdogrul et al	2004
O. Erdogrul et al	2004
O. Erdogrul et al	2004
O. Erdogrul et al	2004
O. Erdogrul et al	2004
Obebe, Oluwasola Olaiya et al.	2020
Obebe, Oluwasola Olaiya et al.	2020
Obebe, Oluwasola Olaiya et al.	2020
Obebe, Oluwasola Olaiya et al.	2020
Obebe, Oluwasola Olaiya et al.	2020
Obebe, Oluwasola Olaiya et al.	2020
Obebe, Oluwasola Olaiya et al.	2020
Olyaei, A; Hajivandi, L	2013
Olyaei, A; Hajivandi, L	2013
Olyaei, A; Hajivandi, L	2013

Olyaei, A; Hajivandi, L	2013
Olyaei, A; Hajivandi, L	2013
Olyaei, A; Hajivandi, L	2013
Olyaei, A; Hajivandi, L	2013
Olyaei, A; Hajivandi, L	2013
Olyaei, A; Hajivandi, L	2013
Olyaei, A; Hajivandi, L	2013
Olyaei, A; Hajivandi, L	2013
Olyaei, A; Hajivandi, L	2013
Olyaei, A; Hajivandi, L	2013
Ouhood Mozahim Shakir et al.	2019
Ouhood Mozahim Shakir et al.	2019
Ouhood Mozahim Shakir et al.	2019
Ouhood Mozahim Shakir et al.	2019
Ouhood Mozahim Shakir et al.	2019
Ouhood Mozahim Shakir et al.	2019
Ouhood Mozahim Shakir et al.	2019
Ouhood Mozahim Shakir et al.	2019
Ouhood Mozahim Shakir et al.	2019
Ouhood Mozahim Shakir et al.	2019
Ouhood Mozahim Shakir et al.	2019
Ouhood Mozahim Shakir et al.	2019
Ouhood Mozahim Shakir et al.	2019
Ouhood Mozahim Shakir et al.	2019
Ouhood Mozahim Shakir et al.	2019
Ouhood Mozahim Shakir et al.	2019
Ouhood Mozahim Shakir et al.	2019
Ouhood Mozahim Shakir et al.	2019
Ouhood Mozahim Shakir et al.	2019
Ouhood Mozahim Shakir et al.	2019
Ouhood Mozahim Shakir et al.	2019
Paula et al.	2003
Paula et al.	2003
Peña, Y P et al.	2013
Peña, Y P et al.	2013
Peña, Y P et al.	2013
Peña, Y P et al.	2013
Peña, Y P et al.	2013
Peña, Y P et al.	2013
Peña, Y P et al.	2013
Peña, Y P et al.	2013
Peña, Y P et al.	2013
Peña, Y P et al.	2013
Peña, Y P et al.	2013
Peña, Y P et al.	2013
Peña, Y P et al.	2013
Peña, Y P et al.	2013
Peña, Y P et al.	2013
Peña, Y P et al.	2013
Peña, Y P et al.	2013

Peña, Y P et al.	2013
Peña, Y P et al.	2013
Peña, Y P et al.	2013
Peña, Y P et al.	2013
Peña, Y P et al.	2013
Peña, Y P et al.	2013
Peña, Y P et al.	2013
Peña, Y P et al.	2013
Peña, Y P et al.	2013
Peña, Y P et al.	2013
Peña, Y P et al.	2013
Peña, Y P et al.	2013
Peña, Y P et al.	2013
Peña, Y P et al.	2013
Peña, Y P et al.	2013
Peña, Y P et al.	2013
Peña, Y P et al.	2013
Peña, Y P et al.	2013
Peña, Y P et al.	2013
Peña, Y P et al.	2013
Peña, Y P et al.	2013
Perim, L V et al.	2020
Perim, L V et al.	2020
Perim, L V et al.	2020
Perim, L V et al.	2020
Perim, L V et al.	2020
Pinto-FerreIran et al.	2019
Pinto-FerreIran et al.	2019
Pinto-FerreIran et al.	2019
Pinto-FerreIran et al.	2019
Pinto-FerreIran et al.	2019
Pinto-FerreIran et al.	2019
Pinto-FerreIran et al.	2019
Pinto-FerreIran et al.	2019
Pinto-FerreIran et al.	2019
Pinto-FerreIran et al.	2019
Pinto-FerreIran et al.	2019
Pinto-FerreIran et al.	2020
Pinto-FerreIran et al.	2020
Pinto-FerreIran et al.	2020
Pinto-FerreIran et al.	2019
Punsawad, Chuchard et al.	2019
Punsawad, Chuchard et al.	2019
Punsawad, Chuchard et al.	2019
Punsawad, Chuchard et al.	2019
Punsawad, Chuchard et al.	2019
Punsawad, Chuchard et al.	2019
Punsawad, Chuchard et al.	2019
Punsawad, Chuchard et al.	2019
Punsawad, Chuchard et al.	2019

Punsawad, Chuchard et al.	2019
Punsawad, Chuchard et al.	2019
Punsawad, Chuchard et al.	2019
Punsawad, Chuchard et al.	2019
Punsawad, Chuchard et al.	2019
Punsawad, Chuchard et al.	2019
Punsawad, Chuchard et al.	2019
Punsawad, Chuchard et al.	2019
Punsawad, Chuchard et al.	2019
Punsawad, Chuchard et al.	2019
Punsawad, Chuchard et al.	2019
Punsawad, Chuchard et al.	2019
Punsawad, Chuchard et al.	2019
Punsawad, Chuchard et al.	2019
Punsawad, Chuchard et al.	2019
Punsawad, Chuchard et al.	2019
Punsawad, Chuchard et al.	2019
Punsawad, Chuchard et al.	2019
Punsawad, Chuchard et al.	2019
Punsawad, Chuchard et al.	2019
Punsawad, Chuchard et al.	2019
Punsawad, Chuchard et al.	2019
Punsawad, Chuchard et al.	2019
Punsawad, Chuchard et al.	2019
Punsawad, Chuchard et al.	2019
Punsawad, Chuchard et al.	2019
Punsawad, Chuchard et al.	2019
Punsawad, Chuchard et al.	2019
Punsawad, Chuchard et al.	2019
Punsawad, Chuchard et al.	2019
Punsawad, Chuchard et al.	2019
Punsawad, Chuchard et al.	2019
Punsawad, Chuchard et al.	2019
Punsawad, Chuchard et al.	2019
Punsawad, Chuchard et al.	2019
Punsawad, Chuchard et al.	2019
Punsawad, Chuchard et al.	2019
Punsawad, Chuchard et al.	2019
Punsawad, Chuchard et al.	2019
Punsawad, Chuchard et al.	2019
Punsawad, Chuchard et al.	2019
R. Ada et al	2018
R. Ada et al	2018
R. Ada et al	2018
R. Ada et al	2018
R. Ada et al	2018
R. Ada et al	2018
R. Ada et al	2018
R. Ada et al	2018
R. Ada et al	2018
R. Ada et al	2018

R. Ada et al	2018
R. Ada et al	2018
R. Ada et al	2018
R. Ada et al	2018
R. Ada et al	2018
R. Ada et al	2018
R. Ada et al	2018
R. Ada et al	2018
R. Ada et al	2018
R. Ada et al	2018
R. Mohammed et al	2019
R. Mohammed et al	2019
R. Mohammed et al	2019
R. Mohammed et al	2019
R. Mohammed et al	2019
R. Mohammed et al	2019
R. Mohammed et al	2019
R. Mohammed et al	2019
Rafael et al	2017
Rafael et al	2017
Rafael et al	2017
Rafael et al	2017
Rafael et al	2017
Rafael et al	2017
Rafael et al	2017
Rafael et al	2017
Robertson, L J et al.	2001
Robertson, L J et al.	2001
Robertson, L J et al.	2001
Robertson, L J et al.	2001
Robertson, L J et al.	2001
Robertson, L J et al.	2001
Robertson, L J et al.	2001
Robertson, L J et al.	2001
Robertson, L J et al.	2001
Robertson, L J et al.	2001
Robertson, L J et al.	2001
Robertson, L J et al.	2001
Robertson, L J et al.	2001
Robertson, L J et al.	2001
Robertson, L J et al.	2001
Robertson, L J et al.	2001
Robertson, L J et al.	2001
Robertson, L J et al.	2001
Robertson, L J et al.	2001
Robertson, L J et al.	2001
Robertson, L J et al.	2001
Robertson, L J et al.	2001
Robertson, L J et al.	2001
Robertson, L J et al.	2001
Robertson, L J et al.	2001
Robertson, L J et al.	2001
Robertson, L J et al.	2001
Robertson, L J et al.	2001
Robertson, L J et al.	2001
Robertson, L J et al.	2001

Robertson, L J et al.	2001
Robertson, L J et al.	2001
Robertson, L J et al.	2001
Robertson, L J et al.	2001
Robertson, L J et al.	2001
Rodrigues et al.	2020
Rodrigues et al.	2020
Rodrigues et al.	2020
Rodrigues et al.	2020
Rodrigues et al.	2020
Rodrigues et al.	2020
Rodrigues et al.	2020
Rodrigues et al.	2020
Rodrigues et al.	2020
Rodrigues et al.	2020
Rodrigues et al.	2020
Rodrigues et al.	2020
Rodrigues et al.	2020
Rodrigues et al.	2020
Rodrigues et al.	2020
Rodrigues et al.	2020
Rodrigues et al.	2020
Rodrigues et al.	2020
Rodrigues et al.	2020
Rzezutka, A. et al	2010
Rzezutka, A. et al	2010
Rzezutka, A. et al	2010
Rzezutka, A. et al	2010
Rzezutka, A. et al	2010
Rzezutka, A. et al	2010
S. Uga et al	2009
S. Uga et al	2009
S. Uga et al	2009
S. Uga et al	2009
S. Uga et al	2009
S. Uga et al	2009
S. Uga et al	2009
S. Uga et al	2009
S. Uga et al	2009
S. Uga et al	2009
S. Uga et al	2009
S. Uga et al	2009
S. Uga et al	2009
S. Uga et al	2009
S. Uga et al	2009
S. Uga et al	2009
S. Uga et al	2009
S. Uga et al	2009
S. Uga et al	2009
S. Uga et al	2009
S. Uga et al	2009
S. Uga et al	2009
S. Uga et al	2009
S. Uga et al	2009

S. Uga et al	2009
S. Uga et al	2009
S. Uga et al	2009
S. Uga et al	2009
S. Uga et al	2009
S. Uga et al	2009
S. Uga et al	2009
S. Uga et al	2009
S. Uga et al	2009
S. Uga et al	2009
S. Uga et al	2009
S. Uga et al	2009
S. Uga et al	2009
S. Uga et al	2009
S. Uga et al	2009
S. Uga et al	2009
S. Uga et al	2009
S. Uga et al	2009
S. Uga et al	2009
S. Uga et al	2009
S. Uga et al	2009
S. Uga et al	2009
S. Uga et al	2009
S. Uga et al	2009
S. Uga et al	2009
S. Uga et al	2009
S. Uga et al	2009
S. Uga et al	2009
S. Uga et al	2009
S. Uga et al	2009
S. Uga et al	2009
S. Uga et al	2009
S. Uga et al	2009
S. Uga et al	2009
S. Uga et al	2009
S. Uga et al	2009
S. Uga et al	2009
S. Uga et al	2009
S. Uga et al	2009
S. Uga et al	2009
S. Uga et al	2009
S. Uga et al	2009
S. Uga et al	2009
S. Uga et al	2009
S. Uga et al	2009
S. Uga et al	2009
S. Uga et al	2009
S. Uga et al	2009
S. Uga et al	2009
S. Uga et al	2009
S. Uga et al	2009

S. Yusuf et al	2016
S. Yusuf et al	2016
S. Yusuf et al	2016
S. Yusuf et al	2016
S. Yusuf et al	2016
S. Yusuf et al	2016
S. Yusuf et al	2016
S.Ranjbar-Bahadori et al	2013
S.Ranjbar-Bahadori et al	2013
S.Ranjbar-Bahadori et al	2013
S.Ranjbar-Bahadori et al	2013
S.Ranjbar-Bahadori et al	2013
S.Ranjbar-Bahadori et al	2013
Saki, J. et al.,	2013
Saki, J. et al.,	2013
Saki, J. et al.,	2013
Saki, J. et al.,	2013
Saki, J. et al.,	2013
Saki, J. et al.,	2013
Saki, J. et al.,	2013
Saki, J. et al.,	2013
Saki, J. et al.,	2013
Saki, J. et al.,	2013
Saki, J. et al.,	2013
Saki, J. et al.,	2013
Saki, J. et al.,	2013
Saki, J. et al.,	2013
Saki, J. et al.,	2013
Saki, J. et al.,	2013
Saki, J. et al.,	2013
Saki, J. et al.,	2013
Saki, J. et al.,	2013
Shafa-ul-Haq et al.	2014
Shafa-ul-Haq et al.	2014
Shafa-ul-Haq et al.	2014
Shafa-ul-Haq et al.	2014
Shafa-ul-Haq et al.	2014
Shafa-ul-Haq et al.	2014
Shafa-ul-Haq et al.	2014
Shafa-ul-Haq et al.	2014
Shafa-ul-Haq et al.	2014
Shafa-ul-Haq et al.	2014
Shafa-ul-Haq et al.	2014
Shafa-ul-Haq et al.	2014
Shafa-ul-Haq et al.	2014
Shafa-ul-Haq et al.	2014
Shafa-ul-Haq et al.	2014
Shafa-ul-Haq et al.	2014
Shafa-ul-Haq et al.	2014
Shafa-ul-Haq et al.	2014
Shafa-ul-Haq et al.	2014
Shafa-ul-Haq et al.	2014
Shafa-ul-Haq et al	2014

Shafa-ul-Haq et al.	2014
Shafa-ul-Haq et al.	2014
Shafa-ul-Haq et al.	2014
Shafa-ul-Haq et al.	2014
Shafa-ul-Haq et al.	2014
Shafa-ul-Haq et al.	2014
Shafa-ul-Haq et al.	2014
Shafa-ul-Haq et al.	2014
Shafa-ul-Haq et al.	2014
Shafa-ul-Haq et al.	2014
Shafa-ul-Haq et al.	2014
Shafa-ul-Haq et al.	2014
Shafa-ul-Haq et al.	2014
Shafa-ul-Haq et al.	2014
Shafa-ul-Haq et al.	2014
Shafa-ul-Haq et al.	2014
Shafa-ul-Haq et al.	2014
Shafa-ul-Haq et al.	2014
Shafa-ul-Haq et al.	2014
Shafa-ul-Haq et al.	2014
Shafa-ul-Haq et al.	2014
Shafa-ul-Haq et al.	2014
Shafa-ul-Haq et al.	2014
Shafa-ul-Haq et al.	2014
Shafa-ul-Haq et al.	2014
Shafa-ul-Haq et al.	2014
Shafa-ul-Haq et al.	2014
Shafa-ul-Haq et al.	2014
Shafa-ul-Haq et al.	2014
Shafa-ul-Haq et al.	2014
Shafa-ul-Haq et al.	2014
Shafa-ul-Haq et al.	2014
Shafa-ul-Haq et al.	2014
Shafa-ul-Haq et al.	2014
Shafa-ul-Haq et al.	2014
Shafa-ul-Haq et al.	2014
Shafa-ul-Haq et al.	2014
Shafa-ul-Haq et al.	2014
Shafa-ul-Haq et al.	2014
Shafa-ul-Haq et al.	2014
Shafa-ul-Haq et al.	2014
Shafa-ul-Haq et al.	2014
Shafa-ul-Haq et al.	2014
Shafa-ul-Haq et al.	2014
Shafa-ul-Haq et al.	2014
Shafa-ul-Haq et al.	2014
Shafa-ul-Haq et al.	2014
Shafa-ul-Haq et al.	2014
Shafa-ul-Haq et al.	2014
Shafa-ul-Haq et al.	2014
Shafa-ul-Haq et al.	2014

Shafa-ul-Haq et al.	2014
Shafa-ul-Haq et al.	2014
Shafa-ul-Haq et al.	2014
Shafa-ul-Haq et al.	2014
Shafa-ul-Haq et al.	2014
Shafa-ul-Haq et al.	2014
Shafa-ul-Haq et al.	2014
Shafa-ul-Haq et al.	2014
Shafa-ul-Haq et al.	2014
Shafa-ul-Haq et al.	2014
Shafa-ul-Haq et al.	2014
Shafa-ul-Haq et al.	2014
Shafa-ul-Haq et al.	2014
Shafa-ul-Haq et al.	2014
Shafa-ul-Haq et al.	2014
Shafa-ul-Haq et al.	2014
Shafa-ul-Haq et al.	2014
Shafa-ul-Haq et al.	2014
Shafa-ul-Haq et al.	2014
Shafa-ul-Haq et al.	2014
Shafa-ul-Haq et al.	2014
Shafa-ul-Haq et al.	2014
Shafa-ul-Haq et al.	2014
Shafa-ul-Haq et al.	2014
Shafa-ul-Haq et al.	2014
Shafa-ul-Haq et al.	2014
Shafa-ul-Haq et al.	2014
Shafa-ul-Haq et al.	2014
Shafa-ul-Haq et al.	2014
Shafa-ul-Haq et al.	2014
Shafa-ul-Haq et al.	2014
Shafa-ul-Haq et al.	2014
Shafa-ul-Haq et al.	2014
Shafa-ul-Haq et al.	2014
Shafa-ul-Haq et al.	2014
Shafa-ul-Haq et al.	2014
Shafa-ul-Haq et al.	2014
Shafa-ul-Haq et al.	2014
Shafa-ul-Haq et al.	2014
Shafa-ul-Haq et al.	2014
Shafa-ul-Haq et al.	2014
Shafa-ul-Haq et al.	2014
Shafa-ul-Haq et al.	2014
Shafa-ul-Haq et al.	2014
Shafa-ul-Haq et al.	2014
Shafa-ul-Haq et al.	2014
Shafa-ul-Haq et al.	2014
Shafa-ul-Haq et al.	2014
Shahnazi et al.	2010
Shahnazi et al.	2010
Shahnazi et al.	2010
Shahnazi et al.	2010

Shahnazi et al.	2010
Shahnazi et al.	2010
Shahnazi et al.	2010
Shahnazi et al.	2010
Shahnazi et al.	2010
Shahnazi et al.	2010
Shahnazi et al.	2010
Shahnazi et al.	2010
Shahnazi et al.	2010
Shahnazi et al.	2010
Shahnazi et al.	2010
Shahnazi et al.	2010
Shahnazi et al.	2010
Shahnazi et al.	2010
Shahnazi et al.	2010
Shahnazi et al.	2010
Shahnazi et al.	2010
Shahnazi et al.	2010
Shahnazi et al.	2010
Shahnazi et al.	2010
Shahnazi et al.	2010
Shahnazi et al.	2010
Shahnazi et al.	2010
Shahnazi et al.	2010
Shahnazi et al.	2010
Shahnazi et al.	2010
Shahnazi et al.	2010
Shahnazi et al.	2010
Shahnazi et al.	2010
Shahnazi et al.	2010
Shahnazi et al.	2010
Shahnazi et al.	2010
Shahnazi et al.	2010
Shahnazi et al.	2010
Shahnazi et al.	2010
Shahnazi et al.	2010
Shahnazi et al.	2010
Shahnazi et al.	2010
Shahnazi et al.	2010
Shahnazi et al.	2010
Shahnazi et al.	2010
Shahnazi et al.	2010
Shahnazi et al.	2010
Shahnazi et al.	2010
Shahnazi et al.	2010
Shahnazi et al.	2010
Shahnazi et al.	2010
Shahnazi et al.	2010
Shahnazi et al.	2010
Shahnazi et al.	2010
Shahnazi et al.	2010

Shahnazi et al.	2010
Shahnazi et al.	2010
Shahnazi et al.	2010
Shahnazi et al.	2010
Shahnazi et al.	2010
Shahnazi et al.	2010
Shahnazi et al.	2010
Shahnazi et al.	2010
Shahnazi et al.	2010
Shahnazi et al.	2010
Shahnazi et al.	2010
Shahnazi et al.	2010
Shahnazi et al.	2010
Shahnazi et al.	2010
Shahnazi et al.	2010
Shahnazi et al.	2010
Shahnazi et al.	2010
Shahnazi et al.	2010
Shahnazi et al.	2010
Shahnazi et al.	2010
Shahnazi et al.	2010
Shahnazi et al.	2010
Shahnazi et al.	2010
Shahnazi et al.	2010
Shahnazi et al.	2010
Shahnazi et al.	2010
Shahnazi et al.	2010
Shahnazi et al.	2010
Shahnazi et al.	2010
Shahnazi et al.	2010
Shahnazi et al.	2010
Shahnazi et al.	2010
Shahnazi et al.	2010
Shahnazi et al.	2010
Shahnazi et al.	2010
Silva, et al	2014
Silva, et al	2014
Silva, et al	2014
Silva, et al	2014
Silva, et al	2014
Silva, et al	2014
Silva, et al	2014
Silva, et al	2014
Silva, et al	2014
Silva, et al	2014
Silva, et al	2014
Silva, et al	2014
SIM, S et al.	2017
SIM, S et al.	2017
SIM, S et al.	2017
SIM, S et al.	2017
SIM, S et al.	2017
SIM, S et al.	2017
SIM, S et al.	2017
SIM, S et al.	2017

SIM, S et al.	2017
SIM, S et al.	2017
SIMões et al	2001
SIMões et al	2001
SIMões et al	2001
SIMões et al	2001
SIMões et al	2001
SIMões et al	2001
SIMões et al	2001
SIMões et al	2001
SIMões et al	2001
SIMões et al	2001
SIMões et al	2001
SIMões et al	2001
SIMões et al	2001
SIMões et al	2001
SIMões et al	2001
SIMões et al	2001
SIMões et al	2001
SIMões et al	2001
SIMões et al	2001
SIMões et al	2001
SIMões et al	2001
SIMões et al	2001
SIMões et al	2001
SIMões et al	2001
SIMões et al	2001
SIMões et al	2001
SIMões et al	2001
SIMões et al	2001
SIMões et al	2001
SIMões et al	2001
SIMões et al	2001
SIMões et al	2001
Siyadatpanah, A et al.	2013
Siyadatpanah, A et al.	2013
Siyadatpanah, A et al.	2013
Siyadatpanah, A et al.	2013
Siyadatpanah, A et al.	2013
Siyadatpanah, A et al.	2013
Siyadatpanah, A et al.	2013
Siyadatpanah, A et al.	2013
Siyadatpanah, A et al.	2013
Siyadatpanah, A et al.	2013
Siyadatpanah, A et al.	2013
Siyadatpanah, A et al.	2013
Siyadatpanah, A et al.	2013
Siyadatpanah, A et al.	2013
Siyadatpanah, A et al.	2013
Siyadatpanah, A et al.	2013
Siyadatpanah, A et al.	2013

Siyadatpanah, A et al.	2013
Siyadatpanah, A et al.	2013
Siyadatpanah, A et al.	2013
Siyadatpanah, A et al.	2013
Siyadatpanah, A et al.	2013
Siyadatpanah, A et al.	2013
Siyadatpanah, A et al.	2013
Siyadatpanah, A et al.	2013
Siyadatpanah, A et al.	2013
Siyadatpanah, A et al.	2013
Siyadatpanah, A et al.	2013
Siyadatpanah, A et al.	2013
Siyadatpanah, A et al.	2013
Siyadatpanah, A et al.	2013
Siyadatpanah, A et al.	2013
Siyadatpanah, A et al.	2013
Siyadatpanah, A et al.	2013
Siyadatpanah, A et al.	2013
Siyadatpanah, A et al.	2013
Siyadatpanah, A et al.	2013
Siyadatpanah, A et al.	2013
Siyadatpanah, A et al.	2013
Siyadatpanah, A et al.	2013
Siyadatpanah, A et al.	2013
Siyadatpanah, A et al.	2013
Siyadatpanah, A et al.	2013
Siyadatpanah, A et al.	2013
Siyadatpanah, A et al.	2013
Siyadatpanah, A et al.	2013
Siyadatpanah, A et al.	2013
Siyadatpanah, A et al.	2013
Siyadatpanah, A et al.	2013
Siyadatpanah, A et al.	2013
Siyadatpanah, A et al.	2013
Siyadatpanah, A et al.	2013
Siyadatpanah, A et al.	2013
Siyadatpanah, A et al.	2013
Siyadatpanah, A et al.	2013
Siyadatpanah, A et al.	2013
Siyadatpanah, A et al.	2013
Siyadatpanah, A et al.	2013
Siyadatpanah, A et al.	2013
Siyadatpanah, A et al.	2013
Siyadatpanah, A et al.	2013
Siyadatpanah, A et al.	2013
Siyadatpanah, A et al.	2013
Siyadatpanah, A et al.	2013
Siyadatpanah, A et al.	2013
Siyadatpanah, A et al.	2013
Siyadatpanah, A et al.	2013
Siyadatpanah, A et al.	2013
Siyadatpanah, A et al.	2013
Siyadatpanah, A et al.	2013
Siyadatpanah, A et al.	2013

Siyadatpanah, A et al.	2013
Siyadatpanah, A et al.	2013
Siyadatpanah, A et al.	2013
Siyadatpanah, A et al.	2013
Siyadatpanah, A et al.	2013
Siyadatpanah, A et al.	2013
Siyadatpanah, A et al.	2013
Siyadatpanah, A et al.	2013
Siyadatpanah, A et al.	2013
Siyadatpanah, A et al.	2013
Siyadatpanah, A et al.	2013
Siyadatpanah, A et al.	2013
Siyadatpanah, A et al.	2013
Siyadatpanah, A et al.	2013
Siyadatpanah, A et al.	2013
Siyadatpanah, A et al.	2013
Siyadatpanah, A et al.	2013
Siyadatpanah, A et al.	2013
Siyadatpanah, A et al.	2013
Siyadatpanah, A et al.	2013
Siyadatpanah, A et al.	2013
Siyadatpanah, A et al.	2013
Siyadatpanah, A et al.	2013
Siyadatpanah, A et al.	2013
Siyadatpanah, A et al.	2013
Siyadatpanah, A et al.	2013
Siyadatpanah, A et al.	2013
Siyadatpanah, A et al.	2013
Siyadatpanah, A et al.	2013
Siyadatpanah, A et al.	2013
Siyadatpanah, A et al.	2013
Siyadatpanah, A et al.	2013
Siyadatpanah, A et al.	2013
Siyadatpanah, A et al.	2013
Siyadatpanah, A et al.	2013
Siyadatpanah, A et al.	2013
Siyadatpanah, A et al.	2013
Siyadatpanah, A et al.	2013
Siyadatpanah, A et al.	2013
Soares et al.	2006
Soares et al.	2006
Soares et al.	2006
Soares et al.	2006
Soares et al.	2006
Soares et al.	2006
Soares et al.	2006
Soares et al.	2006
Soares et al.	2006
Soares et al.	2006
Soares et al	2006

Soares et al.	2006
Soares et al.	2006
Soares et al.	2006
Soares et al.	2006
Soares et al.	2006
Soares et al.	2006
Soares et al.	2006
Soares et al.	2006
Soares et al.	2006
Soares et al.	2006
Soares et al.	2006
Soares et al.	2006
Soares et al.	2006
Soares et al.	2006
Soares et al.	2006
Soares et al.	2006
Soares et al.	2006
Soares et al.	2006
Soares et al.	2006
Soares et al.	2006
Soares et al.	2006
Soares et al.	2006
Soares et al.	2006
Soares et al.	2006
Soares et al.	2006
Srikanth & Durgadas	2004
Srikanth & Durgadas	2004
Srikanth & Durgadas	2004
Srikanth & Durgadas	2004
Srikanth & Durgadas	2004
Srikanth & Durgadas	2004
T Chaiphongpachara et al.	2019
T Chaiphongpachara et al.	2019
T Chaiphongpachara et al.	2019
T Chaiphongpachara et al.	2019
T Chaiphongpachara et al.	2019
T Chaiphongpachara et al.	2019
T Chaiphongpachara et al.	2019
T Chaiphongpachara et al.	2019
T Chaiphongpachara et al.	2019
T Chaiphongpachara et al.	2019
T Chaiphongpachara et al.	2019
T Chaiphongpachara et al.	2019
T Chaiphongpachara et al.	2019
T Chaiphongpachara et al.	2019
T Chaiphongpachara et al.	2019
T. Caradonna et al	2017
T. Caradonna et al	2017
T. Caradonna et al	2017
T. Caradonna et al	2017
T. Caradonna et al	2017
T. Caradonna et al	2017

T. Klapec et al.	2012
T. Klapec et al.	2012
T. Klapec et al.	2012
T. Klapec et al.	2012
T. Klapec et al.	2012
T. Klapec et al.	2012
T. Klapec et al.	2012
T. Klapec et al.	2012
T. Klapec et al.	2012
T. Klapec et al.	2012
T. Klapec et al.	2012
T. Klapec et al.	2012
T. Klapec et al.	2012
T. Klapec et al.	2012
T. Klapec et al.	2012
T. Klapec et al.	2012
T. Klapec et al.	2012
T. Klapec et al.	2012
T. Klapec et al.	2012
T. Klapec et al.	2012
T. Klapec et al.	2012
T. Klapec et al.	2012
T. Klapec et al.	2012
T. Klapec et al.	2012
T. Klapec et al.	2012
T. Klapec et al.	2012
T. Klapec et al.	2012
T. Klapec et al.	2012
T. Klapec et al.	2012
T. Klapec et al.	2012
T. Klapec et al.	2012
T. Klapec et al.	2012
T. Klapec et al.	2012
T. Klapec et al.	2012
T. Klapec et al.	2012
T. Klapec et al.	2012
T. Klapec et al.	2012
T. Klapec et al.	2012
T. Klapec et al.	2012
T. Klapec et al.	2012
T. Klapec et al.	2012
T.T. Nguyen et al	2016
T.T. Nguyen et al	2016
Taghipour, Ali et al.	2019
Taghipour, Ali et al.	2019
Taghipour, Ali et al.	2019
Taghipour, Ali et al.	2019
Taghipour, Ali et al.	2019
Taghipour, Ali et al.	2019
Taghipour, Ali et al.	2019

Taghipour, Ali et al.	2019
Taghipour, Ali et al.	2019
Tiyo et al.	2016
Tiyo et al.	2016
Tiyo et al.	2016
Tiyo et al.	2016
Tiyo et al.	2016
Tiyo et al.	2016
Tiyo et al.	2016
Tram et al.	2008
Tram et al.	2008
Tram et al.	2008
Tram et al.	2008
Tram et al.	2008
Tram et al.	2008
Tram et al.	2008
Tram et al.	2008
Tram et al.	2008
Tram et al.	2008
Utaaker, K S et al.	2017
Utaaker, K S et al.	2017
V. Hgraperova et al	2018
V. Hgraperova et al	2018
V. Hgraperova et al	2018
V. Hgraperova et al	2018
V. Hgraperova et al	2018
V. Hgraperova et al	2018
V. Hgraperova et al	2018
V. Hgraperova et al	2018
V. Hgraperova et al	2018
V. Hgraperova et al	2018
V. Hgraperova et al	2018
V. Hgraperova et al	2018
V. Hgraperova et al	2018
V. Hgraperova et al	2018
V. Hgraperova et al	2018
V. Hgraperova et al	2018
W. Istifanus et al	2018
W. Istifanus et al	2018
W. Istifanus et al	2018
W. Istifanus et al	2018
W. Istifanus et al	2018
W. Istifanus et al	2018
W. Istifanus et al	2018
W. Istifanus et al	2018
W. Istifanus et al	2018
W. Istifanus et al	2018
W. Istifanus et al	2018
W. Istifanus et al	2018
W. Istifanus et al	2018
W. Istifanus et al	2018
W. Istifanus et al	2018

W. Istifanus et al	2018
W. Istifanus et al	2018
W. Istifanus et al	2018
W. Istifanus et al	2018
W. Istifanus et al	2018
W. Istifanus et al	2018
W. Istifanus et al	2018
W. Istifanus et al	2018
W. Istifanus et al	2018
W. Istifanus et al	2018
W. Istifanus et al	2018
W. Istifanus et al	2018
W. Istifanus et al	2018
W. Istifanus et al	2018
W. Istifanus et al	2018
W. Istifanus et al	2018
W. Istifanus et al	2018
W. Istifanus et al	2018
W. Istifanus et al	2018
W. Istifanus et al	2018
W. Istifanus et al	2018
W. Istifanus et al	2018
W. Istifanus et al	2018
W. Istifanus et al	2018
W. Istifanus et al	2018
W. Istifanus et al	2018
W. Istifanus et al	2018
W. Istifanus et al	2018
W. Istifanus et al	2018
W. Istifanus et al	2018
W. Istifanus et al	2018
W. Istifanus et al	2018
W. Istifanus et al	2018
W. Istifanus et al	2018
W. Istifanus et al	2018
W. Istifanus et al	2018
W. Istifanus et al	2018
W. Istifanus et al	2018
W. Istifanus et al	2018
W. Istifanus et al	2018
W. Istifanus et al	2018
W. Istifanus et al	2018
W. Istifanus et al	2018
W. Istifanus et al	2018
W. Istifanus et al	2018
W. Istifanus et al	2018
W. Istifanus et al	2018
W. Istifanus et al	2018
W. Istifanus et al	2018
W. Istifanus et al	2018

W. Istifanus et al	2018
W. Istifanus et al	2018
W. Istifanus et al	2018
W. Istifanus et al	2018
W. Istifanus et al	2018
W. Khan	2017
W. Khan	2017
W. Khan	2017
W. Khan	2017
W. Khan	2017
W. Khan	2017
W. Khan	2017
W. Khan	2017
W. Khan	2017
W. Khan	2017
W. Khan	2017
W. Khan	2017
W. Khan	2017
W. Khan	2017
W. Khan	2017
W. Khan	2017
W. Khan	2017
W. Khan	2017
W. Khan	2017
W. Khan	2017
W. Khan	2017
W. Khan	2017
W. Khan	2017
W. Khan	2017
W. Khan	2017
W. Khan	2017
W. Khan	2017
W. Khan	2017
W. Khan	2017
W. Khan	2017
W. Khan	2017
W. Khan	2017
W. Khan	2017
W. Khan	2017
W. Khan	2017
W. Khan	2017
W. Khan	2017
W. Khan	2017
W. Khan	2017
W. Khan	2017
W. Khan	2017
W. Khan	2017
W. Khan	2017
W. Khan	2017
W. Khan	2017

W. Khan	2017
W. Khan	2017
W. Khan	2017
W. Khan	2017
W. Khan	2017
W. Khan	2017
W. Khan	2017
Xiuping Li, et al.	2020
Xiuping Li, et al.	2020
Xiuping Li, et al.	2020
Xiuping Li, et al.	2020
Xiuping Li, et al.	2020
Xiuping Li, et al.	2020
Xiuping Li, et al.	2020
Xiuping Li, et al.	2020
Xiuping Li, et al.	2020
Xiuping Li, et al.	2020
Xiuping Li, et al.	2020
Xiuping Li, et al.	2020
Xiuping Li, et al.	2020
Xiuping Li, et al.	2020
Xiuping Li, et al.	2020
Xiuping Li, et al.	2020
Xiuping Li, et al.	2020
Xiuping Li, et al.	2020
Xiuping Li, et al.	2020
Y. Ismail et al.	2016
Y. Ismail et al.	2016
Y. Ismail et al.	2016
Y. Ismail et al.	2016
Y. Ismail et al.	2016
Y. Ismail et al.	2016
Y. Ismail et al.	2016
Y. Ismail et al.	2016
Y. Ismail et al.	2016
Y. Ismail et al.	2016
Y. Ismail et al.	2016
Y. Ismail et al.	2016
Y. Ismail et al.	2016
Y. Ismail et al.	2016
Y. Ismail et al.	2016
Y. Ismail et al.	2016
Y. Ismail et al.	2016
Y. Ismail et al.	2016
Y. Ismail et al.	2016
Y. Ismail et al.	2016
Y. Ismail et al.	2016
Y. Ismail et al.	2016
Y. Ismail et al.	2016
Y. Ismail et al.	2016

Y. Ismail et al.	2016
Y. Ismail et al.	2016
Y. Ismail et al.	2016
Y. Ismail et al.	2016
Y. Ismail et al.	2016
Y. Ismail et al.	2016
Y. Ismail et al.	2016
Y. Ismail et al.	2016
Y. Ismail et al.	2016
Y. Ismail et al.	2016
Y. Ismail et al.	2016
Y. Ismail et al.	2016
Y. Ismail et al.	2016
Y. Ismail et al.	2016
Y. Ismail et al.	2016
Y. Ismail et al.	2016
Y. Ismail et al.	2016
Y. Ismail et al.	2016
Y. Ismail et al.	2016
Y. Ismail et al.	2016
Y. Ismail et al.	2016
Y. Ismail et al.	2016
Y. Ismail et al.	2016
Y. Ismail et al.	2016
Y. Ismail et al.	2016
Y. Ismail et al.	2016
Z. Salavati et al.	2017
Z. Salavati et al.	2017
Z. Salavati et al.	2017
Z. Salavati et al.	2017
Z. Salavati et al.	2017
Z. Salavati et al.	2017
Z. Salavati et al.	2017
Z. Salavati et al.	2017
Z. Salavati et al.	2017
Z. Salavati et al.	2017

PARASITES	VEGETABLES
Cryptosporidium spp.	basil
Cryptosporidium spp.	parsley
Cryptosporidium spp.	tarragon
Cryptosporidium spp.	mint
Cryptosporidium spp.	leek
Cryptosporidium spp.	coriander
Cryptosporidium spp.	chive
Cryptosporidium spp.	radish
Cryptosporidium spp.	lettuce
Ascaris spp.	multiple vegetables
Dicrocoelium spp.	multiple vegetables
Dicrocoelium spp.	multiple vegetables
Entamoeba coli	multiple vegetables
Entamoeba coli	multiple vegetables
Fasciola hepatica	multiple vegetables
Fasciola hepatica	multiple vegetables
Giardia lamblia	multiple vegetables
Giardia lamblia	multiple vegetables
Hymenolepis nana	multiple vegetables
Hymenolepis nana	multiple vegetables
Taenia spp.	multiple vegetables
Taenia spp.	multiple vegetables
Trichostrongylus spp.	multiple vegetables
Ascaris lumbricoides	pepper
Ascaris lumbricoides	pepper
Ascaris lumbricoides	tomato
Ascaris lumbricoides	cucumber
Ascaris lumbricoides	cucumber
Ascaris lumbricoides	pepper
Ascaris lumbricoides	tomato
Ascaris lumbricoides	cucumber
Ascaris lumbricoides	cucumber
Ascaris lumbricoides	pepper
Ascaris lumbricoides	tomato
Ascaris lumbricoides	lettuce
Ascaris lumbricoides	tomato
Ascaris lumbricoides	carrot
Ascaris lumbricoides	carrot
Ascaris lumbricoides	cabbage
Ascaris lumbricoides	cabbage
Ascaris lumbricoides	lettuce
Ascaris lumbricoides	onion
Cryptosporidium spp.	tomato
Cryptosporidium spp.	cucumber
Cryptosporidium spp.	cucumber
Cryptosporidium spp.	pepper
Cryptosporidium spp.	pepper
Cryptosporidium spp.	tomato
Cryptosporidium spp.	carrot

Cryptosporidium spp.	lettuce
Cryptosporidium spp.	cabbage
Cryptosporidium spp.	pepper
Cryptosporidium spp.	pepper
Cryptosporidium spp.	tomato
Cryptosporidium spp.	cucumber
Cryptosporidium spp.	tomato
Cryptosporidium spp.	onion
Cryptosporidium spp.	cucumber
Cryptosporidium spp.	carrot
Cryptosporidium spp.	cabbage
Cryptosporidium spp.	lettuce
Entamoeba coli	pepper
Entamoeba coli	tomato
Entamoeba coli	cucumber
Entamoeba coli	onion
Entamoeba coli	pepper
Entamoeba coli	tomato
Entamoeba coli	cucumber
Entamoeba coli	cabbage
Entamoeba coli	carrot
Entamoeba coli	lettuce
Enterobius vermicularis	pepper
Enterobius vermicularis	tomato
Enterobius vermicularis	cucumber
Enterobius vermicularis	cucumber
Enterobius vermicularis	pepper
Enterobius vermicularis	tomato
Enterobius vermicularis	pepper
Enterobius vermicularis	tomato
Enterobius vermicularis	onion
Enterobius vermicularis	cabbage
Enterobius vermicularis	pepper
Enterobius vermicularis	cucumber
Enterobius vermicularis	carrot
Enterobius vermicularis	lettuce
Enterobius vermicularis	cucumber
Enterobius vermicularis	cabbage
Enterobius vermicularis	carrot
Enterobius vermicularis	tomato
Enterobius vermicularis	lettuce
Strongyloides spp.	pepper
Strongyloides spp.	pepper
Strongyloides spp.	tomato
Strongyloides spp.	cucumber
Strongyloides spp.	tomato
Strongyloides spp.	onion
Strongyloides spp.	cucumber
Strongyloides spp.	cabbage
Strongyloides spp.	lettuce
Strongyloides spp.	carrot
Taenia spp.	pepper
Taenia spp.	pepper
Taenia spp.	tomato
Taenia spp.	cucumber
Taenia spp.	cucumber
Taenia spp.	tomato
Taenia spp.	onion
Taenia spp.	cabbage

Taenia spp.	lettuce
Taenia spp.	carrot
Toxocara spp.	pepper
Toxocara spp.	tomato
Toxocara spp.	cucumber
Toxocara spp.	onion
Toxocara spp.	tomato
Toxocara spp.	cucumber
Toxocara spp.	carrot
Toxocara spp.	pepper
Toxocara spp.	lettuce
Toxocara spp.	cabbage
Entamoeba histolytica	lettuce
Entamoeba histolytica	lettuce
hookworms	lettuce
hookworms	lettuce
hookworms	lettuce
hookworms	lettuce
hookworms	lettuce
hookworms	lettuce
hookworms	lettuce
hookworms	lettuce
hookworms	lettuce
hookworms	lettuce
Ancylostoma duadenale	parsley
Ancylostoma duadenale	potato
Ancylostoma duadenale	tomato
Ancylostoma duadenale	radish
Ancylostoma duadenale	cress
Ancylostoma duadenale	beetroot
Ancylostoma duadenale	green cabbage
Ancylostoma duadenale	turnip
Ancylostoma duadenale	celery
Ancylostoma duadenale	leek
Ancylostoma duadenale	anise
Ancylostoma duadenale	lettuce
Ascaris lumbricoides	parsley
Ascaris lumbricoides	tomato
Ascaris lumbricoides	cress
Ascaris lumbricoides	potato
Ascaris lumbricoides	radish
Ascaris lumbricoides	beetroot
Ascaris lumbricoides	green cabbage
Ascaris lumbricoides	turnip
Ascaris lumbricoides	lettuce
Ascaris lumbricoides	anise
Ascaris lumbricoides	celery
Ascaris lumbricoides	leek
Balantidium coli	potato
Balantidium coli	cress
Balantidium coli	tomato
Balantidium coli	parsley
Balantidium coli	radish
Balantidium coli	green cabbage
Balantidium coli	turnip
Balantidium coli	celery
Balantidium coli	leek
Balantidium coli	beetroot
Balantidium coli	lettuce
Balantidium coli	anise

Blastocystis hominis	potato
Blastocystis hominis	radish
Blastocystis hominis	cress
Blastocystis hominis	leek
Blastocystis hominis	parsley
Blastocystis hominis	green cabbage
Blastocystis hominis	celery
Blastocystis hominis	turnip
Blastocystis hominis	tomato
Blastocystis hominis	beetroot
Blastocystis hominis	anise
Blastocystis hominis	lettuce
Cryptosporidium parvum	potato
Cryptosporidium parvum	radish
Cryptosporidium parvum	tomato
Cryptosporidium parvum	parsley
Cryptosporidium parvum	cress
Cryptosporidium parvum	beetroot
Cryptosporidium parvum	green cabbage
Cryptosporidium parvum	lettuce
Cryptosporidium parvum	turnip
Cryptosporidium parvum	leek
Cryptosporidium parvum	celery
Cryptosporidium parvum	anise
Cyclospora cayetanensis	lettuce
Cyclospora cayetanensis	potato
Cyclospora cayetanensis	green cabbage
Cyclospora cayetanensis	tomato
Cyclospora cayetanensis	parsley
Cyclospora cayetanensis	anise
Cyclospora cayetanensis	radish
Cyclospora cayetanensis	beetroot
Cyclospora cayetanensis	turnip
Cyclospora cayetanensis	cress
Cyclospora cayetanensis	celery
Cyclospora cayetanensis	leek
Cyclospora spp.	tomato
Cyclospora spp.	potato
Cyclospora spp.	parsley
Cyclospora spp.	radish
Cyclospora spp.	cress
Cyclospora spp.	beetroot
Cyclospora spp.	leek
Cyclospora spp.	green cabbage
Cyclospora spp.	lettuce
Cyclospora spp.	turnip
Cyclospora spp.	celery
Cystoisospora spp.	anise
Entamoeba coli	parsley
Entamoeba coli	cress
Entamoeba coli	potato
Entamoeba coli	celery
Entamoeba coli	radish

Entamoeba coli	beetroot
Entamoeba coli	lettuce
Entamoeba coli	turnip
Entamoeba coli	tomato
Entamoeba coli	anise
Entamoeba coli	green cabbage
Entamoeba coli	leek
Entamoeba histolytica	potato
Entamoeba histolytica	radish
Entamoeba histolytica	cress
Entamoeba histolytica	tomato
Entamoeba histolytica	parsley
Entamoeba histolytica	leek
Entamoeba histolytica	beetroot
Entamoeba histolytica	green cabbage
Entamoeba histolytica	turnip
Entamoeba histolytica	celery
Entamoeba histolytica	anise
Entamoeba histolytica	lettuce
Fasciola hepatica	parsley
Fasciola hepatica	potato
Fasciola hepatica	cress
Fasciola hepatica	tomato
Fasciola hepatica	radish
Fasciola hepatica	celery
Fasciola hepatica	beetroot
Fasciola hepatica	green cabbage
Fasciola hepatica	turnip
Fasciola hepatica	leek
Fasciola hepatica	anise
Fasciola hepatica	lettuce
Giardia lamblia	tomato
Giardia lamblia	potato
Giardia lamblia	cress
Giardia lamblia	parsley
Giardia lamblia	radish
Giardia lamblia	green cabbage
Giardia lamblia	turnip
Giardia lamblia	beetroot
Giardia lamblia	celery
Giardia lamblia	leek
Giardia lamblia	anise
Giardia lamblia	lettuce
Hymenolepis nana	parsley
Hymenolepis nana	tomato
Hymenolepis nana	potato
Hymenolepis nana	radish
Hymenolepis nana	cress
Hymenolepis nana	celery
Hymenolepis nana	leek
Hymenolepis nana	beetroot
Hymenolepis nana	green cabbage

Hymenolepis nana	turnip
Hymenolepis nana	anise
Hymenolepis nana	lettuce
Strongyloides stercoralis	parsley
Strongyloides stercoralis	potato
Strongyloides stercoralis	tomato
Strongyloides stercoralis	radish
Strongyloides stercoralis	cress
Strongyloides stercoralis	green cabbage
Strongyloides stercoralis	beetroot
Strongyloides stercoralis	celery
Strongyloides stercoralis	turnip
Strongyloides stercoralis	leek
Strongyloides stercoralis	anise
Strongyloides stercoralis	lettuce
Taenia spp.	tomato
Taenia spp.	potato
Taenia spp.	parsley
Taenia spp.	radish
Taenia spp.	beetroot
Taenia spp.	turnip
Taenia spp.	cress
Taenia spp.	leek
Taenia spp.	celery
Taenia spp.	anise
Taenia spp.	green cabbage
Taenia spp.	lettuce
Toxocara spp.	tomato
Toxocara spp.	potato
Toxocara spp.	parsley
Toxocara spp.	cress
Toxocara spp.	radish
Toxocara spp.	beetroot
Toxocara spp.	green cabbage
Toxocara spp.	turnip
Toxocara spp.	leek
Toxocara spp.	lettuce
Toxocara spp.	celery
Toxocara spp.	anise
Trichuris trichiura	parsley
Trichuris trichiura	potato
Trichuris trichiura	tomato
Trichuris trichiura	radish
Trichuris trichiura	cress
Trichuris trichiura	beetroot
Trichuris trichiura	green cabbage
Trichuris trichiura	turnip
Trichuris trichiura	leek
Trichuris trichiura	celery
Trichuris trichiura	lettuce
Trichuris trichiura	anise
Ascaris spp.	chive

Ascaris spp.	mint
Ascaris spp.	shallot
Ascaris spp.	parsley
Ascaris spp.	spinach
Ascaris spp.	radish
Ascaris spp.	lettuce
hookworms	shallot
hookworms	chive
hookworms	radish
hookworms	lettuce
hookworms	spinach
hookworms	parsley
hookworms	mint
Hymenolepis nana	shallot
Hymenolepis nana	lettuce
Hymenolepis nana	chive
Hymenolepis nana	mint
Hymenolepis nana	radish
Hymenolepis nana	spinach
Hymenolepis nana	parsley
Taenia spp.	chive
Taenia spp.	radish
Taenia spp.	parsley
Taenia spp.	shallot
Taenia spp.	mint
Taenia spp.	spinach
Taenia spp.	lettuce
Toxocara spp.	radish
Toxocara spp.	mint
Toxocara spp.	shallot
Toxocara spp.	lettuce
Toxocara spp.	spinach
Toxocara spp.	parsley
Toxocara spp.	chive
Trichostrongylus spp.	mint
Trichostrongylus spp.	chive
Trichostrongylus spp.	shallot
Trichostrongylus spp.	parsley
Trichostrongylus spp.	spinach
Trichostrongylus spp.	lettuce
Trichostrongylus spp.	radish
Trichuris trichiura	chive
Trichuris trichiura	shallot
Trichuris trichiura	radish
Trichuris trichiura	lettuce
Trichuris trichiura	mint
Trichuris trichiura	spinach
Trichuris trichiura	parsley
Ascaris lumbricoides	cress
Ascaris lumbricoides	cabbage
Ascaris lumbricoides	lettuce

Entamoeba coli	chive
Entamoeba coli	cress
Entamoeba Histolytica	lettuce
Entamoeba Histolytica	cress
Enterobius vermicularis	lettuce
Enterobius vermicularis	cress
Enterobius vermicularis	chive
Giardia lamblia	cabbage
hookworms	mint
hookworms	cabbage
hookworms	chive
Ascaris lumbricoides	eggplant
Ascaris lumbricoides	boldo
Ascaris lumbricoides	cashew
Ascaris lumbricoides	mango
Ascaris lumbricoides	lettuce
Ascaris lumbricoides	tomato
Ascaris lumbricoides	spinach
Ascaris lumbricoides	carrot
Ascaris lumbricoides	pumpkin leaf
Ascaris lumbricoides	cabbage
hookworms	eggplant
hookworms	spinach
hookworms	boldo
hookworms	cashew
hookworms	carrot
hookworms	tomato
hookworms	lettuce
hookworms	mango
hookworms	pumpkin leaf
hookworms	cabbage
Strongyloides stercoralis	eggplant
Strongyloides stercoralis	lettuce
Strongyloides stercoralis	cashew
Strongyloides stercoralis	mango
Strongyloides stercoralis	boldo
Strongyloides stercoralis	carrot
Strongyloides stercoralis	pumpkin leaf
Strongyloides stercoralis	cabbage
Strongyloides stercoralis	tomato
Strongyloides stercoralis	spinach
Trichuris trichiura	eggplant
Trichuris trichiura	lettuce
Trichuris trichiura	cashew
Trichuris trichiura	mango
Trichuris trichiura	boldo
Trichuris trichiura	carrot
Trichuris trichiura	tomato
Trichuris trichiura	spinach
Trichuris trichiura	pumpkin leaf

Trichuris trichiura	cabbage
Ascaris spp.	tomato
Ascaris spp.	tomato
Ascaris spp.	lettuce
Ascaris spp.	cress
Ascaris spp.	cucumber
Ascaris spp.	cress
Ascaris spp.	cucumber
Ascaris spp.	lettuce
Giardia spp.	lettuce
Giardia spp.	tomato
Giardia spp.	cress
Giardia spp.	lettuce
Giardia spp.	tomato
Giardia spp.	cucumber
Giardia spp.	cress
Giardia spp.	cucumber
Taenia/Echinococcus	tomato
Taenia/Echinococcus	lettuce
Taenia/Echinococcus	cress
Taenia/Echinococcus	cucumber
Taenia/Echinococcus	tomato
Taenia/Echinococcus	cress
Taenia/Echinococcus	lettuce
Taenia/Echinococcus	cucumber
Toxocara canis	tomato
Toxocara canis	cucumber
Toxocara canis	tomato
Toxocara canis	cucumber
Toxocara canis	lettuce
Toxocara canis	cress
Toxocara canis	cress
Toxocara canis	lettuce
Toxocara cati	cress
Toxocara cati	cucumber
Toxocara cati	tomato
Toxocara cati	tomato
Toxocara cati	lettuce
Toxocara cati	cucumber
Toxocara cati	lettuce
Toxocara cati	cress
Ascaris spp.	eggplant
Ascaris spp.	lettuce
hookworms	cucumber
hookworms	lettuce
Strongyloides stercoralis	carrot
Strongyloides stercoralis	cabbage
Taenia/Echinococcus	lettuce
Trichuris spp.	lettuce
Trichuris spp.	eggplant

Ascaris lumbricoides	lettuce
Ascaris lumbricoides	parsley
Enterobius vermicularis	mint
Taenia spp.	cress
Taenia spp.	spinach
Taenia spp.	carrot
Toxocara spp.	mint
Toxocara spp.	arugula
Toxocara spp.	spinach
Ascaris lumbricoides	cress
Ascaris lumbricoides	coriander
Ascaris lumbricoides	tarragon
Ascaris lumbricoides	radish
Ascaris lumbricoides	mint
Ascaris lumbricoides	arugula
Ascaris lumbricoides	parsley
Ascaris lumbricoides	lettuce
Blastocystis spp.	tarragon
Blastocystis spp.	coriander
Blastocystis spp.	radish
Blastocystis spp.	arugula
Blastocystis spp.	mint
Blastocystis spp.	cress
Blastocystis spp.	parsley
Blastocystis spp.	lettuce
Entamoeba spp.	cress
Entamoeba spp.	coriander
Entamoeba spp.	tarragon
Entamoeba spp.	radish
Entamoeba spp.	arugula
Entamoeba spp.	mint
Entamoeba spp.	lettuce
Entamoeba spp.	parsley
Giardia lamblia	coriander
Giardia lamblia	tarragon
Giardia lamblia	mint
Giardia lamblia	cress
Giardia lamblia	radish
Giardia lamblia	arugula
Giardia lamblia	parsley
Giardia lamblia	lettuce
Hymenolepis nana	mint
Hymenolepis nana	coriander
Hymenolepis nana	tarragon
Hymenolepis nana	radish
Hymenolepis nana	arugula
Hymenolepis nana	parsley
Hymenolepis nana	lettuce
Hymenolepis nana	cress
Strongyloides spp.	lettuce

Strongyloides spp.	mint
Strongyloides spp.	cress
Strongyloides spp.	coriander
Strongyloides spp.	tarragon
Strongyloides spp.	radish
Strongyloides spp.	parsley
Strongyloides spp.	arugula
Taenia spp.	lettuce
Taenia spp.	mint
Taenia spp.	cress
Taenia spp.	coriander
Taenia spp.	tarragon
Taenia spp.	arugula
Taenia spp.	parsley
Taenia spp.	radish
Ancylostoma duodenale	leek
Ancylostoma duodenale	cress
Ancylostoma duodenale	lettuce
Ancylostoma duodenale	radish
Ancylostoma duodenale	chive
Ascaris lumbricoides	leek
Ascaris lumbricoides	cress
Ascaris lumbricoides	lettuce
Ascaris lumbricoides	radish
Blastocystis hominis	lettuce
Blastocystis hominis	cress
Blastocystis hominis	radish
Blastocystis hominis	chive
Cryptosporidium parvum	leek
Cryptosporidium parvum	cress
Entamoeba coli	radish
Entamoeba coli	leek
Entamoeba coli	cress
Entamoeba coli	chive
Entamoeba histolytica	radish
Entamoeba histolytica	chive
Enterobius vermicularis	lettuce
Enterobius vermicularis	radish
Giardia lamblia	leek
Giardia lamblia	lettuce
Iodamoeba butschlii	leek
Strongyloides stercoralis	cress
Strongyloides stercoralis	radish
Strongyloides stercoralis	chive
Toxocara canis	lettuce
Toxocara canis	radish
Trichostrongylus colubriformes	chive
Ascaris lumbricoides	ready-to-eat salad
Ascaris lumbricoides	bell pepper
Ascaris lumbricoides	cabbage

Ascaris lumbricoides	carrot
Ascaris lumbricoides	tomato
Balantidium coli	tomato
Balantidium coli	ready-to-eat salad
Balantidium coli	carrot
Balantidium coli	cabbage
Balantidium coli	bell pepper
Cryptosporidium spp.	cabbage
Cryptosporidium spp.	ready-to-eat salad
Cryptosporidium spp.	bell pepper
Cryptosporidium spp.	carrot
Cryptosporidium spp.	tomato
Cyclospora spp.	carrot
Cyclospora spp.	cabbage
Cyclospora spp.	ready-to-eat salad
Cyclospora spp.	bell pepper
Cyclospora spp.	tomato
Entamoeba histolytica	ready-to-eat salad
Entamoeba histolytica	bell pepper
Entamoeba histolytica	cabbage
Entamoeba histolytica	carrot
Entamoeba histolytica	tomato
Giardia lamblia	ready-to-eat salad
Giardia lamblia	tomato
Giardia lamblia	carrot
Giardia lamblia	bell pepper
Giardia lamblia	cabbage
hookworms	ready-to-eat salad
hookworms	carrot
hookworms	bell pepper
hookworms	tomato
hookworms	cabbage
Strongyloides spp.	ready-to-eat salad
Strongyloides spp.	carrot
Strongyloides spp.	bell pepper
Strongyloides spp.	cabbage
Strongyloides spp.	tomato
Ascaris lumbricoides	radish
Ascaris lumbricoides	mint
Ascaris lumbricoides	coriander
Ascaris lumbricoides	lettuce
Ascaris lumbricoides	parsley
Entamoeba spp.	radish
Entamoeba spp.	coriander
Entamoeba spp.	mint
Entamoeba spp.	lettuce
Entamoeba spp.	parsley
Enterobius vermicularis	radish
Enterobius vermicularis	coriander
Enterobius vermicularis	mint

Enterobius vermicularis	lettuce
Enterobius vermicularis	parsley
Giardia lamblia	coriander
Giardia lamblia	radish
Giardia lamblia	mint
Giardia lamblia	lettuce
Giardia lamblia	parsley
Ascaris lumbricoides	radish
Ascaris lumbricoides	mint
Ascaris lumbricoides	chive
Endolimax nana	chard
Endolimax nana	tomato
Entamoeba coli	cucumber
Entamoeba coli	radish
Entamoeba coli	cress
Entamoeba coli	chive
Entamoeba coli	mint
Entamoeba complex	cucumber
Entamoeba complex	mint
Entamoeba complex	cress
Entamoeba complex	broccoli
Entamoeba complex	radish
Entamoeba complex	chive
Enterobius vermicularis	broccoli
Enterobius vermicularis	parsley
Giardia lamblia	lettuce
Hymenolepis nana	lettuce
Strongyloides stercoralis	radish
Strongyloides stercoralis	lettuce
Strongyloides stercoralis	cress
Strongyloides stercoralis	chive
Trichuris trichiura	parsley
Trichuris trichiura	cress
Trichuris trichiura	chive
Ancylostoma spp.	multiple vegetables
Ascaris spp.	multiple vegetables
Blastocystis hominis	multiple vegetables
Dicrocoelium spp.	multiple vegetables
Entamoeba coli	multiple vegetables
Fasciola spp.	multiple vegetables
Giardia lamblia	multiple vegetables
Hymenolepis spp.	multiple vegetables
Taenia spp.	multiple vegetables
Toxoplasma gondii	multiple vegetables
Trichostrongylus spp.	multiple vegetables
Ascaris lumbricoides	lettuce
Ascaris spp.	parsley
Balantidium coli	lettuce
Cystoisospora spp.	parsley
Dipylidium caninum	lettuce

Entamoeba coli	parsley
Entamoeba coli	lettuce
Entamoeba histolytica/dispar	lettuce
Enterobius vermicularis	lettuce
Fasciola hepatica	parsley
Fasciola hepatica	lettuce
Giardia lamblia	lettuce
hookworms	parsley
hookworms	lettuce
Hymenolepis nana	lettuce
Hymenolepis spp.	parsley
Oxyuridae	parsley
Oxyuridae	lettuce
Strongylidae	parsley
Strongyloides stercoralis	lettuce
Toxocara cati	lettuce
Toxocara spp.	lettuce
Trichuris spp.	lettuce
Cryptosporidium spp.	lettuce
Cryptosporidium spp.	cabbage
Cryptosporidium spp.	lettuce
Giardia spp.	lettuce
Giardia spp.	cabbage
Giardia spp.	lettuce
Ascaris lumbricoides	multiple fruits
Ascaris lumbricoides	multiple vegetables
hookworms	multiple fruits
hookworms	multiple vegetables
Paramphistomum spp.	multiple vegetables
Paramphistomum spp.	multiple fruits
Strongyloides spp.	multiple fruits
Strongyloides spp.	multiple vegetables
Taenia spp.	multiple vegetables
Taenia spp.	multiple fruits
Toxocara canis	multiple fruits
Toxocara canis	multiple vegetables
Trichuris spp.	multiple fruits
Trichuris spp.	multiple vegetables
Cryptosporidium spp.	spinach
Cyclospora spp.	spinach
Giardia spp.	spinach
helminths	spinach
Entamoeba spp.	tomato
Entamoeba spp.	carrot
Entamoeba spp.	lettuce
hookworms	tomato
hookworms	carrot
hookworms	lettuce
nematode	carrot
nematode	tomato

nematode	lettuce
nematode	carrot
nematode	tomato
nematode	lettuce
Toxocara spp.	tomato
Toxocara spp.	lettuce
Toxocara spp.	carrot
Cyclospora spp.	cauliflower
Cyclospora spp.	chive
Cyclospora spp.	radish
Cyclospora spp.	carrot
Cyclospora spp.	cabbage
Cyclospora spp.	mustard
Cyclospora spp.	spinach
Cyclospora spp.	lettuce
Cryptosporidium spp.	lettuce
Cryptosporidium spp.	multiple vegetables
Giardia spp.	lettuce
Giardia spp.	multiple vegetables
Ascaris lumbricoides	tomato
Ascaris lumbricoides	carrot
Ascaris lumbricoides	bell pepper
Ascaris lumbricoides	cabbage
Ascaris lumbricoides	eggplant
Ascaris lumbricoides	broadleaf basil
Ascaris lumbricoides	pepper
Ascaris lumbricoides	gangronema
Ascaris lumbricoides	pumpkin leaf
Ascaris lumbricoides	purslane
hookworms	gangronema
hookworms	carrot
hookworms	pepper
hookworms	eggplant
hookworms	broadleaf basil
hookworms	pumpkin leaf
hookworms	bell pepper
hookworms	cabbage
hookworms	tomato
hookworms	purslane
Strongyloides stercoralis	gangronema
Strongyloides stercoralis	purslane
Strongyloides stercoralis	bell pepper
Strongyloides stercoralis	tomato
Strongyloides stercoralis	eggplant
Strongyloides stercoralis	carrot
Strongyloides stercoralis	pepper
Strongyloides stercoralis	cabbage
Strongyloides stercoralis	broadleaf basil
Strongyloides stercoralis	pumpkin leaf
Trichuris trichiura	eggplant

Trichuris trichiura	broadleaf basil
Trichuris trichiura	carrot
Trichuris trichiura	pumpkin leaf
Trichuris trichiura	cabbage
Trichuris trichiura	purslane
Trichuris trichiura	pepper
Trichuris trichiura	bell pepper
Trichuris trichiura	tomato
Trichuris trichiura	gangronema
Ancylostoma spp.	multiple vegetables
Enterobius spp.	multiple vegetables
Strongyloides spp.	multiple vegetables
Taenia spp.	multiple vegetables
Toxocara spp.	multiple vegetables
Trichuris spp.	multiple vegetables
Ascaris spp.	tomato
Ascaris spp.	beetroot
Ascaris spp.	potato
Ascaris spp.	carrot
Ascaris spp.	mint
Ascaris spp.	coriander
Ascaris spp.	spinach
Ascaris spp.	ginger
Ascaris spp.	onion
Ascaris spp.	cabbage
Ascaris lumbricoides	multiple vegetables
Cryptosporidium spp.	multiple vegetables
Cyclospora spp.	multiple vegetables
Cyclospora spp.	multiple vegetables
Entamoeba histolytica/dispar	multiple vegetables
Giardia lamblia	multiple vegetables
Hymenolepis diminuta	multiple vegetables
Hymenolepis nana	multiple vegetables
Toxocara spp.	multiple vegetables
Ascaris lumbricoides	tomato
Ascaris lumbricoides	avocado
Ascaris lumbricoides	carrot
Ascaris lumbricoides	lettuce
Ascaris lumbricoides	bell pepper
Ascaris lumbricoides	cabbage
Cyclospora spp.	avocado
Cyclospora spp.	bell pepper
Cyclospora spp.	tomato
Cyclospora spp.	lettuce
Cyclospora spp.	carrot
Cyclospora spp.	cabbage
Entamoeba histolytica/dispar	tomato
Entamoeba histolytica/dispar	lettuce
Entamoeba histolytica/dispar	avocado
Entamoeba histolytica/dispar	bell pepper
Entamoeba histolytica/dispar	carrot
Entamoeba histolytica/dispar	cabbage
Giardia lamblia	avocado
Giardia lamblia	tomato
Giardia lamblia	lettuce
Giardia lamblia	bell pepper

Giardia lamblia	carrot
Giardia lamblia	cabbage
Hymenolepis diminuta	tomato
Hymenolepis diminuta	lettuce
Hymenolepis diminuta	avocado
Hymenolepis diminuta	carrot
Hymenolepis diminuta	bell pepper
Hymenolepis diminuta	cabbage
Hymenolepis nana	tomato
Hymenolepis nana	avocado
Hymenolepis nana	carrot
Hymenolepis nana	lettuce
Hymenolepis nana	bell pepper
Hymenolepis nana	cabbage
Toxocara spp.	tomato
Toxocara spp.	avocado
Toxocara spp.	bell pepper
Toxocara spp.	carrot
Toxocara spp.	lettuce
Toxocara spp.	cabbage
Cryptosporidium spp.	lettuce
Cryptosporidium spp.	coriander
Cryptosporidium spp.	parsley
Giardia lamblia	lettuce
Giardia lamblia	parsley
Giardia lamblia	coriander
Toxoplasma gondii	radish
Toxoplasma gondii	lettuce
Toxoplasma gondii	carrot
Toxoplasma gondii	coriander
Toxoplasma gondii	parsley
Toxoplasma gondii	lettuce
Toxoplasma gondii	lettuce
Toxoplasma gondii	lettuce
Toxoplasma gondii	lettuce
Toxoplasma gondii	lettuce
Toxoplasma gondii	raspberry
Toxoplasma gondii	raspberry
Toxoplasma gondii	ready-to-eat salad
Toxoplasma gondii	ready-to-eat salad
Toxoplasma gondii	ready-to-eat salad
Toxoplasma gondii	ready-to-eat salad
Toxoplasma gondii	cress
Toxoplasma gondii	carrot
Toxoplasma gondii	coriander
Toxoplasma gondii	blueberry
Toxoplasma gondii	blueberry
Toxoplasma gondii	arugula
Toxoplasma gondii	parsley
Toxoplasma gondii	parsley
Toxoplasma gondii	parsley

Ascaris lumbricoides	parsley
Ascaris lumbricoides	lettuce
Ascaris lumbricoides	coriander
hookworms	cabbage
hookworms	coriander
hookworms	mint
hookworms	centella
hookworms	leek
hookworms	coriander
hookworms	parsley
hookworms	lettuce
Strongyloides stercoralis	Chinese morning glory
Strongyloides stercoralis	basil
Strongyloides stercoralis	cabbage
Strongyloides stercoralis	mint
Strongyloides stercoralis	coriander
Strongyloides stercoralis	coriander
Strongyloides stercoralis	centella
Strongyloides stercoralis	leek
Strongyloides stercoralis	parsley
Toxocara spp.	mint
Toxocara spp.	centella
Toxocara spp.	basil
Toxocara spp.	coriander
Toxocara spp.	cabbage
Trichuris trichiura	Chinese morning glory
Trichuris trichiura	basil
Trichuris trichiura	cabbage
Trichuris trichiura	coriander
Ascaris spp.	parsley
Ascaris spp.	chive
Ascaris spp.	leek
Ascaris spp.	bell pepper
Ascaris spp.	celery
Ascaris spp.	cabbage
Ascaris spp.	onion
Ascaris spp.	lettuce
Ascaris spp.	tomato
Ascaris spp.	coriander
Blastocystis hominis	parsley
Blastocystis hominis	leek
Blastocystis hominis	bell pepper
Blastocystis hominis	celery
Blastocystis hominis	onion
Blastocystis hominis	cabbage
Blastocystis hominis	tomato
Blastocystis hominis	coriander
Blastocystis hominis	lettuce
Blastocystis hominis	chive
Cryptosporidium spp.	parsley

Cryptosporidium spp.	leek
Cryptosporidium spp.	bell pepper
Cryptosporidium spp.	onion
Cryptosporidium spp.	tomato
Cryptosporidium spp.	coriander
Cryptosporidium spp.	chive
Cryptosporidium spp.	celery
Cryptosporidium spp.	cabbage
Cryptosporidium spp.	lettuce
Cyclospora spp.	parsley
Cyclospora spp.	leek
Cyclospora spp.	bell pepper
Cyclospora spp.	onion
Cyclospora spp.	tomato
Cyclospora spp.	coriander
Cyclospora spp.	celery
Cyclospora spp.	lettuce
Cyclospora spp.	cabbage
Cyclospora spp.	chive
Dipylidium caninum	parsley
Dipylidium caninum	leek
Dipylidium caninum	bell pepper
Dipylidium caninum	celery
Dipylidium caninum	onion
Dipylidium caninum	cabbage
Dipylidium caninum	lettuce
Dipylidium caninum	tomato
Dipylidium caninum	chive
Dipylidium caninum	coriander
Entamoeba coli	parsley
Entamoeba coli	leek
Entamoeba coli	bell pepper
Entamoeba coli	onion
Entamoeba coli	cabbage
Entamoeba coli	lettuce
Entamoeba coli	tomato
Entamoeba coli	coriander
Entamoeba coli	chive
Entamoeba coli	celery
Entamoeba histolytica/dispar	parsley
Entamoeba histolytica/dispar	leek
Entamoeba histolytica/dispar	bell pepper
Entamoeba histolytica/dispar	celery
Entamoeba histolytica/dispar	onion
Entamoeba histolytica/dispar	lettuce
Entamoeba histolytica/dispar	tomato
Entamoeba histolytica/dispar	coriander
Entamoeba histolytica/dispar	chive
Entamoeba histolytica/dispar	cabbage
hookworms	parsley
hookworms	leek
hookworms	bell pepper

hookworms	celery
hookworms	onion
hookworms	cabbage
hookworms	lettuce
hookworms	tomato
hookworms	coriander
hookworms	chive
Toxocara canis	parsley
Toxocara canis	leek
Toxocara canis	bell pepper
Toxocara canis	celery
Toxocara canis	onion
Toxocara canis	lettuce
Toxocara canis	coriander
Toxocara canis	chive
Toxocara canis	cabbage
Toxocara canis	tomato
Giardia spp.	endive
Giardia spp.	lettuce
Ascaris spp.	chive
Ascaris spp.	leek
Ascaris spp.	parsley
Ascaris spp.	arugula
Ascaris spp.	lettuce
Cryptosporidium spp.	chive
Cryptosporidium spp.	leek
Cryptosporidium spp.	lettuce
Cryptosporidium spp.	parsley
Cryptosporidium spp.	arugula
Cyclospora cayetanensis	chive
Cyclospora cayetanensis	leek
Cyclospora cayetanensis	parsley
Cyclospora cayetanensis	arugula
Cyclospora cayetanensis	lettuce
Giardia spp.	leek
Giardia spp.	parsley
Giardia spp.	arugula
Giardia spp.	lettuce
Hymenolepis nana	arugula
Hymenolepis nana	parsley
Hymenolepis nana	lettuce
Microsporidia spp.	leek
Microsporidia spp.	chive
Microsporidia spp.	arugula
Microsporidia spp.	lettuce
Microsporidia spp.	parsley
Toxocara spp.	leek
Toxocara spp.	chive
Toxocara spp.	parsley
Toxocara spp.	arugula
Toxocara spp.	lettuce
Eimeria spp.	ready-to-eat salad

Toxoplasma gondii	thyme
Toxoplasma gondii	lettuce
Toxoplasma gondii	thyme
Toxoplasma gondii	lettuce
Toxoplasma gondii	parsley
Toxoplasma gondii	cress
Toxoplasma gondii	dill
Toxoplasma gondii	mint
Toxoplasma gondii	parsley
Toxoplasma gondii	cress
Toxoplasma gondii	dill
Toxoplasma gondii	mint
Cryptosporidium parvum	ready-to-eat salad
Cyclospora cayetanensis	ready-to-eat salad
Giardia lamblia	ready-to-eat salad
Ascaris lumbricoides	leek
Ascaris lumbricoides	cress
Ascaris lumbricoides	lettuce
Entamoeba spp.	leek
Entamoeba spp.	cress
Entamoeba spp.	lettuce
Enterobius vermicularis	leek
Enterobius vermicularis	lettuce
Enterobius vermicularis	cress
Giardia lamblia	leek
Giardia lamblia	cress
Giardia lamblia	lettuce
helminths	leek
helminths	cress
helminths	lettuce
Hymenolepis nana	leek
Hymenolepis nana	lettuce
Hymenolepis nana	cress
Ascaris lumbricoides	chive
Entamoeba spp.	chive
Enterobius vermicularis	chive
Giardia lamblia	chive
helminths	chive
Hymenolepis nana	chive
Ascaris lumbricoides	parsley
Entamoeba spp.	parsley
Enterobius vermicularis	parsley
Giardia lamblia	parsley
helminths	parsley
Hymenolepis nana	parsley
Ascaris spp.	cress
Ascaris spp.	spinach
Ascaris spp.	parsley
Ascaris spp.	lettuce
Ascaris spp.	strawberry
Entamoeba histolytica	spinach
Entamoeba histolytica	cress
Entamoeba histolytica	lettuce

Entamoeba histolytica	parsley
Entamoeba histolytica	strawberry
Enterobius vermicularis	cress
Enterobius vermicularis	spinach
Enterobius vermicularis	lettuce
Enterobius vermicularis	parsley
Enterobius vermicularis	strawberry
Giardia spp.	spinach
Giardia spp.	cress
Giardia spp.	parsley
Giardia spp.	lettuce
Giardia spp.	strawberry
Ascaris spp.	radish
Ascaris spp.	cress
Ascaris spp.	mint
Ascaris spp.	leek
Ascaris spp.	chive
Endolimax nana	mint
Endolimax nana	chive
Endolimax nana	cress
Endolimax nana	leek
Endolimax nana	radish
Entamoeba coli	mint
Entamoeba coli	cress
Entamoeba coli	radish
Entamoeba coli	leek
Entamoeba coli	chive
Enterobius vermicularis	mint
Enterobius vermicularis	cress
Enterobius vermicularis	chive
Enterobius vermicularis	radish
Enterobius vermicularis	leek
Fasciola hepatica	leek
Fasciola hepatica	chive
Fasciola hepatica	radish
Fasciola hepatica	cress
Fasciola hepatica	mint
Giardia spp.	chive
Giardia spp.	radish
Giardia spp.	mint
Giardia spp.	leek
Giardia spp.	cress
Iodamoeba butschlii	mint
Iodamoeba butschlii	chive
Iodamoeba butschlii	cress
Iodamoeba butschlii	leek
Iodamoeba butschlii	radish
Strongyloides stercoralis	mint
Strongyloides stercoralis	cress
Strongyloides stercoralis	leek
Strongyloides stercoralis	chive
Strongyloides stercoralis	radish
Ascaris lumbricoides	multiple vegetables

Entamoeba histolytica/dispar	multiple vegetables
Giardia lamblia	multiple vegetables
Haemonchus contortus	multiple vegetables
Hymenolepis diminuta	multiple vegetables
Hymenolepis nana	multiple vegetables
Toxocara spp.	multiple vegetables
Ascaris lumbricoides	tarragon
Ascaris lumbricoides	dill
Ascaris lumbricoides	parsley
Ascaris lumbricoides	coriander
Ascaris lumbricoides	purslane
Ascaris lumbricoides	mint
Ascaris lumbricoides	chive
Ascaris lumbricoides	leek
Ascaris lumbricoides	radish
Ascaris lumbricoides	cress
Ascaris lumbricoides	basil
Entamoeba coli	purslane
Entamoeba coli	chive
Entamoeba coli	tarragon
Entamoeba coli	parsley
Entamoeba coli	radish
Entamoeba coli	basil
Entamoeba coli	leek
Entamoeba coli	cress
Entamoeba coli	coriander
Entamoeba coli	mint
Entamoeba coli	dill
Giardia spp.	tarragon
Giardia spp.	dill
Giardia spp.	parsley
Giardia spp.	coriander
Giardia spp.	chive
Giardia spp.	cress
Giardia spp.	purslane
Giardia spp.	radish
Giardia spp.	basil
Giardia spp.	mint
Giardia spp.	leek
Taenia spp.	purslane
Taenia spp.	chive
Taenia spp.	tarragon
Taenia spp.	mint
Taenia spp.	dill
Taenia spp.	radish
Taenia spp.	coriander
Taenia spp.	leek
Taenia spp.	parsley
Taenia spp.	cress
Taenia spp.	basil
Toxocara spp.	purslane
Toxocara spp.	tarragon
Toxocara spp.	mint
Toxocara spp.	dill

Toxocara spp.	parsley
Toxocara spp.	basil
Toxocara spp.	coriander
Toxocara spp.	leek
Toxocara spp.	chive
Toxocara spp.	radish
Toxocara spp.	cress
Trichostrongylus spp.	leek
Trichostrongylus spp.	dill
Trichostrongylus spp.	radish
Trichostrongylus spp.	cress
Trichostrongylus spp.	coriander
Trichostrongylus spp.	tarragon
Trichostrongylus spp.	chive
Trichostrongylus spp.	basil
Trichostrongylus spp.	mint
Trichostrongylus spp.	parsley
Trichostrongylus spp.	purslane
Cryptosporidium spp.	lettuce
Cryptosporidium spp.	arugula
Giardia spp.	chard
Giardia spp.	spinach
Giardia spp.	chive
Giardia spp.	arugula
Giardia spp.	endive
Giardia spp.	lettuce
Toxoplasma gondii	arugula
Toxoplasma gondii	lettuce
Ascaris lumbricoides	carrot
Ascaris lumbricoides	cucumber
Ascaris lumbricoides	lettuce
Ascaris lumbricoides	pea
Ascaris lumbricoides	cabbage
Entamoeba coli	tomato
Entamoeba coli	lettuce
Entamoeba coli	cucumber
Entamoeba coli	chive
Entamoeba coli	carrot
Entamoeba coli	pea
Entamoeba coli	cress
Entamoeba coli	cabbage
Entamoeba spp.	pea
Entamoeba spp.	chive
Entamoeba spp.	carrot
Entamoeba spp.	lettuce
Entamoeba spp.	cabbage
Entamoeba spp.	cucumber
Entamoeba spp.	cress
Giardia spp.	carrot
Hymenolepis nana	chive
Hymenolepis nana	carrot
Strongyloides stercoralis	tomato
Strongyloides stercoralis	chive
Strongyloides stercoralis	cucumber
Taenia spp.	cabbage
Taenia spp.	lettuce
Taenia spp.	cress
Taenia spp.	pea

Cryptosporidium spp.	lettuce
Cryptosporidium spp.	chive
Cryptosporidium spp.	cucumber
Cryptosporidium spp.	tomato
Cryptosporidium spp.	carrot
Cryptosporidium spp.	cress
Cryptosporidium spp.	radish
Cryptosporidium spp.	parsley
Cryptosporidium spp.	dill
Cryptosporidium spp.	cress
Cryptosporidium spp.	chive
Cryptosporidium spp.	dill
Cryptosporidium spp.	cucumber
Cryptosporidium spp.	radish
Cryptosporidium spp.	parsley
Cryptosporidium spp.	lettuce
Cryptosporidium spp.	carrot
Cryptosporidium spp.	tomato
Entamoeba histolytica	chive
Entamoeba histolytica	tomato
Entamoeba histolytica	cress
Entamoeba histolytica	radish
Entamoeba histolytica	carrot
Entamoeba histolytica	cucumber
Entamoeba histolytica	lettuce
Entamoeba histolytica	dill
Entamoeba histolytica	parsley
Entamoeba histolytica	carrot
Entamoeba histolytica	radish
Entamoeba histolytica	parsley
Entamoeba histolytica	cucumber
Entamoeba histolytica	lettuce
Entamoeba histolytica	chive
Entamoeba histolytica	cress
Entamoeba histolytica	tomato
Entamoeba histolytica	dill
Microsporidia spp.	cucumber
Microsporidia spp.	cress
Microsporidia spp.	carrot
Microsporidia spp.	radish
Microsporidia spp.	tomato
Microsporidia spp.	chive
Microsporidia spp.	parsley
Microsporidia spp.	dill
Microsporidia spp.	lettuce
Microsporidia spp.	radish
Microsporidia spp.	chive
Microsporidia spp.	cucumber
Microsporidia spp.	lettuce
Microsporidia spp.	carrot
Microsporidia spp.	cress
Microsporidia spp.	parsley
Microsporidia spp.	dill
Microsporidia spp.	tomato
Ancylostoma spp.	cabbage
roundworms	lettuce
roundworms	cabbage

Ascaris spp.	lettuce
Ascaris spp.	cabbage
Entamoeba coli	cabbage
Entamoeba histolytica	cabbage
Enterobius vermicularis	lettuce
Enterobius vermicularis	cabbage
Giardia lamblia	cabbage
Trichostrongylus spp.	lettuce
Trichostrongylus spp.	cabbage
Trichuris trichiura	lettuce
Trichuris trichiura	cabbage
Ascaris lumbricoides	lettuce
Ascaris lumbricoides	lettuce
Ascaris lumbricoides	lettuce
Endolimax nana	lettuce
Endolimax nana	lettuce
Endolimax nana	lettuce
Entamoeba coli	lettuce
Entamoeba coli	lettuce
Entamoeba coli	lettuce
Entamoeba histolytica	lettuce
Entamoeba histolytica	lettuce
Entamoeba histolytica	lettuce
Giardia lamblia	lettuce
Giardia lamblia	lettuce
Giardia lamblia	lettuce
hookworms	lettuce
hookworms	lettuce
hookworms	lettuce
Iodamoeba butschlii	lettuce
Iodamoeba butschlii	lettuce
Iodamoeba butschlii	lettuce
Strongyloides stercoralis	lettuce
Strongyloides stercoralis	lettuce
Strongyloides stercoralis	lettuce
Taenia spp.	lettuce
Taenia spp.	lettuce
Taenia spp.	lettuce
Trichostrongylus spp.	lettuce
Trichostrongylus spp.	lettuce
Trichostrongylus spp.	lettuce
Entamoeba spp.	lettuce
Entamoeba spp.	lettuce
Entamoeba spp.	lettuce
Entamoeba spp.	lettuce
nematode	lettuce
nematode	lettuce
nematode	lettuce
nematode	lettuce
nematode	lettuce
nematode	lettuce
nematode	lettuce
nematode	lettuce
unsporulated oocyst	lettuce
unsporulated oocyst	lettuce
unsporulated oocyst	lettuce
Strongyloides spp.	lettuce
Strongyloides spp.	lettuce

Strongyloides spp.	lettuce
Strongyloides spp.	lettuce
Toxocara spp.	lettuce
Toxocara spp.	lettuce
Toxocara spp.	lettuce
unsporulated oocyst	lettuce
Ancylostoma duodenale	multiple vegetables
Ascaris lumbricoides	multiple vegetables
Balantidium coli	multiple vegetables
cocids	multiple vegetables
Diphyllobothrium latum	multiple vegetables
Dipylidium caninum	multiple vegetables
Entamoeba coli	multiple vegetables
Entamoeba histolytica	multiple vegetables
Enterobius vermicularis	multiple vegetables
Fasciola hepatica	multiple vegetables
Giardia lamblia	multiple vegetables
Heterophyes heterophyes	multiple vegetables
Hymenolepis nana	multiple vegetables
nematode	multiple vegetables
Schistosoma haematobium	multiple vegetables
Schistosoma japonicum	multiple vegetables
Schistosoma mansoni	multiple vegetables
Strongyloides stercoralis	multiple vegetables
Taenia saginata	multiple vegetables
Trichomonas hominis	multiple vegetables
Trichuris trichiura	multiple vegetables
Ascaris lumbricoides	mint
Ascaris lumbricoides	radish
Ascaris lumbricoides	parsley
Ascaris lumbricoides	cress
Ascaris lumbricoides	lettuce
Ascaris lumbricoides	celery
Echinococcus spp.	mint
Echinococcus spp.	radish
Echinococcus spp.	parsley
Echinococcus spp.	cress
Echinococcus spp.	lettuce
Echinococcus spp.	celery
Entamoeba histolytica	mint
Entamoeba histolytica	radish
Entamoeba histolytica	cress
Entamoeba histolytica	parsley
Entamoeba histolytica	lettuce
Entamoeba histolytica	celery
Fasciola hepatica	mint
Fasciola hepatica	radish
Fasciola hepatica	cress
Fasciola hepatica	parsley
Fasciola hepatica	lettuce
Fasciola hepatica	celery
Giardia lamblia	mint
Giardia lamblia	radish
Giardia lamblia	parsley
Giardia lamblia	cress
Giardia lamblia	lettuce
Giardia lamblia	celery
hookworms	mint

hookworms	radish
hookworms	lettuce
hookworms	celery
hookworms	parsley
hookworms	cress
Hymenolepis nana	mint
Hymenolepis nana	radish
Hymenolepis nana	parsley
Hymenolepis nana	lettuce
Hymenolepis nana	cress
Hymenolepis nana	celery
Strongyloides stercoralis	mint
Strongyloides stercoralis	radish
Strongyloides stercoralis	lettuce
Strongyloides stercoralis	cress
Strongyloides stercoralis	celery
Strongyloides stercoralis	parsley
Toxoplasma gondii	mint
Toxoplasma gondii	radish
Toxoplasma gondii	lettuce
Toxoplasma gondii	cress
Toxoplasma gondii	celery
Toxoplasma gondii	parsley
Trichuris trichiura	mint
Trichuris trichiura	radish
Trichuris trichiura	parsley
Trichuris trichiura	cress
Trichuris trichiura	lettuce
Trichuris trichiura	celery
Cryptosporidium spp.	plum
Cryptosporidium spp.	orange
Cryptosporidium spp.	watermelon
Cryptosporidium spp.	melon
Cryptosporidium spp.	nectarine
Cryptosporidium spp.	pear
Cryptosporidium spp.	peach
Cryptosporidium spp.	grape
Cryptosporidium spp.	mint
Cryptosporidium spp.	potato
Cryptosporidium spp.	eggplant
Cryptosporidium spp.	spinach
Cryptosporidium spp.	lettuce
Cryptosporidium spp.	apple
Cryptosporidium spp.	celery
Cryptosporidium spp.	beetroot
Cryptosporidium spp.	chive
Cryptosporidium spp.	carrot
Cryptosporidium spp.	turmeric
Cryptosporidium spp.	endive
Cryptosporidium spp.	cucumber
Cryptosporidium spp.	bell pepper
Cryptosporidium spp.	cabbage
Cryptosporidium spp.	parsley
Cryptosporidium spp.	tomato
Cryptosporidium spp.	strawberry
Cryptosporidium spp.	dandelion
Giardia spp.	plum
Giardia spp.	orange

Giardia spp.	watermelon
Giardia spp.	melon
Giardia spp.	nectarine
Giardia spp.	pear
Giardia spp.	peach
Giardia spp.	grape
Giardia spp.	mint
Giardia spp.	potato
Giardia spp.	eggplant
Giardia spp.	spinach
Giardia spp.	lettuce
Giardia spp.	apple
Giardia spp.	strawberry
Giardia spp.	celery
Giardia spp.	beetroot
Giardia spp.	chive
Giardia spp.	carrot
Giardia spp.	turmeric
Giardia spp.	dandelion
Giardia spp.	endive
Giardia spp.	cucumber
Giardia spp.	bell pepper
Giardia spp.	cabbage
Giardia spp.	parsley
Giardia spp.	tomato
Ancylostoma spp.	savory
Ancylostoma spp.	spinach
Ancylostoma spp.	tomato
Ancylostoma spp.	parsley
Ancylostoma spp.	dill
Ancylostoma spp.	radish
Ancylostoma spp.	pumpkin
Ancylostoma spp.	carrot
Ancylostoma spp.	cucumber
Ancylostoma spp.	coriander
Ancylostoma spp.	mint
Ancylostoma spp.	basil
Ancylostoma spp.	cabbage
Ancylostoma spp.	leek
Ancylostoma spp.	lettuce
Ascaris lumbricoides	savory
Ascaris lumbricoides	spinach
Ascaris lumbricoides	tomato
Ascaris lumbricoides	dill
Ascaris lumbricoides	radish
Ascaris lumbricoides	pumpkin
Ascaris lumbricoides	mint
Ascaris lumbricoides	coriander
Ascaris lumbricoides	cabbage
Ascaris lumbricoides	cucumber
Ascaris lumbricoides	carrot
Ascaris lumbricoides	parsley
Ascaris lumbricoides	leek
Ascaris lumbricoides	basil
Ascaris lumbricoides	lettuce
Ascaris lumbricoides	savory
Ascaris lumbricoides	mint
Ascaris lumbricoides	basil

Ascaris lumbricoides	coriander
Ascaris lumbricoides	parsley
Ascaris lumbricoides	cucumber
Ascaris lumbricoides	leek
Ascaris lumbricoides	dill
Ascaris lumbricoides	spinach
Ascaris lumbricoides	radish
Ascaris lumbricoides	carrot
Ascaris lumbricoides	lettuce
Cryptosporidium parvum	savory
Cryptosporidium parvum	basil
Cryptosporidium parvum	spinach
Cryptosporidium parvum	tomato
Cryptosporidium parvum	dill
Cryptosporidium parvum	radish
Cryptosporidium parvum	pumpkin
Cryptosporidium parvum	cucumber
Cryptosporidium parvum	mint
Cryptosporidium parvum	coriander
Cryptosporidium parvum	carrot
Cryptosporidium parvum	cabbage
Cryptosporidium parvum	parsley
Cryptosporidium parvum	leek
Cryptosporidium parvum	lettuce
Cryptosporidium parvum	coriander
Cryptosporidium parvum	savory
Cryptosporidium parvum	dill
Cryptosporidium parvum	carrot
Cryptosporidium parvum	basil
Cryptosporidium parvum	cucumber
Cryptosporidium parvum	parsley
Cryptosporidium parvum	lettuce
Cryptosporidium parvum	mint
Cryptosporidium parvum	leek
Cryptosporidium parvum	spinach
Cryptosporidium parvum	radish
Dicrocoelium dendriticum	savory
Dicrocoelium dendriticum	basil
Dicrocoelium dendriticum	parsley
Dicrocoelium dendriticum	coriander
Dicrocoelium dendriticum	spinach
Dicrocoelium dendriticum	dill
Dicrocoelium dendriticum	mint
Dicrocoelium dendriticum	pumpkin
Dicrocoelium dendriticum	tomato
Dicrocoelium dendriticum	carrot
Dicrocoelium dendriticum	radish
Dicrocoelium dendriticum	cucumber
Dicrocoelium dendriticum	cabbage
Dicrocoelium dendriticum	lettuce
Dicrocoelium dendriticum	leek
Entamoeba coli	spinach
Entamoeba coli	radish
Entamoeba coli	cabbage
Entamoeba coli	lettuce
Entamoeba coli	savory
Entamoeba coli	tomato
Entamoeba coli	carrot

Entamoeba coli	cucumber
Entamoeba coli	basil
Entamoeba coli	pumpkin
Entamoeba coli	mint
Entamoeba coli	leek
Entamoeba coli	coriander
Entamoeba coli	dill
Entamoeba coli	parsley
Entamoeba coli	coriander
Entamoeba coli	savory
Entamoeba coli	cucumber
Entamoeba coli	spinach
Entamoeba coli	dill
Entamoeba coli	radish
Entamoeba coli	carrot
Entamoeba coli	parsley
Entamoeba coli	mint
Entamoeba coli	basil
Entamoeba coli	leek
Entamoeba coli	lettuce
Fasciola hepatica	dill
Fasciola hepatica	savory
Fasciola hepatica	coriander
Fasciola hepatica	radish
Fasciola hepatica	parsley
Fasciola hepatica	mint
Fasciola hepatica	basil
Fasciola hepatica	cabbage
Fasciola hepatica	spinach
Fasciola hepatica	pumpkin
Fasciola hepatica	tomato
Fasciola hepatica	carrot
Fasciola hepatica	lettuce
Fasciola hepatica	cucumber
Fasciola hepatica	leek
Fasciola hepatica	coriander
Fasciola hepatica	savory
Fasciola hepatica	spinach
Fasciola hepatica	radish
Fasciola hepatica	lettuce
Fasciola hepatica	parsley
Fasciola hepatica	mint
Fasciola hepatica	dill
Fasciola hepatica	basil
Fasciola hepatica	cucumber
Fasciola hepatica	carrot
Fasciola hepatica	leek
Giardia lamblia	savory
Giardia lamblia	dill
Giardia lamblia	pumpkin
Giardia lamblia	cabbage
Giardia lamblia	tomato
Giardia lamblia	mint

Giardia lamblia	spinach
Giardia lamblia	cucumber
Giardia lamblia	carrot
Giardia lamblia	radish
Giardia lamblia	basil
Giardia lamblia	coriander
Giardia lamblia	parsley
Giardia lamblia	leek
Giardia lamblia	lettuce
Giardia lamblia	coriander
Giardia lamblia	savory
Giardia lamblia	leek
Giardia lamblia	dill
Giardia lamblia	radish
Giardia lamblia	parsley
Giardia lamblia	mint
Giardia lamblia	basil
Giardia lamblia	spinach
Giardia lamblia	lettuce
Giardia lamblia	carrot
Giardia lamblia	cucumber
Hymenolepis nana	spinach
Hymenolepis nana	tomato
Hymenolepis nana	dill
Hymenolepis nana	radish
Hymenolepis nana	pumpkin
Hymenolepis nana	carrot
Hymenolepis nana	savory
Hymenolepis nana	coriander
Hymenolepis nana	basil
Hymenolepis nana	parsley
Hymenolepis nana	cucumber
Hymenolepis nana	mint
Hymenolepis nana	cabbage
Hymenolepis nana	leek
Hymenolepis nana	lettuce
Hymenolepis nana	coriander
Hymenolepis nana	savory
Hymenolepis nana	cucumber
Hymenolepis nana	dill
Hymenolepis nana	radish
Hymenolepis nana	carrot
Hymenolepis nana	parsley
Hymenolepis nana	basil
Hymenolepis nana	spinach
Hymenolepis nana	mint
Hymenolepis nana	leek
Hymenolepis nana	lettuce
Strongyloides stercoralis	savory
Strongyloides stercoralis	basil
Strongyloides stercoralis	spinach
Strongyloides stercoralis	tomato
Strongyloides stercoralis	parsley

Strongyloides stercoralis	dill
Strongyloides stercoralis	radish
Strongyloides stercoralis	leek
Strongyloides stercoralis	mint
Strongyloides stercoralis	lettuce
Strongyloides stercoralis	coriander
Strongyloides stercoralis	pumpkin
Strongyloides stercoralis	cucumber
Strongyloides stercoralis	carrot
Strongyloides stercoralis	cabbage
Strongyloides stercoralis	savory
Strongyloides stercoralis	radish
Strongyloides stercoralis	carrot
Strongyloides stercoralis	cucumber
Strongyloides stercoralis	dill
Strongyloides stercoralis	spinach
Strongyloides stercoralis	leek
Strongyloides stercoralis	mint
Strongyloides stercoralis	coriander
Strongyloides stercoralis	lettuce
Strongyloides stercoralis	basil
Strongyloides stercoralis	parsley
Taenia spp.	cucumber
Taenia spp.	cabbage
Taenia spp.	spinach
Taenia spp.	dill
Taenia spp.	tomato
Taenia spp.	mint
Taenia spp.	carrot
Taenia spp.	savory
Taenia spp.	pumpkin
Taenia spp.	radish
Taenia spp.	parsley
Taenia spp.	coriander
Taenia spp.	basil
Taenia spp.	leek
Taenia spp.	lettuce
Taenia spp.	cucumber
Taenia spp.	mint
Taenia spp.	coriander
Taenia spp.	radish
Taenia spp.	savory
Taenia spp.	carrot
Taenia spp.	dill
Taenia spp.	parsley
Taenia spp.	basil
Taenia spp.	spinach
Taenia spp.	lettuce
Taenia spp.	leek
Toxocara canis	savory
Toxocara canis	radish

Toxocara canis	coriander
Toxocara canis	dill
Toxocara canis	cabbage
Toxocara canis	pumpkin
Toxocara canis	mint
Toxocara canis	basil
Toxocara canis	spinach
Toxocara canis	tomato
Toxocara canis	parsley
Toxocara canis	carrot
Toxocara canis	cucumber
Toxocara canis	lettuce
Toxocara canis	leek
Toxocara canis	coriander
Toxocara canis	dill
Toxocara canis	carrot
Toxocara canis	parsley
Toxocara canis	mint
Toxocara canis	radish
Toxocara canis	basil
Toxocara canis	savory
Toxocara canis	cucumber
Toxocara canis	spinach
Toxocara canis	leek
Toxocara canis	lettuce
Toxocara cati	dill
Toxocara cati	savory
Toxocara cati	basil
Toxocara cati	spinach
Toxocara cati	radish
Toxocara cati	coriander
Toxocara cati	cabbage
Toxocara cati	pumpkin
Toxocara cati	mint
Toxocara cati	parsley
Toxocara cati	tomato
Toxocara cati	cucumber
Toxocara cati	carrot
Toxocara cati	lettuce
Toxocara cati	leek
Toxocara cati	coriander
Toxocara cati	savory
Toxocara cati	spinach
Toxocara cati	dill
Toxocara cati	radish
Toxocara cati	carrot
Toxocara cati	parsley
Toxocara cati	mint
Toxocara cati	cucumber
Toxocara cati	basil
Toxocara cati	lettuce

Toxocara cati	leek
Entamoeba coli	lettuce
Entamoeba coli	dill
Entamoeba coli	pepper
Entamoeba coli	cress
Entamoeba coli	radish
Entamoeba coli	tomato
Entamoeba coli	parsley
Entamoeba coli	coriander
Entamoeba coli	carrot
Entamoeba coli	cucumber
Entamoeba histolytica	cress
Entamoeba histolytica	lettuce
Entamoeba histolytica	tomato
Entamoeba histolytica	carrot
Entamoeba histolytica	parsley
Entamoeba histolytica	dill
Entamoeba histolytica	radish
Entamoeba histolytica	coriander
Entamoeba histolytica	cucumber
Entamoeba histolytica	pepper
Giardia lamblia	cress
Giardia lamblia	lettuce
Giardia lamblia	dill
Giardia lamblia	radish
Giardia lamblia	tomato
Giardia lamblia	parsley
Giardia lamblia	coriander
Giardia lamblia	cucumber
Giardia lamblia	pepper
Giardia lamblia	carrot
Cryptosporidium spp.	cabbage
Cryptosporidium spp.	lettuce
Cryptosporidium spp.	lettuce
Giardia spp.	cabbage
Giardia spp.	lettuce
Giardia spp.	lettuce
Ascaris lumbricoides	multiple vegetables
Entamoeba histolytica	multiple vegetables
hookworms	multiple vegetables
Hymenolepis nana	multiple vegetables
Strongyloides stercoralis	multiple vegetables
Trichomonas hominis	multiple vegetables
Trichuris trichiura	multiple vegetables
Enterocytozoom spp.	sprouts
Enterocytozoom spp.	ready-to-eat salad
Enterocytozoom spp.	fruits
Echinococcus granulosus	multiple vegetables
Hydatigera taeniaeformis	multiple vegetables
Hydatigera taeniaeformis	multiple vegetables

Taenia crassiceps	multiple vegetables
Taenia hydatigena	multiple vegetables
Taenia hydatigena	multiple vegetables
Taenia polyacantha	multiple vegetables
Taenia saginata	multiple vegetables
Taenia spp.	multiple vegetables
tapeworms	multiple vegetables
tapeworms	multiple vegetables
Trichuris ovis	multiple vegetables
Ascaris lumbricoides	fruits
Ascaris lumbricoides	ready-to-eat salad
Ascaris lumbricoides	roots
hookworms	fruits
hookworms	ready-to-eat salad
hookworms	roots
Trichuris trichiura	fruits
Trichuris trichiura	roots
Trichuris trichiura	ready-to-eat salad
Cryptosporidium parvum	potato
Cryptosporidium parvum	spinach
Cryptosporidium parvum	wild fruits
Cryptosporidium parvum	perilla
Cryptosporidium parvum	cherry tomato
Cryptosporidium parvum	carrot
Cryptosporidium parvum	blueberry
Cryptosporidium parvum	cabbage
Ascaris spp.	pumpkin
Ascaris spp.	pumpkin
Ascaris spp.	leek
Ascaris spp.	broccoli
Ascaris spp.	sprouts
Ascaris spp.	cauliflower
Ascaris spp.	vinegar
Ascaris spp.	lettuce
Ascaris spp.	anise
Ascaris spp.	anise
Ascaris spp.	celery
Ascaris spp.	broccoli
Ascaris spp.	sprouts
Ascaris spp.	onion
Ascaris spp.	cauliflower
Ascaris spp.	turnip
Ascaris spp.	turnip
Ascaris spp.	string bean
Ascaris spp.	string bean
Ascaris spp.	vinegar
Ascaris spp.	pumpkin
Ascaris spp.	lettuce
Ascaris spp.	cabbage
Ascaris spp.	potato
Ascaris spp.	potato

Ascaris spp.	strawberry
Ascaris spp.	cabbage
Ascaris spp.	parsley
Ascaris spp.	celery
Ascaris spp.	carrot
Ascaris spp.	parsley
Ascaris spp.	pumpkin
Ascaris spp.	carrot
Ascaris spp.	strawberry
Ascaris spp.	rhubarb
Ascaris spp.	beetroot
Ascaris spp.	rhubarb
Ascaris spp.	beetroot
Ascaris spp.	onion
Ascaris spp.	leek
Toxocara spp.	pumpkin
Toxocara spp.	pumpkin
Toxocara spp.	leek
Toxocara spp.	broccoli
Toxocara spp.	sprouts
Toxocara spp.	cauliflower
Toxocara spp.	vinegar
Toxocara spp.	lettuce
Toxocara spp.	anise
Toxocara spp.	anise
Toxocara spp.	celery
Toxocara spp.	broccoli
Toxocara spp.	onion
Toxocara spp.	cauliflower
Toxocara spp.	turnip
Toxocara spp.	turnip
Toxocara spp.	string bean
Toxocara spp.	string bean
Toxocara spp.	vinegar
Toxocara spp.	pumpkin
Toxocara spp.	lettuce
Toxocara spp.	cabbage
Toxocara spp.	potato
Toxocara spp.	strawberry
Toxocara spp.	cabbage
Toxocara spp.	rhubarb
Toxocara spp.	parsley
Toxocara spp.	parsley
Toxocara spp.	beetroot
Toxocara spp.	strawberry
Toxocara spp.	sprouts
Toxocara spp.	potato
Toxocara spp.	celery
Toxocara spp.	beetroot
Toxocara spp.	rhubarb
Toxocara spp.	pumpkin

Toxocara spp.	carrot
Toxocara spp.	leek
Toxocara spp.	onion
Toxocara spp.	carrot
Trichuris spp.	pumpkin
Trichuris spp.	pumpkin
Trichuris spp.	leek
Trichuris spp.	broccoli
Trichuris spp.	sprouts
Trichuris spp.	cauliflower
Trichuris spp.	vinegar
Trichuris spp.	lettuce
Trichuris spp.	anise
Trichuris spp.	anise
Trichuris spp.	celery
Trichuris spp.	broccoli
Trichuris spp.	sprouts
Trichuris spp.	onion
Trichuris spp.	cauliflower
Trichuris spp.	turnip
Trichuris spp.	turnip
Trichuris spp.	string bean
Trichuris spp.	string bean
Trichuris spp.	vinegar
Trichuris spp.	pumpkin
Trichuris spp.	lettuce
Trichuris spp.	cabbage
Trichuris spp.	potato
Trichuris spp.	potato
Trichuris spp.	strawberry
Trichuris spp.	cabbage
Trichuris spp.	parsley
Trichuris spp.	celery
Trichuris spp.	carrot
Trichuris spp.	parsley
Trichuris spp.	pumpkin
Trichuris spp.	onion
Trichuris spp.	carrot
Trichuris spp.	strawberry
Trichuris spp.	leek
Trichuris spp.	rhubarb
Trichuris spp.	beetroot
Trichuris spp.	rhubarb
Trichuris spp.	beetroot
Ascaris lumbricoides	arugula
Ascaris lumbricoides	cabbage
Ascaris lumbricoides	chive
Ascaris lumbricoides	tomato
Ascaris lumbricoides	cucumber
Ascaris lumbricoides	bell pepper

Ascaris lumbricoides	carrot
Ascaris lumbricoides	parsley
Ascaris lumbricoides	lettuce
Taenia spp.	arugula
Taenia spp.	cabbage
Taenia spp.	tomato
Taenia spp.	bell pepper
Taenia spp.	lettuce
Taenia spp.	chive
Taenia spp.	cucumber
Taenia spp.	carrot
Taenia spp.	parsley
Toxocara spp.	arugula
Toxocara spp.	cabbage
Toxocara spp.	chive
Toxocara spp.	tomato
Toxocara spp.	cucumber
Toxocara spp.	bell pepper
Toxocara spp.	carrot
Toxocara spp.	parsley
Toxocara spp.	lettuce
Balantidium coli	chive
Balantidium coli	carrot
Balantidium coli	lettuce
Balantidium coli	cabbage
Balantidium coli	tomato
Cryptosporidium spp.	tomato
Cryptosporidium spp.	carrot
Cryptosporidium spp.	chive
Cryptosporidium spp.	cabbage
Cryptosporidium spp.	lettuce
Fasciola spp.	chive
Fasciola spp.	carrot
Fasciola spp.	cabbage
Fasciola spp.	tomato
Fasciola spp.	lettuce
Strongyloides stercoralis	tomato
Strongyloides stercoralis	cabbage
Strongyloides stercoralis	carrot
Strongyloides stercoralis	lettuce
Strongyloides stercoralis	chive
Cryptosporidium spp.	chard
Cryptosporidium spp.	multiple vegetables
Cryptosporidium spp.	green cabbage
Cryptosporidium spp.	green leaves
Cryptosporidium spp.	lettuce
Cryptosporidium spp.	ready-to-eat salad
Cryptosporidium spp.	lettuce
Cryptosporidium spp.	arugula
Cryptosporidium spp.	spinach

Cyclospora spp.	chard
Cyclospora spp.	multiple vegetables
Cyclospora spp.	green cabbage
Cyclospora spp.	green leaves
Cyclospora spp.	lettuce
Cyclospora spp.	lettuce
Cyclospora spp.	ready-to-eat salad
Cyclospora spp.	spinach
Cyclospora spp.	arugula
Toxoplasma gondii	chard
Toxoplasma gondii	multiple vegetables
Toxoplasma gondii	green cabbage
Toxoplasma gondii	green leaves
Toxoplasma gondii	arugula
Toxoplasma gondii	lettuce
Toxoplasma gondii	ready-to-eat salad
Toxoplasma gondii	lettuce
Toxoplasma gondii	spinach
Toxoplasma gondii	strawberry
Toxoplasma gondii	radish
Toxoplasma gondii	lettuce
Toxoplasma gondii	carrot
Toxoplasma gondii	strawberry
Toxoplasma gondii	radish
Toxoplasma gondii	carrot
Toxoplasma gondii	lettuce
Echinococcus multilocularis	wild fruits
Echinococcus multilocularis	raspberry
Echinococcus multilocularis	multiple vegetables
Cryptosporidium parvum	potato
Cryptosporidium parvum	pepper
Cryptosporidium parvum	mustard
Cryptosporidium parvum	watermelon
Cryptosporidium parvum	cabbage
Cryptosporidium parvum	spinach
Cryptosporidium parvum	schizonepta
Cryptosporidium parvum	endive
Cryptosporidium parvum	anise
Cryptosporidium parvum	chrysanthemum
Cryptosporidium parvum	spinach
Cryptosporidium parvum	bean
Cryptosporidium parvum	cucumber
Cryptosporidium parvum	lettuce
Cryptosporidium parvum	cabbage
Cryptosporidium parvum	cabbage
Cryptosporidium parvum	celery
Cryptosporidium parvum	chive
Cryptosporidium parvum	coriander
Cryptosporidium parvum	lettuce
Cryptosporidium parvum	chive

Cyclospora cayetanensis	potato
Cyclospora cayetanensis	pepper
Cyclospora cayetanensis	mustard
Cyclospora cayetanensis	watermelon
Cyclospora cayetanensis	cabbage
Cyclospora cayetanensis	spinach
Cyclospora cayetanensis	schizonepta
Cyclospora cayetanensis	endive
Cyclospora cayetanensis	anise
Cyclospora cayetanensis	chrysanthemum
Cyclospora cayetanensis	spinach
Cyclospora cayetanensis	bean
Cyclospora cayetanensis	cucumber
Cyclospora cayetanensis	cabbage
Cyclospora cayetanensis	cabbage
Cyclospora cayetanensis	celery
Cyclospora cayetanensis	chive
Cyclospora cayetanensis	chive
Cyclospora cayetanensis	coriander
Cyclospora cayetanensis	lettuce
Cyclospora cayetanensis	lettuce
Enterocytozoon bieneusi	pepper
Enterocytozoon bieneusi	mustard
Enterocytozoon bieneusi	cabbage
Enterocytozoon bieneusi	spinach
Enterocytozoon bieneusi	schizonepta
Enterocytozoon bieneusi	chrysanthemum
Enterocytozoon bieneusi	cabbage
Enterocytozoon bieneusi	potato
Enterocytozoon bieneusi	watermelon
Enterocytozoon bieneusi	endive
Enterocytozoon bieneusi	anise
Enterocytozoon bieneusi	cucumber
Enterocytozoon bieneusi	lettuce
Enterocytozoon bieneusi	cabbage
Enterocytozoon bieneusi	celery
Enterocytozoon bieneusi	coriander
Enterocytozoon bieneusi	spinach
Enterocytozoon bieneusi	bean
Enterocytozoon bieneusi	chive
Enterocytozoon bieneusi	chive
Enterocytozoon bieneusi	lettuce
Ascaris spp.	chive
Ascaris spp.	arugula
Ascaris spp.	lettuce
Ascaris spp.	lettuce
Ascaris spp.	chive
Ascaris spp.	chive
Ascaris spp.	arugula
Ascaris spp.	lettuce
Ascaris spp.	arugula

Endolimax nana	lettuce
Endolimax nana	arugula
Endolimax nana	chive
Endolimax nana	lettuce
Endolimax nana	lettuce
Endolimax nana	chive
Endolimax nana	chive
Endolimax nana	arugula
Endolimax nana	arugula
Entamoeba coli	arugula
Entamoeba coli	lettuce
Entamoeba coli	chive
Entamoeba coli	arugula
Entamoeba coli	chive
Entamoeba coli	arugula
Entamoeba coli	lettuce
Entamoeba coli	chive
Entamoeba coli	lettuce
Entamoeba histolytica/dispar	lettuce
Entamoeba histolytica/dispar	chive
Entamoeba histolytica/dispar	arugula
Entamoeba histolytica/dispar	lettuce
Entamoeba histolytica/dispar	lettuce
Entamoeba histolytica/dispar	chive
Entamoeba histolytica/dispar	chive
Entamoeba histolytica/dispar	arugula
Entamoeba histolytica/dispar	arugula
Fasciola hepatica	lettuce
Fasciola hepatica	chive
Fasciola hepatica	arugula
Fasciola hepatica	lettuce
Fasciola hepatica	chive
Fasciola hepatica	chive
Fasciola hepatica	arugula
Fasciola hepatica	lettuce
Fasciola hepatica	arugula
Giardia spp.	lettuce
Giardia spp.	chive
Giardia spp.	arugula
Giardia spp.	lettuce
Giardia spp.	lettuce
Giardia spp.	chive
Giardia spp.	chive
Giardia spp.	arugula
Giardia spp.	arugula
hookworms	chive
hookworms	arugula
hookworms	lettuce
hookworms	lettuce
hookworms	chive
hookworms	arugula

hookworms	arugula
hookworms	chive
hookworms	lettuce
Hymenolepis nana	chive
Hymenolepis nana	lettuce
Hymenolepis nana	arugula
Hymenolepis nana	lettuce
Hymenolepis nana	chive
Hymenolepis nana	chive
Hymenolepis nana	arugula
Hymenolepis nana	arugula
Hymenolepis nana	lettuce
nematode larva	arugula
nematode larva	lettuce
nematode larva	chive
nematode larva	chive
nematode larva	arugula
nematode larva	arugula
nematode larva	lettuce
nematode larva	lettuce
nematode larva	chive
Taenia/ Echinococcus	lettuce
Taenia/ Echinococcus	chive
Taenia/ Echinococcus	arugula
Taenia/ Echinococcus	lettuce
Taenia/ Echinococcus	lettuce
Taenia/ Echinococcus	chive
Taenia/ Echinococcus	arugula
Taenia/ Echinococcus	arugula
Taenia/ Echinococcus	chive
Cryptosporidium spp.	strawberry
Cryptosporidium spp.	celery
Cryptosporidium spp.	coriander
Cryptosporidium spp.	blackberry
Cryptosporidium spp.	lettuce
Cyclospora cayetanensis	blackberry
Cyclospora cayetanensis	strawberry
Cyclospora cayetanensis	celery
Cyclospora cayetanensis	coriander
Cyclospora cayetanensis	lettuce
Ascaris spp.	lettuce
Ascaris spp.	leek
Ascaris spp.	tarragon
Ascaris spp.	radish
Ascaris spp.	mint
Ascaris spp.	cress
Ascaris spp.	leek
Ascaris spp.	chive
Ascaris spp.	basil
Ascaris spp.	radish

Ascaris spp.	parsley
Ascaris spp.	lettuce
Blastocystis spp.	mint
Blastocystis spp.	basil
Blastocystis spp.	mint
Blastocystis spp.	leek
Blastocystis spp.	lettuce
Blastocystis spp.	basil
Blastocystis spp.	radish
Entamoeba histolytica	cress
Entamoeba histolytica	basil
Entamoeba spp.	mint
Entamoeba spp.	lettuce
Entamoeba spp.	radish
Giardia spp.	mint
Giardia spp.	chive
Giardia spp.	lettuce
Giardia spp.	basil
Giardia spp.	radish
hookworms	lettuce
hookworms	leek
hookworms	radish
hookworms	basil
hookworms	mint
hookworms	cress
hookworms	leek
hookworms	chive
hookworms	basil
hookworms	radish
hookworms	parsley
hookworms	lettuce
Hymenolepis nana	leek
Hymenolepis nana	tarragon
Hymenolepis nana	mint
Hymenolepis nana	leek
Hymenolepis nana	chive
Hymenolepis nana	radish
Hymenolepis nana	parsley
Hymenolepis nana	lettuce
nematode larva	lettuce
nematode larva	lettuce
nematode larva	chive
nematode larva	chive
nematode larva	basil
nematode larva	parsley
nematode larva	mint
nematode larva	mint
nematode larva	cress
nematode larva	leek
nematode larva	chive

nematode larva	chive
nematode larva	coriander
nematode larva	tarragon
nematode larva	basil
nematode larva	basil
nematode larva	radish
nematode larva	lettuce
nematode larva	lettuce
nematode larva	leek
nematode larva	radish
Physaloptera spp.	basil
Physaloptera spp.	radish
roundworms	multiple vegetables
Ascaris lumbricoides	multiple vegetables
Entamoeba histolytica	multiple vegetables
hookworms	multiple vegetables
Iodamoeba spp.	multiple vegetables
Trichuris trichiura	multiple vegetables
Cryptosporidium spp.	coriander
Cryptosporidium spp.	celery
Cryptosporidium spp.	chive
Cryptosporidium spp.	centella
Cryptosporidium spp.	cabbage
Cryptosporidium spp.	carrot
Cryptosporidium spp.	tomato
Cryptosporidium spp.	purslane
Cryptosporidium spp.	spinach
Cryptosporidium spp.	jute mallow
Cryptosporidium spp.	pumpkin
Cryptosporidium spp.	lettuce
Toxoplasma gondii	arugula
Toxoplasma gondii	endive
Toxoplasma gondii	parsley
Toxoplasma gondii	lettuce
Ascaris lumbricoides	pepper
Ascaris lumbricoides	lettuce
Ascaris lumbricoides	cabbage
Ascaris lumbricoides	cabbage
Ascaris lumbricoides	beetroot
Ascaris lumbricoides	cress
Ascaris lumbricoides	radish
Ascaris lumbricoides	bell pepper
Ascaris lumbricoides	chive
Ascaris lumbricoides	carrot
Ascaris lumbricoides	tomato
Entamoeba histolytica/dispar	pepper
Entamoeba histolytica/dispar	cabbage
Entamoeba histolytica/dispar	radish
Entamoeba histolytica/dispar	beetroot
Entamoeba histolytica/dispar	cress

Entamoeba histolytica/dispar	bell pepper
Entamoeba histolytica/dispar	tomato
Entamoeba histolytica/dispar	lettuce
Entamoeba histolytica/dispar	cabbage
Entamoeba histolytica/dispar	carrot
Entamoeba histolytica/dispar	chive
Giardia lamblia	pepper
Giardia lamblia	cabbage
Giardia lamblia	cabbage
Giardia lamblia	beetroot
Giardia lamblia	lettuce
Giardia lamblia	radish
Giardia lamblia	bell pepper
Giardia lamblia	chive
Giardia lamblia	tomato
Giardia lamblia	carrot
Giardia lamblia	cress
hookworms	pepper
hookworms	lettuce
hookworms	cabbage
hookworms	cabbage
hookworms	radish
hookworms	bell pepper
hookworms	chive
hookworms	tomato
hookworms	carrot
hookworms	beetroot
hookworms	cress
Strongyloides stercoralis	pepper
Strongyloides stercoralis	lettuce
Strongyloides stercoralis	cabbage
Strongyloides stercoralis	cabbage
Strongyloides stercoralis	beetroot
Strongyloides stercoralis	radish
Strongyloides stercoralis	bell pepper
Strongyloides stercoralis	chive
Strongyloides stercoralis	tomato
Strongyloides stercoralis	carrot
Strongyloides stercoralis	cress
Trichuris trichiura	pepper
Trichuris trichiura	lettuce
Trichuris trichiura	cabbage
Trichuris trichiura	beetroot
Trichuris trichiura	cress
Trichuris trichiura	radish
Trichuris trichiura	bell pepper
Trichuris trichiura	chive
Trichuris trichiura	tomato
Trichuris trichiura	carrot
Trichuris trichiura	cabbage

Strongyloides stercoralis	coriander
Ascaris lumbricoides	coriander
Ascaris lumbricoides	celery
Ascaris lumbricoides	parsley
Ascaris lumbricoides	spinach
Ascaris lumbricoides	lettuce
Ascaris lumbricoides	mint
hookworms	coriander
hookworms	celery
hookworms	parsley
hookworms	spinach
hookworms	lettuce
hookworms	mint
Trichuris trichiura	celery
Trichuris trichiura	parsley
Trichuris trichiura	lettuce
Trichuris trichiura	coriander
Trichuris trichiura	spinach
Trichuris trichiura	mint
Ascaris lumbricoides	lettuce
Ascaris lumbricoides	lettuce
Ascaris lumbricoides	lettuce
Ascaris lumbricoides	lettuce
Cyclospora spp.	lettuce
Cyclospora spp.	lettuce
Cyclospora spp.	lettuce
Cyclospora spp.	lettuce
Endolimax nana	lettuce
Endolimax nana	lettuce
Endolimax nana	lettuce
Endolimax nana	lettuce
Entamoeba coli	lettuce
Entamoeba coli	lettuce
Entamoeba coli	lettuce
Entamoeba coli	lettuce
Entamoeba histolytica	lettuce
Entamoeba histolytica	lettuce
Entamoeba histolytica	lettuce
Entamoeba histolytica	lettuce
Giardia lamblia	lettuce
Giardia lamblia	lettuce
Giardia lamblia	lettuce
Giardia lamblia	lettuce
hookworms	lettuce
hookworms	lettuce
hookworms	lettuce
hookworms	lettuce
hookworms	lettuce
hookworms	lettuce
hookworms	lettuce
hookworms	lettuce

hookworms	lettuce
hookworms	lettuce
hookworms	lettuce
hookworms	lettuce
Hymenolepis nana	lettuce
Hymenolepis nana	lettuce
Hymenolepis nana	lettuce
Hymenolepis nana	lettuce
Iodamoeba butschlii	lettuce
Iodamoeba butschlii	lettuce
Iodamoeba butschlii	lettuce
Iodamoeba butschlii	lettuce
Strongyloides stercoralis	lettuce
Strongyloides stercoralis	lettuce
Strongyloides stercoralis	lettuce
Strongyloides stercoralis	lettuce
Strongyloides stercoralis	lettuce
Strongyloides stercoralis	lettuce
Strongyloides stercoralis	lettuce
Strongyloides stercoralis	lettuce
Ascaris spp.	cress
Ascaris spp.	spinach
Ascaris spp.	parsley
Ascaris spp.	lettuce
Ascaris spp.	strawberry
Entamoeba histolytica	spinach
Entamoeba histolytica	cress
Entamoeba histolytica	lettuce
Entamoeba histolytica	parsley
Entamoeba histolytica	strawberry
Enterobius vermicularis	cress
Enterobius vermicularis	spinach
Enterobius vermicularis	lettuce
Enterobius vermicularis	parsley
Enterobius vermicularis	strawberry
Giardia spp.	spinach
Giardia spp.	cress
Giardia spp.	parsley
Giardia spp.	lettuce
Giardia spp.	strawberry
Ascaris suum	celosia
Entamoeba histolytica/dispar	mulukhiyah
Strongyloides stercoralis	lettuce
Strongyloides stercoralis	pumpkin
Strongyloides stercoralis	mulukhiyah
Trichostrongylus spp.	celosia
Trichostrongylus spp.	mulukhiyah
Ascaris lumbricoides	multiple vegetables
Dicrocoelium spp.	multiple vegetables
Enterobius vermicularis	multiple vegetables

Fasciola spp.	multiple vegetables
Giardia lamblia	multiple vegetables
Hymenolepis nana	multiple vegetables
nematode	multiple vegetables
Taenia spp.	multiple vegetables
Toxascaris leonina	multiple vegetables
Toxocara canis	multiple vegetables
Toxocara cati	multiple vegetables
Trichostrongylus spp.	multiple vegetables
Trichuris trichiura	multiple vegetables
Ascaris lumbricoides	lettuce
Echinococcus granulosus	lettuce
Entamoeba histolytica	beetroot
Entamoeba histolytica	pear
Entamoeba histolytica	celery
Entamoeba histolytica	carrot
Entamoeba histolytica	apple
Entamoeba histolytica	tomato
Entamoeba histolytica	grape
Entamoeba histolytica	cucumber
Entamoeba histolytica	radish
Entamoeba histolytica	lettuce
Giardia lamblia	beetroot
Giardia lamblia	carrot
Giardia lamblia	apple
Giardia lamblia	celery
Giardia lamblia	radish
Giardia lamblia	pear
Giardia lamblia	tomato
Hymenolepis nana	grape
Hymenolepis nana	cucumber
Entamoeba coli	lettuce
nematode larva	lettuce
Cryptosporidium spp.	oregano
Cryptosporidium spp.	leek
Cryptosporidium spp.	endive
Cryptosporidium spp.	turnip
Cryptosporidium spp.	pepper
Cryptosporidium spp.	mint
Cryptosporidium spp.	garlic
Cryptosporidium spp.	parsley
Cryptosporidium spp.	celery
Cryptosporidium spp.	beetroot
Cryptosporidium spp.	tomato
Cryptosporidium spp.	carrot
Cryptosporidium spp.	chive
Cryptosporidium spp.	radish
Cryptosporidium spp.	cabbage
Cryptosporidium spp.	spinach
Cryptosporidium spp.	chard

Cryptosporidium spp.	lettuce
Cryptosporidium spp.	cress
Giardia spp.	oregano
Giardia spp.	leek
Giardia spp.	endive
Giardia spp.	turnip
Giardia spp.	pepper
Giardia spp.	mint
Giardia spp.	garlic
Giardia spp.	parsley
Giardia spp.	celery
Giardia spp.	beetroot
Giardia spp.	tomato
Giardia spp.	cress
Giardia spp.	chive
Giardia spp.	radish
Giardia spp.	spinach
Giardia spp.	chard
Giardia spp.	carrot
Giardia spp.	cabbage
Giardia spp.	lettuce
Acanthamoeba spp.	ready-to-eat salad
Endolimax nana	ready-to-eat salad
Entamoeba coli	ready-to-eat salad
Schistosoma mansoni	ready-to-eat salad
Toxoplasma gondii	ready-to-eat salad
Chilomatix spp.	lettuce
Dipylidium spp.	endive
Entamoeba spp.	endive
Entamoeba spp.	lettuce
hookworms	endive
nematode larva	chive
nematode larva	endive
nematode larva	lettuce
unsporulated oocyst	arugula
unsporulated oocyst	lettuce
Strongyloides spp.	lettuce
Toxoplasma gondii	lettuce
Toxoplasma gondii	lettuce
Toxoplasma gondii	lettuce
Trichuris spp.	lettuce
Ascaris lumbricoides	mint
Ascaris lumbricoides	centella
Ascaris lumbricoides	potato
Ascaris lumbricoides	leek
Ascaris lumbricoides	coriander
Ascaris lumbricoides	basil
Ascaris lumbricoides	cabbage
Ascaris lumbricoides	celery
Ascaris lumbricoides	lettuce

Ascaris lumbricoides	coriander
hookworms	potato
hookworms	basil
hookworms	lettuce
hookworms	cabbage
hookworms	mint
hookworms	leek
hookworms	celery
hookworms	coriander
hookworms	centella
hookworms	coriander
Strongyloides stercoralis	lettuce
Strongyloides stercoralis	potato
Strongyloides stercoralis	basil
Strongyloides stercoralis	cabbage
Strongyloides stercoralis	mint
Strongyloides stercoralis	coriander
Strongyloides stercoralis	coriander
Strongyloides stercoralis	centella
Strongyloides stercoralis	leek
Strongyloides stercoralis	celery
Toxocara spp.	lettuce
Toxocara spp.	coriander
Toxocara spp.	potato
Toxocara spp.	celery
Toxocara spp.	leek
Toxocara spp.	mint
Toxocara spp.	centella
Toxocara spp.	basil
Toxocara spp.	coriander
Toxocara spp.	cabbage
Trichuris trichiura	mint
Trichuris trichiura	lettuce
Trichuris trichiura	centella
Trichuris trichiura	coriander
Trichuris trichiura	celery
Trichuris trichiura	leek
Trichuris trichiura	potato
Trichuris trichiura	basil
Trichuris trichiura	cabbage
Trichuris trichiura	coriander
Ancylostoma duodenale	purslane
Ancylostoma duodenale	pumpkin
Ancylostoma duodenale	eggplant
Ancylostoma duodenale	onion
Ancylostoma duodenale	cabbage
Ancylostoma duodenale	tomato
Ascaris lumbricoides	purslane
Ascaris lumbricoides	eggplant
Entamoeba histolytica	purslane
Entamoeba histolytica	eggplant

Entamoeba histolytica	onion
Necator americanus	purslane
Necator americanus	onion
Necator americanus	cabbage
Strongyloides stercoralis	purslane
Strongyloides stercoralis	eggplant
Strongyloides stercoralis	onion
Strongyloides stercoralis	cabbage
Trichuris trichiura	cabbage
Trichuris trichiura	tomato
Cryptosporidium spp.	multiple vegetables
Emeria spp.	multiple vegetables
Entamoeba spp.	multiple vegetables
Enterobius vermicularis	multiple vegetables
Fasciola hepatica	multiple vegetables
Giardia lamblia	multiple vegetables
Hymenolepis nana	multiple vegetables
Toxoplasma gondii	multiple vegetables
Giardia lamblia	green cabbage
Giardia lamblia	arugula
Giardia lamblia	endive
Giardia lamblia	lettuce
Giardia lamblia	green cabbage
Giardia lamblia	arugula
Giardia lamblia	lettuce
Giardia lamblia	endive
roundworms	sprouts
roundworms	parsley
roundworms	dill
roundworms	raspberry
roundworms	sprouts
roundworms	ready-to-eat salad
roundworms	strawberry
roundworms	sprouts
roundworms	lettuce
Cryptosporidium spp.	sprouts
Cryptosporidium spp.	dill
Cryptosporidium spp.	parsley
Cryptosporidium spp.	raspberry
Cryptosporidium spp.	sprouts
Cryptosporidium spp.	ready-to-eat salad
Cryptosporidium spp.	strawberry
Cryptosporidium spp.	lettuce
Cryptosporidium spp.	sprouts
Cyclospora spp.	sprouts
Cyclospora spp.	dill
Cyclospora spp.	parsley
Cyclospora spp.	raspberry
Cyclospora spp.	ready-to-eat salad
Cyclospora spp.	lettuce
Cyclospora spp.	sprouts
Cyclospora spp.	strawberry
Giardia spp.	parsley
Giardia spp.	raspberry
Giardia spp.	sprouts
Giardia spp.	ready-to-eat salad

Giardia spp.	sprouts
Giardia spp.	dill
Giardia spp.	strawberry
Giardia spp.	lettuce
Giardia spp.	sprouts
Ascaris lumbricoides	lettuce
Ascaris lumbricoides	lettuce
Blastocystis hominis	lettuce
Blastocystis hominis	lettuce
Endolimax nana	lettuce
Endolimax nana	lettuce
Entamoeba coli	lettuce
Entamoeba coli	lettuce
Entamoeba histolytica	lettuce
Entamoeba histolytica	lettuce
Giardia spp.	lettuce
hookworms	lettuce
hookworms	lettuce
Strongyloides stercoralis	lettuce
Strongyloides stercoralis	lettuce
Taenia spp.	lettuce
Taenia spp.	lettuce
Trichuris trichiura	lettuce
Trichuris trichiura	lettuce
Cryptosporidium parvum	celery
Cryptosporidium spp.	cabbage
Cryptosporidium spp.	cabbage
Cryptosporidium spp.	cabbage
Cryptosporidium spp.	cabbage
Cryptosporidium spp.	leek
Ascaridia galli	pumpkin
Ascaridia galli	yam
Ascaridia galli	horseradish
Ascaridia galli	cucumber
Ascaridia galli	carrot
Ascaridia galli	tomato
Ascaridia galli	coriander
Ascaridia galli	lettuce
Ascaridia galli	endive
Ascaridia galli	basil
Ascaridia galli	centella
Ascaridia galli	turnip
Ascaridia galli	vietnamese balm
Ascaridia galli	mint
Ascaridia galli	potato
Ascaris spp.	horseradish
Ascaris spp.	cucumber
Ascaris spp.	centella
Ascaris spp.	pumpkin
Ascaris spp.	yam
Ascaris spp.	tomato
Ascaris spp.	endive
Ascaris spp.	carrot
Ascaris spp.	coriander

Ascaris spp.	turnip
Ascaris spp.	lettuce
Ascaris spp.	vietnamese balm
Ascaris spp.	basil
Ascaris spp.	mint
Ascaris spp.	potato
Taenia spp.	centella
Taenia spp.	pumpkin
Taenia spp.	yam
Taenia spp.	horseradish
Taenia spp.	turnip
Taenia spp.	cucumber
Taenia spp.	carrot
Taenia spp.	tomato
Taenia spp.	coriander
Taenia spp.	vietnamese balm
Taenia spp.	lettuce
Taenia spp.	endive
Taenia spp.	basil
Taenia spp.	mint
Taenia spp.	potato
Toxocara spp.	centella
Toxocara spp.	pumpkin
Toxocara spp.	yam
Toxocara spp.	horseradish
Toxocara spp.	cucumber
Toxocara spp.	carrot
Toxocara spp.	tomato
Toxocara spp.	lettuce
Toxocara spp.	endive
Toxocara spp.	coriander
Toxocara spp.	vietnamese balm
Toxocara spp.	basil
Toxocara spp.	turnip
Toxocara spp.	mint
Toxocara spp.	potato
Trichuris spp.	centella
Trichuris spp.	pumpkin
Trichuris spp.	yam
Trichuris spp.	horseradish
Trichuris spp.	cucumber
Trichuris spp.	carrot
Trichuris spp.	tomato
Trichuris spp.	vietnamese balm
Trichuris spp.	endive
Trichuris spp.	turnip
Trichuris spp.	coriander
Trichuris spp.	lettuce
Trichuris spp.	basil
Trichuris spp.	potato
Trichuris spp.	mint

Ascaris lumbricoides	multiple vegetables
Entamoeba coli	multiple vegetables
Enterobius vermicularis	multiple vegetables
hookworms	multiple vegetables
Hymenolepis nana	multiple vegetables
tapeworms	multiple vegetables
Trichuris trichiura	multiple vegetables
Cryptosporidium spp.	basil
Cryptosporidium spp.	leek
Cryptosporidium spp.	coriander
Cryptosporidium spp.	mint
Cryptosporidium spp.	chive
Cryptosporidium spp.	cress
Entamoeba coli	spinach
Entamoeba coli	lettuce
Entamoeba coli	cress
Entamoeba coli	chive
Entamoeba coli	parsley
Entamoeba coli	leek
Entamoeba coli	radish
Entamoeba histolytica/dispar	cress
Entamoeba histolytica/dispar	leek
Entamoeba histolytica/dispar	radish
Giardia lamblia	spinach
Giardia lamblia	mint
Giardia lamblia	leek
Giardia lamblia	basil
Giardia lamblia	parsley
Giardia lamblia	chive
Giardia lamblia	lettuce
Giardia lamblia	cress
Giardia lamblia	radish
Ascaris spp.	bell pepper
Ascaris spp.	tomato
Ascaris spp.	beetroot
Ascaris spp.	coriander
Ascaris spp.	cucumber
Ascaris spp.	radish
Ascaris spp.	carrot
Ascaris spp.	lettuce
Ascaris spp.	mint
Ascaris spp.	cabbage
Entamoeba spp.	mint
Entamoeba spp.	beetroot
Entamoeba spp.	carrot
Entamoeba spp.	coriander
Entamoeba spp.	cucumber
Entamoeba spp.	radish
Entamoeba spp.	tomato
Entamoeba spp.	lettuce
Entamoeba spp.	bell pepper
Entamoeba spp.	cabbage
Enterobius spp.	lettuce

Enterobius spp.	mint
Enterobius spp.	beetroot
Enterobius spp.	cucumber
Enterobius spp.	bell pepper
Enterobius spp.	radish
Enterobius spp.	cabbage
Enterobius spp.	carrot
Enterobius spp.	coriander
Enterobius spp.	tomato
Fasciola spp.	lettuce
Fasciola spp.	mint
Fasciola spp.	bell pepper
Fasciola spp.	radish
Fasciola spp.	beetroot
Fasciola spp.	carrot
Fasciola spp.	coriander
Fasciola spp.	cucumber
Fasciola spp.	cabbage
Fasciola spp.	tomato
Giardia spp.	coriander
Giardia spp.	cucumber
Giardia spp.	bell pepper
Giardia spp.	tomato
Giardia spp.	lettuce
Giardia spp.	beetroot
Giardia spp.	carrot
Giardia spp.	radish
Giardia spp.	cabbage
Giardia spp.	mint
hookworms	beetroot
hookworms	carrot
hookworms	coriander
hookworms	cucumber
hookworms	tomato
hookworms	bell pepper
hookworms	radish
hookworms	lettuce
hookworms	mint
hookworms	cabbage
Hymenolepis spp.	lettuce
Hymenolepis spp.	mint
Hymenolepis spp.	beetroot
Hymenolepis spp.	bell pepper
Hymenolepis spp.	carrot
Hymenolepis spp.	coriander
Hymenolepis spp.	cucumber
Hymenolepis spp.	radish
Hymenolepis spp.	cabbage
Hymenolepis spp.	tomato
Taenia spp.	bell pepper
Taenia spp.	tomato

Taenia spp.	lettuce
Taenia spp.	mint
Taenia spp.	beetroot
Taenia spp.	carrot
Taenia spp.	coriander
Taenia spp.	cucumber
Taenia spp.	radish
Taenia spp.	cabbage
Toxocara spp.	mint
Toxocara spp.	cucumber
Toxocara spp.	bell pepper
Toxocara spp.	radish
Toxocara spp.	tomato
Toxocara spp.	beetroot
Toxocara spp.	carrot
Toxocara spp.	coriander
Toxocara spp.	cabbage
Toxocara spp.	lettuce
Toxoplasma gondii	mint
Toxoplasma gondii	beetroot
Toxoplasma gondii	carrot
Toxoplasma gondii	cucumber
Toxoplasma gondii	bell pepper
Toxoplasma gondii	cabbage
Toxoplasma gondii	tomato
Toxoplasma gondii	lettuce
Toxoplasma gondii	coriander
Toxoplasma gondii	radish
Trichostrongylidae	lettuce
Trichostrongylidae	tomato
Trichostrongylidae	coriander
Trichostrongylidae	bell pepper
Trichostrongylidae	mint
Trichostrongylidae	beetroot
Trichostrongylidae	carrot
Trichostrongylidae	cucumber
Trichostrongylidae	radish
Trichostrongylidae	cabbage
Trichuris spp.	carrot
Trichuris spp.	coriander
Trichuris spp.	cucumber
Trichuris spp.	radish
Trichuris spp.	beetroot
Trichuris spp.	bell pepper
Trichuris spp.	tomato
Trichuris spp.	mint
Trichuris spp.	cabbage
Trichuris spp.	lettuce
Ascaris lumbricoides	basil
Ascaris lumbricoides	cress
Ascaris lumbricoides	mint
Ascaris lumbricoides	tarragon

Ascaris lumbricoides	parsley
Ascaris lumbricoides	lettuce
Ascaris lumbricoides	coriander
Ascaris lumbricoides	radish
Ascaris lumbricoides	chive
Ascaris lumbricoides	leek
Dicrocoelium dendriticum	mint
Dicrocoelium dendriticum	tarragon
Dicrocoelium dendriticum	chive
Dicrocoelium dendriticum	lettuce
Dicrocoelium dendriticum	coriander
Dicrocoelium dendriticum	radish
Dicrocoelium dendriticum	leek
Dicrocoelium dendriticum	basil
Dicrocoelium dendriticum	cress
Dicrocoelium dendriticum	parsley
Entamoeba coli	basil
Entamoeba coli	mint
Entamoeba coli	parsley
Entamoeba coli	lettuce
Entamoeba coli	radish
Entamoeba coli	leek
Entamoeba coli	cress
Entamoeba coli	tarragon
Entamoeba coli	chive
Entamoeba coli	coriander
Entamoeba histolytica	basil
Entamoeba histolytica	cress
Entamoeba histolytica	tarragon
Entamoeba histolytica	parsley
Entamoeba histolytica	chive
Entamoeba histolytica	lettuce
Entamoeba histolytica	coriander
Entamoeba histolytica	radish
Entamoeba histolytica	mint
Entamoeba histolytica	leek
Entamoeba histolytica	leek
Giardia lamblia	basil
Giardia lamblia	cress
Giardia lamblia	mint
Giardia lamblia	tarragon
Giardia lamblia	lettuce
Giardia lamblia	coriander
Giardia lamblia	radish
Giardia lamblia	parsley
Giardia lamblia	chive
Hymenolepis nana	basil
Hymenolepis nana	cress
Hymenolepis nana	mint
Hymenolepis nana	tarragon
Hymenolepis nana	parsley

Hymenolepis nana	chive
Hymenolepis nana	lettuce
Hymenolepis nana	coriander
Hymenolepis nana	radish
Hymenolepis nana	leek
Taenia spp.	basil
Taenia spp.	cress
Taenia spp.	mint
Taenia spp.	tarragon
Taenia spp.	chive
Taenia spp.	coriander
Taenia spp.	radish
Taenia spp.	leek
Taenia spp.	parsley
Taenia spp.	lettuce
Trichostrongylus spp.	basil
Trichostrongylus spp.	mint
Trichostrongylus spp.	tarragon
Trichostrongylus spp.	parsley
Trichostrongylus spp.	lettuce
Trichostrongylus spp.	coriander
Trichostrongylus spp.	radish
Trichostrongylus spp.	chive
Trichostrongylus spp.	cress
Trichostrongylus spp.	leek
Trichuris trichiura	basil
Trichuris trichiura	cress
Trichuris trichiura	mint
Trichuris trichiura	tarragon
Trichuris trichiura	chive
Trichuris trichiura	coriander
Trichuris trichiura	radish
Trichuris trichiura	leek
Trichuris trichiura	parsley
Trichuris trichiura	lettuce
Ascaris lumbricoides	lettuce
Ascaris lumbricoides	lettuce
Ascaris lumbricoides	lettuce
Entamoeba coli	lettuce
Entamoeba coli	lettuce
Entamoeba coli	lettuce
Entamoeba histolytica	lettuce
Entamoeba histolytica	lettuce
Entamoeba histolytica	lettuce
Giardia spp.	lettuce
Giardia spp.	lettuce
Giardia spp.	lettuce
Cryptosporidium spp.	sprouts
Cryptosporidium spp.	blueberry
Cryptosporidium spp.	cabbage
Cryptosporidium spp.	perilla
Cryptosporidium spp.	cherry tomato
Cryptosporidium spp.	chive
Cystoisospora spp.	blueberry
Cystoisospora spp.	sprouts

Cystoisospora spp.	cherry tomato
Cystoisospora spp.	cabbage
Entamoeba spp.	arugula
Entamoeba spp.	parsley
Entamoeba spp.	endive
Entamoeba spp.	lettuce
Entamoeba spp.	arugula
Entamoeba spp.	parsley
Entamoeba spp.	endive
Entamoeba spp.	lettuce
hookworms	arugula
hookworms	arugula
hookworms	endive
hookworms	endive
hookworms	lettuce
hookworms	lettuce
hookworms	lettuce
hookworms	lettuce
Strongyloides stercoralis	parsley
Strongyloides stercoralis	parsley
Strongyloides stercoralis	lettuce
Strongyloides stercoralis	lettuce
Strongyloides stercoralis	arugula
Strongyloides stercoralis	arugula
Strongyloides stercoralis	endive
Strongyloides stercoralis	endive
Trichostrongylidae	endive
Trichostrongylidae	endive
Trichostrongylidae	arugula
Trichostrongylidae	arugula
Trichostrongylidae	lettuce
Trichostrongylidae	lettuce
Trichostrongylidae	lettuce
Trichostrongylidae	lettuce
Dicrocoelium spp.	mint
Dicrocoelium spp.	chive
Dicrocoelium spp.	chive
Dicrocoelium spp.	coriander
Dicrocoelium spp.	basil
Dicrocoelium spp.	radish
Dicrocoelium spp.	spinach
Dicrocoelium spp.	lettuce
Dicrocoelium spp.	cress
Dicrocoelium spp.	parsley
Entamoeba coli	chive
Entamoeba coli	coriander
Entamoeba coli	spinach
Entamoeba coli	lettuce
Entamoeba coli	basil
Entamoeba coli	radish
Entamoeba coli	parsley

Entamoeba coli	mint
Entamoeba coli	chive
Entamoeba coli	cress
Entamoeba histolytica/dispar	lettuce
Entamoeba histolytica/dispar	mint
Entamoeba histolytica/dispar	cress
Entamoeba histolytica/dispar	chive
Entamoeba histolytica/dispar	chive
Entamoeba histolytica/dispar	coriander
Entamoeba histolytica/dispar	basil
Entamoeba histolytica/dispar	spinach
Entamoeba histolytica/dispar	radish
Entamoeba histolytica/dispar	parsley
Fasciola hepatica	lettuce
Fasciola hepatica	mint
Fasciola hepatica	cress
Fasciola hepatica	coriander
Fasciola hepatica	basil
Fasciola hepatica	radish
Fasciola hepatica	spinach
Fasciola hepatica	chive
Fasciola hepatica	chive
Fasciola hepatica	parsley
Giardia spp.	chive
Giardia spp.	mint
Giardia spp.	cress
Giardia spp.	chive
Giardia spp.	basil
Giardia spp.	parsley
Giardia spp.	coriander
Giardia spp.	spinach
Giardia spp.	lettuce
Giardia spp.	radish
Hymenolepis nana	spinach
Hymenolepis nana	mint
Hymenolepis nana	cress
Hymenolepis nana	chive
Hymenolepis nana	chive
Hymenolepis nana	coriander
Hymenolepis nana	basil
Hymenolepis nana	radish
Hymenolepis nana	lettuce
Hymenolepis nana	parsley
nematode	coriander
nematode	cress
nematode	chive
nematode	basil
nematode	radish
nematode	spinach
nematode	lettuce
nematode	mint
nematode	chive
nematode	parsley
unsporulated oocyst	mint

unsporulated oocyst	cress
unsporulated oocyst	chive
unsporulated oocyst	chive
unsporulated oocyst	coriander
unsporulated oocyst	basil
unsporulated oocyst	lettuce
unsporulated oocyst	radish
unsporulated oocyst	parsley
unsporulated oocyst	spinach
Taenia spp.	mint
Taenia spp.	cress
Taenia spp.	chive
Taenia spp.	chive
Taenia spp.	coriander
Taenia spp.	basil
Taenia spp.	radish
Taenia spp.	spinach
Taenia spp.	lettuce
Taenia spp.	parsley
Toxocara spp.	spinach
Toxocara spp.	cress
Toxocara spp.	chive
Toxocara spp.	chive
Toxocara spp.	coriander
Toxocara spp.	basil
Toxocara spp.	parsley
Toxocara spp.	lettuce
Toxocara spp.	mint
Toxocara spp.	radish
Trichostrongylus spp.	lettuce
Trichostrongylus spp.	mint
Trichostrongylus spp.	cress
Trichostrongylus spp.	chive
Trichostrongylus spp.	coriander
Trichostrongylus spp.	chive
Trichostrongylus spp.	basil
Trichostrongylus spp.	radish
Trichostrongylus spp.	parsley
Trichostrongylus spp.	spinach
Ascaris lumbricoides	arugula
Ascaris lumbricoides	lettuce
Ascaris lumbricoides	cress
Blastocystis hominis	arugula
Blastocystis hominis	lettuce
Blastocystis hominis	cress
Endolimax nana	cress
Endolimax nana	lettuce
Endolimax nana	arugula
Entamoeba spp.	arugula
Entamoeba spp.	lettuce

Entamoeba spp.	cress
Enterobius vermicularis	cress
Enterobius vermicularis	arugula
Enterobius vermicularis	lettuce
Giardia spp.	cress
Giardia spp.	arugula
Giardia spp.	lettuce
hookworms	arugula
hookworms	lettuce
hookworms	cress
Hymenolepis nana	arugula
Hymenolepis nana	lettuce
Hymenolepis nana	cress
Strongyloides spp.	arugula
Strongyloides spp.	lettuce
Strongyloides spp.	cress
Toxocara canis	lettuce
Toxocara canis	cress
Toxocara canis	arugula
Trichostrongylus spp.	arugula
Trichostrongylus spp.	lettuce
Trichostrongylus spp.	cress
Trichuris trichiura	lettuce
Trichuris trichiura	arugula
Trichuris trichiura	cress
Giardia lamblia	pumpkin
Giardia lamblia	carrot
Giardia lamblia	tomato
Giardia lamblia	ready-to-eat salad
Giardia lamblia	cabbage
Giardia lamblia	lettuce
Ascaris spp.	CANTAO
Ascaris spp.	cabbage
Ascaris spp.	mint
Ascaris spp.	celery
Ascaris spp.	leek
Ascaris spp.	green cabbage
Ascaris spp.	chive
Ascaris spp.	cabbage
Ascaris spp.	parsley
Ascaris spp.	lettuce
Enterobius vermicularis	celery
Enterobius vermicularis	lettuce
Fasciola spp.	cabbage
hookworms	leek
hookworms	chive
Blastocystis hominis	ready-to-eat salad
Cryptosporidium spp.	ready-to-eat salad
Cyclospora cayetanensis	ready-to-eat salad
Dientamoeba fragilis	ready-to-eat salad
Giardia lamblia	ready-to-eat salad
Toxoplasma gondii	ready-to-eat salad

Ascaris spp.	bean
Ascaris spp.	bean
Ascaris spp.	lettuce
Ascaris spp.	celery
Ascaris spp.	broccoli
Ascaris spp.	cauliflower
Ascaris spp.	anise
Ascaris spp.	anise
Ascaris spp.	turnip
Ascaris spp.	vinegar
Ascaris spp.	pumpkin
Ascaris spp.	lettuce
Ascaris spp.	cabbage
Ascaris spp.	potato
Ascaris spp.	cabbage
Ascaris spp.	parsley
Ascaris spp.	parsley
Ascaris spp.	strawberry
Ascaris spp.	rhubarb
Ascaris spp.	beetroot
Ascaris spp.	rhubarb
Ascaris spp.	beetroot
Ascaris spp.	onion
Ascaris spp.	leek
Toxocara spp.	turnip
Toxocara spp.	strawberry
Toxocara spp.	beetroot leaves
Toxocara spp.	potato
Toxocara spp.	celery
Toxocara spp.	beetroot
Toxocara spp.	rhubarb
Toxocara spp.	pumpkin
Toxocara spp.	carrot
Toxocara spp.	leek
Toxocara spp.	onion
Toxocara spp.	carrot
Trichuris spp.	onion
Trichuris spp.	rhubarb
Trichuris spp.	beetroot
Trichuris spp.	rhubarb
Trichuris spp.	beetroot
Cryptosporidium spp.	multiple vegetables
Giardia spp.	multiple vegetables
AMOEBA	multiple vegetables
Ascaris spp.	multiple vegetables
Cryptosporidium spp.	multiple vegetables
Entamoeba coli	multiple vegetables
Enterobius spp.	multiple vegetables
Fasciola spp.	multiple vegetables
Toxascaris leonina	multiple vegetables

Toxocara spp.	multiple vegetables
Trichuris spp.	multiple vegetables
Giardia lamblia	chive
Giardia lamblia	green cabbage
Giardia lamblia	arugula
Giardia lamblia	endive
Giardia lamblia	parsley
Giardia lamblia	cress
Giardia lamblia	lettuce
Cyclospora cayetanensis	marjoram
Cyclospora cayetanensis	mint
Cyclospora cayetanensis	lettuce
Cyclospora cayetanensis	basil
Cyclospora cayetanensis	coriander
Cyclospora cayetanensis	marjoram
Cyclospora cayetanensis	lettuce
Cyclospora cayetanensis	basil
Cyclospora cayetanensis	coriander
Cyclospora cayetanensis	mint
Cryptosporidium spp.	multiple vegetables
Giardia spp.	multiple vegetables
Cryptosporidium spp.	anise
Cryptosporidium spp.	carrot
Cryptosporidium spp.	cucumber
Cryptosporidium spp.	lettuce
Cryptosporidium spp.	cucumber
Cryptosporidium spp.	carrot
Cryptosporidium spp.	lettuce
Cryptosporidium spp.	anise
Giardia lamblia	anise
Giardia lamblia	cucumber
Giardia lamblia	lettuce
Giardia lamblia	carrot
Giardia lamblia	cucumber
Giardia lamblia	carrot
Giardia lamblia	lettuce
Giardia lamblia	anise
Ancylostoma duadenale	guava
Ancylostoma duadenale	spinach
Ancylostoma duadenale	banana
Ancylostoma duadenale	mango
Ancylostoma duadenale	onion
Ancylostoma duadenale	tomato
Ancylostoma duadenale	lettuce
Ancylostoma duadenale	cabbage
Ancylostoma duadenale	orange
Ancylostoma duadenale	carrot
Ascaris lumbricoides	guava
Ascaris lumbricoides	spinach
Ascaris lumbricoides	onion
Ascaris lumbricoides	mango
Ascaris lumbricoides	banana

Ascaris lumbricoides	carrot
Ascaris lumbricoides	cabbage
Ascaris lumbricoides	orange
Ascaris lumbricoides	tomato
Ascaris lumbricoides	lettuce
Entamoeba coli	guava
Entamoeba coli	mango
Entamoeba coli	spinach
Entamoeba coli	banana
Entamoeba coli	onion
Entamoeba coli	orange
Entamoeba coli	tomato
Entamoeba coli	carrot
Entamoeba coli	cabbage
Entamoeba coli	lettuce
Giardia lamblia	guava
Giardia lamblia	mango
Giardia lamblia	onion
Giardia lamblia	lettuce
Giardia lamblia	cabbage
Giardia lamblia	orange
Giardia lamblia	carrot
Giardia lamblia	spinach
Giardia lamblia	banana
Giardia lamblia	tomato
Hymenolepis nana	guava
Hymenolepis nana	spinach
Hymenolepis nana	lettuce
Hymenolepis nana	cabbage
Hymenolepis nana	orange
Hymenolepis nana	tomato
Hymenolepis nana	carrot
Hymenolepis nana	mango
Hymenolepis nana	banana
Hymenolepis nana	onion
Strongyloides stercoralis	guava
Strongyloides stercoralis	mango
Strongyloides stercoralis	spinach
Strongyloides stercoralis	banana
Strongyloides stercoralis	lettuce
Strongyloides stercoralis	tomato
Strongyloides stercoralis	carrot
Strongyloides stercoralis	orange
Strongyloides stercoralis	onion
Strongyloides stercoralis	cabbage
Taenia spp.	guava
Taenia spp.	mango
Taenia spp.	spinach
Taenia spp.	banana
Taenia spp.	tomato

Taenia spp.	onion
Taenia spp.	cabbage
Taenia spp.	orange
Taenia spp.	carrot
Taenia spp.	lettuce
Ascaris lumbricoides	onion
Ascaris lumbricoides	pepper
Ascaris lumbricoides	ginger
Ascaris lumbricoides	okra
Ascaris lumbricoides	cucumber
Ascaris lumbricoides	carrot
Ascaris lumbricoides	mustard
Ascaris lumbricoides	tomato
Ascaris lumbricoides	potato
Ascaris lumbricoides	yam
Ascaris lumbricoides	spinach
Ascaris lumbricoides	garlic
Ascaris lumbricoides	coriander
Entamoeba histolytica	ginger
Entamoeba histolytica	mustard
Entamoeba histolytica	cucumber
Entamoeba histolytica	onion
Entamoeba histolytica	spinach
Entamoeba histolytica	coriander
Entamoeba histolytica	pepper
Entamoeba histolytica	okra
Entamoeba histolytica	carrot
Entamoeba histolytica	yam
Entamoeba histolytica	garlic
Entamoeba histolytica	tomato
Entamoeba histolytica	potato
Taenia saginata	pepper
Taenia saginata	potato
Taenia saginata	cucumber
Taenia saginata	onion
Taenia saginata	spinach
Taenia saginata	coriander
Taenia saginata	ginger
Taenia saginata	okra
Taenia saginata	carrot
Taenia saginata	garlic
Taenia saginata	tomato
Taenia saginata	mustard
Taenia saginata	yam
Trichuris trichiura	yam
Trichuris trichiura	cucumber
Trichuris trichiura	ginger
Trichuris trichiura	potato
Trichuris trichiura	onion
Trichuris trichiura	spinach

Trichuris trichiura	pepper
Trichuris trichiura	carrot
Trichuris trichiura	garlic
Trichuris trichiura	mustard
Trichuris trichiura	tomato
Trichuris trichiura	coriander
Trichuris trichiura	okra
Cryptosporidium andersoni	corn cob husk
Cryptosporidium andersoni	annual sow-thistle
Cryptosporidium parvum	amaranth
Cryptosporidium parvum	chive
Cryptosporidium parvum	lettuce
Cryptosporidium parvum	lettuce
Cryptosporidium parvum	cabbage
Cryptosporidium parvum	asparagus leaves
Cryptosporidium parvum	cabbage
Giardia lamblia	chrysanthemum
Giardia lamblia	chive
Giardia lamblia	annual sow-thistle
Giardia lamblia	amaranth
Giardia lamblia	lettuce
Giardia lamblia	celery
Giardia lamblia	cabbage
Giardia lamblia	asparagus leaves
Giardia lamblia	cabbage
Giardia lamblia	lettuce
Ascaris spp.	cucumber
Ascaris spp.	cucumber
Ascaris spp.	tomato
Ascaris spp.	tomato
Ascaris spp.	parsley
Ascaris spp.	parsley
Ascaris spp.	lettuce
Ascaris spp.	lettuce
Ascaris spp.	cucumber
Ascaris spp.	cucumber
Ascaris spp.	tomato
Ascaris spp.	parsley
Ascaris spp.	lettuce
Ascaris spp.	parsley
Ascaris spp.	tomato
Ascaris spp.	lettuce
Entamoeba histolytica	lettuce
Entamoeba histolytica	lettuce
Entamoeba histolytica	tomato
Entamoeba histolytica	tomato
Fasciola spp.	tomato
Fasciola spp.	tomato
Fasciola spp.	parsley
Fasciola spp.	parsley

Fasciola spp.	lettuce
Fasciola spp.	lettuce
Fasciola spp.	lettuce
Fasciola spp.	lettuce
Giardia spp.	tomato
Giardia spp.	lettuce
Giardia spp.	lettuce
Giardia spp.	tomato
Giardia spp.	tomato
Giardia spp.	lettuce
Giardia spp.	tomato
Giardia spp.	lettuce
TaenialEchinococcus	parsley
TaenialEchinococcus	parsley
TaenialEchinococcus	lettuce
TaenialEchinococcus	lettuce
TaenialEchinococcus	parsley
TaenialEchinococcus	lettuce
TaenialEchinococcus	parsley
TaenialEchinococcus	lettuce
Toxocara spp.	lettuce
Toxocara spp.	lettuce
Toxocara spp.	cucumber
Toxocara spp.	lettuce
Toxocara spp.	cucumber
Toxocara spp.	lettuce
Ascaris lumbricoides	multiple vegetables
Dicrocoelium spp.	multiple vegetables
Entamoeba coli	multiple vegetables
Entamoeba histolytica/dispar	multiple vegetables
Fasciola spp.	multiple vegetables
Giardia spp.	multiple vegetables
Hymenolepis nana	multiple vegetables
Taenia/Echinococcus	multiple vegetables
Toxocara spp.	multiple vegetables
Trichostrongylus spp.	multiple vegetables

NATURE OF VEGETABLE	TYPE OF CONSUMPTION	STUDY COUNTRIES
herbaceous	mixed	Iran
herbaceous	mixed	Iran
herbaceous	mixed	Iran
herbaceous	mixed	Iran
tuberous	mixed	Iran
herbaceous	mixed	Iran
herbaceous	mixed	Iran
tuberous	mixed	Iran
herbaceous	raw	Iran
inconclusive	mixed	Iran
inconclusive	mixed	Iran
inconclusive	mixed	Iran
inconclusive	mixed	Iran
inconclusive	mixed	Iran
inconclusive	mixed	Iran
inconclusive	mixed	Iran
inconclusive	mixed	Iran
inconclusive	mixed	Iran
inconclusive	mixed	Iran
inconclusive	mixed	Iran
inconclusive	mixed	Iran
inconclusive	mixed	Iran
inconclusive	mixed	Iran
fruits	mixed	Iran
fruits	mixed	Iran
fruits	mixed	Iran
fruits	mixed	Iran
fruits	mixed	Iran
fruits	mixed	Iran
fruits	mixed	Iran
fruits	mixed	Iran
fruits	mixed	Iran
fruits	mixed	Iran
fruits	mixed	Iran
herbaceous	raw	Iran
fruits	mixed	Iran
tuberous	mixed	Iran
tuberous	mixed	Iran
herbaceous	mixed	Iran
herbaceous	mixed	Iran
herbaceous	raw	Iran
tuberous	mixed	Iran
fruits	mixed	Iran
fruits	mixed	Iran
fruits	mixed	Iran
fruits	mixed	Iran
fruits	mixed	Iran
fruits	mixed	Iran
tuberous	mixed	Iran

herbaceous	raw	Iran
herbaceous	mixed	Iran
fruits	mixed	Iran
fruits	mixed	Iran
fruits	mixed	Iran
fruits	mixed	Iran
fruits	mixed	Iran
tuberous	mixed	Iran
fruits	mixed	Iran
tuberous	mixed	Iran
herbaceous	mixed	Iran
herbaceous	raw	Iran
fruits	mixed	Iran
fruits	mixed	Iran
fruits	mixed	Iran
tuberous	mixed	Iran
fruits	mixed	Iran
fruits	mixed	Iran
fruits	mixed	Iran
herbaceous	mixed	Iran
tuberous	mixed	Iran
herbaceous	raw	Iran
fruits	mixed	Iran
fruits	mixed	Iran
fruits	mixed	Iran
fruits	mixed	Iran
fruits	mixed	Iran
fruits	mixed	Iran
fruits	mixed	Iran
fruits	mixed	Iran
tuberous	mixed	Iran
herbaceous	mixed	Iran
fruits	mixed	Iran
fruits	mixed	Iran
tuberous	mixed	Iran
herbaceous	raw	Iran
fruits	mixed	Iran
herbaceous	mixed	Iran
tuberous	mixed	Iran
fruits	mixed	Iran
herbaceous	raw	Iran
fruits	mixed	Iran
fruits	mixed	Iran
fruits	mixed	Iran
fruits	mixed	Iran
fruits	mixed	Iran
tuberous	mixed	Iran
fruits	mixed	Iran
herbaceous	mixed	Iran
herbaceous	raw	Iran
tuberous	mixed	Iran
fruits	mixed	Iran
fruits	mixed	Iran
fruits	mixed	Iran
fruits	mixed	Iran
fruits	mixed	Iran
fruits	mixed	Iran
tuberous	mixed	Iran
herbaceous	mixed	Iran

herbaceous	raw	Iran
tuberous	mixed	Iran
fruits	mixed	Iran
fruits	mixed	Iran
fruits	mixed	Iran
tuberous	mixed	Iran
fruits	mixed	Iran
fruits	mixed	Iran
tuberous	mixed	Iran
fruits	mixed	Iran
herbaceous	raw	Iran
herbaceous	mixed	Iran
herbaceous	raw	Brazil
herbaceous	raw	Brazil
herbaceous	raw	Brazil
herbaceous	raw	Brazil
herbaceous	raw	Brazil
herbaceous	raw	Brazil
herbaceous	raw	Brazil
herbaceous	raw	Brazil
herbaceous	raw	Brazil
herbaceous	raw	Brazil
herbaceous	raw	Brazil
herbaceous	raw	Brazil
herbaceous	mixed	Iraq
tuberous	cooked	Iraq
fruits	mixed	Iraq
tuberous	mixed	Iraq
herbaceous	mixed	Iraq
tuberous	mixed	Iraq
herbaceous	mixed	Iraq
tuberous	mixed	Iraq
herbaceous	mixed	Iraq
tuberous	mixed	Iraq
herbaceous	mixed	Iraq
herbaceous	raw	Iraq
herbaceous	mixed	Iraq
fruits	mixed	Iraq
herbaceous	mixed	Iraq
tuberous	cooked	Iraq
tuberous	mixed	Iraq
tuberous	mixed	Iraq
herbaceous	mixed	Iraq
tuberous	mixed	Iraq
herbaceous	raw	Iraq
herbaceous	mixed	Iraq
herbaceous	mixed	Iraq
tuberous	mixed	Iraq
tuberous	cooked	Iraq
herbaceous	mixed	Iraq
fruits	mixed	Iraq
herbaceous	mixed	Iraq
tuberous	mixed	Iraq
herbaceous	mixed	Iraq
tuberous	mixed	Iraq
herbaceous	mixed	Iraq
tuberous	mixed	Iraq
tuberous	mixed	Iraq
herbaceous	raw	Iraq
herbaceous	mixed	Iraq

tuberous	cooked	Iraq
tuberous	mixed	Iraq
herbaceous	mixed	Iraq
tuberous	mixed	Iraq
herbaceous	mixed	Iraq
herbaceous	mixed	Iraq
herbaceous	mixed	Iraq
tuberous	mixed	Iraq
fruits	mixed	Iraq
tuberous	mixed	Iraq
herbaceous	mixed	Iraq
herbaceous	raw	Iraq
tuberous	cooked	Iraq
tuberous	mixed	Iraq
fruits	mixed	Iraq
herbaceous	mixed	Iraq
herbaceous	mixed	Iraq
tuberous	mixed	Iraq
herbaceous	mixed	Iraq
herbaceous	raw	Iraq
tuberous	mixed	Iraq
tuberous	mixed	Iraq
herbaceous	mixed	Iraq
herbaceous	mixed	Iraq
herbaceous	raw	Iraq
tuberous	cooked	Iraq
herbaceous	mixed	Iraq
fruits	mixed	Iraq
herbaceous	mixed	Iraq
herbaceous	mixed	Iraq
tuberous	mixed	Iraq
tuberous	mixed	Iraq
tuberous	mixed	Iraq
herbaceous	mixed	Iraq
herbaceous	mixed	Iraq
tuberous	mixed	Iraq
fruits	mixed	Iraq
tuberous	cooked	Iraq
herbaceous	mixed	Iraq
tuberous	mixed	Iraq
herbaceous	mixed	Iraq
tuberous	mixed	Iraq
tuberous	mixed	Iraq
herbaceous	mixed	Iraq
herbaceous	raw	Iraq
tuberous	mixed	Iraq
herbaceous	mixed	Iraq
herbaceous	mixed	Iraq
herbaceous	mixed	Iraq
herbaceous	mixed	Iraq
tuberous	cooked	Iraq
herbaceous	mixed	Iraq
tuberous	mixed	Iraq

tuberous	mixed	Iraq
herbaceous	raw	Iraq
tuberous	mixed	Iraq
fruits	mixed	Iraq
herbaceous	mixed	Iraq
herbaceous	mixed	Iraq
tuberous	mixed	Iraq
tuberous	cooked	Iraq
tuberous	mixed	Iraq
herbaceous	mixed	Iraq
fruits	mixed	Iraq
herbaceous	mixed	Iraq
tuberous	mixed	Iraq
tuberous	mixed	Iraq
herbaceous	mixed	Iraq
tuberous	mixed	Iraq
herbaceous	mixed	Iraq
herbaceous	mixed	Iraq
herbaceous	raw	Iraq
herbaceous	mixed	Iraq
tuberous	cooked	Iraq
herbaceous	mixed	Iraq
fruits	mixed	Iraq
tuberous	mixed	Iraq
herbaceous	mixed	Iraq
tuberous	mixed	Iraq
herbaceous	mixed	Iraq
tuberous	mixed	Iraq
tuberous	mixed	Iraq
herbaceous	mixed	Iraq
herbaceous	raw	Iraq
fruits	mixed	Iraq
tuberous	cooked	Iraq
herbaceous	mixed	Iraq
herbaceous	mixed	Iraq
tuberous	mixed	Iraq
herbaceous	mixed	Iraq
tuberous	mixed	Iraq
tuberous	mixed	Iraq
herbaceous	mixed	Iraq
tuberous	mixed	Iraq
herbaceous	mixed	Iraq
herbaceous	raw	Iraq
herbaceous	mixed	Iraq
fruits	mixed	Iraq
tuberous	cooked	Iraq
tuberous	mixed	Iraq
herbaceous	mixed	Iraq
herbaceous	mixed	Iraq
tuberous	mixed	Iraq
tuberous	mixed	Iraq
herbaceous	mixed	Iraq

tuberous	mixed	Iraq
herbaceous	mixed	Iraq
herbaceous	raw	Iraq
herbaceous	mixed	Iraq
tuberous	cooked	Iraq
fruits	mixed	Iraq
tuberous	mixed	Iraq
herbaceous	mixed	Iraq
herbaceous	mixed	Iraq
tuberous	mixed	Iraq
herbaceous	mixed	Iraq
tuberous	mixed	Iraq
tuberous	mixed	Iraq
herbaceous	mixed	Iraq
herbaceous	raw	Iraq
fruits	mixed	Iraq
tuberous	cooked	Iraq
herbaceous	mixed	Iraq
tuberous	mixed	Iraq
tuberous	mixed	Iraq
tuberous	mixed	Iraq
herbaceous	mixed	Iraq
tuberous	mixed	Iraq
herbaceous	mixed	Iraq
herbaceous	mixed	Iraq
herbaceous	mixed	Iraq
herbaceous	raw	Iraq
fruits	mixed	Iraq
tuberous	cooked	Iraq
herbaceous	mixed	Iraq
herbaceous	mixed	Iraq
tuberous	mixed	Iraq
tuberous	mixed	Iraq
herbaceous	mixed	Iraq
tuberous	mixed	Iraq
tuberous	mixed	Iraq
herbaceous	raw	Iraq
herbaceous	mixed	Iraq
herbaceous	mixed	Iraq
herbaceous	mixed	Iraq
tuberous	cooked	Iraq
fruits	mixed	Iraq
tuberous	mixed	Iraq
herbaceous	mixed	Iraq
tuberous	mixed	Iraq
herbaceous	mixed	Iraq
tuberous	mixed	Iraq
tuberous	mixed	Iraq
herbaceous	mixed	Iraq
herbaceous	raw	Iraq
herbaceous	mixed	Iraq
herbaceous	mixed	Iran

herbaceous	mixed	Iran
tuberous	mixed	Iran
herbaceous	mixed	Iran
herbaceous	cooked	Iran
tuberous	mixed	Iran
herbaceous	raw	Iran
tuberous	mixed	Iran
herbaceous	mixed	Iran
tuberous	mixed	Iran
herbaceous	raw	Iran
herbaceous	cooked	Iran
herbaceous	mixed	Iran
herbaceous	mixed	Iran
tuberous	mixed	Iran
herbaceous	raw	Iran
herbaceous	mixed	Iran
herbaceous	mixed	Iran
tuberous	mixed	Iran
herbaceous	cooked	Iran
herbaceous	mixed	Iran
herbaceous	mixed	Iran
tuberous	mixed	Iran
herbaceous	mixed	Iran
tuberous	mixed	Iran
herbaceous	mixed	Iran
herbaceous	cooked	Iran
herbaceous	raw	Iran
tuberous	mixed	Iran
herbaceous	mixed	Iran
tuberous	mixed	Iran
herbaceous	raw	Iran
herbaceous	cooked	Iran
herbaceous	mixed	Iran
herbaceous	mixed	Iran
herbaceous	mixed	Iran
herbaceous	mixed	Iran
tuberous	mixed	Iran
herbaceous	mixed	Iran
herbaceous	cooked	Iran
herbaceous	raw	Iran
tuberous	mixed	Iran
herbaceous	mixed	Iran
tuberous	mixed	Iran
tuberous	mixed	Iran
herbaceous	raw	Iran
herbaceous	mixed	Iran
herbaceous	cooked	Iran
herbaceous	mixed	Iran
herbaceous	mixed	Saudi Arabia
herbaceous	mixed	Saudi Arabia
herbaceous	raw	Saudi Arabia

herbaceous	mixed	Saudi Arabia
herbaceous	mixed	Saudi Arabia
herbaceous	raw	Saudi Arabia
herbaceous	mixed	Saudi Arabia
herbaceous	raw	Saudi Arabia
herbaceous	mixed	Saudi Arabia
herbaceous	mixed	Saudi Arabia
herbaceous	mixed	Saudi Arabia
herbaceous	mixed	Saudi Arabia
herbaceous	mixed	Saudi Arabia
herbaceous	mixed	Saudi Arabia
fruits	cooked	Nigeria
herbaceous	mixed	Nigeria
fruits	raw	Nigeria
fruits	raw	Nigeria
herbaceous	raw	Nigeria
fruits	mixed	Nigeria
herbaceous	cooked	Nigeria
tuberous	mixed	Nigeria
herbaceous	mixed	Nigeria
herbaceous	mixed	Nigeria
fruits	cooked	Nigeria
herbaceous	cooked	Nigeria
herbaceous	mixed	Nigeria
fruits	mixed	Nigeria
tuberous	mixed	Nigeria
fruits	mixed	Nigeria
herbaceous	raw	Nigeria
fruits	raw	Nigeria
herbaceous	mixed	Nigeria
herbaceous	mixed	Nigeria
fruits	cooked	Nigeria
herbaceous	raw	Nigeria
fruits	raw	Nigeria
fruits	raw	Nigeria
herbaceous	mixed	Nigeria
tuberous	mixed	Nigeria
herbaceous	mixed	Nigeria
herbaceous	mixed	Nigeria
fruits	mixed	Nigeria
herbaceous	cooked	Nigeria
fruits	cooked	Nigeria
herbaceous	raw	Nigeria
fruits	raw	Nigeria
fruits	raw	Nigeria
herbaceous	mixed	Nigeria
tuberous	mixed	Nigeria
fruits	mixed	Nigeria
herbaceous	cooked	Nigeria
herbaceous	mixed	Nigeria

herbaceous	mixed	Nigeria
fruits	mixed	Libya
fruits	mixed	Libya
herbaceous	raw	Libya
herbaceous	mixed	Libya
fruits	mixed	Libya
herbaceous	mixed	Libya
fruits	mixed	Libya
herbaceous	raw	Libya
herbaceous	raw	Libya
fruits	mixed	Libya
herbaceous	mixed	Libya
herbaceous	raw	Libya
fruits	mixed	Libya
fruits	mixed	Libya
herbaceous	mixed	Libya
fruits	mixed	Libya
fruits	mixed	Libya
herbaceous	raw	Libya
herbaceous	mixed	Libya
fruits	mixed	Libya
fruits	mixed	Libya
herbaceous	mixed	Libya
herbaceous	raw	Libya
fruits	mixed	Libya
fruits	mixed	Libya
fruits	mixed	Libya
fruits	mixed	Libya
fruits	mixed	Libya
herbaceous	raw	Libya
herbaceous	mixed	Libya
herbaceous	mixed	Libya
herbaceous	raw	Libya
herbaceous	mixed	Libya
fruits	mixed	Libya
fruits	mixed	Libya
fruits	mixed	Libya
herbaceous	raw	Libya
fruits	mixed	Libya
herbaceous	raw	Libya
herbaceous	mixed	Libya
fruits	cooked	Nigeria
herbaceous	raw	Nigeria
fruits	mixed	Nigeria
herbaceous	raw	Nigeria
tuberous	mixed	Nigeria
herbaceous	mixed	Nigeria
herbaceous	raw	Nigeria
herbaceous	raw	Nigeria
fruits	cooked	Nigeria

herbaceous	raw	Turkey
herbaceous	mixed	Turkey
herbaceous	mixed	Turkey
herbaceous	mixed	Turkey
herbaceous	cooked	Turkey
tuberous	mixed	Turkey
herbaceous	mixed	Turkey
herbaceous	mixed	Turkey
herbaceous	cooked	Turkey
herbaceous	mixed	Syria
herbaceous	mixed	Syria
herbaceous	mixed	Syria
tuberous	mixed	Syria
herbaceous	mixed	Syria
herbaceous	mixed	Syria
herbaceous	mixed	Syria
herbaceous	raw	Syria
herbaceous	mixed	Syria
herbaceous	mixed	Syria
tuberous	mixed	Syria
herbaceous	mixed	Syria
herbaceous	mixed	Syria
herbaceous	mixed	Syria
herbaceous	mixed	Syria
herbaceous	raw	Syria
herbaceous	mixed	Syria
herbaceous	mixed	Syria
herbaceous	mixed	Syria
tuberous	mixed	Syria
herbaceous	mixed	Syria
herbaceous	mixed	Syria
herbaceous	raw	Syria
herbaceous	mixed	Syria
herbaceous	mixed	Syria
herbaceous	mixed	Syria
herbaceous	mixed	Syria
herbaceous	mixed	Syria
tuberous	mixed	Syria
herbaceous	mixed	Syria
herbaceous	mixed	Syria
herbaceous	raw	Syria
herbaceous	mixed	Syria
herbaceous	mixed	Syria
herbaceous	mixed	Syria
tuberous	mixed	Syria
herbaceous	mixed	Syria
herbaceous	mixed	Syria
herbaceous	raw	Syria
herbaceous	mixed	Syria
herbaceous	raw	Syria

herbaceous	mixed	Syria
herbaceous	mixed	Syria
herbaceous	mixed	Syria
herbaceous	mixed	Syria
tuberous	mixed	Syria
herbaceous	mixed	Syria
herbaceous	mixed	Syria
herbaceous	raw	Syria
herbaceous	mixed	Syria
herbaceous	mixed	Syria
herbaceous	mixed	Syria
herbaceous	mixed	Syria
herbaceous	mixed	Syria
herbaceous	mixed	Syria
tuberous	mixed	Syria
tuberous	mixed	Saudi Arabia
herbaceous	mixed	Saudi Arabia
herbaceous	raw	Saudi Arabia
tuberous	mixed	Saudi Arabia
herbaceous	mixed	Saudi Arabia
tuberous	mixed	Saudi Arabia
herbaceous	mixed	Saudi Arabia
herbaceous	raw	Saudi Arabia
tuberous	mixed	Saudi Arabia
herbaceous	raw	Saudi Arabia
herbaceous	mixed	Saudi Arabia
tuberous	mixed	Saudi Arabia
herbaceous	mixed	Saudi Arabia
tuberous	mixed	Saudi Arabia
herbaceous	mixed	Saudi Arabia
tuberous	mixed	Saudi Arabia
tuberous	mixed	Saudi Arabia
herbaceous	mixed	Saudi Arabia
herbaceous	mixed	Saudi Arabia
tuberous	mixed	Saudi Arabia
herbaceous	mixed	Saudi Arabia
herbaceous	raw	Saudi Arabia
tuberous	mixed	Saudi Arabia
tuberous	mixed	Saudi Arabia
herbaceous	raw	Saudi Arabia
tuberous	mixed	Saudi Arabia
herbaceous	mixed	Saudi Arabia
tuberous	mixed	Saudi Arabia
herbaceous	mixed	Saudi Arabia
herbaceous	raw	Saudi Arabia
tuberous	mixed	Saudi Arabia
herbaceous	mixed	Saudi Arabia
herbaceous	raw	Ethiopia
fruits	mixed	Ethiopia
herbaceous	mixed	Ethiopia

tuberous	mixed	Ethiopia
fruits	mixed	Ethiopia
fruits	mixed	Ethiopia
herbaceous	raw	Ethiopia
tuberous	mixed	Ethiopia
herbaceous	mixed	Ethiopia
fruits	mixed	Ethiopia
herbaceous	mixed	Ethiopia
herbaceous	raw	Ethiopia
fruits	mixed	Ethiopia
tuberous	mixed	Ethiopia
fruits	mixed	Ethiopia
tuberous	mixed	Ethiopia
herbaceous	mixed	Ethiopia
herbaceous	raw	Ethiopia
fruits	mixed	Ethiopia
fruits	mixed	Ethiopia
herbaceous	raw	Ethiopia
fruits	mixed	Ethiopia
herbaceous	mixed	Ethiopia
tuberous	mixed	Ethiopia
fruits	mixed	Ethiopia
herbaceous	raw	Ethiopia
fruits	mixed	Ethiopia
tuberous	mixed	Ethiopia
fruits	mixed	Ethiopia
herbaceous	mixed	Ethiopia
herbaceous	raw	Ethiopia
tuberous	mixed	Ethiopia
fruits	mixed	Ethiopia
fruits	mixed	Ethiopia
herbaceous	mixed	Ethiopia
herbaceous	raw	Ethiopia
tuberous	mixed	Ethiopia
fruits	mixed	Ethiopia
herbaceous	mixed	Ethiopia
fruits	mixed	Ethiopia
tuberous	mixed	Syria
herbaceous	mixed	Syria
herbaceous	mixed	Syria
herbaceous	raw	Syria
herbaceous	mixed	Syria
tuberous	mixed	Syria
herbaceous	mixed	Syria
herbaceous	mixed	Syria
herbaceous	raw	Syria
herbaceous	mixed	Syria
tuberous	mixed	Syria
herbaceous	mixed	Syria
herbaceous	mixed	Syria

herbaceous	raw	Syria
herbaceous	mixed	Syria
herbaceous	mixed	Syria
tuberous	mixed	Syria
herbaceous	mixed	Syria
herbaceous	raw	Syria
herbaceous	mixed	Syria
tuberous	mixed	United Arab Emirates
herbaceous	mixed	United Arab Emirates
herbaceous	mixed	United Arab Emirates
herbaceous	mixed	United Arab Emirates
fruits	mixed	United Arab Emirates
fruits	mixed	United Arab Emirates
tuberous	mixed	United Arab Emirates
herbaceous	mixed	United Arab Emirates
herbaceous	mixed	United Arab Emirates
herbaceous	mixed	United Arab Emirates
fruits	mixed	United Arab Emirates
herbaceous	mixed	United Arab Emirates
herbaceous	mixed	United Arab Emirates
herbaceous	mixed	United Arab Emirates
tuberous	mixed	United Arab Emirates
herbaceous	mixed	United Arab Emirates
herbaceous	mixed	United Arab Emirates
herbaceous	mixed	United Arab Emirates
herbaceous	raw	United Arab Emirates
herbaceous	raw	United Arab Emirates
tuberous	mixed	United Arab Emirates
herbaceous	raw	United Arab Emirates
herbaceous	mixed	United Arab Emirates
herbaceous	mixed	United Arab Emirates
herbaceous	mixed	United Arab Emirates
herbaceous	mixed	United Arab Emirates
herbaceous	mixed	United Arab Emirates
inconclusive	mixed	Saudi Arabia
inconclusive	mixed	Saudi Arabia
inconclusive	mixed	Saudi Arabia
inconclusive	mixed	Saudi Arabia
inconclusive	mixed	Saudi Arabia
inconclusive	mixed	Saudi Arabia
inconclusive	mixed	Saudi Arabia
inconclusive	mixed	Saudi Arabia
inconclusive	mixed	Saudi Arabia
inconclusive	mixed	Saudi Arabia
inconclusive	mixed	Saudi Arabia
herbaceous	raw	Brazil
herbaceous	mixed	Brazil
herbaceous	raw	Brazil
herbaceous	mixed	Brazil
herbaceous	raw	Brazil

herbaceous	mixed	Brazil
herbaceous	raw	Brazil
herbaceous	raw	Brazil
herbaceous	raw	Brazil
herbaceous	mixed	Brazil
herbaceous	raw	Brazil
herbaceous	raw	Brazil
herbaceous	mixed	Brazil
herbaceous	raw	Brazil
herbaceous	raw	Brazil
herbaceous	mixed	Brazil
herbaceous	mixed	Brazil
herbaceous	raw	Brazil
herbaceous	mixed	Brazil
herbaceous	raw	Brazil
herbaceous	raw	Brazil
herbaceous	raw	Brazil
herbaceous	raw	Brazil
herbaceous	raw	Spain
herbaceous	mixed	Spain
herbaceous	raw	Spain
herbaceous	raw	Spain
herbaceous	mixed	Spain
herbaceous	raw	Spain
fruits	raw	Nigeria
inconclusive	mixed	Nigeria
fruits	raw	Nigeria
inconclusive	mixed	Nigeria
inconclusive	mixed	Nigeria
fruits	raw	Nigeria
fruits	raw	Nigeria
inconclusive	mixed	Nigeria
inconclusive	mixed	Nigeria
fruits	raw	Nigeria
fruits	raw	Nigeria
inconclusive	mixed	Nigeria
fruits	raw	Nigeria
inconclusive	mixed	Nigeria
herbaceous	cooked	Cambodia
herbaceous	cooked	Cambodia
herbaceous	cooked	Cambodia
herbaceous	cooked	Cambodia
fruits	mixed	Brazil
tuberous	mixed	Brazil
herbaceous	raw	Brazil
fruits	mixed	Brazil
tuberous	mixed	Brazil
herbaceous	raw	Brazil
tuberous	mixed	Brazil
fruits	mixed	Brazil

herbaceous	raw	Brazil
tuberous	mixed	Brazil
fruits	mixed	Brazil
herbaceous	raw	Brazil
fruits	mixed	Brazil
herbaceous	raw	Brazil
tuberous	mixed	Brazil
herbaceous	mixed	Nepal
herbaceous	mixed	Nepal
tuberous	mixed	Nepal
tuberous	mixed	Nepal
herbaceous	mixed	Nepal
herbaceous	cooked	Nepal
herbaceous	cooked	Nepal
herbaceous	raw	Nepal
herbaceous	raw	Iran
herbaceous	mixed	Iran
herbaceous	raw	Iran
herbaceous	mixed	Iran
fruits	mixed	Nigeria
tuberous	mixed	Nigeria
fruits	mixed	Nigeria
herbaceous	mixed	Nigeria
fruits	cooked	Nigeria
herbaceous	mixed	Nigeria
fruits	mixed	Nigeria
herbaceous	cooked	Nigeria
herbaceous	mixed	Nigeria
herbaceous	mixed	Nigeria
herbaceous	cooked	Nigeria
tuberous	mixed	Nigeria
fruits	mixed	Nigeria
fruits	cooked	Nigeria
herbaceous	mixed	Nigeria
herbaceous	mixed	Nigeria
fruits	mixed	Nigeria
herbaceous	mixed	Nigeria
fruits	mixed	Nigeria
herbaceous	mixed	Nigeria
herbaceous	cooked	Nigeria
herbaceous	mixed	Nigeria
fruits	mixed	Nigeria
fruits	mixed	Nigeria
fruits	cooked	Nigeria
tuberous	mixed	Nigeria
fruits	mixed	Nigeria
herbaceous	mixed	Nigeria
herbaceous	mixed	Nigeria
herbaceous	mixed	Nigeria
fruits	cooked	Nigeria

herbaceous	mixed	Nigeria
tuberous	mixed	Nigeria
herbaceous	mixed	Nigeria
herbaceous	mixed	Nigeria
herbaceous	mixed	Nigeria
fruits	mixed	Nigeria
fruits	mixed	Nigeria
fruits	mixed	Nigeria
herbaceous	cooked	Nigeria
inconclusive	mixed	Nigeria
inconclusive	mixed	Nigeria
inconclusive	mixed	Nigeria
inconclusive	mixed	Nigeria
inconclusive	mixed	Nigeria
inconclusive	mixed	Nigeria
fruits	mixed	India
tuberous	mixed	India
tuberous	cooked	India
tuberous	mixed	India
herbaceous	mixed	India
herbaceous	mixed	India
herbaceous	cooked	India
tuberous	mixed	India
tuberous	mixed	India
herbaceous	mixed	India
inconclusive	mixed	Ethiopia
inconclusive	mixed	Ethiopia
inconclusive	mixed	Ethiopia
inconclusive	mixed	Ethiopia
inconclusive	mixed	Ethiopia
inconclusive	mixed	Ethiopia
inconclusive	mixed	Ethiopia
inconclusive	mixed	Ethiopia
inconclusive	mixed	Ethiopia
fruits	mixed	Ethiopia
fruits	raw	Ethiopia
tuberous	mixed	Ethiopia
herbaceous	raw	Ethiopia
fruits	mixed	Ethiopia
herbaceous	mixed	Ethiopia
fruits	raw	Ethiopia
fruits	mixed	Ethiopia
fruits	mixed	Ethiopia
herbaceous	raw	Ethiopia
tuberous	mixed	Ethiopia
herbaceous	mixed	Ethiopia
fruits	mixed	Ethiopia
herbaceous	raw	Ethiopia
fruits	raw	Ethiopia
fruits	mixed	Ethiopia
tuberous	mixed	Ethiopia
herbaceous	mixed	Ethiopia
fruits	raw	Ethiopia
fruits	mixed	Ethiopia
herbaceous	raw	Ethiopia
fruits	mixed	Ethiopia

tuberous	mixed	Ethiopia
herbaceous	mixed	Ethiopia
fruits	mixed	Ethiopia
herbaceous	raw	Ethiopia
fruits	raw	Ethiopia
tuberous	mixed	Ethiopia
fruits	mixed	Ethiopia
herbaceous	mixed	Ethiopia
fruits	mixed	Ethiopia
fruits	raw	Ethiopia
tuberous	mixed	Ethiopia
herbaceous	raw	Ethiopia
fruits	mixed	Ethiopia
herbaceous	mixed	Ethiopia
fruits	mixed	Ethiopia
fruits	raw	Ethiopia
fruits	mixed	Ethiopia
tuberous	mixed	Ethiopia
herbaceous	raw	Ethiopia
herbaceous	mixed	Ethiopia
herbaceous	raw	Morocco
herbaceous	mixed	Morocco
herbaceous	mixed	Morocco
herbaceous	raw	Morocco
herbaceous	mixed	Morocco
herbaceous	mixed	Morocco
tuberous	mixed	Morocco
herbaceous	raw	Morocco
tuberous	mixed	Morocco
herbaceous	mixed	Morocco
herbaceous	mixed	Morocco
herbaceous	raw	Portugal
herbaceous	raw	Portugal
herbaceous	raw	Portugal
herbaceous	raw	Portugal
herbaceous	raw	Portugal
fruits	raw	Portugal
fruits	raw	Portugal
herbaceous	raw	Spain
herbaceous	raw	Spain
herbaceous	raw	Spain
herbaceous	raw	Portugal
herbaceous	mixed	Portugal
tuberous	mixed	Portugal
herbaceous	mixed	Portugal
fruits	mixed	Portugal
fruits	mixed	Portugal
herbaceous	mixed	Spain
herbaceous	mixed	Portugal
herbaceous	mixed	Portugal
herbaceous	mixed	Portugal

herbaceous	mixed	Thailand
herbaceous	raw	Thailand
herbaceous	mixed	Thailand
herbaceous	mixed	Thailand
herbaceous	mixed	Thailand
herbaceous	mixed	Thailand
herbaceous	mixed	Thailand
tuberous	mixed	Thailand
herbaceous	mixed	Thailand
herbaceous	mixed	Thailand
herbaceous	raw	Thailand
herbaceous	mixed	Thailand
herbaceous	mixed	Thailand
herbaceous	mixed	Thailand
herbaceous	mixed	Thailand
herbaceous	mixed	Thailand
herbaceous	mixed	Thailand
herbaceous	mixed	Thailand
tuberous	mixed	Thailand
herbaceous	mixed	Thailand
herbaceous	mixed	Thailand
herbaceous	mixed	Thailand
herbaceous	mixed	Thailand
herbaceous	mixed	Thailand
herbaceous	mixed	Thailand
herbaceous	mixed	Thailand
herbaceous	mixed	Thailand
herbaceous	mixed	Thailand
herbaceous	mixed	Thailand
herbaceous	mixed	Venezuela
herbaceous	mixed	Venezuela
tuberous	mixed	Venezuela
fruits	mixed	Venezuela
herbaceous	mixed	Venezuela
herbaceous	mixed	Venezuela
tuberous	mixed	Venezuela
herbaceous	raw	Venezuela
fruits	mixed	Venezuela
herbaceous	mixed	Venezuela
herbaceous	mixed	Venezuela
tuberous	mixed	Venezuela
fruits	mixed	Venezuela
herbaceous	mixed	Venezuela
tuberous	mixed	Venezuela
herbaceous	mixed	Venezuela
fruits	mixed	Venezuela
herbaceous	mixed	Venezuela
herbaceous	raw	Venezuela
herbaceous	mixed	Venezuela
herbaceous	mixed	Venezuela

tuberous	mixed	Venezuela
fruits	mixed	Venezuela
tuberous	mixed	Venezuela
fruits	mixed	Venezuela
herbaceous	mixed	Venezuela
herbaceous	mixed	Venezuela
herbaceous	mixed	Venezuela
herbaceous	mixed	Venezuela
herbaceous	raw	Venezuela
herbaceous	mixed	Venezuela
tuberous	mixed	Venezuela
fruits	mixed	Venezuela
tuberous	mixed	Venezuela
fruits	mixed	Venezuela
herbaceous	mixed	Venezuela
herbaceous	mixed	Venezuela
herbaceous	raw	Venezuela
herbaceous	mixed	Venezuela
herbaceous	mixed	Venezuela
herbaceous	mixed	Venezuela
tuberous	mixed	Venezuela
fruits	mixed	Venezuela
herbaceous	mixed	Venezuela
tuberous	mixed	Venezuela
herbaceous	mixed	Venezuela
herbaceous	raw	Venezuela
fruits	mixed	Venezuela
herbaceous	mixed	Venezuela
herbaceous	mixed	Venezuela
herbaceous	mixed	Venezuela
tuberous	mixed	Venezuela
fruits	mixed	Venezuela
tuberous	mixed	Venezuela
herbaceous	mixed	Venezuela
herbaceous	raw	Venezuela
fruits	mixed	Venezuela
herbaceous	mixed	Venezuela
herbaceous	mixed	Venezuela
herbaceous	mixed	Venezuela
herbaceous	mixed	Venezuela
tuberous	mixed	Venezuela
fruits	mixed	Venezuela
herbaceous	mixed	Venezuela
tuberous	mixed	Venezuela
herbaceous	raw	Venezuela
fruits	mixed	Venezuela
herbaceous	mixed	Venezuela
herbaceous	mixed	Venezuela
herbaceous	mixed	Venezuela
herbaceous	mixed	Venezuela
tuberous	mixed	Venezuela
fruits	mixed	Venezuela

herbaceous	mixed	Venezuela
tuberous	mixed	Venezuela
herbaceous	mixed	Venezuela
herbaceous	raw	Venezuela
fruits	mixed	Venezuela
herbaceous	mixed	Venezuela
herbaceous	mixed	Venezuela
herbaceous	mixed	Venezuela
tuberous	mixed	Venezuela
fruits	mixed	Venezuela
herbaceous	mixed	Venezuela
tuberous	mixed	Venezuela
herbaceous	raw	Venezuela
herbaceous	mixed	Venezuela
herbaceous	mixed	Venezuela
herbaceous	mixed	Venezuela
fruits	mixed	Venezuela
herbaceous	mixed	Brazil
herbaceous	raw	Brazil
herbaceous	mixed	Egypt
tuberous	mixed	Egypt
herbaceous	mixed	Egypt
herbaceous	mixed	Egypt
herbaceous	raw	Egypt
herbaceous	mixed	Egypt
tuberous	mixed	Egypt
herbaceous	raw	Egypt
herbaceous	mixed	Egypt
herbaceous	raw	Egypt
herbaceous	mixed	Egypt
tuberous	mixed	Egypt
herbaceous	mixed	Egypt
herbaceous	mixed	Egypt
herbaceous	raw	Egypt
tuberous	mixed	Egypt
herbaceous	mixed	Egypt
herbaceous	raw	Egypt
herbaceous	raw	Egypt
herbaceous	mixed	Egypt
herbaceous	mixed	Egypt
herbaceous	raw	Egypt
tuberous	mixed	Egypt
herbaceous	mixed	Egypt
herbaceous	mixed	Egypt
herbaceous	raw	Egypt
herbaceous	mixed	Egypt
tuberous	mixed	Egypt
herbaceous	mixed	Egypt
herbaceous	mixed	Egypt
herbaceous	mixed	Egypt
herbaceous	raw	Egypt
herbaceous	raw	Brazil

herbaceous	mixed	Palestine
herbaceous	raw	Palestine
herbaceous	mixed	Palestine
herbaceous	raw	Palestine
herbaceous	mixed	Palestine
herbaceous	mixed	Palestine
herbaceous	mixed	Palestine
herbaceous	mixed	Palestine
herbaceous	mixed	Palestine
herbaceous	mixed	Palestine
herbaceous	mixed	Palestine
herbaceous	mixed	Palestine
herbaceous	raw	Canada
herbaceous	raw	Canada
herbaceous	raw	Canada
tuberous	mixed	Egypt
herbaceous	mixed	Egypt
herbaceous	raw	Egypt
tuberous	mixed	Egypt
herbaceous	mixed	Egypt
herbaceous	raw	Egypt
tuberous	mixed	Egypt
herbaceous	raw	Egypt
herbaceous	mixed	Egypt
tuberous	mixed	Egypt
herbaceous	mixed	Egypt
herbaceous	raw	Egypt
tuberous	mixed	Egypt
herbaceous	mixed	Egypt
herbaceous	raw	Egypt
tuberous	mixed	Egypt
herbaceous	raw	Egypt
herbaceous	mixed	Egypt
herbaceous	mixed	Egypt
herbaceous	mixed	Egypt
herbaceous	mixed	Egypt
herbaceous	mixed	Egypt
herbaceous	mixed	Egypt
herbaceous	mixed	Egypt
herbaceous	mixed	Egypt
herbaceous	mixed	Egypt
herbaceous	mixed	Egypt
herbaceous	mixed	Egypt
herbaceous	mixed	Egypt
herbaceous	mixed	Egypt
herbaceous	mixed	Turkey
herbaceous	cooked	Turkey
herbaceous	mixed	Turkey
herbaceous	raw	Turkey
fruits	raw	Turkey
herbaceous	cooked	Turkey
herbaceous	mixed	Turkey
herbaceous	raw	Turkey

herbaceous	mixed	Turkey
fruits	raw	Turkey
herbaceous	mixed	Turkey
herbaceous	cooked	Turkey
herbaceous	raw	Turkey
herbaceous	mixed	Turkey
fruits	raw	Turkey
herbaceous	cooked	Turkey
herbaceous	mixed	Turkey
herbaceous	mixed	Turkey
herbaceous	raw	Turkey
fruits	raw	Turkey
tuberous	mixed	Iran
herbaceous	mixed	Iran
herbaceous	mixed	Iran
tuberous	mixed	Iran
herbaceous	mixed	Iran
herbaceous	mixed	Iran
herbaceous	mixed	Iran
herbaceous	mixed	Iran
tuberous	mixed	Iran
tuberous	mixed	Iran
herbaceous	mixed	Iran
herbaceous	mixed	Iran
tuberous	mixed	Iran
tuberous	mixed	Iran
herbaceous	mixed	Iran
herbaceous	mixed	Iran
herbaceous	mixed	Iran
herbaceous	mixed	Iran
tuberous	mixed	Iran
tuberous	mixed	Iran
tuberous	mixed	Iran
herbaceous	mixed	Iran
tuberous	mixed	Iran
herbaceous	mixed	Iran
herbaceous	mixed	Iran
herbaceous	mixed	Iran
tuberous	mixed	Iran
herbaceous	mixed	Iran
tuberous	mixed	Iran
herbaceous	mixed	Iran
herbaceous	mixed	Iran
herbaceous	mixed	Iran
herbaceous	mixed	Iran
tuberous	mixed	Iran
tuberous	mixed	Iran
herbaceous	mixed	Iran
herbaceous	mixed	Iran
tuberous	mixed	Iran
herbaceous	mixed	Iran
tuberous	mixed	Iran
inconclusive	mixed	Ethiopia

inconclusive	mixed	Ethiopia
inconclusive	mixed	Ethiopia
inconclusive	mixed	Ethiopia
inconclusive	mixed	Ethiopia
inconclusive	mixed	Ethiopia
inconclusive	mixed	Ethiopia
herbaceous	mixed	Iran
herbaceous	mixed	Iran
herbaceous	mixed	Iran
herbaceous	mixed	Iran
herbaceous	mixed	Iran
herbaceous	mixed	Iran
herbaceous	mixed	Iran
tuberous	mixed	Iran
tuberous	mixed	Iran
herbaceous	mixed	Iran
herbaceous	mixed	Iran
herbaceous	mixed	Iran
herbaceous	mixed	Iran
herbaceous	mixed	Iran
herbaceous	mixed	Iran
tuberous	mixed	Iran
herbaceous	mixed	Iran
tuberous	mixed	Iran
herbaceous	mixed	Iran
herbaceous	mixed	Iran
herbaceous	mixed	Iran
herbaceous	mixed	Iran
herbaceous	mixed	Iran
herbaceous	mixed	Iran
herbaceous	mixed	Iran
herbaceous	mixed	Iran
herbaceous	mixed	Iran
herbaceous	mixed	Iran
herbaceous	mixed	Iran
tuberous	mixed	Iran
herbaceous	mixed	Iran
herbaceous	mixed	Iran
tuberous	mixed	Iran
herbaceous	mixed	Iran
herbaceous	mixed	Iran
herbaceous	mixed	Iran
herbaceous	mixed	Iran
herbaceous	mixed	Iran
tuberous	mixed	Iran
herbaceous	mixed	Iran
tuberous	mixed	Iran
herbaceous	mixed	Iran
herbaceous	mixed	Iran
herbaceous	mixed	Iran
herbaceous	mixed	Iran
herbaceous	mixed	Iran
herbaceous	mixed	Iran
herbaceous	mixed	Iran

herbaceous	mixed	Iran
herbaceous	mixed	Iran
herbaceous	mixed	Iran
tuberous	mixed	Iran
herbaceous	mixed	Iran
tuberous	mixed	Iran
herbaceous	mixed	Iran
tuberous	mixed	Iran
herbaceous	mixed	Iran
tuberous	mixed	Iran
herbaceous	mixed	Iran
herbaceous	mixed	Iran
herbaceous	mixed	Iran
herbaceous	mixed	Iran
herbaceous	mixed	Iran
herbaceous	mixed	Iran
herbaceous	mixed	Iran
herbaceous	mixed	Iran
herbaceous	raw	Brazil
herbaceous	mixed	Brazil
herbaceous	mixed	Brazil
herbaceous	cooked	Brazil
herbaceous	mixed	Brazil
herbaceous	mixed	Brazil
herbaceous	mixed	Brazil
herbaceous	raw	Brazil
herbaceous	mixed	Brazil
herbaceous	raw	Brazil
tuberous	mixed	Saudi Arabia
fruits	mixed	Saudi Arabia
herbaceous	raw	Saudi Arabia
fruits	cooked	Saudi Arabia
herbaceous	mixed	Saudi Arabia
fruits	mixed	Saudi Arabia
herbaceous	raw	Saudi Arabia
fruits	mixed	Saudi Arabia
herbaceous	mixed	Saudi Arabia
tuberous	mixed	Saudi Arabia
fruits	cooked	Saudi Arabia
herbaceous	mixed	Saudi Arabia
herbaceous	mixed	Saudi Arabia
fruits	cooked	Saudi Arabia
herbaceous	mixed	Saudi Arabia
tuberous	mixed	Saudi Arabia
herbaceous	raw	Saudi Arabia
herbaceous	mixed	Saudi Arabia
fruits	mixed	Saudi Arabia
herbaceous	mixed	Saudi Arabia
tuberous	mixed	Saudi Arabia
herbaceous	mixed	Saudi Arabia
tuberous	mixed	Saudi Arabia
fruits	mixed	Saudi Arabia
herbaceous	mixed	Saudi Arabia
fruits	mixed	Saudi Arabia
herbaceous	mixed	Saudi Arabia
herbaceous	raw	Saudi Arabia
herbaceous	mixed	Saudi Arabia
fruits	cooked	Saudi Arabia

herbaceous	raw	Egypt
herbaceous	mixed	Egypt
fruits	mixed	Egypt
fruits	mixed	Egypt
tuberous	mixed	Egypt
herbaceous	mixed	Egypt
tuberous	mixed	Egypt
herbaceous	mixed	Egypt
herbaceous	mixed	Egypt
herbaceous	mixed	Egypt
herbaceous	mixed	Egypt
herbaceous	mixed	Egypt
fruits	mixed	Egypt
tuberous	mixed	Egypt
herbaceous	mixed	Egypt
herbaceous	raw	Egypt
tuberous	mixed	Egypt
fruits	mixed	Egypt
herbaceous	mixed	Egypt
fruits	mixed	Egypt
herbaceous	mixed	Egypt
tuberous	mixed	Egypt
tuberous	mixed	Egypt
fruits	mixed	Egypt
herbaceous	raw	Egypt
herbaceous	mixed	Egypt
herbaceous	mixed	Egypt
tuberous	mixed	Egypt
tuberous	mixed	Egypt
herbaceous	mixed	Egypt
fruits	mixed	Egypt
herbaceous	raw	Egypt
herbaceous	mixed	Egypt
herbaceous	mixed	Egypt
fruits	mixed	Egypt
herbaceous	mixed	Egypt
fruits	mixed	Egypt
herbaceous	mixed	Egypt
tuberous	mixed	Egypt
tuberous	mixed	Egypt
fruits	mixed	Egypt
herbaceous	mixed	Egypt
herbaceous	mixed	Egypt
herbaceous	mixed	Egypt
herbaceous	raw	Egypt
tuberous	mixed	Egypt
herbaceous	mixed	Egypt
fruits	mixed	Egypt
herbaceous	raw	Egypt
tuberous	mixed	Egypt
herbaceous	mixed	Egypt
herbaceous	mixed	Egypt
herbaceous	mixed	Egypt
fruits	mixed	Egypt
herbaceous	mixed	Philippines
herbaceous	raw	Philippines
herbaceous	mixed	Philippines

herbaceous	raw	Philippines
herbaceous	mixed	Philippines
herbaceous	mixed	Philippines
herbaceous	mixed	Philippines
herbaceous	raw	Philippines
herbaceous	mixed	Philippines
herbaceous	mixed	Philippines
herbaceous	raw	Philippines
herbaceous	mixed	Philippines
herbaceous	raw	Philippines
herbaceous	mixed	Philippines
herbaceous	raw	Brazil
herbaceous	raw	Brazil
herbaceous	raw	Brazil
herbaceous	raw	Brazil
herbaceous	raw	Brazil
herbaceous	raw	Brazil
herbaceous	raw	Brazil
herbaceous	raw	Brazil
herbaceous	raw	Brazil
herbaceous	raw	Brazil
herbaceous	raw	Brazil
herbaceous	raw	Brazil
herbaceous	raw	Brazil
herbaceous	raw	Brazil
herbaceous	raw	Brazil
herbaceous	raw	Brazil
herbaceous	raw	Brazil
herbaceous	raw	Brazil
herbaceous	raw	Brazil
herbaceous	raw	Brazil
herbaceous	raw	Brazil
herbaceous	raw	Brazil
herbaceous	raw	Brazil
herbaceous	raw	Brazil
herbaceous	raw	Brazil
herbaceous	raw	Brazil
herbaceous	raw	Brazil
herbaceous	raw	Brazil
herbaceous	raw	Brazil
herbaceous	raw	Brazil
herbaceous	raw	Brazil
herbaceous	raw	Brazil
herbaceous	raw	Brazil
herbaceous	raw	Brazil
herbaceous	raw	Brazil
herbaceous	raw	Brazil
herbaceous	raw	Brazil
herbaceous	raw	Brazil
herbaceous	raw	Brazil
herbaceous	raw	Brazil
herbaceous	raw	Brazil
herbaceous	raw	Brazil
herbaceous	raw	Brazil
herbaceous	raw	Brazil
herbaceous	raw	Brazil
herbaceous	raw	Brazil
herbaceous	raw	Brazil

herbaceous	raw	Brazil
herbaceous	raw	Brazil
herbaceous	raw	Brazil
herbaceous	raw	Brazil
herbaceous	raw	Brazil
herbaceous	raw	Brazil
inconclusive	mixed	Iraq
inconclusive	mixed	Iraq
inconclusive	mixed	Iraq
inconclusive	mixed	Iraq
inconclusive	mixed	Iraq
inconclusive	mixed	Iraq
inconclusive	mixed	Iraq
inconclusive	mixed	Iraq
inconclusive	mixed	Iraq
inconclusive	mixed	Iraq
inconclusive	mixed	Iraq
inconclusive	mixed	Iraq
inconclusive	mixed	Iraq
inconclusive	mixed	Iraq
inconclusive	mixed	Iraq
inconclusive	mixed	Iraq
inconclusive	mixed	Iraq
inconclusive	mixed	Iraq
inconclusive	mixed	Iraq
inconclusive	mixed	Iraq
inconclusive	mixed	Iraq
herbaceous	mixed	Iraq
tuberous	mixed	Iraq
herbaceous	mixed	Iraq
herbaceous	mixed	Iraq
herbaceous	raw	Iraq
herbaceous	mixed	Iraq
herbaceous	mixed	Iraq
tuberous	mixed	Iraq
herbaceous	mixed	Iraq
herbaceous	mixed	Iraq
herbaceous	raw	Iraq
herbaceous	mixed	Iraq
herbaceous	mixed	Iraq
tuberous	mixed	Iraq
herbaceous	mixed	Iraq
herbaceous	mixed	Iraq
herbaceous	raw	Iraq
herbaceous	mixed	Iraq
herbaceous	mixed	Iraq
tuberous	mixed	Iraq
herbaceous	mixed	Iraq
herbaceous	mixed	Iraq
herbaceous	raw	Iraq
herbaceous	mixed	Iraq
herbaceous	mixed	Iraq
tuberous	mixed	Iraq
herbaceous	mixed	Iraq
herbaceous	mixed	Iraq
herbaceous	raw	Iraq
herbaceous	mixed	Iraq
herbaceous	mixed	Iraq

tuberous	mixed	Iraq
herbaceous	raw	Iraq
herbaceous	mixed	Iraq
herbaceous	mixed	Iraq
herbaceous	mixed	Iraq
herbaceous	mixed	Iraq
tuberous	mixed	Iraq
herbaceous	mixed	Iraq
herbaceous	raw	Iraq
herbaceous	mixed	Iraq
herbaceous	mixed	Iraq
herbaceous	mixed	Iraq
tuberous	mixed	Iraq
herbaceous	raw	Iraq
herbaceous	mixed	Iraq
herbaceous	mixed	Iraq
herbaceous	mixed	Iraq
herbaceous	mixed	Iraq
tuberous	mixed	Iraq
herbaceous	raw	Iraq
herbaceous	mixed	Iraq
herbaceous	mixed	Iraq
herbaceous	mixed	Iraq
herbaceous	mixed	Iraq
tuberous	mixed	Iraq
herbaceous	mixed	Iraq
herbaceous	mixed	Iraq
herbaceous	raw	Iraq
herbaceous	mixed	Iraq
fruits	raw	Greece
fruits	raw	Greece
fruits	raw	Greece
fruits	raw	Greece
fruits	raw	Greece
fruits	raw	Greece
fruits	raw	Greece
fruits	raw	Greece
herbaceous	mixed	Greece
tuberous	cooked	Greece
fruits	cooked	Greece
herbaceous	cooked	Greece
herbaceous	raw	Greece
fruits	raw	Greece
herbaceous	mixed	Greece
tuberous	mixed	Greece
herbaceous	mixed	Greece
tuberous	mixed	Greece
tuberous	mixed	Greece
herbaceous	mixed	Greece
fruits	mixed	Greece
fruits	mixed	Greece
herbaceous	mixed	Greece
herbaceous	mixed	Greece
fruits	mixed	Greece
fruits	raw	Greece
herbaceous	mixed	Greece
fruits	raw	Greece
fruits	raw	Greece

fruits	raw	Greece
fruits	raw	Greece
fruits	raw	Greece
fruits	raw	Greece
fruits	raw	Greece
fruits	raw	Greece
herbaceous	mixed	Greece
tuberous	cooked	Greece
fruits	cooked	Greece
herbaceous	cooked	Greece
herbaceous	raw	Greece
fruits	raw	Greece
fruits	raw	Greece
herbaceous	mixed	Greece
tuberous	mixed	Greece
herbaceous	mixed	Greece
tuberous	mixed	Greece
tuberous	mixed	Greece
herbaceous	mixed	Greece
herbaceous	mixed	Greece
fruits	mixed	Greece
fruits	mixed	Greece
herbaceous	mixed	Greece
herbaceous	mixed	Greece
fruits	mixed	Greece
herbaceous	cooked	Iran
herbaceous	cooked	Iran
fruits	mixed	Iran
herbaceous	mixed	Iran
herbaceous	mixed	Iran
tuberous	mixed	Iran
fruits	cooked	Iran
tuberous	mixed	Iran
fruits	mixed	Iran
herbaceous	mixed	Iran
herbaceous	mixed	Iran
herbaceous	mixed	Iran
herbaceous	mixed	Iran
tuberous	mixed	Iran
herbaceous	raw	Iran
herbaceous	cooked	Iran
herbaceous	cooked	Iran
fruits	mixed	Iran
herbaceous	mixed	Iran
tuberous	mixed	Iran
fruits	cooked	Iran
herbaceous	mixed	Iran
herbaceous	mixed	Iran
herbaceous	mixed	Iran
fruits	mixed	Iran
tuberous	mixed	Iran
herbaceous	mixed	Iran
tuberous	mixed	Iran
herbaceous	mixed	Iran
herbaceous	raw	Iran
herbaceous	cooked	Iran
herbaceous	mixed	Iran
herbaceous	mixed	Iran

herbaceous	mixed	Iran
herbaceous	mixed	Iran
fruits	mixed	Iran
tuberous	mixed	Iran
herbaceous	mixed	Iran
herbaceous	cooked	Iran
tuberous	mixed	Iran
tuberous	mixed	Iran
herbaceous	raw	Iran
herbaceous	cooked	Iran
herbaceous	mixed	Iran
herbaceous	cooked	Iran
fruits	mixed	Iran
herbaceous	mixed	Iran
tuberous	mixed	Iran
fruits	cooked	Iran
fruits	mixed	Iran
herbaceous	mixed	Iran
herbaceous	mixed	Iran
tuberous	mixed	Iran
herbaceous	mixed	Iran
herbaceous	mixed	Iran
tuberous	mixed	Iran
herbaceous	raw	Iran
herbaceous	mixed	Iran
herbaceous	cooked	Iran
herbaceous	mixed	Iran
tuberous	mixed	Iran
herbaceous	mixed	Iran
fruits	mixed	Iran
herbaceous	mixed	Iran
herbaceous	raw	Iran
herbaceous	mixed	Iran
tuberous	mixed	Iran
herbaceous	cooked	Iran
tuberous	mixed	Iran
herbaceous	cooked	Iran
herbaceous	mixed	Iran
herbaceous	mixed	Iran
herbaceous	mixed	Iran
herbaceous	cooked	Iran
herbaceous	mixed	Iran
herbaceous	mixed	Iran
fruits	cooked	Iran
fruits	mixed	Iran
tuberous	mixed	Iran
tuberous	mixed	Iran
fruits	mixed	Iran
herbaceous	mixed	Iran
herbaceous	raw	Iran
tuberous	mixed	Iran
herbaceous	cooked	Iran
tuberous	mixed	Iran
herbaceous	mixed	Iran
herbaceous	raw	Iran
herbaceous	cooked	Iran
fruits	mixed	Iran
tuberous	mixed	Iran

fruits	mixed	Iran
herbaceous	mixed	Iran
fruits	cooked	Iran
herbaceous	mixed	Iran
tuberous	mixed	Iran
herbaceous	mixed	Iran
herbaceous	mixed	Iran
herbaceous	mixed	Iran
herbaceous	mixed	Iran
herbaceous	cooked	Iran
fruits	mixed	Iran
herbaceous	cooked	Iran
herbaceous	mixed	Iran
tuberous	mixed	Iran
tuberous	mixed	Iran
herbaceous	mixed	Iran
herbaceous	mixed	Iran
herbaceous	mixed	Iran
tuberous	mixed	Iran
herbaceous	raw	Iran
herbaceous	mixed	Iran
herbaceous	cooked	Iran
herbaceous	mixed	Iran
tuberous	mixed	Iran
herbaceous	mixed	Iran
herbaceous	mixed	Iran
herbaceous	mixed	Iran
herbaceous	mixed	Iran
herbaceous	cooked	Iran
fruits	cooked	Iran
fruits	mixed	Iran
tuberous	mixed	Iran
herbaceous	raw	Iran
fruits	mixed	Iran
tuberous	mixed	Iran
herbaceous	mixed	Iran
herbaceous	cooked	Iran
herbaceous	cooked	Iran
tuberous	mixed	Iran
herbaceous	raw	Iran
herbaceous	mixed	Iran
herbaceous	mixed	Iran
herbaceous	mixed	Iran
herbaceous	mixed	Iran
fruits	mixed	Iran
tuberous	mixed	Iran
tuberous	mixed	Iran
herbaceous	cooked	Iran
herbaceous	mixed	Iran
fruits	cooked	Iran
herbaceous	mixed	Iran
fruits	mixed	Iran
herbaceous	mixed	Iran

herbaceous	cooked	Iran
fruits	mixed	Iran
tuberous	mixed	Iran
tuberous	mixed	Iran
herbaceous	mixed	Iran
herbaceous	mixed	Iran
herbaceous	mixed	Iran
tuberous	mixed	Iran
herbaceous	raw	Iran
herbaceous	mixed	Iran
herbaceous	cooked	Iran
tuberous	mixed	Iran
herbaceous	mixed	Iran
tuberous	mixed	Iran
herbaceous	mixed	Iran
herbaceous	mixed	Iran
herbaceous	mixed	Iran
herbaceous	cooked	Iran
herbaceous	raw	Iran
tuberous	mixed	Iran
fruits	mixed	Iran
herbaceous	cooked	Iran
fruits	mixed	Iran
herbaceous	mixed	Iran
tuberous	mixed	Iran
fruits	cooked	Iran
tuberous	mixed	Iran
herbaceous	cooked	Iran
herbaceous	mixed	Iran
herbaceous	mixed	Iran
herbaceous	mixed	Iran
fruits	mixed	Iran
herbaceous	mixed	Iran
herbaceous	mixed	Iran
tuberous	mixed	Iran
herbaceous	raw	Iran
herbaceous	mixed	Iran
herbaceous	cooked	Iran
fruits	mixed	Iran
herbaceous	mixed	Iran
tuberous	mixed	Iran
tuberous	mixed	Iran
herbaceous	mixed	Iran
herbaceous	mixed	Iran
herbaceous	cooked	Iran
herbaceous	mixed	Iran
tuberous	mixed	Iran
herbaceous	raw	Iran
herbaceous	cooked	Iran
herbaceous	mixed	Iran
herbaceous	cooked	Iran
fruits	mixed	Iran
herbaceous	mixed	Iran

herbaceous	mixed	Iran
tuberous	mixed	Iran
tuberous	mixed	Iran
herbaceous	mixed	Iran
herbaceous	raw	Iran
herbaceous	mixed	Iran
fruits	cooked	Iran
fruits	mixed	Iran
tuberous	mixed	Iran
herbaceous	mixed	Iran
herbaceous	cooked	Iran
tuberous	mixed	Iran
tuberous	mixed	Iran
fruits	mixed	Iran
herbaceous	mixed	Iran
herbaceous	cooked	Iran
tuberous	mixed	Iran
herbaceous	mixed	Iran
herbaceous	mixed	Iran
herbaceous	raw	Iran
herbaceous	mixed	Iran
herbaceous	mixed	Iran
fruits	mixed	Iran
herbaceous	mixed	Iran
herbaceous	cooked	Iran
herbaceous	mixed	Iran
fruits	mixed	Iran
herbaceous	mixed	Iran
tuberous	mixed	Iran
herbaceous	cooked	Iran
fruits	cooked	Iran
tuberous	mixed	Iran
herbaceous	mixed	Iran
herbaceous	mixed	Iran
herbaceous	mixed	Iran
tuberous	mixed	Iran
herbaceous	raw	Iran
fruits	mixed	Iran
herbaceous	mixed	Iran
herbaceous	mixed	Iran
tuberous	mixed	Iran
herbaceous	cooked	Iran
tuberous	mixed	Iran
herbaceous	mixed	Iran
herbaceous	mixed	Iran
herbaceous	mixed	Iran
herbaceous	cooked	Iran
herbaceous	raw	Iran
tuberous	mixed	Iran
herbaceous	cooked	Iran
tuberous	mixed	Iran

herbaceous	mixed	Iran
herbaceous	mixed	Iran
herbaceous	mixed	Iran
fruits	cooked	Iran
herbaceous	mixed	Iran
herbaceous	mixed	Iran
herbaceous	cooked	Iran
fruits	mixed	Iran
herbaceous	mixed	Iran
tuberous	mixed	Iran
fruits	mixed	Iran
herbaceous	raw	Iran
tuberous	mixed	Iran
herbaceous	mixed	Iran
herbaceous	mixed	Iran
tuberous	mixed	Iran
herbaceous	mixed	Iran
herbaceous	mixed	Iran
tuberous	mixed	Iran
herbaceous	mixed	Iran
herbaceous	cooked	Iran
fruits	mixed	Iran
herbaceous	cooked	Iran
tuberous	mixed	Iran
herbaceous	raw	Iran
herbaceous	mixed	Iran
herbaceous	cooked	Iran
herbaceous	mixed	Iran
herbaceous	cooked	Iran
tuberous	mixed	Iran
herbaceous	mixed	Iran
herbaceous	mixed	Iran
fruits	cooked	Iran
herbaceous	mixed	Iran
herbaceous	mixed	Iran
fruits	mixed	Iran
fruits	mixed	Iran
tuberous	mixed	Iran
herbaceous	raw	Iran
tuberous	mixed	Iran
herbaceous	mixed	Iran
herbaceous	cooked	Iran
herbaceous	cooked	Iran
herbaceous	mixed	Iran
tuberous	mixed	Iran
tuberous	mixed	Iran
herbaceous	mixed	Iran
herbaceous	mixed	Iran
fruits	mixed	Iran
herbaceous	mixed	Iran
herbaceous	raw	Iran

tuberous	mixed	Iran
herbaceous	raw	Egypt
herbaceous	mixed	Egypt
fruits	mixed	Egypt
herbaceous	mixed	Egypt
tuberous	mixed	Egypt
fruits	mixed	Egypt
herbaceous	mixed	Egypt
herbaceous	mixed	Egypt
tuberous	mixed	Egypt
fruits	mixed	Egypt
herbaceous	mixed	Egypt
herbaceous	raw	Egypt
fruits	mixed	Egypt
tuberous	mixed	Egypt
herbaceous	mixed	Egypt
herbaceous	mixed	Egypt
tuberous	mixed	Egypt
herbaceous	mixed	Egypt
fruits	mixed	Egypt
fruits	mixed	Egypt
herbaceous	mixed	Egypt
herbaceous	raw	Egypt
herbaceous	mixed	Egypt
tuberous	mixed	Egypt
fruits	mixed	Egypt
herbaceous	mixed	Egypt
herbaceous	mixed	Egypt
fruits	mixed	Egypt
fruits	mixed	Egypt
tuberous	mixed	Egypt
herbaceous	mixed	Spain
herbaceous	raw	Spain
herbaceous	raw	Spain
herbaceous	mixed	Spain
herbaceous	raw	Spain
herbaceous	raw	Spain
inconclusive	mixed	Nigeria
inconclusive	mixed	Nigeria
inconclusive	mixed	Nigeria
inconclusive	mixed	Nigeria
inconclusive	mixed	Nigeria
inconclusive	mixed	Nigeria
inconclusive	mixed	Nigeria
inconclusive	mixed	Poland
fruits	mixed	Poland
fruits	raw	Poland
inconclusive	mixed	European countries
inconclusive	mixed	Switzerland
inconclusive	mixed	European countries

inconclusive	mixed	European countries
inconclusive	mixed	Switzerland
inconclusive	mixed	European countries
inconclusive	mixed	Switzerland
inconclusive	mixed	European countries
inconclusive	mixed	European countries
inconclusive	mixed	Switzerland
inconclusive	mixed	European countries
inconclusive	mixed	Switzerland
fruits	raw	Pakistan
herbaceous	mixed	Pakistan
tuberous	mixed	Pakistan
fruits	raw	Pakistan
herbaceous	mixed	Pakistan
tuberous	mixed	Pakistan
fruits	raw	Pakistan
tuberous	mixed	Pakistan
herbaceous	mixed	Pakistan
tuberous	cooked	Korea
herbaceous	cooked	Korea
fruits	raw	Korea
herbaceous	mixed	Korea
fruits	mixed	Korea
tuberous	mixed	Korea
fruits	mixed	Korea
herbaceous	mixed	Korea
fruits	cooked	Poland
fruits	cooked	Poland
tuberous	mixed	Poland
herbaceous	mixed	Poland
inconclusive	mixed	Poland
herbaceous	mixed	Poland
herbaceous	mixed	Poland
herbaceous	raw	Poland
herbaceous	mixed	Poland
herbaceous	mixed	Poland
herbaceous	mixed	Poland
herbaceous	mixed	Poland
inconclusive	mixed	Poland
tuberous	mixed	Poland
herbaceous	mixed	Poland
tuberous	mixed	Poland
tuberous	mixed	Poland
fruits	mixed	Poland
fruits	mixed	Poland
herbaceous	mixed	Poland
fruits	cooked	Poland
herbaceous	raw	Poland
herbaceous	mixed	Poland
tuberous	cooked	Poland
tuberous	cooked	Poland

fruits	raw	Poland
herbaceous	mixed	Poland
herbaceous	mixed	Poland
herbaceous	mixed	Poland
tuberous	mixed	Poland
herbaceous	mixed	Poland
fruits	cooked	Poland
tuberous	mixed	Poland
fruits	raw	Poland
herbaceous	mixed	Poland
tuberous	mixed	Poland
herbaceous	mixed	Poland
tuberous	mixed	Poland
tuberous	mixed	Poland
tuberous	mixed	Poland
fruits	cooked	Poland
fruits	cooked	Poland
tuberous	mixed	Poland
herbaceous	mixed	Poland
inconclusive	mixed	Poland
herbaceous	mixed	Poland
herbaceous	mixed	Poland
herbaceous	raw	Poland
herbaceous	mixed	Poland
herbaceous	mixed	Poland
herbaceous	mixed	Poland
herbaceous	mixed	Poland
tuberous	mixed	Poland
herbaceous	mixed	Poland
tuberous	mixed	Poland
tuberous	mixed	Poland
fruits	mixed	Poland
fruits	mixed	Poland
herbaceous	mixed	Poland
fruits	cooked	Poland
herbaceous	raw	Poland
herbaceous	mixed	Poland
tuberous	cooked	Poland
fruits	raw	Poland
herbaceous	mixed	Poland
herbaceous	mixed	Poland
herbaceous	mixed	Poland
herbaceous	mixed	Poland
tuberous	mixed	Poland
fruits	raw	Poland
inconclusive	mixed	Poland
tuberous	cooked	Poland
herbaceous	mixed	Poland
tuberous	mixed	Poland
herbaceous	mixed	Poland
fruits	cooked	Poland

tuberous	mixed	Poland
tuberous	mixed	Poland
tuberous	mixed	Poland
tuberous	mixed	Poland
fruits	cooked	Poland
fruits	cooked	Poland
tuberous	mixed	Poland
herbaceous	mixed	Poland
inconclusive	mixed	Poland
herbaceous	mixed	Poland
herbaceous	mixed	Poland
herbaceous	raw	Poland
herbaceous	mixed	Poland
herbaceous	mixed	Poland
herbaceous	mixed	Poland
herbaceous	mixed	Poland
inconclusive	mixed	Poland
tuberous	mixed	Poland
herbaceous	mixed	Poland
tuberous	mixed	Poland
tuberous	mixed	Poland
fruits	mixed	Poland
fruits	mixed	Poland
herbaceous	mixed	Poland
fruits	cooked	Poland
herbaceous	raw	Poland
herbaceous	mixed	Poland
tuberous	cooked	Poland
tuberous	cooked	Poland
fruits	raw	Poland
herbaceous	mixed	Poland
herbaceous	mixed	Poland
herbaceous	mixed	Poland
tuberous	mixed	Poland
herbaceous	mixed	Poland
fruits	cooked	Poland
tuberous	mixed	Poland
tuberous	mixed	Poland
fruits	raw	Poland
tuberous	mixed	Poland
herbaceous	mixed	Poland
tuberous	mixed	Poland
herbaceous	mixed	Poland
tuberous	mixed	Poland
herbaceous	mixed	Turkey
herbaceous	mixed	Turkey
herbaceous	mixed	Turkey
fruits	mixed	Turkey
fruits	mixed	Turkey
fruits	mixed	Turkey

tuberous	mixed	Turkey
herbaceous	mixed	Turkey
herbaceous	raw	Turkey
herbaceous	mixed	Turkey
herbaceous	mixed	Turkey
fruits	mixed	Turkey
fruits	mixed	Turkey
herbaceous	raw	Turkey
herbaceous	mixed	Turkey
fruits	mixed	Turkey
tuberous	mixed	Turkey
herbaceous	mixed	Turkey
herbaceous	mixed	Turkey
herbaceous	mixed	Turkey
herbaceous	mixed	Turkey
fruits	mixed	Turkey
fruits	mixed	Turkey
fruits	mixed	Turkey
tuberous	mixed	Turkey
herbaceous	mixed	Turkey
herbaceous	raw	Turkey
herbaceous	mixed	Ghana
tuberous	mixed	Ghana
herbaceous	raw	Ghana
herbaceous	mixed	Ghana
fruits	mixed	Ghana
fruits	mixed	Ghana
tuberous	mixed	Ghana
herbaceous	mixed	Ghana
herbaceous	mixed	Ghana
herbaceous	raw	Ghana
herbaceous	mixed	Ghana
tuberous	mixed	Ghana
herbaceous	mixed	Ghana
fruits	mixed	Ghana
herbaceous	raw	Ghana
fruits	mixed	Ghana
herbaceous	mixed	Ghana
tuberous	mixed	Ghana
herbaceous	raw	Ghana
herbaceous	mixed	Ghana
herbaceous	mixed	Canada
inconclusive	mixed	Canada
herbaceous	mixed	Canada
inconclusive	mixed	Canada
herbaceous	raw	Canada
herbaceous	raw	Canada
herbaceous	raw	Canada
herbaceous	mixed	Canada
herbaceous	cooked	Canada

herbaceous	mixed	Canada
inconclusive	mixed	Canada
herbaceous	mixed	Canada
inconclusive	mixed	Canada
herbaceous	raw	Canada
herbaceous	raw	Canada
herbaceous	raw	Canada
herbaceous	cooked	Canada
herbaceous	mixed	Canada
herbaceous	mixed	Canada
inconclusive	mixed	Canada
herbaceous	mixed	Canada
inconclusive	mixed	Canada
herbaceous	mixed	Canada
herbaceous	raw	Canada
herbaceous	raw	Canada
herbaceous	raw	Canada
herbaceous	cooked	Canada
fruits	raw	Poland
tuberous	mixed	Poland
herbaceous	raw	Poland
tuberous	mixed	Poland
fruits	raw	Poland
tuberous	mixed	Poland
tuberous	mixed	Poland
herbaceous	raw	Poland
fruits	raw	Poland
fruits	raw	Poland
inconclusive	mixed	Poland
tuberous	cooked	China
fruits	mixed	China
herbaceous	cooked	China
fruits	raw	China
herbaceous	mixed	China
herbaceous	cooked	China
herbaceous	mixed	China
herbaceous	mixed	China
herbaceous	mixed	China
herbaceous	raw	China
herbaceous	cooked	China
fruits	cooked	China
fruits	mixed	China
herbaceous	raw	China
herbaceous	mixed	China
herbaceous	mixed	China
herbaceous	mixed	China
herbaceous	mixed	China
herbaceous	mixed	China
herbaceous	raw	China
herbaceous	mixed	China

tuberous	cooked	China
fruits	mixed	China
herbaceous	cooked	China
fruits	raw	China
herbaceous	mixed	China
herbaceous	cooked	China
herbaceous	mixed	China
herbaceous	mixed	China
herbaceous	mixed	China
herbaceous	raw	China
herbaceous	cooked	China
fruits	cooked	China
fruits	mixed	China
herbaceous	mixed	China
herbaceous	mixed	China
herbaceous	mixed	China
herbaceous	mixed	China
herbaceous	mixed	China
herbaceous	mixed	China
herbaceous	raw	China
herbaceous	raw	China
fruits	mixed	China
herbaceous	cooked	China
herbaceous	mixed	China
herbaceous	cooked	China
herbaceous	mixed	China
herbaceous	raw	China
herbaceous	mixed	China
tuberous	cooked	China
fruits	raw	China
herbaceous	mixed	China
herbaceous	mixed	China
fruits	mixed	China
herbaceous	raw	China
herbaceous	mixed	China
herbaceous	mixed	China
herbaceous	mixed	China
herbaceous	cooked	China
fruits	cooked	China
herbaceous	mixed	China
herbaceous	mixed	China
herbaceous	raw	China
herbaceous	mixed	Brazil
herbaceous	mixed	Brazil
herbaceous	raw	Brazil
herbaceous	raw	Brazil
herbaceous	mixed	Brazil
herbaceous	mixed	Brazil
herbaceous	mixed	Brazil
herbaceous	raw	Brazil
herbaceous	mixed	Brazil

herbaceous	raw	Brazil
herbaceous	mixed	Brazil
herbaceous	mixed	Brazil
herbaceous	raw	Brazil
herbaceous	raw	Brazil
herbaceous	mixed	Brazil
herbaceous	mixed	Brazil
herbaceous	mixed	Brazil
herbaceous	mixed	Brazil
herbaceous	mixed	Brazil
herbaceous	raw	Brazil
herbaceous	mixed	Brazil
herbaceous	mixed	Brazil
herbaceous	mixed	Brazil
herbaceous	mixed	Brazil
herbaceous	raw	Brazil
herbaceous	mixed	Brazil
herbaceous	raw	Brazil
herbaceous	raw	Brazil
herbaceous	mixed	Brazil
herbaceous	mixed	Brazil
herbaceous	raw	Brazil
herbaceous	raw	Brazil
herbaceous	mixed	Brazil
herbaceous	mixed	Brazil
herbaceous	mixed	Brazil
herbaceous	mixed	Brazil
herbaceous	raw	Brazil
herbaceous	mixed	Brazil
herbaceous	mixed	Brazil
herbaceous	raw	Brazil
herbaceous	mixed	Brazil
herbaceous	mixed	Brazil
herbaceous	mixed	Brazil
herbaceous	raw	Brazil
herbaceous	mixed	Brazil
herbaceous	raw	Brazil
herbaceous	mixed	Brazil
herbaceous	mixed	Brazil
herbaceous	raw	Brazil
herbaceous	raw	Brazil
herbaceous	mixed	Brazil
herbaceous	mixed	Brazil
herbaceous	mixed	Brazil
herbaceous	mixed	Brazil
herbaceous	mixed	Brazil
herbaceous	mixed	Brazil
herbaceous	raw	Brazil
herbaceous	raw	Brazil
herbaceous	mixed	Brazil
herbaceous	mixed	Brazil

herbaceous	mixed	Brazil
herbaceous	mixed	Brazil
herbaceous	raw	Brazil
herbaceous	mixed	Brazil
herbaceous	raw	Brazil
herbaceous	mixed	Brazil
herbaceous	raw	Brazil
herbaceous	mixed	Brazil
herbaceous	mixed	Brazil
herbaceous	mixed	Brazil
herbaceous	mixed	Brazil
herbaceous	raw	Brazil
herbaceous	mixed	Brazil
herbaceous	raw	Brazil
herbaceous	mixed	Brazil
herbaceous	mixed	Brazil
herbaceous	mixed	Brazil
herbaceous	mixed	Brazil
herbaceous	raw	Brazil
herbaceous	raw	Brazil
herbaceous	mixed	Brazil
herbaceous	raw	Brazil
herbaceous	mixed	Brazil
herbaceous	mixed	Brazil
herbaceous	raw	Brazil
herbaceous	raw	Brazil
herbaceous	mixed	Brazil
herbaceous	mixed	Brazil
herbaceous	mixed	Brazil
herbaceous	mixed	Brazil
fruits	raw	Costa Rica
herbaceous	mixed	Costa Rica
herbaceous	mixed	Costa Rica
fruits	raw	Costa Rica
herbaceous	raw	Costa Rica
fruits	raw	Costa Rica
fruits	raw	Costa Rica
herbaceous	mixed	Costa Rica
herbaceous	mixed	Costa Rica
herbaceous	raw	Costa Rica
herbaceous	raw	Iran
tuberous	mixed	Iran
herbaceous	mixed	Iran
tuberous	mixed	Iran
herbaceous	mixed	Iran
herbaceous	mixed	Iran
tuberous	mixed	Iran
herbaceous	mixed	Iran
herbaceous	mixed	Iran
tuberous	mixed	Iran

herbaceous	mixed	Iran
herbaceous	raw	Iran
herbaceous	mixed	Iran
herbaceous	mixed	Iran
herbaceous	mixed	Iran
tuberous	mixed	Iran
herbaceous	raw	Iran
herbaceous	mixed	Iran
tuberous	mixed	Iran
herbaceous	mixed	Iran
herbaceous	mixed	Iran
herbaceous	mixed	Iran
herbaceous	raw	Iran
tuberous	mixed	Iran
herbaceous	mixed	Iran
herbaceous	mixed	Iran
herbaceous	raw	Iran
herbaceous	mixed	Iran
tuberous	mixed	Iran
herbaceous	raw	Iran
tuberous	mixed	Iran
tuberous	mixed	Iran
herbaceous	mixed	Iran
herbaceous	mixed	Iran
herbaceous	mixed	Iran
tuberous	mixed	Iran
herbaceous	mixed	Iran
herbaceous	mixed	Iran
tuberous	mixed	Iran
herbaceous	mixed	Iran
herbaceous	raw	Iran
tuberous	mixed	Iran
herbaceous	mixed	Iran
herbaceous	mixed	Iran
tuberous	mixed	Iran
herbaceous	mixed	Iran
tuberous	mixed	Iran
herbaceous	mixed	Iran
herbaceous	raw	Iran
herbaceous	raw	Iran
herbaceous	raw	Iran
herbaceous	mixed	Iran
herbaceous	mixed	Iran
herbaceous	mixed	Iran
herbaceous	mixed	Iran
herbaceous	mixed	Iran
herbaceous	mixed	Iran
herbaceous	mixed	Iran
tuberous	mixed	Iran
herbaceous	mixed	Iran

herbaceous	mixed	Iran
herbaceous	mixed	Iran
herbaceous	mixed	Iran
herbaceous	mixed	Iran
herbaceous	mixed	Iran
tuberous	mixed	Iran
herbaceous	raw	Iran
herbaceous	raw	Iran
tuberous	mixed	Iran
tuberous	mixed	Iran
herbaceous	mixed	Iran
tuberous	mixed	Iran
inconclusive	mixed	Philippines
inconclusive	mixed	Philippines
inconclusive	mixed	Philippines
inconclusive	mixed	Philippines
inconclusive	mixed	Philippines
inconclusive	mixed	Philippines
herbaceous	mixed	Malaysia
herbaceous	mixed	Malaysia
herbaceous	mixed	Malaysia
tuberous	mixed	Malaysia
herbaceous	mixed	Nigeria
tuberous	mixed	Nigeria
fruits	mixed	Nigeria
herbaceous	mixed	Nigeria
herbaceous	cooked	Nigeria
herbaceous	mixed	Nigeria
fruits	cooked	Nigeria
herbaceous	raw	Nigeria
herbaceous	mixed	Brazil
herbaceous	mixed	Brazil
herbaceous	mixed	Brazil
herbaceous	raw	Brazil
fruits	mixed	Sudan
herbaceous	raw	Sudan
herbaceous	mixed	Sudan
herbaceous	mixed	Sudan
tuberous	mixed	Sudan
herbaceous	mixed	Sudan
tuberous	mixed	Sudan
fruits	mixed	Sudan
herbaceous	mixed	Sudan
tuberous	mixed	Sudan
fruits	mixed	Sudan
fruits	mixed	Sudan
herbaceous	mixed	Sudan
tuberous	mixed	Sudan
tuberous	mixed	Sudan
herbaceous	mixed	Sudan

fruits	mixed	Sudan
fruits	mixed	Sudan
herbaceous	raw	Sudan
herbaceous	mixed	Sudan
tuberous	mixed	Sudan
herbaceous	mixed	Sudan
fruits	mixed	Sudan
herbaceous	mixed	Sudan
herbaceous	mixed	Sudan
tuberous	mixed	Sudan
herbaceous	raw	Sudan
tuberous	mixed	Sudan
fruits	mixed	Sudan
herbaceous	mixed	Sudan
fruits	mixed	Sudan
tuberous	mixed	Sudan
herbaceous	mixed	Sudan
fruits	mixed	Sudan
herbaceous	raw	Sudan
herbaceous	mixed	Sudan
herbaceous	mixed	Sudan
tuberous	mixed	Sudan
fruits	mixed	Sudan
herbaceous	mixed	Sudan
fruits	mixed	Sudan
tuberous	mixed	Sudan
tuberous	mixed	Sudan
herbaceous	mixed	Sudan
fruits	mixed	Sudan
herbaceous	raw	Sudan
herbaceous	mixed	Sudan
herbaceous	mixed	Sudan
tuberous	mixed	Sudan
tuberous	mixed	Sudan
fruits	mixed	Sudan
herbaceous	mixed	Sudan
fruits	mixed	Sudan
tuberous	mixed	Sudan
herbaceous	mixed	Sudan
fruits	mixed	Sudan
herbaceous	raw	Sudan
herbaceous	mixed	Sudan
tuberous	mixed	Sudan
herbaceous	mixed	Sudan
tuberous	mixed	Sudan
fruits	mixed	Sudan
herbaceous	mixed	Sudan
fruits	mixed	Sudan
tuberous	mixed	Sudan
herbaceous	mixed	Sudan

herbaceous	mixed	India
herbaceous	mixed	India
herbaceous	mixed	India
herbaceous	mixed	India
herbaceous	cooked	India
herbaceous	raw	India
herbaceous	mixed	India
herbaceous	mixed	India
herbaceous	mixed	India
herbaceous	mixed	India
herbaceous	cooked	India
herbaceous	raw	India
herbaceous	mixed	India
herbaceous	mixed	India
herbaceous	mixed	India
herbaceous	raw	India
herbaceous	mixed	India
herbaceous	cooked	India
herbaceous	mixed	India
herbaceous	raw	Brazil
herbaceous	raw	Brazil
herbaceous	raw	Brazil
herbaceous	raw	Brazil
herbaceous	raw	Brazil
herbaceous	raw	Brazil
herbaceous	raw	Brazil
herbaceous	raw	Brazil
herbaceous	raw	Brazil
herbaceous	raw	Brazil
herbaceous	raw	Brazil
herbaceous	raw	Brazil
herbaceous	raw	Brazil
herbaceous	raw	Brazil
herbaceous	raw	Brazil
herbaceous	raw	Brazil
herbaceous	raw	Brazil
herbaceous	raw	Brazil
herbaceous	raw	Brazil
herbaceous	raw	Brazil
herbaceous	raw	Brazil
herbaceous	raw	Brazil
herbaceous	raw	Brazil
herbaceous	raw	Brazil
herbaceous	raw	Brazil
herbaceous	raw	Brazil
herbaceous	raw	Brazil
herbaceous	raw	Brazil
herbaceous	raw	Brazil
herbaceous	raw	Brazil
herbaceous	raw	Brazil
herbaceous	raw	Brazil

herbaceous	raw	Brazil
herbaceous	raw	Brazil
herbaceous	raw	Brazil
herbaceous	raw	Brazil
herbaceous	raw	Brazil
herbaceous	raw	Brazil
herbaceous	raw	Brazil
herbaceous	raw	Brazil
herbaceous	raw	Brazil
herbaceous	raw	Brazil
herbaceous	raw	Brazil
herbaceous	raw	Brazil
herbaceous	raw	Brazil
herbaceous	raw	Brazil
herbaceous	raw	Brazil
herbaceous	raw	Brazil
herbaceous	raw	Brazil
herbaceous	raw	Brazil
herbaceous	raw	Brazil
herbaceous	raw	Brazil
herbaceous	mixed	Turkey
herbaceous	cooked	Turkey
herbaceous	mixed	Turkey
herbaceous	raw	Turkey
fruits	raw	Turkey
herbaceous	cooked	Turkey
herbaceous	mixed	Turkey
herbaceous	raw	Turkey
herbaceous	mixed	Turkey
fruits	raw	Turkey
herbaceous	mixed	Turkey
herbaceous	cooked	Turkey
herbaceous	raw	Turkey
herbaceous	mixed	Turkey
fruits	raw	Turkey
herbaceous	cooked	Turkey
herbaceous	mixed	Turkey
herbaceous	mixed	Turkey
herbaceous	raw	Turkey
fruits	raw	Turkey
herbaceous	raw	Nigeria
herbaceous	cooked	Nigeria
herbaceous	raw	Nigeria
fruits	cooked	Nigeria
herbaceous	cooked	Nigeria
herbaceous	raw	Nigeria
herbaceous	cooked	Nigeria
inconclusive	mixed	Iran
inconclusive	mixed	Iran
inconclusive	mixed	Iran

inconclusive	mixed	Iran
inconclusive	mixed	Iran
inconclusive	mixed	Iran
inconclusive	mixed	Iran
inconclusive	mixed	Iran
inconclusive	mixed	Iran
inconclusive	mixed	Iran
inconclusive	mixed	Iran
inconclusive	mixed	Iran
inconclusive	mixed	Iran
herbaceous	raw	Iraq
herbaceous	raw	Iraq
tuberous	mixed	Iraq
fruits	raw	Iraq
herbaceous	mixed	Iraq
tuberous	mixed	Iraq
fruits	raw	Iraq
fruits	mixed	Iraq
fruits	raw	Iraq
fruits	mixed	Iraq
tuberous	mixed	Iraq
herbaceous	raw	Iraq
tuberous	mixed	Iraq
tuberous	mixed	Iraq
fruits	raw	Iraq
herbaceous	mixed	Iraq
tuberous	mixed	Iraq
fruits	raw	Iraq
fruits	mixed	Iraq
fruits	raw	Iraq
fruits	mixed	Iraq
herbaceous	raw	Brazil
herbaceous	raw	Brazil
herbaceous	raw	Cuba
tuberous	mixed	Cuba
herbaceous	mixed	Cuba
tuberous	mixed	Cuba
fruits	mixed	Cuba
herbaceous	mixed	Cuba
tuberous	mixed	Cuba
herbaceous	mixed	Cuba
herbaceous	mixed	Cuba
tuberous	mixed	Cuba
fruits	mixed	Cuba
tuberous	mixed	Cuba
herbaceous	mixed	Cuba
tuberous	mixed	Cuba
herbaceous	mixed	Cuba
herbaceous	cooked	Cuba
herbaceous	mixed	Cuba

herbaceous	raw	Cuba
herbaceous	mixed	Cuba
herbaceous	raw	Cuba
tuberous	mixed	Cuba
herbaceous	mixed	Cuba
tuberous	mixed	Cuba
fruits	mixed	Cuba
herbaceous	mixed	Cuba
tuberous	mixed	Cuba
herbaceous	mixed	Cuba
herbaceous	mixed	Cuba
tuberous	mixed	Cuba
fruits	mixed	Cuba
herbaceous	mixed	Cuba
herbaceous	mixed	Cuba
tuberous	mixed	Cuba
herbaceous	cooked	Cuba
herbaceous	mixed	Cuba
tuberous	mixed	Cuba
herbaceous	mixed	Cuba
herbaceous	raw	Cuba
herbaceous	raw	Brazil
herbaceous	raw	Brazil
herbaceous	raw	Brazil
herbaceous	raw	Brazil
herbaceous	raw	Brazil
herbaceous	raw	Brazil
herbaceous	mixed	Brazil
herbaceous	mixed	Brazil
herbaceous	raw	Brazil
herbaceous	mixed	Brazil
herbaceous	mixed	Brazil
herbaceous	mixed	Brazil
herbaceous	raw	Brazil
herbaceous	mixed	Brazil
herbaceous	raw	Brazil
herbaceous	raw	Brazil
herbaceous	raw	Brazil
herbaceous	raw	Brazil
herbaceous	raw	Brazil
herbaceous	raw	Brazil
herbaceous	mixed	Thailand
herbaceous	mixed	Thailand
tuberous	cooked	Thailand
tuberous	mixed	Thailand
herbaceous	mixed	Thailand
herbaceous	mixed	Thailand
herbaceous	mixed	Thailand
herbaceous	mixed	Thailand
herbaceous	raw	Thailand

herbaceous	mixed	Thailand
tuberous	cooked	Thailand
herbaceous	mixed	Thailand
herbaceous	raw	Thailand
herbaceous	mixed	Thailand
herbaceous	mixed	Thailand
tuberous	mixed	Thailand
herbaceous	mixed	Thailand
herbaceous	mixed	Thailand
herbaceous	mixed	Thailand
herbaceous	mixed	Thailand
herbaceous	raw	Thailand
tuberous	cooked	Thailand
herbaceous	mixed	Thailand
herbaceous	mixed	Thailand
herbaceous	mixed	Thailand
herbaceous	mixed	Thailand
herbaceous	mixed	Thailand
herbaceous	mixed	Thailand
tuberous	mixed	Thailand
herbaceous	mixed	Thailand
herbaceous	raw	Thailand
herbaceous	mixed	Thailand
tuberous	cooked	Thailand
herbaceous	mixed	Thailand
tuberous	mixed	Thailand
herbaceous	mixed	Thailand
herbaceous	mixed	Thailand
herbaceous	mixed	Thailand
herbaceous	mixed	Thailand
herbaceous	mixed	Thailand
herbaceous	mixed	Thailand
herbaceous	raw	Thailand
herbaceous	mixed	Thailand
herbaceous	mixed	Thailand
herbaceous	mixed	Thailand
tuberous	mixed	Thailand
tuberous	cooked	Thailand
herbaceous	mixed	Thailand
herbaceous	mixed	Thailand
herbaceous	mixed	Thailand
herbaceous	mixed	Nigeria
fruits	cooked	Nigeria
fruits	cooked	Nigeria
tuberous	mixed	Nigeria
herbaceous	mixed	Nigeria
fruits	mixed	Nigeria
herbaceous	mixed	Nigeria
fruits	cooked	Nigeria
herbaceous	mixed	Nigeria
fruits	cooked	Nigeria

tuberous	mixed	Nigeria
herbaceous	mixed	Nigeria
tuberous	mixed	Nigeria
herbaceous	mixed	Nigeria
herbaceous	mixed	Nigeria
fruits	cooked	Nigeria
tuberous	mixed	Nigeria
herbaceous	mixed	Nigeria
herbaceous	mixed	Nigeria
fruits	mixed	Nigeria
inconclusive	mixed	Iraq
inconclusive	mixed	Iraq
inconclusive	mixed	Iraq
inconclusive	mixed	Iraq
inconclusive	mixed	Iraq
inconclusive	mixed	Iraq
inconclusive	mixed	Iraq
inconclusive	mixed	Iraq
herbaceous	mixed	Brazil
herbaceous	mixed	Brazil
herbaceous	mixed	Brazil
herbaceous	raw	Brazil
herbaceous	mixed	Brazil
herbaceous	mixed	Brazil
herbaceous	raw	Brazil
herbaceous	mixed	Brazil
inconclusive	mixed	Norway
herbaceous	mixed	Norway
herbaceous	mixed	Norway
fruits	raw	Norway
inconclusive	mixed	Norway
herbaceous	raw	Norway
fruits	raw	Norway
inconclusive	mixed	Norway
herbaceous	raw	Norway
inconclusive	mixed	Norway
herbaceous	mixed	Norway
herbaceous	mixed	Norway
fruits	raw	Norway
inconclusive	mixed	Norway
herbaceous	raw	Norway
fruits	raw	Norway
herbaceous	raw	Norway
inconclusive	mixed	Norway
inconclusive	mixed	Norway
herbaceous	mixed	Norway
herbaceous	mixed	Norway
fruits	raw	Norway
herbaceous	raw	Norway
herbaceous	raw	Norway
inconclusive	mixed	Norway
fruits	raw	Norway
herbaceous	mixed	Norway
fruits	raw	Norway
inconclusive	mixed	Norway
herbaceous	raw	Norway

inconclusive	mixed	Norway
herbaceous	mixed	Norway
fruits	raw	Norway
herbaceous	raw	Norway
inconclusive	mixed	Norway
herbaceous	raw	Brazil
herbaceous	raw	Brazil
herbaceous	raw	Brazil
herbaceous	raw	Brazil
herbaceous	raw	Brazil
herbaceous	raw	Brazil
herbaceous	raw	Brazil
herbaceous	raw	Brazil
herbaceous	raw	Brazil
herbaceous	raw	Brazil
herbaceous	raw	Brazil
herbaceous	raw	Brazil
herbaceous	raw	Brazil
herbaceous	raw	Brazil
herbaceous	raw	Brazil
herbaceous	raw	Brazil
herbaceous	raw	Brazil
herbaceous	raw	Brazil
herbaceous	raw	Brazil
herbaceous	mixed	Poland
herbaceous	mixed	Poland
herbaceous	mixed	Poland
herbaceous	mixed	Poland
herbaceous	mixed	Poland
tuberous	mixed	Poland
fruits	cooked	Vietnam
tuberous	cooked	Vietnam
tuberous	raw	Vietnam
fruits	mixed	Vietnam
tuberous	mixed	Vietnam
fruits	mixed	Vietnam
herbaceous	mixed	Vietnam
herbaceous	raw	Vietnam
herbaceous	mixed	Vietnam
herbaceous	mixed	Vietnam
herbaceous	mixed	Vietnam
tuberous	mixed	Vietnam
herbaceous	mixed	Vietnam
herbaceous	cooked	Vietnam
tuberous	cooked	Vietnam
tuberous	raw	Vietnam
fruits	mixed	Vietnam
herbaceous	mixed	Vietnam
fruits	cooked	Vietnam
tuberous	cooked	Vietnam
fruits	mixed	Vietnam
herbaceous	mixed	Vietnam
tuberous	mixed	Vietnam
herbaceous	mixed	Vietnam

tuberous	mixed	Vietnam
herbaceous	raw	Vietnam
herbaceous	mixed	Vietnam
herbaceous	mixed	Vietnam
herbaceous	cooked	Vietnam
tuberous	cooked	Vietnam
herbaceous	mixed	Vietnam
fruits	cooked	Vietnam
tuberous	cooked	Vietnam
tuberous	raw	Vietnam
tuberous	mixed	Vietnam
fruits	mixed	Vietnam
tuberous	mixed	Vietnam
fruits	mixed	Vietnam
herbaceous	mixed	Vietnam
herbaceous	mixed	Vietnam
herbaceous	raw	Vietnam
herbaceous	mixed	Vietnam
herbaceous	mixed	Vietnam
herbaceous	cooked	Vietnam
tuberous	cooked	Vietnam
herbaceous	mixed	Vietnam
fruits	cooked	Vietnam
tuberous	cooked	Vietnam
tuberous	raw	Vietnam
fruits	mixed	Vietnam
tuberous	mixed	Vietnam
fruits	mixed	Vietnam
herbaceous	raw	Vietnam
herbaceous	mixed	Vietnam
herbaceous	mixed	Vietnam
herbaceous	mixed	Vietnam
herbaceous	mixed	Vietnam
tuberous	mixed	Vietnam
herbaceous	cooked	Vietnam
tuberous	cooked	Vietnam
herbaceous	mixed	Vietnam
fruits	cooked	Vietnam
tuberous	cooked	Vietnam
tuberous	raw	Vietnam
fruits	mixed	Vietnam
tuberous	mixed	Vietnam
fruits	mixed	Vietnam
herbaceous	mixed	Vietnam
herbaceous	mixed	Vietnam
tuberous	mixed	Vietnam
herbaceous	mixed	Vietnam
herbaceous	raw	Vietnam
herbaceous	mixed	Vietnam
tuberous	cooked	Vietnam
herbaceous	cooked	Vietnam

inconclusive	mixed	Nigeria
inconclusive	mixed	Nigeria
inconclusive	mixed	Nigeria
inconclusive	mixed	Nigeria
inconclusive	mixed	Nigeria
inconclusive	mixed	Nigeria
inconclusive	mixed	Nigeria
herbaceous	mixed	Iran
tuberous	mixed	Iran
herbaceous	mixed	Iran
herbaceous	mixed	Iran
herbaceous	mixed	Iran
herbaceous	mixed	Iran
herbaceous	cooked	Iran
herbaceous	raw	Iran
herbaceous	mixed	Iran
herbaceous	mixed	Iran
herbaceous	mixed	Iran
tuberous	mixed	Iran
tuberous	mixed	Iran
herbaceous	mixed	Iran
tuberous	mixed	Iran
tuberous	mixed	Iran
herbaceous	cooked	Iran
herbaceous	mixed	Iran
tuberous	mixed	Iran
herbaceous	mixed	Iran
herbaceous	mixed	Iran
herbaceous	mixed	Iran
herbaceous	raw	Iran
herbaceous	mixed	Iran
tuberous	mixed	Iran
fruits	mixed	Pakistan
fruits	mixed	Pakistan
tuberous	mixed	Pakistan
herbaceous	mixed	Pakistan
fruits	mixed	Pakistan
tuberous	mixed	Pakistan
tuberous	mixed	Pakistan
herbaceous	raw	Pakistan
herbaceous	mixed	Pakistan
herbaceous	mixed	Pakistan
herbaceous	mixed	Pakistan
tuberous	mixed	Pakistan
tuberous	mixed	Pakistan
herbaceous	mixed	Pakistan
fruits	mixed	Pakistan
tuberous	mixed	Pakistan
fruits	mixed	Pakistan
herbaceous	raw	Pakistan
fruits	mixed	Pakistan
herbaceous	mixed	Pakistan
herbaceous	raw	Pakistan

herbaceous	mixed	Pakistan
tuberous	mixed	Pakistan
fruits	mixed	Pakistan
fruits	mixed	Pakistan
tuberous	mixed	Pakistan
herbaceous	mixed	Pakistan
tuberous	mixed	Pakistan
herbaceous	mixed	Pakistan
fruits	mixed	Pakistan
herbaceous	raw	Pakistan
herbaceous	mixed	Pakistan
fruits	mixed	Pakistan
tuberous	mixed	Pakistan
tuberous	mixed	Pakistan
tuberous	mixed	Pakistan
herbaceous	mixed	Pakistan
fruits	mixed	Pakistan
herbaceous	mixed	Pakistan
fruits	mixed	Pakistan
herbaceous	mixed	Pakistan
fruits	mixed	Pakistan
fruits	mixed	Pakistan
fruits	mixed	Pakistan
herbaceous	raw	Pakistan
tuberous	mixed	Pakistan
tuberous	mixed	Pakistan
tuberous	mixed	Pakistan
herbaceous	mixed	Pakistan
herbaceous	mixed	Pakistan
tuberous	mixed	Pakistan
tuberous	mixed	Pakistan
herbaceous	mixed	Pakistan
fruits	mixed	Pakistan
fruits	mixed	Pakistan
fruits	mixed	Pakistan
tuberous	mixed	Pakistan
herbaceous	raw	Pakistan
herbaceous	mixed	Pakistan
herbaceous	mixed	Pakistan
herbaceous	raw	Pakistan
herbaceous	mixed	Pakistan
tuberous	mixed	Pakistan
fruits	mixed	Pakistan
tuberous	mixed	Pakistan
herbaceous	mixed	Pakistan
fruits	mixed	Pakistan
tuberous	mixed	Pakistan
herbaceous	mixed	Pakistan
fruits	mixed	Pakistan
fruits	mixed	Pakistan
fruits	mixed	Pakistan

herbaceous	raw	Pakistan
herbaceous	mixed	Pakistan
tuberous	mixed	Pakistan
tuberous	mixed	Pakistan
herbaceous	mixed	Pakistan
fruits	mixed	Pakistan
tuberous	mixed	Pakistan
herbaceous	mixed	Pakistan
herbaceous	mixed	Pakistan
fruits	mixed	Pakistan
fruits	mixed	Pakistan
tuberous	mixed	Pakistan
fruits	mixed	Pakistan
tuberous	mixed	Pakistan
tuberous	mixed	Pakistan
herbaceous	mixed	Pakistan
herbaceous	mixed	Pakistan
herbaceous	raw	Pakistan
herbaceous	mixed	Pakistan
tuberous	mixed	Pakistan
tuberous	mixed	Pakistan
fruits	mixed	Pakistan
fruits	mixed	Pakistan
herbaceous	mixed	Pakistan
fruits	mixed	Pakistan
herbaceous	raw	Pakistan
herbaceous	mixed	Pakistan
tuberous	mixed	Pakistan
herbaceous	raw	Pakistan
fruits	mixed	Pakistan
herbaceous	mixed	Pakistan
fruits	mixed	Pakistan
herbaceous	mixed	Pakistan
tuberous	mixed	Pakistan
tuberous	mixed	Pakistan
fruits	mixed	Pakistan
tuberous	mixed	Pakistan
herbaceous	mixed	Pakistan
tuberous	mixed	Pakistan
herbaceous	mixed	Pakistan
fruits	mixed	Pakistan
tuberous	mixed	Pakistan
tuberous	mixed	Pakistan
fruits	mixed	Pakistan
fruits	mixed	Pakistan
herbaceous	mixed	Pakistan
herbaceous	mixed	Pakistan
herbaceous	raw	Pakistan
herbaceous	mixed	Iran
herbaceous	mixed	Iran
herbaceous	mixed	Iran
herbaceous	mixed	Iran

herbaceous	mixed	Iran
herbaceous	raw	Iran
herbaceous	mixed	Iran
tuberous	mixed	Iran
herbaceous	mixed	Iran
tuberous	mixed	Iran
herbaceous	mixed	Iran
herbaceous	mixed	Iran
herbaceous	mixed	Iran
herbaceous	raw	Iran
herbaceous	mixed	Iran
tuberous	mixed	Iran
tuberous	mixed	Iran
herbaceous	mixed	Iran
herbaceous	mixed	Iran
herbaceous	mixed	Iran
herbaceous	mixed	Iran
herbaceous	mixed	Iran
herbaceous	mixed	Iran
herbaceous	raw	Iran
tuberous	mixed	Iran
tuberous	mixed	Iran
herbaceous	mixed	Iran
herbaceous	mixed	Iran
herbaceous	mixed	Iran
herbaceous	mixed	Iran
herbaceous	mixed	Iran
herbaceous	mixed	Iran
herbaceous	mixed	Iran
herbaceous	mixed	Iran
herbaceous	mixed	Iran
herbaceous	raw	Iran
herbaceous	mixed	Iran
tuberous	mixed	Iran
herbaceous	mixed	Iran
tuberous	mixed	Iran
tuberous	mixed	Iran
herbaceous	mixed	Iran
herbaceous	mixed	Iran
herbaceous	mixed	Iran
herbaceous	mixed	Iran
herbaceous	raw	Iran
herbaceous	mixed	Iran
tuberous	mixed	Iran
herbaceous	mixed	Iran
herbaceous	mixed	Iran
herbaceous	mixed	Iran
herbaceous	mixed	Iran
herbaceous	mixed	Iran
herbaceous	mixed	Iran
herbaceous	mixed	Iran

herbaceous	mixed	Iran
herbaceous	raw	Iran
herbaceous	mixed	Iran
tuberous	mixed	Iran
tuberous	mixed	Iran
herbaceous	mixed	Iran
herbaceous	mixed	Iran
herbaceous	mixed	Iran
herbaceous	mixed	Iran
herbaceous	mixed	Iran
herbaceous	mixed	Iran
tuberous	mixed	Iran
tuberous	mixed	Iran
herbaceous	mixed	Iran
herbaceous	raw	Iran
herbaceous	mixed	Iran
herbaceous	mixed	Iran
herbaceous	mixed	Iran
herbaceous	mixed	Iran
herbaceous	raw	Iran
herbaceous	mixed	Iran
tuberous	mixed	Iran
herbaceous	mixed	Iran
herbaceous	mixed	Iran
tuberous	mixed	Iran
herbaceous	mixed	Iran
herbaceous	mixed	Iran
herbaceous	mixed	Iran
herbaceous	mixed	Iran
herbaceous	mixed	Iran
herbaceous	mixed	Iran
tuberous	mixed	Iran
tuberous	mixed	Iran
herbaceous	mixed	Iran
herbaceous	raw	Iran
herbaceous	raw	Brazil
herbaceous	raw	Brazil
herbaceous	raw	Brazil
herbaceous	raw	Brazil
herbaceous	raw	Brazil
herbaceous	raw	Brazil
herbaceous	raw	Brazil
herbaceous	raw	Brazil
herbaceous	raw	Brazil
herbaceous	raw	Brazil
herbaceous	raw	Brazil
herbaceous	raw	Brazil
inconclusive	mixed	Korea
fruits	mixed	Korea
herbaceous	mixed	Korea
herbaceous	mixed	Korea
fruits	mixed	Korea
herbaceous	mixed	Korea
fruits	mixed	Korea
inconclusive	mixed	Korea

fruits	mixed	Korea
herbaceous	mixed	Korea
herbaceous	mixed	Brazil
herbaceous	mixed	Brazil
herbaceous	mixed	Brazil
herbaceous	raw	Brazil
herbaceous	mixed	Brazil
herbaceous	mixed	Brazil
herbaceous	mixed	Brazil
herbaceous	raw	Brazil
herbaceous	mixed	Brazil
herbaceous	mixed	Brazil
herbaceous	mixed	Brazil
herbaceous	mixed	Brazil
herbaceous	raw	Brazil
herbaceous	raw	Brazil
herbaceous	raw	Brazil
herbaceous	raw	Brazil
herbaceous	mixed	Brazil
herbaceous	mixed	Brazil
herbaceous	raw	Brazil
herbaceous	raw	Brazil
herbaceous	mixed	Brazil
herbaceous	mixed	Brazil
herbaceous	mixed	Brazil
herbaceous	mixed	Brazil
herbaceous	mixed	Brazil
herbaceous	mixed	Brazil
herbaceous	mixed	Brazil
herbaceous	mixed	Brazil
herbaceous	raw	Brazil
herbaceous	raw	Brazil
herbaceous	raw	Brazil
herbaceous	raw	Brazil
herbaceous	mixed	Iran
herbaceous	mixed	Iran
herbaceous	mixed	Iran
herbaceous	mixed	Iran
herbaceous	mixed	Iran
tuberous	mixed	Iran
herbaceous	cooked	Iran
herbaceous	raw	Iran
herbaceous	mixed	Iran
herbaceous	mixed	Iran
herbaceous	mixed	Iran
herbaceous	mixed	Iran
herbaceous	cooked	Iran
herbaceous	raw	Iran
herbaceous	mixed	Iran
tuberous	mixed	Iran
herbaceous	mixed	Iran

herbaceous	mixed	Iran
herbaceous	mixed	Iran
herbaceous	mixed	Iran
herbaceous	raw	Iran
herbaceous	mixed	Iran
herbaceous	mixed	Iran
herbaceous	mixed	Iran
herbaceous	mixed	Iran
herbaceous	mixed	Iran
herbaceous	mixed	Iran
herbaceous	cooked	Iran
tuberous	mixed	Iran
herbaceous	mixed	Iran
herbaceous	raw	Iran
herbaceous	mixed	Iran
herbaceous	mixed	Iran
herbaceous	mixed	Iran
herbaceous	mixed	Iran
tuberous	mixed	Iran
herbaceous	cooked	Iran
herbaceous	mixed	Iran
herbaceous	mixed	Iran
herbaceous	mixed	Iran
herbaceous	mixed	Iran
herbaceous	mixed	Iran
herbaceous	mixed	Iran
herbaceous	mixed	Iran
herbaceous	mixed	Iran
herbaceous	mixed	Iran
herbaceous	mixed	Iran
herbaceous	cooked	Iran
herbaceous	raw	Iran
tuberous	mixed	Iran
herbaceous	cooked	Iran
herbaceous	mixed	Iran
herbaceous	mixed	Iran
herbaceous	mixed	Iran
herbaceous	mixed	Iran
herbaceous	mixed	Iran
herbaceous	mixed	Iran
tuberous	mixed	Iran
herbaceous	raw	Iran
herbaceous	mixed	Iran
herbaceous	mixed	Iran
herbaceous	mixed	Iran
herbaceous	mixed	Iran
herbaceous	mixed	Iran
tuberous	mixed	Iran
herbaceous	cooked	Iran
herbaceous	raw	Iran
herbaceous	mixed	Iran
herbaceous	mixed	Iran
herbaceous	mixed	Iran
herbaceous	mixed	Iran

herbaceous	mixed	Iran
herbaceous	mixed	Iran
herbaceous	mixed	Iran
herbaceous	mixed	Iran
herbaceous	mixed	Iran
herbaceous	raw	Iran
tuberous	mixed	Iran
herbaceous	mixed	Iran
herbaceous	cooked	Iran
herbaceous	mixed	Iran
herbaceous	mixed	Iran
herbaceous	mixed	Iran
herbaceous	mixed	Iran
herbaceous	mixed	Iran
herbaceous	mixed	Iran
tuberous	mixed	Iran
herbaceous	cooked	Iran
herbaceous	raw	Iran
herbaceous	mixed	Iran
herbaceous	cooked	Iran
herbaceous	mixed	Iran
herbaceous	mixed	Iran
herbaceous	mixed	Iran
herbaceous	mixed	Iran
herbaceous	mixed	Iran
herbaceous	mixed	Iran
herbaceous	raw	Iran
herbaceous	mixed	Iran
tuberous	mixed	Iran
herbaceous	raw	Iran
herbaceous	mixed	Iran
herbaceous	mixed	Iran
herbaceous	mixed	Iran
herbaceous	mixed	Iran
herbaceous	mixed	Iran
herbaceous	mixed	Iran
tuberous	mixed	Iran
herbaceous	mixed	Iran
herbaceous	cooked	Iran
herbaceous	mixed	Brazil
herbaceous	raw	Brazil
herbaceous	mixed	Brazil
herbaceous	mixed	Brazil
herbaceous	raw	Brazil
herbaceous	mixed	Brazil
herbaceous	mixed	Brazil
herbaceous	raw	Brazil
herbaceous	mixed	Brazil
herbaceous	mixed	Brazil
herbaceous	raw	Brazil

herbaceous	mixed	Brazil
herbaceous	mixed	Brazil
herbaceous	mixed	Brazil
herbaceous	raw	Brazil
herbaceous	mixed	Brazil
herbaceous	mixed	Brazil
herbaceous	raw	Brazil
herbaceous	mixed	Brazil
herbaceous	raw	Brazil
herbaceous	mixed	Brazil
herbaceous	mixed	Brazil
herbaceous	raw	Brazil
herbaceous	mixed	Brazil
herbaceous	mixed	Brazil
herbaceous	raw	Brazil
herbaceous	mixed	Brazil
herbaceous	raw	Brazil
herbaceous	mixed	Brazil
herbaceous	mixed	Brazil
herbaceous	mixed	Brazil
herbaceous	raw	Brazil
herbaceous	mixed	Brazil
herbaceous	raw	Brazil
herbaceous	mixed	Brazil
herbaceous	mixed	Brazil
fruits	cooked	Eritrea
tuberous	mixed	Eritrea
fruits	mixed	Eritrea
herbaceous	mixed	Eritrea
herbaceous	mixed	Eritrea
herbaceous	raw	Eritrea
herbaceous	cooked	Thailand
herbaceous	mixed	Thailand
herbaceous	mixed	Thailand
herbaceous	mixed	Thailand
tuberous	mixed	Thailand
herbaceous	mixed	Thailand
herbaceous	mixed	Thailand
herbaceous	mixed	Thailand
herbaceous	mixed	Thailand
herbaceous	raw	Thailand
herbaceous	mixed	Thailand
herbaceous	raw	Thailand
herbaceous	mixed	Thailand
tuberous	mixed	Thailand
herbaceous	mixed	Thailand
herbaceous	raw	ITALIA
herbaceous	raw	ITALIA
herbaceous	raw	ITALIA
herbaceous	raw	ITALIA
herbaceous	raw	ITALIA
herbaceous	raw	ITALIA

fruits	cooked	Poland
fruits	cooked	Poland
herbaceous	raw	Poland
herbaceous	mixed	Poland
herbaceous	mixed	Poland
herbaceous	mixed	Poland
herbaceous	mixed	Poland
herbaceous	mixed	Poland
tuberous	mixed	Poland
herbaceous	mixed	Poland
fruits	cooked	Poland
herbaceous	raw	Poland
herbaceous	mixed	Poland
tuberous	cooked	Poland
herbaceous	mixed	Poland
herbaceous	mixed	Poland
herbaceous	mixed	Poland
fruits	raw	Poland
herbaceous	mixed	Poland
tuberous	mixed	Poland
herbaceous	mixed	Poland
tuberous	mixed	Poland
tuberous	mixed	Poland
tuberous	mixed	Poland
tuberous	mixed	Poland
fruits	raw	Poland
herbaceous	cooked	Poland
tuberous	cooked	Poland
herbaceous	mixed	Poland
tuberous	mixed	Poland
herbaceous	mixed	Poland
fruits	cooked	Poland
tuberous	mixed	Poland
tuberous	mixed	Poland
tuberous	mixed	Poland
tuberous	mixed	Poland
tuberous	mixed	Poland
herbaceous	mixed	Poland
tuberous	mixed	Poland
herbaceous	mixed	Poland
tuberous	mixed	Poland
inconclusive	mixed	Vietnam
inconclusive	mixed	Vietnam
inconclusive	mixed	Iran
inconclusive	mixed	Iran
inconclusive	mixed	Iran
inconclusive	mixed	Iran
inconclusive	mixed	Iran
inconclusive	mixed	Iran
inconclusive	mixed	Iran

inconclusive	mixed	Iran
inconclusive	mixed	Iran
herbaceous	mixed	Brazil
herbaceous	mixed	Brazil
herbaceous	mixed	Brazil
herbaceous	mixed	Brazil
herbaceous	mixed	Brazil
herbaceous	mixed	Brazil
herbaceous	raw	Brazil
herbaceous	mixed	Vietnam
herbaceous	mixed	Vietnam
herbaceous	raw	Vietnam
herbaceous	mixed	Vietnam
herbaceous	mixed	Vietnam
herbaceous	mixed	Vietnam
herbaceous	raw	Vietnam
herbaceous	mixed	Vietnam
herbaceous	mixed	Vietnam
herbaceous	mixed	Vietnam
inconclusive	mixed	India
inconclusive	mixed	India
herbaceous	mixed	Czech
tuberous	mixed	Czech
fruits	mixed	Czech
herbaceous	raw	Czech
fruits	mixed	Czech
tuberous	mixed	Czech
herbaceous	raw	Czech
herbaceous	mixed	Czech
herbaceous	mixed	Czech
fruits	mixed	Czech
herbaceous	raw	Czech
tuberous	mixed	Czech
fruits	mixed	Czech
tuberous	mixed	Czech
herbaceous	raw	Czech
herbaceous	mixed	Czech
fruits	raw	Nigeria
herbaceous	cooked	Nigeria
fruits	raw	Nigeria
fruits	raw	Nigeria
tuberous	mixed	Nigeria
fruits	mixed	Nigeria
herbaceous	raw	Nigeria
herbaceous	mixed	Nigeria
fruits	raw	Nigeria
tuberous	mixed	Nigeria
fruits	raw	Nigeria
herbaceous	cooked	Nigeria
tuberous	mixed	Nigeria
fruits	raw	Nigeria
fruits	raw	Nigeria

tuberous	mixed	Nigeria
herbaceous	mixed	Nigeria
fruits	raw	Nigeria
fruits	mixed	Nigeria
herbaceous	raw	Nigeria
fruits	raw	Nigeria
fruits	raw	Nigeria
herbaceous	cooked	Nigeria
fruits	raw	Nigeria
tuberous	mixed	Nigeria
fruits	raw	Nigeria
fruits	mixed	Nigeria
tuberous	mixed	Nigeria
herbaceous	mixed	Nigeria
herbaceous	raw	Nigeria
fruits	raw	Nigeria
fruits	raw	Nigeria
tuberous	mixed	Nigeria
herbaceous	raw	Nigeria
herbaceous	mixed	Nigeria
fruits	raw	Nigeria
tuberous	mixed	Nigeria
herbaceous	cooked	Nigeria
fruits	raw	Nigeria
fruits	mixed	Nigeria
fruits	raw	Nigeria
herbaceous	cooked	Nigeria
herbaceous	raw	Nigeria
herbaceous	mixed	Nigeria
fruits	raw	Nigeria
fruits	mixed	Nigeria
tuberous	mixed	Nigeria
fruits	raw	Nigeria
fruits	raw	Nigeria
tuberous	mixed	Nigeria
fruits	raw	Nigeria
fruits	raw	Nigeria
herbaceous	cooked	Nigeria
fruits	raw	Nigeria
herbaceous	raw	Nigeria
fruits	mixed	Nigeria
tuberous	mixed	Nigeria
fruits	raw	Nigeria
tuberous	mixed	Nigeria
herbaceous	mixed	Nigeria
fruits	raw	Nigeria
fruits	raw	Nigeria
herbaceous	cooked	Nigeria
fruits	raw	Nigeria
fruits	mixed	Nigeria

tuberous	mixed	Nigeria
herbaceous	mixed	Nigeria
fruits	raw	Nigeria
tuberous	mixed	Nigeria
herbaceous	raw	Nigeria
tuberous	mixed	Pakistan
fruits	mixed	Pakistan
tuberous	mixed	Pakistan
fruits	cooked	Pakistan
fruits	mixed	Pakistan
tuberous	mixed	Pakistan
herbaceous	mixed	Pakistan
fruits	mixed	Pakistan
tuberous	cooked	Pakistan
tuberous	cooked	Pakistan
herbaceous	cooked	Pakistan
tuberous	mixed	Pakistan
herbaceous	mixed	Pakistan
tuberous	mixed	Pakistan
herbaceous	mixed	Pakistan
fruits	mixed	Pakistan
tuberous	mixed	Pakistan
herbaceous	cooked	Pakistan
herbaceous	mixed	Pakistan
fruits	mixed	Pakistan
fruits	cooked	Pakistan
tuberous	mixed	Pakistan
tuberous	cooked	Pakistan
tuberous	mixed	Pakistan
fruits	mixed	Pakistan
tuberous	cooked	Pakistan
fruits	mixed	Pakistan
tuberous	cooked	Pakistan
fruits	mixed	Pakistan
tuberous	mixed	Pakistan
herbaceous	cooked	Pakistan
herbaceous	mixed	Pakistan
tuberous	mixed	Pakistan
fruits	cooked	Pakistan
tuberous	mixed	Pakistan
tuberous	mixed	Pakistan
fruits	mixed	Pakistan
herbaceous	mixed	Pakistan
tuberous	cooked	Pakistan
tuberous	cooked	Pakistan
fruits	mixed	Pakistan
tuberous	mixed	Pakistan
tuberous	cooked	Pakistan
tuberous	mixed	Pakistan
herbaceous	cooked	Pakistan

fruits	mixed	Pakistan
tuberous	mixed	Pakistan
tuberous	mixed	Pakistan
herbaceous	mixed	Pakistan
fruits	mixed	Pakistan
herbaceous	mixed	Pakistan
fruits	cooked	Pakistan
inconclusive	cooked	China
herbaceous	mixed	China
herbaceous	mixed	China
herbaceous	mixed	China
herbaceous	raw	China
herbaceous	raw	China
herbaceous	mixed	China
herbaceous	cooked	China
herbaceous	mixed	China
herbaceous	raw	China
herbaceous	mixed	China
herbaceous	mixed	China
herbaceous	mixed	China
herbaceous	raw	China
herbaceous	mixed	China
herbaceous	mixed	China
herbaceous	cooked	China
herbaceous	mixed	China
herbaceous	raw	China
fruits	mixed	Jordan
fruits	mixed	Jordan
fruits	mixed	Jordan
fruits	mixed	Jordan
herbaceous	mixed	Jordan
herbaceous	mixed	Jordan
herbaceous	raw	Jordan
herbaceous	raw	Jordan
fruits	mixed	Jordan
fruits	mixed	Jordan
fruits	mixed	Jordan
herbaceous	mixed	Jordan
herbaceous	raw	Jordan
herbaceous	mixed	Jordan
fruits	mixed	Jordan
herbaceous	raw	Jordan
herbaceous	raw	Jordan
herbaceous	raw	Jordan
fruits	mixed	Jordan
fruits	mixed	Jordan
fruits	mixed	Jordan
fruits	mixed	Jordan
herbaceous	mixed	Jordan
herbaceous	mixed	Jordan

herbaceous	raw	Jordan
herbaceous	raw	Jordan
herbaceous	raw	Jordan
herbaceous	raw	Jordan
fruits	mixed	Jordan
herbaceous	raw	Jordan
herbaceous	raw	Jordan
fruits	mixed	Jordan
fruits	mixed	Jordan
herbaceous	raw	Jordan
fruits	mixed	Jordan
herbaceous	raw	Jordan
herbaceous	mixed	Jordan
herbaceous	mixed	Jordan
herbaceous	raw	Jordan
herbaceous	raw	Jordan
herbaceous	mixed	Jordan
herbaceous	raw	Jordan
herbaceous	mixed	Jordan
herbaceous	raw	Jordan
herbaceous	raw	Jordan
herbaceous	raw	Jordan
fruits	mixed	Jordan
herbaceous	raw	Jordan
fruits	mixed	Jordan
herbaceous	raw	Jordan
inconclusive	mixed	Iran
inconclusive	mixed	Iran
inconclusive	mixed	Iran
inconclusive	mixed	Iran
inconclusive	mixed	Iran
inconclusive	mixed	Iran
inconclusive	mixed	Iran
inconclusive	mixed	Iran
inconclusive	mixed	Iran
inconclusive	mixed	Iran

HDI	TOTAL SAMPLES	POSITIVE SAMPLES	FOOD ORIGIN
high	30	6	vegetable gardens
high	30	6	vegetable gardens
high	30	7	vegetable gardens
high	30	8	vegetable gardens
high	30	9	vegetable gardens
high	30	9	vegetable gardens
high	32	11	vegetable gardens
high	30	14	vegetable gardens
high	33	16	vegetable gardens
high	96	2	markets
high	45	3	vegetable gardens
high	96	6	markets
high	45	8	vegetable gardens
high	96	10	markets
high	45	2	vegetable gardens
high	96	5	markets
high	45	4	vegetable gardens
high	96	7	markets
high	45	2	vegetable gardens
high	96	1	markets
high	45	7	vegetable gardens
high	96	13	markets
high	96	1	markets
high	29	0	vegetable gardens
high	35	0	vegetable gardens
high	35	0	vegetable gardens
high	38	0	vegetable gardens
high	40	0	vegetable gardens
high	40	0	vegetable gardens
high	41	0	vegetable gardens
high	39	1	vegetable gardens
high	42	1	vegetable gardens
high	31	3	vegetable gardens
high	37	0	markets
high	39	0	markets
high	39	0	markets
high	59	3	markets
high	51	4	markets
high	61	5	markets
high	50	6	markets
high	70	7	markets
high	41	9	markets
high	35	0	vegetable gardens
high	39	0	vegetable gardens
high	40	0	vegetable gardens
high	40	0	vegetable gardens
high	35	1	vegetable gardens
high	41	1	vegetable gardens
high	59	1	markets

high	70	2	markets
high	61	3	markets
high	29	0	markets
high	31	0	markets
high	37	0	markets
high	38	0	markets
high	39	0	markets
high	41	1	markets
high	42	1	markets
high	51	2	markets
high	50	4	markets
high	39	5	markets
high	29	0	markets
high	37	0	markets
high	38	0	markets
high	41	0	markets
high	31	1	markets
high	39	1	markets
high	42	2	markets
high	50	2	markets
high	51	2	markets
high	39	3	markets
high	35	0	vegetable gardens
high	35	0	vegetable gardens
high	39	0	vegetable gardens
high	40	0	vegetable gardens
high	40	0	vegetable gardens
high	41	0	vegetable gardens
high	29	0	markets
high	37	0	markets
high	41	0	markets
high	61	0	markets
high	31	1	markets
high	38	1	markets
high	51	1	markets
high	39	2	markets
high	42	2	markets
high	50	2	markets
high	59	2	markets
high	39	3	markets
high	70	3	markets
high	29	0	markets
high	31	0	markets
high	37	0	markets
high	38	0	markets
high	39	0	markets
high	41	0	markets
high	42	0	markets
high	50	1	markets
high	39	2	markets
high	51	2	markets
high	29	0	markets
high	31	0	markets
high	37	0	markets
high	38	0	markets
high	42	1	markets
high	39	2	markets
high	41	3	markets
high	50	3	markets

high	39	6	markets
high	51	7	markets
high	31	0	markets
high	37	0	markets
high	38	0	markets
high	41	0	markets
high	39	2	markets
high	42	2	markets
high	51	2	markets
high	29	3	markets
high	39	4	markets
high	50	5	markets
high	3	2	vegetable gardens
high	3	2	vegetable gardens
high	3	0	vegetable gardens
high	3	0	vegetable gardens
high	3	0	vegetable gardens
high	3	2	vegetable gardens
high	3	2	vegetable gardens
high	3	1	vegetable gardens
high	3	1	vegetable gardens
high	3	1	vegetable gardens
high	3	2	vegetable gardens
high	3	2	vegetable gardens
medium	1031	0	markets
medium	1031	1	markets
medium	1030	3	markets
medium	1032	4	markets
medium	1034	5	markets
medium	1035	8	markets
medium	1036	9	markets
medium	1030	12	markets
medium	1031	14	markets
medium	1034	14	markets
medium	1035	17	markets
medium	1030	18	markets
medium	1031	2	markets
medium	1030	4	markets
medium	1034	4	markets
medium	1031	6	markets
medium	1032	6	markets
medium	1035	6	markets
medium	1036	9	markets
medium	1030	10	markets
medium	1030	12	markets
medium	1035	12	markets
medium	1031	18	markets
medium	1034	18	markets
medium	1031	10	markets
medium	1034	10	markets
medium	1030	12	markets
medium	1031	12	markets
medium	1032	13	markets
medium	1036	14	markets
medium	1030	15	markets
medium	1031	15	markets
medium	1034	15	markets
medium	1035	15	markets
medium	1030	18	markets
medium	1035	18	markets

medium	1031	2	markets
medium	1032	3	markets
medium	1034	5	markets
medium	1034	5	markets
medium	1031	6	markets
medium	1036	6	markets
medium	1031	7	markets
medium	1030	8	markets
medium	1030	8	markets
medium	1035	8	markets
medium	1035	8	markets
medium	1030	9	markets
medium	1031	4	markets
medium	1032	5	markets
medium	1030	6	markets
medium	1031	6	markets
medium	1034	7	markets
medium	1035	7	markets
medium	1036	8	markets
medium	1030	9	markets
medium	1030	9	markets
medium	1034	9	markets
medium	1031	10	markets
medium	1035	10	markets
medium	1030	4	markets
medium	1031	5	markets
medium	1036	5	markets
medium	1030	6	markets
medium	1031	6	markets
medium	1035	6	markets
medium	1032	7	markets
medium	1035	7	markets
medium	1030	8	markets
medium	1034	8	markets
medium	1031	9	markets
medium	1034	9	markets
medium	1030	6	markets
medium	1031	6	markets
medium	1031	6	markets
medium	1032	7	markets
medium	1034	7	markets
medium	1035	7	markets
medium	1034	8	markets
medium	1036	8	markets
medium	1030	9	markets
medium	1030	9	markets
medium	1031	9	markets
medium	1035	9	markets
medium	1031	11	markets
medium	1034	12	markets
medium	1031	13	markets
medium	1031	13	markets
medium	1032	13	markets

medium	1035	13	markets
medium	1030	14	markets
medium	1030	14	markets
medium	1030	14	markets
medium	1035	14	markets
medium	1036	14	markets
medium	1034	20	markets
medium	1031	17	markets
medium	1032	18	markets
medium	1034	18	markets
medium	1030	20	markets
medium	1031	20	markets
medium	1034	21	markets
medium	1035	21	markets
medium	1036	22	markets
medium	1030	23	markets
medium	1031	24	markets
medium	1035	24	markets
medium	1030	25	markets
medium	1031	5	markets
medium	1031	8	markets
medium	1034	8	markets
medium	1030	10	markets
medium	1032	11	markets
medium	1031	13	markets
medium	1035	13	markets
medium	1036	13	markets
medium	1030	14	markets
medium	1034	15	markets
medium	1035	15	markets
medium	1030	16	markets
medium	1030	19	markets
medium	1030	19	markets
medium	1034	19	markets
medium	1031	20	markets
medium	1032	20	markets
medium	1036	21	markets
medium	1030	22	markets
medium	1035	22	markets
medium	1031	23	markets
medium	1034	23	markets
medium	1035	25	markets
medium	1030	26	markets
medium	1031	2	markets
medium	1030	3	markets
medium	1031	4	markets
medium	1032	4	markets
medium	1034	7	markets
medium	1031	8	markets
medium	1034	8	markets
medium	1035	8	markets
medium	1036	12	markets

medium	1030	13	markets
medium	1035	13	markets
medium	1030	14	markets
medium	1031	1	markets
medium	1031	2	markets
medium	1030	3	markets
medium	1032	4	markets
medium	1034	4	markets
medium	1036	5	markets
medium	1035	6	markets
medium	1031	7	markets
medium	1030	8	markets
medium	1034	8	markets
medium	1035	9	markets
medium	1030	12	markets
medium	1030	0	markets
medium	1031	1	markets
medium	1031	1	markets
medium	1032	1	markets
medium	1035	1	markets
medium	1030	2	markets
medium	1034	2	markets
medium	1034	2	markets
medium	1031	4	markets
medium	1035	4	markets
medium	1036	4	markets
medium	1030	6	markets
medium	1030	2	markets
medium	1031	2	markets
medium	1031	2	markets
medium	1034	3	markets
medium	1032	4	markets
medium	1035	6	markets
medium	1036	6	markets
medium	1030	9	markets
medium	1034	10	markets
medium	1030	11	markets
medium	1031	11	markets
medium	1035	11	markets
medium	1031	0	markets
medium	1031	1	markets
medium	1030	2	markets
medium	1032	4	markets
medium	1034	4	markets
medium	1035	8	markets
medium	1036	11	markets
medium	1030	13	markets
medium	1034	15	markets
medium	1031	16	markets
medium	1030	18	markets
medium	1035	18	markets
high	98	1	markets

high	116	2	markets
high	98	3	markets
high	116	3	markets
high	116	5	markets
high	116	5	markets
high	112	7	markets
high	98	1	markets
high	98	2	markets
high	116	3	markets
high	112	4	markets
high	116	4	markets
high	116	4	markets
high	116	5	markets
high	98	1	markets
high	112	1	markets
high	98	2	markets
high	116	2	markets
high	116	3	markets
high	116	4	markets
high	116	4	markets
high	98	0	markets
high	116	0	markets
high	116	0	markets
high	98	1	markets
high	116	1	markets
high	116	2	markets
high	112	3	markets
high	116	0	markets
high	116	1	markets
high	98	2	markets
high	112	2	markets
high	116	2	markets
high	116	2	markets
high	98	4	markets
high	116	0	markets
high	98	1	markets
high	98	1	markets
high	116	1	markets
high	116	2	markets
high	112	3	markets
high	116	4	markets
high	98	0	markets
high	98	1	markets
high	116	1	markets
high	112	2	markets
high	116	3	markets
high	116	4	markets
high	116	6	markets
very high	32	1	markets
very high	32	1	markets
very high	32	2	markets

very high	32	2	markets
very high	32	4	markets
very high	32	1	markets
very high	32	3	markets
very high	32	1	markets
very high	32	1	markets
very high	32	1	markets
very high	32	1	markets
very high	32	1	markets
very high	32	1	markets
very high	32	2	markets
low	20	0	markets
low	20	0	markets
low	20	6	markets
low	20	6	markets
low	20	7	markets
low	20	6	markets
low	20	8	markets
low	20	10	markets
low	20	12	markets
low	20	12	markets
low	20	0	markets
low	20	0	markets
low	20	0	markets
low	20	0	markets
low	20	0	markets
low	20	0	markets
low	20	1	markets
low	20	1	markets
low	20	1	markets
low	20	1	markets
low	20	0	markets
low	20	0	markets
low	20	0	markets
low	20	0	markets
low	20	0	markets
low	20	0	markets
low	20	0	markets
low	20	0	markets
low	20	0	markets
low	20	1	markets
low	20	0	markets
low	20	0	markets
low	20	0	markets
low	20	0	markets
low	20	0	markets
low	20	0	markets
low	20	0	markets
low	20	1	markets
low	20	1	markets

low	20	1	markets
high	8	3	markets
high	28	4	markets
high	6	5	markets
high	6	6	markets
high	8	7	markets
high	21	20	markets
high	28	20	markets
high	21	21	markets
high	6	0	markets
high	8	0	markets
high	6	1	markets
high	21	1	markets
high	28	1	markets
high	8	2	markets
high	21	2	markets
high	28	5	markets
high	8	0	markets
high	6	2	markets
high	6	2	markets
high	8	2	markets
high	28	2	markets
high	21	6	markets
high	21	7	markets
high	28	7	markets
high	8	0	markets
high	8	1	markets
high	28	1	markets
high	28	2	markets
high	6	3	markets
high	6	3	markets
high	21	6	markets
high	21	7	markets
high	6	1	markets
high	8	1	markets
high	8	1	markets
high	28	3	markets
high	6	4	markets
high	28	4	markets
high	21	9	markets
high	21	10	markets
low	200	2	markets
low	300	4	markets
low	130	3	markets
low	300	11	markets
low	150	2	markets
low	250	5	markets
low	300	6	markets
low	300	4	markets
low	200	2	markets

very high	11	1	markets
very high	13	1	markets
very high	10	1	markets
very high	10	1	markets
very high	12	1	markets
very high	12	1	markets
very high	10	1	markets
very high	11	1	markets
very high	12	1	markets
medium	16	0	fairs
medium	16	0	fairs
medium	16	0	fairs
medium	16	0	fairs
medium	16	1	fairs
medium	16	1	fairs
medium	16	1	fairs
medium	16	2	multiple locations
medium	16	0	fairs
medium	16	1	fairs
medium	16	1	fairs
medium	16	1	fairs
medium	16	2	fairs
medium	16	2	fairs
medium	16	2	fairs
medium	16	4	fairs
medium	16	0	fairs
medium	16	0	fairs
medium	16	0	fairs
medium	16	0	fairs
medium	16	0	fairs
medium	16	1	fairs
medium	16	2	fairs
medium	16	2	fairs
medium	16	1	fairs
medium	16	1	fairs
medium	16	2	fairs
medium	16	2	fairs
medium	16	2	fairs
medium	16	2	fairs
medium	16	3	fairs
medium	16	4	fairs
medium	16	0	fairs
medium	16	0	fairs
medium	16	0	fairs
medium	16	0	fairs
medium	16	0	fairs
medium	16	0	fairs
medium	16	1	fairs
medium	16	1	fairs
medium	16	0	fairs

medium	16	0	fairs
medium	16	0	fairs
medium	16	0	fairs
medium	16	0	fairs
medium	16	0	fairs
medium	16	0	fairs
medium	16	1	fairs
medium	16	0	fairs
medium	16	0	fairs
medium	16	0	fairs
medium	16	0	fairs
medium	16	0	fairs
medium	16	0	fairs
medium	16	0	fairs
medium	16	1	fairs
very high	13	1	markets
very high	17	1	markets
very high	17	3	markets
very high	25	3	markets
very high	28	4	markets
very high	13	1	markets
very high	17	1	markets
very high	17	3	markets
very high	25	5	markets
very high	17	1	markets
very high	17	1	markets
very high	25	2	markets
very high	28	4	markets
very high	13	1	markets
very high	17	3	markets
very high	25	1	markets
very high	13	3	markets
very high	17	4	markets
very high	28	4	markets
very high	25	1	markets
very high	28	1	markets
very high	17	2	markets
very high	25	2	markets
very high	13	1	markets
very high	17	1	markets
very high	13	1	markets
very high	17	1	markets
very high	25	1	markets
very high	28	2	markets
very high	17	1	markets
very high	25	1	markets
very high	28	2	markets
low	23	0	markets
low	66	0	markets
low	96	0	markets

low	62	3	markets
low	100	6	markets
low	100	0	markets
low	23	1	markets
low	62	4	markets
low	96	4	markets
low	66	6	markets
low	96	0	markets
low	23	2	markets
low	66	2	markets
low	62	7	markets
low	100	9	markets
low	62	0	markets
low	96	0	markets
low	23	1	markets
low	66	2	markets
low	100	4	markets
low	23	0	markets
low	66	0	markets
low	96	0	markets
low	62	7	markets
low	100	22	markets
low	23	0	markets
low	100	0	markets
low	62	4	markets
low	66	4	markets
low	96	16	markets
low	23	0	markets
low	62	0	markets
low	66	0	markets
low	100	3	markets
low	96	4	markets
low	23	0	markets
low	62	0	markets
low	66	0	markets
low	96	3	markets
low	100	4	markets
medium	25	0	markets
medium	27	0	markets
medium	20	1	markets
medium	28	2	markets
medium	37	2	markets
medium	25	0	markets
medium	20	2	markets
medium	27	2	markets
medium	28	4	markets
medium	37	4	markets
medium	25	0	markets
medium	20	2	markets
medium	27	2	markets

medium	28	2	markets
medium	37	2	markets
medium	20	2	markets
medium	25	2	markets
medium	27	2	markets
medium	28	6	markets
medium	37	6	markets
very high	17	1	multiple locations
very high	20	1	multiple locations
very high	35	1	multiple locations
very high	5	1	multiple locations
very high	18	1	multiple locations
very high	16	1	multiple locations
very high	17	1	multiple locations
very high	26	1	multiple locations
very high	35	1	multiple locations
very high	20	2	multiple locations
very high	16	1	multiple locations
very high	20	1	multiple locations
very high	26	1	multiple locations
very high	10	2	multiple locations
very high	17	2	multiple locations
very high	35	3	multiple locations
very high	10	1	multiple locations
very high	16	1	multiple locations
very high	20	1	multiple locations
very high	20	1	multiple locations
very high	17	1	multiple locations
very high	20	1	multiple locations
very high	26	1	multiple locations
very high	35	1	multiple locations
very high	16	1	multiple locations
very high	26	1	multiple locations
very high	35	2	multiple locations
very high	470	9	markets
very high	470	20	markets
very high	470	13	markets
very high	470	22	markets
very high	470	27	markets
very high	470	11	markets
very high	470	24	markets
very high	470	11	markets
very high	470	15	markets
very high	470	5	markets
very high	470	2	markets
high	60	1	markets
high	60	1	markets
high	60	1	markets
high	60	1	markets
high	60	1	markets

high	60	1	markets
high	60	3	markets
high	60	1	markets
high	60	1	markets
high	60	3	markets
high	60	25	markets
high	60	1	markets
high	60	21	markets
high	60	37	markets
high	60	1	markets
high	60	1	markets
high	60	1	markets
high	60	3	markets
high	60	2	markets
high	60	1	markets
high	60	1	markets
high	60	1	markets
high	60	2	markets
very high	9	7	vegetable gardens
very high	6	2	vegetable gardens
very high	4	3	vegetable gardens
very high	9	5	vegetable gardens
very high	6	2	vegetable gardens
very high	4	3	vegetable gardens
low	59	29	markets
low	131	51	markets
low	59	6	markets
low	131	11	markets
low	131	4	markets
low	59	6	markets
low	59	7	markets
low	131	9	markets
low	131	17	markets
low	59	25	markets
low	59	0	markets
low	131	17	markets
low	59	12	markets
low	131	13	markets
medium	36	6	vegetable gardens
medium	36	3	vegetable gardens
medium	36	20	vegetable gardens
medium	36	4	vegetable gardens
high	12	0	vegetable gardens
high	12	1	vegetable gardens
high	15	3	vegetable gardens
high	12	0	vegetable gardens
high	12	1	vegetable gardens
high	15	4	vegetable gardens
high	12	0	vegetable gardens
high	12	0	vegetable gardens

high	15	1	vegetable gardens
high	12	0	vegetable gardens
high	12	0	vegetable gardens
high	15	2	vegetable gardens
high	12	0	vegetable gardens
high	15	0	vegetable gardens
high	12	1	vegetable gardens
medium	92	0	multiple locations
medium	121	0	multiple locations
medium	122	0	multiple locations
medium	129	0	multiple locations
medium	112	2	multiple locations
medium	165	2	multiple locations
medium	121	3	multiple locations
medium	151	3	multiple locations
high	80	32	markets
high	200	54	markets
high	80	6	markets
high	200	13	markets
low	20	1	markets
low	20	2	markets
low	20	2	markets
low	20	2	markets
low	20	3	markets
low	20	3	markets
low	20	3	markets
low	20	4	markets
low	20	6	markets
low	20	7	markets
low	20	1	markets
low	20	1	markets
low	20	1	markets
low	20	2	markets
low	20	2	markets
low	20	2	markets
low	20	2	markets
low	20	2	markets
low	20	2	markets
low	20	5	markets
low	20	1	markets
low	20	1	markets
low	20	1	markets
low	20	1	markets
low	20	2	markets
low	20	2	markets
low	20	2	markets
low	20	2	markets
low	20	3	markets
low	20	4	markets
low	20	1	markets

low	20	1	markets
low	20	1	markets
low	20	1	markets
low	20	1	markets
low	20	2	markets
low	20	2	markets
low	20	2	markets
low	20	3	markets
low	20	4	markets
low	199	12	markets
low	199	1	markets
low	199	23	markets
low	199	22	markets
low	199	58	markets
low	199	4	markets
medium	6	0	markets
medium	7	0	markets
medium	10	0	markets
medium	10	0	markets
medium	11	0	markets
medium	11	0	markets
medium	15	0	markets
medium	15	0	markets
medium	10	1	markets
medium	17	2	markets
low	360	75	multiple locations
low	360	17	markets
low	360	25	multiple locations
low	360	11	multiple locations
low	360	52	multiple locations
low	360	36	multiple locations
low	360	28	multiple locations
low	360	56	multiple locations
low	360	57	multiple locations
low	45	3	markets
low	45	6	markets
low	45	6	markets
low	45	9	markets
low	45	9	markets
low	45	12	markets
low	45	0	markets
low	45	0	markets
low	45	0	markets
low	45	1	markets
low	45	2	markets
low	45	4	markets
low	45	1	markets
low	45	4	markets
low	45	5	markets
low	45	5	markets
low	45	6	markets
low	45	7	markets
low	45	0	markets
low	45	1	markets
low	45	4	markets
low	45	6	markets

low	45	7	markets
low	45	8	markets
low	45	0	markets
low	45	3	markets
low	45	5	markets
low	45	6	markets
low	45	6	markets
low	45	7	markets
low	45	0	markets
low	45	4	markets
low	45	6	markets
low	45	7	markets
low	45	7	markets
low	45	8	markets
low	45	2	markets
low	45	4	markets
low	45	5	markets
low	45	7	markets
low	45	8	markets
low	45	11	markets
medium	28	1	markets
medium	29	1	markets
medium	29	2	markets
medium	28	1	markets
medium	29	1	markets
medium	29	4	markets
medium	16	1	markets
medium	28	2	markets
medium	30	3	markets
medium	29	4	markets
medium	29	12	markets
very high	35	0	multiple locations
very high	35	0	multiple locations
very high	35	0	multiple locations
very high	35	0	multiple locations
very high	35	0	multiple locations
very high	35	0	multiple locations
very high	35	0	multiple locations
very high	35	0	multiple locations
very high	35	0	multiple locations
very high	35	0	multiple locations
very high	35	0	multiple locations
very high	35	0	multiple locations
very high	35	0	multiple locations
very high	35	0	multiple locations
very high	35	0	multiple locations
very high	35	0	multiple locations
very high	35	0	multiple locations
very high	35	0	multiple locations
very high	35	0	multiple locations
very high	35	0	multiple locations

high	30	1	markets
high	20	3	markets
high	29	3	markets
high	30	2	markets
high	30	4	markets
high	15	6	markets
high	21	7	markets
high	30	7	markets
high	29	8	markets
high	30	9	markets
high	20	1	markets
high	30	1	markets
high	30	1	markets
high	30	1	markets
high	15	2	markets
high	29	2	markets
high	30	2	markets
high	21	4	markets
high	30	6	markets
high	30	9	markets
high	15	1	markets
high	21	1	markets
high	30	1	markets
high	30	2	markets
high	30	2	markets
high	30	1	markets
high	30	1	markets
high	30	2	markets
high	30	3	markets
high	1	0	markets
high	21	0	markets
high	4	1	markets
high	4	1	markets
high	5	1	markets
high	14	1	markets
high	12	2	markets
high	18	2	markets
high	19	2	markets
high	20	3	markets
high	1	0	markets
high	4	0	markets
high	4	0	markets
high	5	0	markets
high	12	0	markets
high	14	0	markets
high	19	0	markets
high	20	0	markets
high	18	1	markets
high	21	1	markets
high	1	0	markets

high	4	0	markets
high	4	0	markets
high	12	0	markets
high	19	0	markets
high	20	0	markets
high	21	0	markets
high	5	2	markets
high	14	2	markets
high	18	3	markets
high	1	0	markets
high	4	0	markets
high	4	0	markets
high	12	0	markets
high	19	0	markets
high	20	0	markets
high	5	1	markets
high	18	2	markets
high	14	4	markets
high	21	4	markets
high	1	0	markets
high	4	0	markets
high	4	0	markets
high	5	0	markets
high	12	0	markets
high	14	0	markets
high	18	0	markets
high	19	0	markets
high	21	0	markets
high	20	1	markets
high	1	0	markets
high	4	0	markets
high	4	0	markets
high	12	0	markets
high	14	0	markets
high	18	0	markets
high	19	0	markets
high	20	0	markets
high	21	0	markets
high	5	1	markets
high	1	0	markets
high	4	0	markets
high	4	0	markets
high	5	0	markets
high	12	0	markets
high	18	0	markets
high	19	0	markets
high	20	0	markets
high	21	0	markets
high	14	1	markets
high	1	0	markets
high	4	0	markets
high	4	0	markets

high	5	0	markets
high	12	0	markets
high	14	0	markets
high	18	0	markets
high	19	0	markets
high	20	0	markets
high	21	1	markets
high	1	0	markets
high	4	0	markets
high	4	0	markets
high	5	0	markets
high	12	0	markets
high	18	0	markets
high	20	0	markets
high	21	0	markets
high	14	1	markets
high	19	1	markets
high	5	0	vegetable gardens
high	6	2	vegetable gardens
high	60	3	multiple locations
high	60	4	multiple locations
high	60	10	multiple locations
high	60	21	multiple locations
high	60	23	multiple locations
high	60	7	markets
high	60	8	markets
high	60	26	markets
high	60	20	multiple locations
high	60	27	multiple locations
high	60	2	multiple locations
high	60	5	multiple locations
high	60	15	multiple locations
high	60	19	multiple locations
high	60	23	multiple locations
high	60	1	multiple locations
high	60	3	multiple locations
high	60	6	multiple locations
high	60	9	multiple locations
high	60	2	multiple locations
high	60	2	multiple locations
high	60	4	multiple locations
high	60	8	multiple locations
high	60	11	multiple locations
high	60	22	multiple locations
high	60	25	multiple locations
high	60	10	multiple locations
high	60	5	multiple locations
high	60	6	multiple locations
high	60	12	multiple locations
high	60	15	multiple locations
high	60	19	multiple locations
high	52	8	markets

high	50	5	markets
high	50	1	markets
high	50	2	markets
high	50	3	markets
high	50	3	markets
high	50	4	markets
high	50	4	markets
high	50	5	markets
high	50	5	markets
high	50	6	markets
high	50	7	markets
high	50	9	markets
very high	544	9	markets
very high	544	10	markets
very high	544	32	markets
high	108	0	markets
high	116	1	markets
high	101	2	markets
high	108	3	markets
high	116	9	markets
high	101	14	markets
high	108	1	markets
high	101	3	markets
high	116	11	markets
high	108	2	markets
high	116	13	markets
high	101	16	markets
high	108	4	markets
high	116	4	markets
high	101	6	markets
high	108	0	markets
high	101	1	markets
high	116	7	markets
high	103	0	markets
high	103	2	markets
high	103	4	markets
high	103	4	markets
high	103	3	markets
high	103	3	markets
high	102	0	markets
high	102	8	markets
high	102	7	markets
high	102	12	markets
high	102	2	markets
high	102	4	markets
very high	10	0	vegetable gardens
very high	10	2	vegetable gardens
very high	20	4	vegetable gardens
very high	15	7	vegetable gardens
very high	50	20	multiple locations
very high	10	0	vegetable gardens
very high	10	0	vegetable gardens
very high	15	8	vegetable gardens

very high	20	9	vegetable gardens
very high	50	12	multiple locations
very high	10	3	vegetable gardens
very high	10	4	vegetable gardens
very high	15	9	vegetable gardens
very high	20	12	vegetable gardens
very high	50	40	multiple locations
very high	10	0	vegetable gardens
very high	10	0	vegetable gardens
very high	20	0	vegetable gardens
very high	15	3	vegetable gardens
very high	50	18	vegetable gardens
high	110	0	markets
high	110	2	markets
high	110	3	markets
high	110	4	markets
high	110	7	markets
high	110	0	markets
high	110	0	markets
high	110	1	markets
high	110	1	markets
high	110	1	markets
high	110	1	markets
high	110	2	markets
high	110	7	markets
high	110	10	markets
high	110	14	markets
high	110	1	markets
high	110	1	markets
high	110	2	markets
high	110	4	markets
high	110	6	markets
high	110	0	markets
high	110	0	markets
high	110	0	markets
high	110	1	markets
high	110	3	markets
high	110	0	markets
high	110	2	markets
high	110	3	markets
high	110	6	markets
high	110	8	markets
high	110	1	markets
high	110	1	markets
high	110	3	markets
high	110	3	markets
high	110	3	markets
high	110	0	markets
high	110	0	markets
high	110	1	markets
high	110	3	markets
high	110	4	markets
low	270	34	markets

low	270	24	markets
low	270	22	markets
low	270	7	markets
low	270	23	markets
low	270	30	markets
low	270	28	markets
high	24	0	markets
high	27	0	markets
high	28	0	markets
high	30	0	markets
high	22	1	markets
high	27	1	markets
high	29	2	markets
high	27	7	markets
high	29	9	markets
high	30	9	markets
high	31	14	markets
high	22	0	markets
high	29	0	markets
high	24	1	markets
high	28	2	markets
high	29	2	markets
high	31	3	markets
high	27	4	markets
high	30	4	markets
high	30	4	markets
high	27	5	markets
high	27	5	markets
high	24	0	markets
high	27	0	markets
high	28	0	markets
high	30	0	markets
high	29	1	markets
high	30	2	markets
high	22	3	markets
high	29	3	markets
high	31	4	markets
high	27	6	markets
high	27	6	markets
high	22	0	markets
high	29	0	markets
high	24	1	markets
high	27	1	markets
high	27	2	markets
high	29	3	markets
high	30	3	markets
high	27	4	markets
high	28	4	markets
high	30	5	markets
high	31	5	markets
high	22	0	markets
high	24	0	markets
high	27	0	markets
high	27	0	markets

high	28	0	markets
high	31	0	markets
high	30	1	markets
high	27	2	markets
high	29	2	markets
high	29	2	markets
high	30	3	markets
high	27	0	markets
high	27	0	markets
high	29	0	markets
high	30	0	markets
high	30	0	markets
high	24	1	markets
high	29	1	markets
high	31	1	markets
high	27	3	markets
high	28	3	markets
high	22	4	markets
high	19	1	vegetable gardens
high	7	1	vegetable gardens
high	1	0	vegetable gardens
high	1	1	vegetable gardens
high	5	1	vegetable gardens
high	7	2	vegetable gardens
high	9	3	vegetable gardens
high	19	4	vegetable gardens
high	7	1	vegetable gardens
high	19	3	vegetable gardens
very high	19	4	markets
very high	38	5	markets
very high	24	6	markets
very high	27	7	markets
very high	29	18	markets
very high	5	2	markets
very high	24	2	markets
very high	38	2	markets
very high	16	3	markets
very high	19	3	markets
very high	27	3	markets
very high	26	4	markets
very high	29	4	markets
very high	27	5	markets
very high	16	6	markets
very high	19	6	markets
very high	26	6	markets
very high	29	6	markets
very high	38	7	markets
very high	26	8	markets
very high	19	4	markets
very high	16	2	markets
very high	19	2	markets
very high	5	3	markets
very high	16	5	markets
very high	38	9	markets
very high	29	1	markets
very high	24	2	markets
very high	26	2	markets
very high	27	3	markets

high	11	0	vegetable gardens
high	11	0	vegetable gardens
high	12	0	vegetable gardens
high	12	0	vegetable gardens
high	12	1	vegetable gardens
high	12	2	vegetable gardens
high	12	2	vegetable gardens
high	14	3	vegetable gardens
high	14	6	vegetable gardens
high	10	0	markets
high	12	0	markets
high	12	0	markets
high	12	0	markets
high	11	1	markets
high	11	1	markets
high	12	1	markets
high	13	2	markets
high	16	2	markets
high	11	0	vegetable gardens
high	12	0	vegetable gardens
high	12	1	vegetable gardens
high	12	1	vegetable gardens
high	12	2	vegetable gardens
high	12	2	vegetable gardens
high	11	3	vegetable gardens
high	14	4	vegetable gardens
high	14	5	vegetable gardens
high	13	0	markets
high	11	1	markets
high	11	1	markets
high	12	1	markets
high	12	2	markets
high	12	2	markets
high	10	3	markets
high	16	3	markets
high	12	4	markets
high	12	0	vegetable gardens
high	12	1	vegetable gardens
high	12	1	vegetable gardens
high	12	1	vegetable gardens
high	12	2	vegetable gardens
high	11	3	vegetable gardens
high	14	3	vegetable gardens
high	14	4	vegetable gardens
high	11	5	vegetable gardens
high	11	0	markets
high	12	1	markets
high	12	1	markets
high	12	2	markets
high	13	2	markets
high	10	3	markets
high	11	3	markets
high	12	4	markets
high	16	4	markets
high	40	4	markets
high	40	11	markets
high	40	26	markets

high	40	11	markets
high	40	25	markets
high	40	3	markets
high	40	2	markets
high	40	1	markets
high	40	6	markets
high	40	1	markets
high	40	2	markets
high	40	8	markets
high	40	1	markets
high	40	16	markets
high	30	0	markets
high	30	3	markets
high	30	6	markets
high	30	3	markets
high	30	15	markets
high	30	18	markets
high	30	4	markets
high	30	21	markets
high	30	23	markets
high	30	3	markets
high	30	10	markets
high	30	15	markets
high	30	0	markets
high	30	0	markets
high	30	5	markets
high	30	0	markets
high	30	0	markets
high	30	3	markets
high	30	0	markets
high	30	0	markets
high	30	3	markets
high	30	1	markets
high	30	18	markets
high	30	21	markets
high	30	0	markets
high	30	2	markets
high	30	3	markets
high	30	0	markets
high	30	0	markets
high	30	3	markets
high	40	2	fairs
high	40	1	markets
high	40	3	markets
high	40	3	markets
high	40	20	fairs
high	40	14	fairs
high	40	17	markets
high	40	20	markets
high	40	20	markets
high	40	9	markets
high	40	9	markets
high	40	14	markets
high	40	7	markets
high	40	7	markets
high	40	9	markets
high	40	15	fairs
high	40	3	markets

high	40	8	markets
high	40	8	markets
high	40	1	markets
high	40	1	markets
high	40	1	markets
high	40	12	fairs
medium	128	13	markets
medium	128	20	markets
medium	128	29	markets
medium	128	5	markets
medium	128	2	markets
medium	128	1	markets
medium	128	6	markets
medium	128	26	markets
medium	128	13	markets
medium	128	1	markets
medium	128	91	markets
medium	128	1	markets
medium	128	1	markets
medium	128	3	markets
medium	128	2	markets
medium	128	1	markets
medium	128	1	markets
medium	128	12	markets
medium	128	1	markets
medium	128	50	markets
medium	128	5	markets
medium	40	1	markets
medium	40	2	markets
medium	70	3	markets
medium	70	5	markets
medium	70	6	markets
medium	70	6	markets
medium	40	0	markets
medium	40	1	markets
medium	70	1	markets
medium	70	2	markets
medium	70	3	markets
medium	70	3	markets
medium	40	1	markets
medium	40	3	markets
medium	70	4	markets
medium	70	4	markets
medium	70	6	markets
medium	70	7	markets
medium	40	0	markets
medium	40	0	markets
medium	70	1	markets
medium	70	1	markets
medium	70	2	markets
medium	70	3	markets
medium	40	1	markets
medium	40	1	markets
medium	70	3	markets
medium	70	4	markets
medium	70	5	markets
medium	70	5	markets
medium	40	0	markets

medium	40	0	markets
medium	70	2	markets
medium	70	2	markets
medium	70	2	markets
medium	70	1	markets
medium	40	0	markets
medium	40	0	markets
medium	70	0	markets
medium	70	1	markets
medium	70	1	markets
medium	70	1	markets
medium	40	0	markets
medium	40	0	markets
medium	70	1	markets
medium	70	1	markets
medium	70	1	markets
medium	70	2	markets
medium	40	0	markets
medium	40	0	markets
medium	70	1	markets
medium	70	2	markets
medium	70	2	markets
medium	70	3	markets
medium	40	0	markets
medium	40	0	markets
medium	70	0	markets
medium	70	1	markets
medium	70	2	markets
medium	70	2	markets
very high	2	0	not indicated
very high	2	0	not indicated
very high	2	0	not indicated
very high	2	0	not indicated
very high	2	0	not indicated
very high	2	0	not indicated
very high	2	0	not indicated
very high	2	0	not indicated
very high	2	0	not indicated
very high	3	0	not indicated
very high	3	0	not indicated
very high	3	0	not indicated
very high	3	0	not indicated
very high	3	0	not indicated
very high	3	0	not indicated
very high	3	0	not indicated
very high	3	0	not indicated
very high	3	0	not indicated
very high	3	0	not indicated
very high	3	0	not indicated
very high	3	0	not indicated
very high	3	0	not indicated
very high	3	0	not indicated
very high	3	0	not indicated
very high	3	0	not indicated
very high	3	1	not indicated
very high	3	1	not indicated
very high	2	0	not indicated
very high	2	0	not indicated

very high	2	0	not indicated
very high	2	0	not indicated
very high	2	0	not indicated
very high	2	0	not indicated
very high	2	0	not indicated
very high	2	0	not indicated
very high	2	0	not indicated
very high	3	0	not indicated
very high	3	0	not indicated
very high	3	0	not indicated
very high	3	0	not indicated
very high	3	0	not indicated
very high	3	0	not indicated
very high	3	0	not indicated
very high	3	0	not indicated
very high	3	0	not indicated
very high	3	0	not indicated
very high	3	0	not indicated
very high	3	0	not indicated
very high	3	0	not indicated
very high	3	0	not indicated
very high	3	0	not indicated
very high	3	0	not indicated
very high	3	0	not indicated
very high	3	0	not indicated
high	83	0	vegetable gardens
high	93	0	vegetable gardens
high	93	0	vegetable gardens
high	95	0	vegetable gardens
high	97	0	vegetable gardens
high	99	0	vegetable gardens
high	100	0	vegetable gardens
high	105	0	vegetable gardens
high	105	0	vegetable gardens
high	110	0	vegetable gardens
high	106	2	vegetable gardens
high	90	5	vegetable gardens
high	110	14	vegetable gardens
high	100	23	vegetable gardens
high	112	25	vegetable gardens
high	83	0	vegetable gardens
high	93	0	vegetable gardens
high	93	0	vegetable gardens
high	97	0	vegetable gardens
high	99	0	vegetable gardens
high	100	0	vegetable gardens
high	106	0	vegetable gardens
high	110	0	vegetable gardens
high	110	0	vegetable gardens
high	105	3	vegetable gardens
high	105	8	vegetable gardens
high	95	20	vegetable gardens
high	100	25	vegetable gardens
high	90	35	vegetable gardens
high	112	48	vegetable gardens
high	80	0	markets
high	110	0	markets
high	120	0	markets

high	74	2	markets
high	103	2	markets
high	80	3	markets
high	98	3	markets
high	100	4	markets
high	88	5	markets
high	100	6	markets
high	103	8	markets
high	101	12	markets
high	83	0	vegetable gardens
high	90	0	vegetable gardens
high	93	0	vegetable gardens
high	93	0	vegetable gardens
high	97	0	vegetable gardens
high	99	0	vegetable gardens
high	100	0	vegetable gardens
high	105	0	vegetable gardens
high	106	0	vegetable gardens
high	110	0	vegetable gardens
high	105	3	vegetable gardens
high	110	10	vegetable gardens
high	95	24	vegetable gardens
high	100	24	vegetable gardens
high	112	51	vegetable gardens
high	74	0	markets
high	80	0	markets
high	100	0	markets
high	103	0	markets
high	120	0	markets
high	80	1	markets
high	103	1	markets
high	101	3	markets
high	110	3	markets
high	98	12	markets
high	88	14	markets
high	100	14	markets
high	83	0	vegetable gardens
high	90	0	vegetable gardens
high	95	0	vegetable gardens
high	110	0	vegetable gardens
high	93	10	vegetable gardens
high	97	13	vegetable gardens
high	106	15	vegetable gardens
high	100	17	vegetable gardens
high	93	18	vegetable gardens
high	105	22	vegetable gardens
high	99	23	vegetable gardens
high	105	27	vegetable gardens
high	110	29	vegetable gardens
high	112	44	vegetable gardens
high	100	51	vegetable gardens
high	93	0	vegetable gardens
high	99	0	vegetable gardens
high	110	6	vegetable gardens
high	112	6	vegetable gardens
high	83	9	vegetable gardens
high	93	9	vegetable gardens
high	105	10	vegetable gardens

high	105	10	vegetable gardens
high	90	13	vegetable gardens
high	100	13	vegetable gardens
high	106	13	vegetable gardens
high	100	14	vegetable gardens
high	110	14	vegetable gardens
high	97	18	vegetable gardens
high	95	23	vegetable gardens
high	74	0	markets
high	80	0	markets
high	80	0	markets
high	88	0	markets
high	100	0	markets
high	100	0	markets
high	103	0	markets
high	103	0	markets
high	110	0	markets
high	120	0	markets
high	98	6	markets
high	101	8	markets
high	97	0	vegetable gardens
high	83	2	vegetable gardens
high	110	3	vegetable gardens
high	99	7	vegetable gardens
high	95	8	vegetable gardens
high	106	10	vegetable gardens
high	90	13	vegetable gardens
high	110	21	vegetable gardens
high	93	22	vegetable gardens
high	100	23	vegetable gardens
high	93	24	vegetable gardens
high	105	29	vegetable gardens
high	112	30	vegetable gardens
high	105	34	vegetable gardens
high	100	67	vegetable gardens
high	74	0	markets
high	80	0	markets
high	88	0	markets
high	100	0	markets
high	101	0	markets
high	103	0	markets
high	110	0	markets
high	100	3	markets
high	120	3	markets
high	80	4	markets
high	103	20	markets
high	98	42	markets
high	83	0	vegetable gardens
high	97	0	vegetable gardens
high	100	0	vegetable gardens
high	110	3	vegetable gardens
high	93	5	vegetable gardens
high	106	5	vegetable gardens

high	93	6	vegetable gardens
high	105	6	vegetable gardens
high	105	8	vegetable gardens
high	99	10	vegetable gardens
high	90	12	vegetable gardens
high	110	13	vegetable gardens
high	95	16	vegetable gardens
high	100	23	vegetable gardens
high	112	39	vegetable gardens
high	74	0	markets
high	80	0	markets
high	98	0	markets
high	100	0	markets
high	100	0	markets
high	103	0	markets
high	110	0	markets
high	120	0	markets
high	88	3	markets
high	101	3	markets
high	103	6	markets
high	80	15	markets
high	93	0	vegetable gardens
high	93	0	vegetable gardens
high	97	0	vegetable gardens
high	99	0	vegetable gardens
high	100	0	vegetable gardens
high	105	0	vegetable gardens
high	83	1	vegetable gardens
high	110	1	vegetable gardens
high	90	2	vegetable gardens
high	95	8	vegetable gardens
high	105	9	vegetable gardens
high	106	11	vegetable gardens
high	110	12	vegetable gardens
high	100	15	vegetable gardens
high	112	19	vegetable gardens
high	74	0	markets
high	80	0	markets
high	80	0	markets
high	100	0	markets
high	100	0	markets
high	103	0	markets
high	103	0	markets
high	120	0	markets
high	88	1	markets
high	110	1	markets
high	98	3	markets
high	101	6	markets
high	83	0	vegetable gardens
high	90	0	vegetable gardens
high	93	0	vegetable gardens
high	93	0	vegetable gardens
high	95	0	vegetable gardens

high	97	0	vegetable gardens
high	99	0	vegetable gardens
high	100	0	vegetable gardens
high	106	0	vegetable gardens
high	112	2	vegetable gardens
high	110	10	vegetable gardens
high	100	14	vegetable gardens
high	105	17	vegetable gardens
high	105	18	vegetable gardens
high	110	31	vegetable gardens
high	80	0	markets
high	100	0	markets
high	103	0	markets
high	80	1	markets
high	100	1	markets
high	88	2	markets
high	98	2	markets
high	110	2	markets
high	74	3	markets
high	101	3	markets
high	120	4	markets
high	103	5	markets
high	105	0	vegetable gardens
high	110	0	vegetable gardens
high	93	1	vegetable gardens
high	97	2	vegetable gardens
high	93	4	vegetable gardens
high	106	5	vegetable gardens
high	105	6	vegetable gardens
high	83	9	vegetable gardens
high	100	11	vegetable gardens
high	99	12	vegetable gardens
high	95	14	vegetable gardens
high	110	15	vegetable gardens
high	90	17	vegetable gardens
high	100	20	vegetable gardens
high	112	31	vegetable gardens
high	80	0	markets
high	110	0	markets
high	74	1	markets
high	100	1	markets
high	80	2	markets
high	103	2	markets
high	100	3	markets
high	103	3	markets
high	120	5	markets
high	88	6	markets
high	101	19	markets
high	98	25	markets
high	83	0	vegetable gardens
high	99	0	vegetable gardens

high	110	2	vegetable gardens
high	97	5	vegetable gardens
high	110	7	vegetable gardens
high	100	9	vegetable gardens
high	106	9	vegetable gardens
high	90	10	vegetable gardens
high	93	10	vegetable gardens
high	93	16	vegetable gardens
high	95	19	vegetable gardens
high	105	20	vegetable gardens
high	105	26	vegetable gardens
high	112	37	vegetable gardens
high	100	39	vegetable gardens
high	74	0	markets
high	100	0	markets
high	103	0	markets
high	103	0	markets
high	110	0	markets
high	100	1	markets
high	120	1	markets
high	80	2	markets
high	80	3	markets
high	88	3	markets
high	98	4	markets
high	101	9	markets
high	97	1	vegetable gardens
high	83	4	vegetable gardens
high	90	5	vegetable gardens
high	93	5	vegetable gardens
high	99	6	vegetable gardens
high	110	9	vegetable gardens
high	110	11	vegetable gardens
high	100	16	vegetable gardens
high	106	17	vegetable gardens
high	95	19	vegetable gardens
high	93	23	vegetable gardens
high	105	23	vegetable gardens
high	105	25	vegetable gardens
high	112	45	vegetable gardens
high	100	47	vegetable gardens
high	74	0	markets
high	80	0	markets
high	88	0	markets
high	100	0	markets
high	100	0	markets
high	103	0	markets
high	103	0	markets
high	110	1	markets
high	80	5	markets
high	120	10	markets
high	101	15	markets

high	98	21	markets
high	5	0	markets
high	11	0	markets
high	13	0	markets
high	1	1	markets
high	4	1	markets
high	9	1	markets
high	10	1	markets
high	12	1	markets
high	10	2	markets
high	13	4	markets
high	1	0	markets
high	5	0	markets
high	9	0	markets
high	10	0	markets
high	10	0	markets
high	11	0	markets
high	4	1	markets
high	12	1	markets
high	13	2	markets
high	13	3	markets
high	1	0	markets
high	5	0	markets
high	11	0	markets
high	4	1	markets
high	9	1	markets
high	10	1	markets
high	12	1	markets
high	13	1	markets
high	13	1	markets
high	10	2	markets
very high	6	2	vegetable gardens
very high	4	3	vegetable gardens
very high	9	7	vegetable gardens
very high	6	2	vegetable gardens
very high	4	3	vegetable gardens
very high	9	5	vegetable gardens
low	450	77	markets
low	450	63	markets
low	450	89	markets
low	450	11	markets
low	450	75	markets
low	450	112	markets
low	450	23	markets
very high	20	1	multiple locations
very high	35	2	multiple locations
very high	25	6	multiple locations
high	141	2	markets
very high	141	5	vegetable gardens
high	141	5	markets

high	141	1	markets
very high	141	4	vegetable gardens
high	141	2	markets
very high	141	2	vegetable gardens
very high	141	1	markets
high	141	2	markets
very high	141	3	vegetable gardens
high	141	2	markets
very high	141	3	vegetable gardens
medium	902	81	markets
medium	457	111	markets
medium	583	124	markets
medium	902	47	markets
medium	457	72	markets
medium	583	80	markets
medium	902	19	markets
medium	583	21	markets
medium	457	30	markets
very high	3	0	markets
very high	3	0	markets
very high	3	0	markets
very high	3	0	markets
very high	3	0	markets
very high	3	1	markets
very high	3	1	markets
very high	3	1	markets
very high	0	0	vegetable gardens
very high	0	0	vegetable gardens
very high	0	0	vegetable gardens
very high	0	0	vegetable gardens
very high	0	0	vegetable gardens
very high	0	0	vegetable gardens
very high	0	0	vegetable gardens
very high	1	0	vegetable gardens
very high	1	0	vegetable gardens
very high	1	0	vegetable gardens
very high	1	0	vegetable gardens
very high	1	0	vegetable gardens
very high	1	0	vegetable gardens
very high	1	0	vegetable gardens
very high	1	0	vegetable gardens
very high	1	0	vegetable gardens
very high	1	0	vegetable gardens
very high	1	0	vegetable gardens
very high	1	0	vegetable gardens
very high	1	0	vegetable gardens
very high	2	0	vegetable gardens
very high	2	0	vegetable gardens
very high	2	0	vegetable gardens
very high	3	0	vegetable gardens
very high	3	0	vegetable gardens

very high	3	0	vegetable gardens
very high	3	0	vegetable gardens
very high	3	0	vegetable gardens
very high	4	0	vegetable gardens
very high	4	0	vegetable gardens
very high	5	0	vegetable gardens
very high	7	0	vegetable gardens
very high	7	0	vegetable gardens
very high	8	0	vegetable gardens
very high	3	1	vegetable gardens
very high	4	1	vegetable gardens
very high	4	1	vegetable gardens
very high	6	1	vegetable gardens
very high	7	1	vegetable gardens
very high	12	1	vegetable gardens
very high	0	0	vegetable gardens
very high	0	0	vegetable gardens
very high	0	0	vegetable gardens
very high	0	0	vegetable gardens
very high	0	0	vegetable gardens
very high	0	0	vegetable gardens
very high	0	0	vegetable gardens
very high	1	0	vegetable gardens
very high	1	0	vegetable gardens
very high	1	0	vegetable gardens
very high	1	0	vegetable gardens
very high	1	0	vegetable gardens
very high	1	0	vegetable gardens
very high	1	0	vegetable gardens
very high	1	0	vegetable gardens
very high	1	0	vegetable gardens
very high	1	0	vegetable gardens
very high	1	0	vegetable gardens
very high	1	0	vegetable gardens
very high	2	0	vegetable gardens
very high	2	0	vegetable gardens
very high	2	0	vegetable gardens
very high	3	0	vegetable gardens
very high	3	0	vegetable gardens
very high	3	0	vegetable gardens
very high	3	0	vegetable gardens
very high	3	0	vegetable gardens
very high	5	0	vegetable gardens
very high	6	0	vegetable gardens
very high	8	0	vegetable gardens
very high	1	1	vegetable gardens
very high	3	1	vegetable gardens
very high	4	1	vegetable gardens
very high	4	1	vegetable gardens
very high	4	1	vegetable gardens
very high	7	1	vegetable gardens

very high	7	1	vegetable gardens
very high	12	1	vegetable gardens
very high	7	2	vegetable gardens
very high	4	3	vegetable gardens
very high	0	0	vegetable gardens
very high	0	0	vegetable gardens
very high	0	0	vegetable gardens
very high	0	0	vegetable gardens
very high	0	0	vegetable gardens
very high	0	0	vegetable gardens
very high	0	0	vegetable gardens
very high	1	0	vegetable gardens
very high	1	0	vegetable gardens
very high	1	0	vegetable gardens
very high	1	0	vegetable gardens
very high	1	0	vegetable gardens
very high	1	0	vegetable gardens
very high	1	0	vegetable gardens
very high	1	0	vegetable gardens
very high	1	0	vegetable gardens
very high	1	0	vegetable gardens
very high	1	0	vegetable gardens
very high	1	0	vegetable gardens
very high	1	0	vegetable gardens
very high	2	0	vegetable gardens
very high	2	0	vegetable gardens
very high	2	0	vegetable gardens
very high	3	0	vegetable gardens
very high	3	0	vegetable gardens
very high	3	0	vegetable gardens
very high	3	0	vegetable gardens
very high	3	0	vegetable gardens
very high	4	0	vegetable gardens
very high	4	0	vegetable gardens
very high	5	0	vegetable gardens
very high	7	0	vegetable gardens
very high	7	0	vegetable gardens
very high	7	0	vegetable gardens
very high	8	0	vegetable gardens
very high	12	0	vegetable gardens
very high	3	1	vegetable gardens
very high	4	1	vegetable gardens
very high	4	1	vegetable gardens
very high	6	1	vegetable gardens
very high	8	0	markets
very high	14	0	markets
very high	15	0	markets
very high	15	0	markets
very high	16	0	markets
very high	16	0	markets

very high	40	0	markets
very high	44	0	markets
very high	35	2	markets
very high	8	0	markets
very high	14	0	markets
very high	15	0	markets
very high	16	0	markets
very high	35	0	markets
very high	15	1	markets
very high	16	1	markets
very high	40	1	markets
very high	44	4	markets
very high	8	0	markets
very high	14	0	markets
very high	15	0	markets
very high	15	0	markets
very high	16	0	markets
very high	16	0	markets
very high	40	0	markets
very high	44	1	markets
very high	35	2	markets
medium	72	1	markets
medium	72	2	markets
medium	72	3	markets
medium	72	21	markets
medium	72	22	markets
medium	72	1	markets
medium	72	4	markets
medium	72	8	markets
medium	72	12	markets
medium	72	15	markets
medium	72	0	markets
medium	72	0	markets
medium	72	0	markets
medium	72	0	markets
medium	72	26	markets
medium	72	0	markets
medium	72	8	markets
medium	72	14	markets
medium	72	39	markets
medium	72	70	markets
very high	39	0	markets
very high	40	0	markets
very high	44	0	markets
very high	91	0	markets
very high	113	0	markets
very high	124	0	markets
very high	226	0	markets
very high	107	1	markets
very high	387	1	markets

very high	39	0	markets
very high	40	0	markets
very high	44	0	markets
very high	91	0	markets
very high	113	0	markets
very high	226	0	markets
very high	124	1	markets
very high	387	1	markets
very high	107	4	markets
very high	39	0	markets
very high	40	0	markets
very high	44	0	markets
very high	91	0	markets
very high	107	0	markets
very high	113	0	markets
very high	124	0	markets
very high	226	0	markets
very high	387	3	markets
very high	1	0	vegetable gardens
very high	6	0	vegetable gardens
very high	15	2	vegetable gardens
very high	19	5	vegetable gardens
very high	59	0	markets
very high	54	3	markets
very high	27	4	markets
very high	35	7	markets
very high	32	3	forests
very high	20	4	vegetable gardens
very high	26	8	vegetable gardens
high	3	0	multiple locations
high	5	0	multiple locations
high	11	0	multiple locations
high	15	0	multiple locations
high	18	0	multiple locations
high	20	0	multiple locations
high	20	0	multiple locations
high	25	0	multiple locations
high	26	0	multiple locations
high	27	0	multiple locations
high	28	0	multiple locations
high	28	0	multiple locations
high	41	0	multiple locations
high	44	0	multiple locations
high	47	0	multiple locations
high	59	0	multiple locations
high	70	0	multiple locations
high	128	0	multiple locations
high	152	0	multiple locations
high	200	0	multiple locations
high	132	1	multiple locations

high	3	0	multiple locations
high	5	0	multiple locations
high	11	0	multiple locations
high	15	0	multiple locations
high	18	0	multiple locations
high	20	0	multiple locations
high	20	0	multiple locations
high	25	0	multiple locations
high	26	0	multiple locations
high	27	0	multiple locations
high	28	0	multiple locations
high	28	0	multiple locations
high	41	0	multiple locations
high	47	0	multiple locations
high	59	0	multiple locations
high	70	0	multiple locations
high	128	0	multiple locations
high	132	0	multiple locations
high	152	0	multiple locations
high	44	1	multiple locations
high	200	1	multiple locations
high	5	0	multiple locations
high	11	0	multiple locations
high	18	0	multiple locations
high	20	0	multiple locations
high	20	0	multiple locations
high	27	0	multiple locations
high	47	0	multiple locations
high	3	1	multiple locations
high	15	1	multiple locations
high	25	1	multiple locations
high	26	1	multiple locations
high	41	1	multiple locations
high	44	1	multiple locations
high	59	1	multiple locations
high	70	1	multiple locations
high	152	1	multiple locations
high	28	3	multiple locations
high	28	4	multiple locations
high	128	4	multiple locations
high	132	5	multiple locations
high	200	13	multiple locations
high	12	0	fairs
high	12	1	fairs
high	12	2	fairs
high	12	0	markets
high	12	0	markets
high	12	0	markets
high	12	0	markets
high	12	1	markets
high	12	2	markets

high	12	0	fairs
high	12	0	fairs
high	12	1	fairs
high	12	0	markets
high	12	0	markets
high	12	0	markets
high	12	0	markets
high	12	0	markets
high	12	0	markets
high	12	3	fairs
high	12	4	fairs
high	12	4	fairs
high	12	0	markets
high	12	1	markets
high	12	1	markets
high	12	2	markets
high	12	2	markets
high	12	5	markets
high	12	0	fairs
high	12	0	fairs
high	12	0	fairs
high	12	0	markets
high	12	0	markets
high	12	0	markets
high	12	0	markets
high	12	0	markets
high	12	3	markets
high	12	0	fairs
high	12	0	fairs
high	12	0	fairs
high	12	0	markets
high	12	0	markets
high	12	0	markets
high	12	0	markets
high	12	1	markets
high	12	2	markets
high	12	0	fairs
high	12	0	fairs
high	12	1	fairs
high	12	0	markets
high	12	0	markets
high	12	0	markets
high	12	0	markets
high	12	0	markets
high	12	0	markets
high	12	1	fairs
high	12	3	fairs
high	12	4	fairs
high	12	0	markets
high	12	0	markets
high	12	0	markets

high	12	1	markets
high	12	2	markets
high	12	4	markets
high	12	1	fairs
high	12	2	fairs
high	12	3	fairs
high	12	0	markets
high	12	0	markets
high	12	0	markets
high	12	0	markets
high	12	0	markets
high	12	1	markets
high	12	3	fairs
high	12	6	fairs
high	12	8	fairs
high	12	0	markets
high	12	0	markets
high	12	1	markets
high	12	4	markets
high	12	4	markets
high	12	5	markets
high	12	0	fairs
high	12	0	fairs
high	12	0	fairs
high	12	0	markets
high	12	0	markets
high	12	0	markets
high	12	0	markets
high	12	1	markets
high	12	2	markets
very high	50	0	markets
very high	50	2	markets
very high	50	2	markets
very high	50	6	markets
very high	50	14	markets
very high	50	0	markets
very high	50	0	markets
very high	50	0	markets
very high	50	0	markets
very high	50	4	markets
high	240	1	fairs
high	240	1	fairs
high	240	1	fairs
high	240	1	fairs
high	240	1	markets
high	240	1	markets
high	240	1	markets
high	240	1	markets
high	240	1	markets
high	240	1	markets

high	240	1	markets
high	240	2	markets
high	240	1	fairs
high	240	2	fairs
high	240	1	markets
high	240	1	markets
high	240	2	markets
high	240	2	markets
high	240	2	markets
high	240	1	fairs
high	240	1	markets
high	240	1	fairs
high	240	1	markets
high	240	1	markets
high	240	1	markets
high	240	1	markets
high	240	2	markets
high	240	2	markets
high	240	2	markets
high	240	1	fairs
high	240	1	fairs
high	240	1	fairs
high	240	2	fairs
high	240	1	markets
high	240	1	markets
high	240	1	markets
high	240	1	markets
high	240	1	markets
high	240	1	markets
high	240	1	markets
high	240	2	markets
high	240	1	fairs
high	240	1	fairs
high	240	1	markets
high	240	1	markets
high	240	1	markets
high	240	1	markets
high	240	1	markets
high	240	2	markets
high	240	1	fairs
high	240	1	fairs
high	240	1	fairs
high	240	1	fairs
high	240	1	fairs
high	240	1	fairs
high	240	1	markets
high	240	1	markets
high	240	1	markets
high	240	1	markets
high	240	1	markets

high	240	1	markets
high	240	1	markets
high	240	1	markets
high	240	1	markets
high	240	1	markets
high	240	1	markets
high	240	2	markets
high	240	2	markets
high	240	2	markets
high	240	2	markets
high	240	1	markets
high	240	1	markets
high	119	17	markets
high	119	87	markets
high	119	10	markets
high	119	33	markets
high	119	5	markets
high	119	8	markets
very high	not indicated	0	vegetable gardens
very high	not indicated	0	vegetable gardens
very high	not indicated	0	vegetable gardens
very high	not indicated	0	vegetable gardens
low	25	4	vegetable gardens
low	25	6	vegetable gardens
low	25	8	vegetable gardens
low	25	9	vegetable gardens
low	25	10	vegetable gardens
low	25	10	vegetable gardens
low	25	11	vegetable gardens
low	25	12	vegetable gardens
high	7	1	fairs
high	40	2	fairs
high	5	1	vegetable gardens
high	168	5	vegetable gardens
low	7	0	fairs
low	11	0	fairs
low	12	0	fairs
low	16	0	fairs
low	19	0	fairs
low	23	0	fairs
low	24	0	fairs
low	25	0	fairs
low	36	0	fairs
low	50	0	fairs
low	36	1	fairs
low	7	0	fairs
low	12	0	fairs
low	24	0	fairs
low	19	1	fairs
low	23	1	fairs

low	25	1	fairs
low	36	1	fairs
low	11	2	fairs
low	16	2	fairs
low	50	3	fairs
low	36	4	fairs
low	7	0	fairs
low	12	0	fairs
low	16	0	fairs
low	19	0	fairs
low	11	1	fairs
low	24	1	fairs
low	25	1	fairs
low	36	1	fairs
low	36	1	fairs
low	50	1	fairs
low	23	2	fairs
low	7	0	fairs
low	11	0	fairs
low	12	0	fairs
low	16	0	fairs
low	24	0	fairs
low	25	0	fairs
low	36	0	fairs
low	36	0	fairs
low	50	0	fairs
low	19	1	fairs
low	23	1	fairs
low	7	0	fairs
low	11	0	fairs
low	12	0	fairs
low	16	0	fairs
low	19	0	fairs
low	24	0	fairs
low	25	0	fairs
low	36	0	fairs
low	36	0	fairs
low	50	1	fairs
low	23	2	fairs
low	7	0	fairs
low	11	0	fairs
low	12	0	fairs
low	19	0	fairs
low	23	0	fairs
low	24	0	fairs
low	25	0	fairs
low	36	0	fairs
low	36	0	fairs
low	50	0	fairs
low	16	1	fairs

medium	116	73	markets
medium	20	7	vegetable gardens
medium	28	7	vegetable gardens
medium	30	7	vegetable gardens
medium	30	11	vegetable gardens
medium	32	14	vegetable gardens
medium	32	16	vegetable gardens
medium	20	1	vegetable gardens
medium	28	1	vegetable gardens
medium	30	1	vegetable gardens
medium	30	2	vegetable gardens
medium	32	3	vegetable gardens
medium	32	3	vegetable gardens
medium	28	0	vegetable gardens
medium	30	0	vegetable gardens
medium	32	0	vegetable gardens
medium	20	1	vegetable gardens
medium	30	1	vegetable gardens
medium	32	1	vegetable gardens
high	20	0	fairs
high	20	0	markets
high	22	0	markets
high	17	1	markets
high	20	1	fairs
high	17	0	markets
high	20	0	markets
high	22	0	markets
high	20	2	fairs
high	20	2	markets
high	17	3	markets
high	22	5	markets
high	20	6	fairs
high	17	3	markets
high	20	8	markets
high	22	9	markets
high	20	1	fairs
high	20	1	markets
high	17	2	markets
high	22	2	markets
high	20	1	fairs
high	20	0	markets
high	22	0	markets
high	17	1	markets
high	20	0	fairs
high	20	4	fairs
high	20	1	fairs
high	22	0	markets
high	17	1	markets
high	20	1	markets
high	22	1	markets
high	17	4	markets

high	20	4	markets
high	20	1	markets
high	17	2	markets
high	22	2	markets
high	20	0	fairs
high	20	0	markets
high	22	0	markets
high	17	1	markets
high	20	0	fairs
high	22	0	markets
high	17	1	markets
high	20	1	markets
high	20	0	fairs
high	20	3	fairs
high	17	0	markets
high	20	0	markets
high	22	0	markets
high	20	1	markets
high	17	2	markets
high	22	2	markets
very high	10	0	vegetable gardens
very high	10	2	vegetable gardens
very high	20	4	vegetable gardens
very high	15	7	vegetable gardens
very high	50	40	vegetable gardens
very high	10	0	vegetable gardens
very high	10	0	vegetable gardens
very high	15	8	vegetable gardens
very high	20	9	vegetable gardens
very high	50	12	vegetable gardens
very high	10	3	vegetable gardens
very high	10	4	vegetable gardens
very high	15	10	vegetable gardens
very high	20	12	vegetable gardens
very high	50	40	vegetable gardens
very high	10	0	vegetable gardens
very high	10	0	vegetable gardens
very high	20	0	vegetable gardens
very high	15	3	vegetable gardens
very high	50	18	vegetable gardens
low	28	2	vegetable gardens
low	28	9	vegetable gardens
low	28	1	vegetable gardens
low	28	2	vegetable gardens
low	28	9	vegetable gardens
low	28	2	vegetable gardens
low	28	9	vegetable gardens
high	270	9	multiple locations
high	270	27	multiple locations
high	270	17	multiple locations

high	270	3	multiple locations
high	270	13	multiple locations
high	270	11	multiple locations
high	270	27	multiple locations
high	270	54	multiple locations
high	270	67	multiple locations
high	270	19	multiple locations
high	270	13	multiple locations
high	270	16	multiple locations
high	270	8	multiple locations
medium	20	1	markets
medium	20	1	markets
medium	20	2	markets
medium	20	3	markets
medium	20	3	markets
medium	20	3	markets
medium	20	4	markets
medium	20	4	markets
medium	20	5	markets
medium	20	5	markets
medium	20	6	markets
medium	20	8	markets
medium	20	1	markets
medium	20	1	markets
medium	20	2	markets
medium	20	2	markets
medium	20	2	markets
medium	20	3	markets
medium	20	3	markets
medium	20	4	markets
medium	20	6	markets
high	30	3	restaurants
high	30	1	restaurants
high	1	0	vegetable gardens
high	1	0	vegetable gardens
high	1	0	vegetable gardens
high	1	0	vegetable gardens
high	1	0	vegetable gardens
high	2	0	vegetable gardens
high	2	0	vegetable gardens
high	2	0	vegetable gardens
high	3	0	vegetable gardens
high	4	0	vegetable gardens
high	4	0	vegetable gardens
high	5	0	vegetable gardens
high	6	0	vegetable gardens
high	6	0	vegetable gardens
high	8	0	vegetable gardens
high	12	0	vegetable gardens
high	14	0	vegetable gardens

high	21	0	vegetable gardens
high	6	1	vegetable gardens
high	1	0	vegetable gardens
high	1	0	vegetable gardens
high	1	0	vegetable gardens
high	1	0	vegetable gardens
high	1	0	vegetable gardens
high	2	0	vegetable gardens
high	2	0	vegetable gardens
high	2	0	vegetable gardens
high	3	0	vegetable gardens
high	4	0	vegetable gardens
high	4	0	vegetable gardens
high	6	0	vegetable gardens
high	6	0	vegetable gardens
high	6	0	vegetable gardens
high	12	0	vegetable gardens
high	14	0	vegetable gardens
high	5	1	vegetable gardens
high	8	1	vegetable gardens
high	21	1	vegetable gardens
high	51	1	restaurants
high	51	2	restaurants
high	51	1	restaurants
high	51	1	restaurants
high	51	9	restaurants
high	19	1	vegetable gardens
high	9	1	vegetable gardens
high	9	3	vegetable gardens
high	19	5	vegetable gardens
high	9	1	vegetable gardens
high	5	1	vegetable gardens
high	9	1	vegetable gardens
high	19	1	vegetable gardens
high	7	1	vegetable gardens
high	19	2	vegetable gardens
high	19	2	vegetable gardens
high	10	0	markets
high	10	0	markets
high	10	1	markets
high	19	1	vegetable gardens
high	15	0	vegetable gardens
high	21	0	vegetable gardens
high	30	0	vegetable gardens
high	30	0	vegetable gardens
high	30	0	vegetable gardens
high	30	0	vegetable gardens
high	30	0	vegetable gardens
high	30	1	vegetable gardens
high	20	3	vegetable gardens

high	29	3	vegetable gardens
high	30	0	vegetable gardens
high	30	0	vegetable gardens
high	20	1	vegetable gardens
high	30	2	vegetable gardens
high	15	6	vegetable gardens
high	30	7	vegetable gardens
high	30	9	vegetable gardens
high	30	4	vegetable gardens
high	21	7	vegetable gardens
high	29	8	vegetable gardens
high	20	0	vegetable gardens
high	30	1	vegetable gardens
high	30	1	vegetable gardens
high	30	1	vegetable gardens
high	15	2	vegetable gardens
high	29	2	vegetable gardens
high	30	2	vegetable gardens
high	21	4	vegetable gardens
high	30	6	vegetable gardens
high	30	9	vegetable gardens
high	20	0	vegetable gardens
high	29	0	vegetable gardens
high	30	0	vegetable gardens
high	30	0	vegetable gardens
high	30	0	vegetable gardens
high	15	1	vegetable gardens
high	21	1	vegetable gardens
high	30	1	vegetable gardens
high	30	2	vegetable gardens
high	30	2	vegetable gardens
high	15	0	vegetable gardens
high	20	0	vegetable gardens
high	21	0	vegetable gardens
high	29	0	vegetable gardens
high	30	0	vegetable gardens
high	30	0	vegetable gardens
high	30	1	vegetable gardens
high	30	1	vegetable gardens
high	30	2	vegetable gardens
high	30	3	vegetable gardens
low	60	0	markets
low	60	0	markets
low	60	0	markets
low	60	0	markets
low	60	0	markets
low	60	0	markets
low	60	0	markets
low	60	0	markets
low	60	0	markets
low	60	0	markets

low	60	0	markets
low	60	0	markets
low	60	0	markets
low	60	0	markets
low	60	0	markets
low	60	0	markets
low	60	0	markets
low	60	0	markets
low	60	0	markets
low	60	0	markets
medium	23	3	markets
medium	23	1	markets
medium	23	6	markets
medium	23	2	markets
medium	23	8	markets
medium	23	10	markets
medium	23	4	markets
medium	23	2	markets
high	10	0	fairs
high	10	0	fairs
high	30	1	fairs
high	80	10	fairs
high	10	0	vegetable gardens
high	10	1	vegetable gardens
high	80	3	vegetable gardens
high	30	4	vegetable gardens
very high	2	0	vegetable gardens
very high	2	0	vegetable gardens
very high	3	0	vegetable gardens
very high	6	0	vegetable gardens
very high	13	0	vegetable gardens
very high	35	0	vegetable gardens
very high	40	0	vegetable gardens
very high	81	0	vegetable gardens
very high	86	0	vegetable gardens
very high	6	0	vegetable gardens
very high	7	0	vegetable gardens
very high	7	0	vegetable gardens
very high	10	0	vegetable gardens
very high	16	0	vegetable gardens
very high	38	0	vegetable gardens
very high	62	0	vegetable gardens
very high	125	5	vegetable gardens
very high	149	14	vegetable gardens
very high	1	0	vegetable gardens
very high	2	0	vegetable gardens
very high	2	0	vegetable gardens
very high	8	0	vegetable gardens
very high	8	0	vegetable gardens
very high	13	0	vegetable gardens
very high	16	0	vegetable gardens
very high	30	0	vegetable gardens
very high	7	0	vegetable gardens
very high	10	0	vegetable gardens
very high	16	0	vegetable gardens
very high	38	0	vegetable gardens

very high	6	1	vegetable gardens
very high	7	2	vegetable gardens
very high	62	2	vegetable gardens
very high	125	2	vegetable gardens
very high	149	3	vegetable gardens
high	100	2	multiple locations
high	100	8	multiple locations
high	100	15	multiple locations
high	100	19	multiple locations
high	100	22	multiple locations
high	100	28	multiple locations
high	100	4	multiple locations
high	100	4	multiple locations
high	100	9	multiple locations
high	100	11	multiple locations
high	100	1	multiple locations
high	100	5	multiple locations
high	100	14	multiple locations
high	100	6	multiple locations
high	100	12	multiple locations
high	100	4	multiple locations
high	100	8	multiple locations
high	100	12	multiple locations
high	100	16	multiple locations
very high	44	1	vegetable gardens
very high	44	1	vegetable gardens
very high	44	1	vegetable gardens
very high	44	1	vegetable gardens
very high	44	1	vegetable gardens
very high	44	1	multiple locations
high	9	0	markets
high	9	0	markets
high	10	0	markets
high	12	0	markets
high	18	0	markets
high	18	0	markets
high	20	0	markets
high	30	0	markets
high	30	0	markets
high	32	0	markets
high	6	2	markets
high	11	2	markets
high	29	4	markets
high	34	4	markets
high	49	8	markets
high	10	0	markets
high	12	0	markets
high	6	1	markets
high	9	1	markets
high	9	1	markets
high	18	2	markets
high	30	2	markets
high	18	4	markets
high	20	4	markets

high	11	6	markets
high	30	7	markets
high	29	8	markets
high	32	16	markets
high	34	32	markets
high	49	38	markets
high	6	0	markets
high	9	0	markets
high	9	0	markets
high	10	0	markets
high	11	0	markets
high	12	0	markets
high	18	0	markets
high	18	0	markets
high	20	0	markets
high	29	0	markets
high	30	0	markets
high	30	0	markets
high	32	0	markets
high	34	0	markets
high	49	4	markets
high	6	0	markets
high	9	0	markets
high	9	0	markets
high	10	0	markets
high	12	0	markets
high	18	0	markets
high	18	0	markets
high	30	0	markets
high	30	0	markets
high	20	2	markets
high	29	2	markets
high	32	2	markets
high	11	4	markets
high	34	4	markets
high	49	10	markets
high	6	0	markets
high	9	0	markets
high	9	0	markets
high	10	0	markets
high	12	0	markets
high	18	0	markets
high	18	0	markets
high	29	0	markets
high	30	0	markets
high	11	2	markets
high	20	2	markets
high	30	4	markets
high	32	10	markets
high	49	12	markets
high	34	18	markets

low	180	45	markets
low	180	20	markets
low	180	26	markets
low	180	13	markets
low	180	2	markets
low	180	44	markets
low	180	8	markets
high	90	1	vegetable gardens
high	90	3	vegetable gardens
high	90	6	vegetable gardens
high	82	7	vegetable gardens
high	54	8	vegetable gardens
high	90	8	vegetable gardens
high	15	1	markets
high	15	1	markets
high	15	1	markets
high	15	1	markets
high	15	1	markets
high	15	2	markets
high	15	2	markets
high	15	1	markets
high	15	1	markets
high	15	2	markets
high	15	1	markets
high	15	1	markets
high	15	1	markets
high	15	1	markets
high	15	1	markets
high	15	2	markets
high	15	3	markets
high	15	3	markets
high	15	5	markets
medium	50	2	multiple locations
medium	50	4	multiple locations
medium	50	5	multiple locations
medium	50	5	multiple locations
medium	50	5	multiple locations
medium	50	6	multiple locations
medium	50	7	multiple locations
medium	50	8	multiple locations
medium	50	8	multiple locations
medium	50	8	multiple locations
medium	50	0	multiple locations
medium	50	0	multiple locations
medium	50	1	multiple locations
medium	50	1	multiple locations
medium	50	1	multiple locations
medium	50	1	multiple locations
medium	50	1	multiple locations
medium	50	2	multiple locations
medium	50	2	multiple locations
medium	50	2	multiple locations
medium	50	1	multiple locations

medium	50	1	multiple locations
medium	50	0	multiple locations
medium	50	0	multiple locations
medium	50	0	multiple locations
medium	50	0	multiple locations
medium	50	0	multiple locations
medium	50	1	multiple locations
medium	50	1	multiple locations
medium	50	1	multiple locations
medium	50	1	multiple locations
medium	50	1	multiple locations
medium	50	0	multiple locations
medium	50	0	multiple locations
medium	50	1	multiple locations
medium	50	1	multiple locations
medium	50	1	multiple locations
medium	50	1	multiple locations
medium	50	1	multiple locations
medium	50	1	multiple locations
medium	50	0	multiple locations
medium	50	0	multiple locations
medium	50	0	multiple locations
medium	50	0	multiple locations
medium	50	1	multiple locations
medium	50	1	multiple locations
medium	50	1	multiple locations
medium	50	1	multiple locations
medium	50	1	multiple locations
medium	50	2	multiple locations
medium	50	0	multiple locations
medium	50	1	multiple locations
medium	50	1	multiple locations
medium	50	1	multiple locations
medium	50	1	multiple locations
medium	50	2	multiple locations
medium	50	2	multiple locations
medium	50	3	multiple locations
medium	50	3	multiple locations
medium	50	3	multiple locations
medium	50	1	multiple locations
medium	50	1	multiple locations
medium	50	1	multiple locations
medium	50	0	multiple locations
medium	50	1	multiple locations
medium	50	1	multiple locations
medium	50	1	multiple locations
medium	50	1	multiple locations
medium	50	1	multiple locations
medium	50	1	multiple locations
medium	50	0	multiple locations
medium	50	0	multiple locations

medium	50	1	multiple locations
medium	50	1	multiple locations
medium	50	1	multiple locations
medium	50	1	multiple locations
medium	50	1	multiple locations
medium	50	1	multiple locations
medium	50	1	multiple locations
medium	50	1	multiple locations
medium	50	0	multiple locations
medium	50	0	multiple locations
medium	50	0	multiple locations
medium	50	0	multiple locations
medium	50	0	multiple locations
medium	50	1	multiple locations
medium	50	1	multiple locations
medium	50	1	multiple locations
medium	50	1	multiple locations
medium	50	2	multiple locations
medium	50	0	multiple locations
medium	50	0	multiple locations
medium	50	0	multiple locations
medium	50	0	multiple locations
medium	50	0	multiple locations
medium	50	0	multiple locations
medium	50	0	multiple locations
medium	50	1	multiple locations
medium	50	1	multiple locations
medium	50	1	multiple locations
medium	50	0	multiple locations
medium	50	0	multiple locations
medium	50	1	multiple locations
medium	50	1	multiple locations
medium	50	2	multiple locations
medium	50	2	multiple locations
medium	50	2	multiple locations
medium	50	2	multiple locations
medium	50	2	multiple locations
medium	50	2	multiple locations
medium	50	0	multiple locations
medium	50	0	multiple locations
medium	50	0	multiple locations
medium	50	0	multiple locations
medium	50	1	multiple locations
medium	50	1	multiple locations
medium	50	1	multiple locations
medium	50	2	multiple locations
medium	50	2	multiple locations
medium	50	3	multiple locations
high	15	0	markets
high	17	0	markets
high	18	0	markets
high	19	0	markets

high	21	0	markets
high	23	0	markets
high	24	0	markets
high	29	0	markets
high	22	2	markets
high	30	3	markets
high	18	0	markets
high	19	0	markets
high	22	0	markets
high	23	0	markets
high	24	0	markets
high	29	0	markets
high	30	0	markets
high	15	1	markets
high	17	1	markets
high	21	1	markets
high	15	0	markets
high	18	0	markets
high	21	0	markets
high	23	0	markets
high	29	0	markets
high	30	0	markets
high	17	1	markets
high	19	1	markets
high	22	2	markets
high	24	2	markets
high	15	0	markets
high	17	0	markets
high	19	0	markets
high	21	0	markets
high	22	0	markets
high	23	0	markets
high	24	0	markets
high	29	0	markets
high	18	1	markets
high	30	2	markets
high	30	2	markets
high	15	0	markets
high	17	0	markets
high	18	0	markets
high	19	0	markets
high	23	0	markets
high	24	0	markets
high	29	0	markets
high	21	1	markets
high	22	3	markets
high	15	0	markets
high	17	0	markets
high	18	0	markets
high	19	0	markets
high	21	0	markets

high	22	0	markets
high	23	0	markets
high	24	0	markets
high	29	1	markets
high	30	1	markets
high	15	0	markets
high	17	0	markets
high	18	0	markets
high	19	0	markets
high	22	0	markets
high	24	0	markets
high	29	0	markets
high	30	0	markets
high	21	2	markets
high	23	2	markets
high	15	0	markets
high	18	0	markets
high	19	0	markets
high	21	0	markets
high	23	0	markets
high	24	0	markets
high	29	0	markets
high	22	1	markets
high	17	2	markets
high	30	3	markets
high	15	0	markets
high	17	0	markets
high	18	0	markets
high	19	0	markets
high	22	0	markets
high	24	0	markets
high	29	0	markets
high	30	0	markets
high	21	1	markets
high	23	1	markets
high	5	0	vegetable gardens
high	5	0	markets
high	5	1	markets
high	5	0	vegetable gardens
high	5	5	markets
high	5	5	markets
high	5	0	vegetable gardens
high	5	1	markets
high	5	2	markets
high	5	0	vegetable gardens
high	5	0	markets
high	5	2	markets
very high	72	1	markets
very high	44	3	markets
very high	70	4	markets
very high	72	5	markets
very high	73	5	markets
very high	73	13	markets
very high	44	1	markets
very high	72	1	markets

very high	73	1	markets
very high	70	2	markets
high	14	0	vegetable gardens
high	25	0	vegetable gardens
high	65	1	vegetable gardens
high	62	2	vegetable gardens
high	14	0	vegetable gardens
high	25	0	vegetable gardens
high	65	1	vegetable gardens
high	62	2	vegetable gardens
high	25	1	vegetable gardens
high	25	1	vegetable gardens
high	14	4	vegetable gardens
high	14	4	vegetable gardens
high	62	4	vegetable gardens
high	62	4	vegetable gardens
high	65	6	vegetable gardens
high	65	6	vegetable gardens
high	25	0	vegetable gardens
high	25	0	vegetable gardens
high	62	0	vegetable gardens
high	62	0	vegetable gardens
high	14	1	vegetable gardens
high	14	1	vegetable gardens
high	65	4	vegetable gardens
high	65	4	vegetable gardens
high	14	0	vegetable gardens
high	14	0	vegetable gardens
high	25	0	vegetable gardens
high	25	0	vegetable gardens
high	62	0	vegetable gardens
high	62	0	vegetable gardens
high	65	1	vegetable gardens
high	65	1	vegetable gardens
high	20	0	vegetable gardens
high	20	0	vegetable gardens
high	20	0	vegetable gardens
high	20	0	vegetable gardens
high	20	0	vegetable gardens
high	20	0	vegetable gardens
high	20	1	vegetable gardens
high	20	1	vegetable gardens
high	20	1	vegetable gardens
high	20	1	vegetable gardens
high	20	0	vegetable gardens
high	20	0	vegetable gardens
high	20	1	vegetable gardens
high	20	1	vegetable gardens
high	20	2	vegetable gardens
high	20	2	vegetable gardens
high	20	2	vegetable gardens

high	20	3	vegetable gardens
high	20	3	vegetable gardens
high	20	4	vegetable gardens
high	20	0	vegetable gardens
high	20	0	vegetable gardens
high	20	0	vegetable gardens
high	20	0	vegetable gardens
high	20	0	vegetable gardens
high	20	0	vegetable gardens
high	20	0	vegetable gardens
high	20	1	vegetable gardens
high	20	1	vegetable gardens
high	20	1	vegetable gardens
high	20	0	vegetable gardens
high	20	0	vegetable gardens
high	20	0	vegetable gardens
high	20	0	vegetable gardens
high	20	0	vegetable gardens
high	20	0	vegetable gardens
high	20	1	vegetable gardens
high	20	1	vegetable gardens
high	20	1	vegetable gardens
high	20	1	vegetable gardens
high	20	0	vegetable gardens
high	20	1	vegetable gardens
high	20	1	vegetable gardens
high	20	1	vegetable gardens
high	20	1	vegetable gardens
high	20	1	vegetable gardens
high	20	2	vegetable gardens
high	20	4	vegetable gardens
high	20	5	vegetable gardens
high	20	5	vegetable gardens
high	20	0	vegetable gardens
high	20	0	vegetable gardens
high	20	0	vegetable gardens
high	20	0	vegetable gardens
high	20	0	vegetable gardens
high	20	0	vegetable gardens
high	20	0	vegetable gardens
high	20	0	vegetable gardens
high	20	1	vegetable gardens
high	20	1	vegetable gardens
high	20	1	vegetable gardens
high	20	2	vegetable gardens
high	20	2	vegetable gardens
high	20	2	vegetable gardens
high	20	2	vegetable gardens
high	20	3	vegetable gardens
high	20	3	vegetable gardens
high	20	3	vegetable gardens
high	20	3	vegetable gardens
high	20	3	vegetable gardens
high	20	0	vegetable gardens

high	20	0	vegetable gardens
high	20	0	vegetable gardens
high	20	0	vegetable gardens
high	20	0	vegetable gardens
high	20	0	vegetable gardens
high	20	1	vegetable gardens
high	20	1	vegetable gardens
high	20	1	vegetable gardens
high	20	2	vegetable gardens
high	20	0	vegetable gardens
high	20	0	vegetable gardens
high	20	0	vegetable gardens
high	20	0	vegetable gardens
high	20	0	vegetable gardens
high	20	0	vegetable gardens
high	20	0	vegetable gardens
high	20	1	vegetable gardens
high	20	1	vegetable gardens
high	20	1	vegetable gardens
high	20	0	vegetable gardens
high	20	0	vegetable gardens
high	20	0	vegetable gardens
high	20	0	vegetable gardens
high	20	0	vegetable gardens
high	20	0	vegetable gardens
high	20	0	vegetable gardens
high	20	1	vegetable gardens
high	20	1	vegetable gardens
high	20	1	vegetable gardens
high	20	0	vegetable gardens
high	20	0	vegetable gardens
high	20	0	vegetable gardens
high	20	0	vegetable gardens
high	20	0	vegetable gardens
high	20	1	vegetable gardens
high	20	1	vegetable gardens
high	20	1	vegetable gardens
high	20	1	vegetable gardens
high	20	2	vegetable gardens
high	250	0	multiple locations
high	250	4	multiple locations
high	250	16	multiple locations
high	250	32	multiple locations
high	250	52	multiple locations
high	250	62	multiple locations
high	250	32	multiple locations
high	250	35	multiple locations
high	250	41	multiple locations
high	250	186	multiple locations
high	250	190	multiple locations

high	250	236	multiple locations
high	250	0	multiple locations
high	250	0	multiple locations
high	250	1	multiple locations
high	250	23	multiple locations
high	250	30	multiple locations
high	250	31	multiple locations
high	250	18	multiple locations
high	250	22	multiple locations
high	250	56	multiple locations
high	250	0	multiple locations
high	250	2	multiple locations
high	250	2	multiple locations
high	250	6	multiple locations
high	250	12	multiple locations
high	250	13	multiple locations
high	250	2	multiple locations
high	250	6	multiple locations
high	250	13	multiple locations
high	250	8	multiple locations
high	250	11	multiple locations
high	250	18	multiple locations
high	250	0	multiple locations
high	250	0	multiple locations
high	250	1	multiple locations
low	6	0	vegetable gardens
low	9	0	vegetable gardens
low	9	2	vegetable gardens
low	12	3	vegetable gardens
low	8	4	vegetable gardens
low	12	6	vegetable gardens
high	16	0	markets
high	16	0	markets
high	16	2	markets
high	16	8	markets
high	16	10	markets
high	16	12	markets
high	16	14	markets
high	16	14	markets
high	16	14	markets
high	16	16	markets
high	16	8	markets
high	16	16	markets
high	16	14	markets
high	16	10	markets
high	16	14	markets
very high	648	3	markets
very high	648	6	markets
very high	648	8	markets
very high	648	1	markets
very high	648	4	markets
very high	648	5	markets

very high	1	0	vegetable gardens
very high	1	0	vegetable gardens
very high	1	0	vegetable gardens
very high	1	0	vegetable gardens
very high	1	0	vegetable gardens
very high	1	0	vegetable gardens
very high	1	0	vegetable gardens
very high	1	0	vegetable gardens
very high	1	0	vegetable gardens
very high	1	0	vegetable gardens
very high	2	0	vegetable gardens
very high	2	0	vegetable gardens
very high	2	0	vegetable gardens
very high	3	0	vegetable gardens
very high	3	0	vegetable gardens
very high	3	0	vegetable gardens
very high	5	0	vegetable gardens
very high	8	0	vegetable gardens
very high	3	1	vegetable gardens
very high	4	1	vegetable gardens
very high	4	1	vegetable gardens
very high	6	1	vegetable gardens
very high	7	1	vegetable gardens
very high	12	1	vegetable gardens
very high	1	0	vegetable gardens
very high	3	0	vegetable gardens
very high	1	1	vegetable gardens
very high	3	1	vegetable gardens
very high	4	1	vegetable gardens
very high	4	1	vegetable gardens
very high	4	1	vegetable gardens
very high	7	1	vegetable gardens
very high	7	1	vegetable gardens
very high	12	1	vegetable gardens
very high	7	2	vegetable gardens
very high	4	3	vegetable gardens
very high	1	0	vegetable gardens
very high	3	1	vegetable gardens
very high	4	1	vegetable gardens
very high	4	1	vegetable gardens
very high	6	1	vegetable gardens
high	26	4	vegetable gardens
high	26	4	vegetable gardens
high	34	3	markets
high	34	2	markets
high	34	3	markets
high	34	1	markets
high	34	1	markets
high	34	1	markets
high	34	1	markets

high	34	3	markets
high	34	1	markets
high	12	1	multiple locations
high	24	1	multiple locations
high	4	2	multiple locations
high	12	2	multiple locations
high	12	2	multiple locations
high	12	4	multiple locations
high	52	4	multiple locations
high	6	1	vegetable gardens
high	13	1	vegetable gardens
high	48	5	vegetable gardens
high	48	6	vegetable gardens
high	36	3	vegetable gardens
high	20	1	markets
high	31	3	markets
high	48	4	markets
high	28	0	markets
high	48	5	markets
medium	284	17	multiple locations
medium	284	13	multiple locations
very high	115	0	vegetable gardens
very high	118	3	vegetable gardens
very high	110	6	vegetable gardens
very high	90	7	vegetable gardens
very high	19	0	markets
very high	20	0	markets
very high	39	0	markets
very high	112	0	markets
very high	115	0	vegetable gardens
very high	110	2	vegetable gardens
very high	90	5	vegetable gardens
very high	118	5	vegetable gardens
very high	19	0	markets
very high	20	0	markets
very high	39	1	markets
very high	112	1	markets
low	16	0	markets
low	38	0	markets
low	42	0	markets
low	35	1	markets
low	68	2	markets
low	133	2	markets
low	82	3	markets
low	86	4	markets
low	89	4	markets
low	187	4	markets
low	16	0	markets
low	38	1	markets
low	68	2	markets
low	35	3	markets
low	42	3	markets

low	187	3	markets
low	86	4	markets
low	89	7	markets
low	133	7	markets
low	82	13	markets
low	16	0	markets
low	35	0	markets
low	38	0	markets
low	42	0	markets
low	68	0	markets
low	89	0	markets
low	133	0	markets
low	187	0	markets
low	86	2	markets
low	82	3	markets
low	16	0	markets
low	35	0	markets
low	68	0	markets
low	82	0	markets
low	86	0	markets
low	89	0	markets
low	187	0	markets
low	38	1	markets
low	42	1	markets
low	133	4	markets
low	16	0	markets
low	38	0	markets
low	82	0	markets
low	86	0	markets
low	89	0	markets
low	133	0	markets
low	187	0	markets
low	35	1	markets
low	42	1	markets
low	68	1	markets
low	16	0	markets
low	35	0	markets
low	38	0	markets
low	42	0	markets
low	82	0	markets
low	133	0	markets
low	187	0	markets
low	89	1	markets
low	68	3	markets
low	86	3	markets
low	16	0	markets
low	35	0	markets
low	38	0	markets
low	42	0	markets
low	133	1	markets

low	68	2	markets
low	86	3	markets
low	89	4	markets
low	187	5	markets
low	82	9	markets
medium	33	0	markets
medium	38	0	markets
medium	41	0	markets
medium	43	0	markets
medium	32	1	markets
medium	43	1	markets
medium	44	1	markets
medium	47	1	markets
medium	48	1	markets
medium	31	2	markets
medium	38	2	markets
low	44	2	markets
medium	38	4	markets
medium	41	0	markets
medium	44	0	markets
medium	32	1	markets
medium	33	1	markets
medium	38	1	markets
medium	38	1	markets
medium	38	1	markets
medium	43	1	markets
medium	43	1	markets
medium	31	2	markets
medium	44	2	markets
medium	47	2	markets
medium	48	3	markets
medium	38	0	markets
medium	48	0	markets
medium	32	1	markets
medium	33	1	markets
medium	38	1	markets
medium	38	1	markets
medium	41	1	markets
medium	43	1	markets
medium	43	1	markets
medium	44	1	markets
medium	47	1	markets
medium	44	2	markets
medium	31	3	markets
medium	31	0	markets
medium	32	0	markets
medium	41	0	markets
medium	48	0	markets
medium	33	1	markets
medium	38	1	markets

medium	38	1	markets
medium	43	1	markets
medium	44	1	markets
medium	44	1	markets
medium	47	1	markets
medium	38	2	markets
medium	43	2	markets
high	12	1	markets
high	31	4	markets
high	17	1	markets
high	29	1	markets
high	98	1	markets
high	152	1	markets
high	35	2	markets
high	49	2	markets
high	62	3	markets
high	25	1	markets
high	29	1	markets
high	31	1	markets
high	17	3	markets
high	152	4	markets
high	17	5	markets
high	35	5	markets
high	49	6	markets
high	62	11	markets
high	98	34	markets
high	7	0	fairs
high	7	0	fairs
high	8	1	fairs
high	8	2	fairs
high	12	2	fairs
high	9	4	fairs
high	7	5	fairs
high	8	6	fairs
high	7	0	markets
high	7	1	markets
high	7	1	markets
high	10	1	markets
high	7	3	markets
high	11	3	markets
high	10	4	markets
high	8	5	markets
high	7	5	fairs
high	8	6	fairs
high	7	1	markets
high	10	4	markets
high	8	1	fairs
high	8	2	fairs
high	12	2	fairs
high	9	4	fairs

high	7	5	fairs
high	8	6	fairs
high	7	3	markets
high	8	5	markets
high	8	1	fairs
high	7	5	fairs
high	8	6	fairs
high	8	2	fairs
high	7	1	markets
high	7	3	markets
high	10	4	markets
high	8	5	markets
high	12	2	fairs
high	9	4	fairs
high	7	5	fairs
high	8	6	fairs
high	10	1	markets
high	7	3	markets
high	11	3	markets
high	8	5	markets
high	7	5	fairs
high	8	6	fairs
high	7	1	markets
high	7	3	markets
high	11	3	markets
high	8	5	markets
high	98	30	markets
high	98	4	markets
high	98	11	markets
high	98	1	markets
high	98	3.00	markets
high	98	6	markets
high	98	5	markets
high	98	19	markets
high	98	19	markets
high	98	3	markets

CULTIVATION TYPE	YEAR OF STUDY
not indicated	2017-2018
not indicated	2017-2018
not indicated	2017-2018
not indicated	2017-2018
not indicated	2017-2018
not indicated	2017-2018
not indicated	2017-2018
not indicated	2017-2018
not indicated	2017-2018
not indicated	2004
not indicated	2004
not indicated	2004
not indicated	2004
not indicated	2004
not indicated	2004
not indicated	2004
not indicated	2004
not indicated	2004
not indicated	2004
not indicated	2004
not indicated	2004
not indicated	2004
not indicated	2004
not indicated	2014-2015
not indicated	2014-2015
not indicated	2014-2015
not indicated	2014-2015
not indicated	2014-2015
not indicated	2014-2015
not indicated	2014-2015
not indicated	2014-2015
not indicated	2014-2015
not indicated	2014-2015
not indicated	2014-2015
not indicated	2014-2015
not indicated	2014-2015
not indicated	2014-2015
not indicated	2014-2015
not indicated	2014-2015
not indicated	2014-2015
not indicated	2014-2015
not indicated	2014-2015
not indicated	2014-2015
not indicated	2014-2015
not indicated	2014-2015
not indicated	2014-2015
not indicated	2014-2015
not indicated	2014-2015
not indicated	2014-2015

not indicated	2014-2015
not indicated	2014-2015
not indicated	2014-2015
not indicated	2014-2015
not indicated	2014-2015
not indicated	2014-2015
not indicated	2014-2015
not indicated	2014-2015
not indicated	2014-2015
not indicated	2014-2015
not indicated	2014-2015
not indicated	2014-2015
not indicated	2014-2015
not indicated	2014-2015
not indicated	2014-2015
not indicated	2014-2015
not indicated	2014-2015
not indicated	2014-2015
not indicated	2014-2015
not indicated	2014-2015
not indicated	2014-2015
not indicated	2014-2015
not indicated	2014-2015
not indicated	2014-2015
not indicated	2014-2015
not indicated	2014-2015
not indicated	2014-2015
not indicated	2014-2015
not indicated	2014-2015
not indicated	2014-2015
not indicated	2014-2015
not indicated	2014-2015
not indicated	2014-2015
not indicated	2014-2015
not indicated	2014-2015
not indicated	2014-2015
not indicated	2014-2015
not indicated	2014-2015
not indicated	2014-2015
not indicated	2014-2015
not indicated	2014-2015
not indicated	2014-2015
not indicated	2014-2015
not indicated	2014-2015
not indicated	2014-2015
not indicated	2014-2015
not indicated	2014-2015
not indicated	2014-2015
not indicated	2014-2015
not indicated	2014-2015
not indicated	2014-2015
not indicated	2014-2015
not indicated	2014-2015
not indicated	2014-2015
not indicated	2014-2015
not indicated	2014-2015
not indicated	2014-2015
not indicated	2014-2015
not indicated	2014-2015

not indicated	2014-2015
not indicated	2014-2015
not indicated	2014-2015
not indicated	2014-2015
not indicated	2014-2015
not indicated	2014-2015
not indicated	2014-2015
not indicated	2014-2015
not indicated	2014-2015
not indicated	2014-2015
not indicated	2014-2015
not indicated	2014-2015
hydroponic	not indicated
hydroponic	not indicated
hydroponic	not indicated
hydroponic	not indicated
hydroponic	not indicated
hydroponic	not indicated
hydroponic	not indicated
hydroponic	not indicated
hydroponic	not indicated
hydroponic	not indicated
hydroponic	not indicated
hydroponic	not indicated
not indicated	2019
not indicated	2019
not indicated	2019
not indicated	2019
not indicated	2019
not indicated	2019
not indicated	2019
not indicated	2019
not indicated	2019
not indicated	2019
not indicated	2019
not indicated	2019
not indicated	2019
not indicated	2019
not indicated	2019
not indicated	2019
not indicated	2019
not indicated	2019
not indicated	2019
not indicated	2019
not indicated	2019
not indicated	2019
not indicated	2019
not indicated	2019
not indicated	2019
not indicated	2019
not indicated	2019
not indicated	2019
not indicated	2019
not indicated	2019
not indicated	2019
not indicated	2019
not indicated	2019
not indicated	2019
not indicated	2019
not indicated	2019

not indicated	2019
not indicated	2019
not indicated	2019
not indicated	2019
not indicated	2019
not indicated	2019
not indicated	2019
not indicated	2019
not indicated	2019
not indicated	2019
not indicated	2019
not indicated	2019
not indicated	2019
not indicated	2019
not indicated	2019
not indicated	2019
not indicated	2019
not indicated	2019
not indicated	2019
not indicated	2019
not indicated	2019
not indicated	2019
not indicated	2019
not indicated	2019
not indicated	2019
not indicated	2019
not indicated	2019
not indicated	2019
not indicated	2019
not indicated	2019
not indicated	2019
not indicated	2019
not indicated	2019
not indicated	2019
not indicated	2019
not indicated	2019
not indicated	2019
not indicated	2019
not indicated	2019
not indicated	2019
not indicated	2019
not indicated	2019
not indicated	2019
not indicated	2019
not indicated	2019
not indicated	2019
not indicated	2019
not indicated	2019
not indicated	2019
not indicated	2019
not indicated	2019
not indicated	2019
not indicated	2019

not indicated	2019
not indicated	2019
not indicated	2019
not indicated	2019
not indicated	2019
not indicated	2019
not indicated	2019
not indicated	2019
not indicated	2019
not indicated	2019
not indicated	2019
not indicated	2019
not indicated	2019
not indicated	2019
not indicated	2019
not indicated	2019
not indicated	2019
not indicated	2019
not indicated	2019
not indicated	2019
not indicated	2019
not indicated	2019
not indicated	2019
not indicated	2019
not indicated	2019
not indicated	2019
not indicated	2019
not indicated	2019
not indicated	2019
not indicated	2019
not indicated	2019
not indicated	2019
not indicated	2019
not indicated	2019
not indicated	2019
not indicated	2019
not indicated	2019
not indicated	2019
not indicated	2019
not indicated	2019
not indicated	2019
not indicated	2019
not indicated	2019
not indicated	2019
not indicated	2019
not indicated	2019
not indicated	2019
not indicated	2019
not indicated	2019
not indicated	2019
not indicated	2019
not indicated	2019

not indicated	2019
not indicated	2019
not indicated	2019
not indicated	2019
not indicated	2019
not indicated	2019
not indicated	2019
not indicated	2019
not indicated	2019
not indicated	2019
not indicated	2019
not indicated	2019
not indicated	2019
not indicated	2019
not indicated	2019
not indicated	2019
not indicated	2019
not indicated	2019
not indicated	2019
not indicated	2019
not indicated	2019
not indicated	2019
not indicated	2019
not indicated	2019
not indicated	2019
not indicated	2019
not indicated	2019
not indicated	2019
not indicated	2019
not indicated	2019
not indicated	2019
not indicated	2019
not indicated	2019
not indicated	2019
not indicated	2019
not indicated	2019
not indicated	2019
not indicated	2019
not indicated	2019
not indicated	2019
not indicated	2019
not indicated	2019
not indicated	2019
not indicated	2019
not indicated	2019
not indicated	2019
not indicated	2019
not indicated	2019
not indicated	2019
not indicated	2019
not indicated	2019
not indicated	not indicated

not indicated	not indicated
not indicated	not indicated
not indicated	not indicated
not indicated	not indicated
not indicated	not indicated
not indicated	not indicated
not indicated	not indicated
not indicated	not indicated
not indicated	not indicated
not indicated	not indicated
not indicated	not indicated
not indicated	not indicated
not indicated	not indicated
not indicated	not indicated
not indicated	not indicated
not indicated	not indicated
not indicated	not indicated
not indicated	not indicated
not indicated	not indicated
not indicated	not indicated
not indicated	not indicated
not indicated	not indicated
not indicated	not indicated
not indicated	not indicated
not indicated	not indicated
not indicated	not indicated
not indicated	not indicated
not indicated	not indicated
not indicated	not indicated
not indicated	not indicated
not indicated	not indicated
not indicated	not indicated
not indicated	not indicated
not indicated	not indicated
not indicated	not indicated
not indicated	not indicated
not indicated	not indicated
not indicated	not indicated
not indicated	not indicated
not indicated	not indicated
not indicated	not indicated
not indicated	not indicated
not indicated	not indicated
not indicated	not indicated
not indicated	not indicated
not indicated	not indicated
not indicated	not indicated
not indicated	not indicated
not indicated	2013
not indicated	2013
not indicated	2013

not indicated	2013
not indicated	2013
not indicated	2013
not indicated	2013
not indicated	2013
not indicated	2013
not indicated	2013
not indicated	2013
not indicated	2013
not indicated	2013
not indicated	2013
not indicated	not indicated
not indicated	not indicated
not indicated	not indicated
not indicated	not indicated
not indicated	not indicated
not indicated	not indicated
not indicated	not indicated
not indicated	not indicated
not indicated	not indicated
not indicated	not indicated
not indicated	not indicated
not indicated	not indicated
not indicated	not indicated
not indicated	not indicated
not indicated	not indicated
not indicated	not indicated
not indicated	not indicated
not indicated	not indicated
not indicated	not indicated
not indicated	not indicated
not indicated	not indicated
not indicated	not indicated
not indicated	not indicated
not indicated	not indicated
not indicated	not indicated
not indicated	not indicated
not indicated	not indicated
not indicated	not indicated
not indicated	not indicated
not indicated	not indicated
not indicated	not indicated
not indicated	not indicated
not indicated	not indicated
not indicated	not indicated
not indicated	not indicated
not indicated	not indicated
not indicated	not indicated
not indicated	not indicated
not indicated	not indicated
not indicated	not indicated

not indicated	not indicated
not indicated	2005-2006
not indicated	2005-2006
not indicated	2005-2006
not indicated	2005-2006
not indicated	2005-2006
not indicated	2005-2006
not indicated	2005-2006
not indicated	2005-2006
not indicated	2005-2006
not indicated	2005-2006
not indicated	2005-2006
not indicated	2005-2006
not indicated	2005-2006
not indicated	2005-2006
not indicated	2005-2006
not indicated	2005-2006
not indicated	2005-2006
not indicated	2005-2006
not indicated	2005-2006
not indicated	2005-2006
not indicated	2005-2006
not indicated	2005-2006
not indicated	2005-2006
not indicated	2005-2006
not indicated	2005-2006
not indicated	2005-2006
not indicated	2005-2006
not indicated	2005-2006
not indicated	2005-2006
not indicated	2005-2006
not indicated	2005-2006
not indicated	2005-2006
not indicated	2005-2006
not indicated	2005-2006
not indicated	2005-2006
not indicated	2005-2006
not indicated	2005-2006
not indicated	2005-2006
not indicated	2005-2006
not indicated	2005-2006
not indicated	2010
not indicated	2010
not indicated	2010
not indicated	2010
not indicated	2010
not indicated	2010
not indicated	2010
not indicated	2010
not indicated	2010

not indicated	not indicated
not indicated	not indicated
not indicated	not indicated
not indicated	not indicated
not indicated	not indicated
not indicated	not indicated
not indicated	not indicated
not indicated	not indicated
not indicated	not indicated
not indicated	2019
not indicated	2019
not indicated	2019
not indicated	2019
not indicated	2019
not indicated	2019
not indicated	2019
not indicated	2019
not indicated	2019
not indicated	2019
not indicated	2019
not indicated	2019
not indicated	2019
not indicated	2019
not indicated	2019
not indicated	2019
not indicated	2019
not indicated	2019
not indicated	2019
not indicated	2019
not indicated	2019
not indicated	2019
not indicated	2019
not indicated	2019
not indicated	2019
not indicated	2019
not indicated	2019
not indicated	2019
not indicated	2019
not indicated	2019
not indicated	2019
not indicated	2019
not indicated	2019
not indicated	2019
not indicated	2019
not indicated	2019
not indicated	2019
not indicated	2019
not indicated	2019
not indicated	2019
not indicated	2019

not indicated	2019
not indicated	2019
not indicated	2019
not indicated	2019
not indicated	2019
not indicated	2019
not indicated	2019
not indicated	2019
not indicated	2019
not indicated	2019
not indicated	2019
not indicated	2019
not indicated	2019
not indicated	2019
not indicated	2019
not indicated	2004- 2005
not indicated	2004- 2005
not indicated	2004- 2005
not indicated	2004- 2005
not indicated	2004- 2005
not indicated	2004- 2005
not indicated	2004- 2005
not indicated	2004- 2005
not indicated	2004- 2005
not indicated	2004- 2005
not indicated	2004- 2005
not indicated	2004- 2005
not indicated	2004- 2005
not indicated	2004- 2005
not indicated	2004- 2005
not indicated	2004- 2005
not indicated	2004- 2005
not indicated	2004- 2005
not indicated	2004- 2005
not indicated	2004- 2005
not indicated	2004- 2005
not indicated	2004- 2005
not indicated	2004- 2005
not indicated	2004- 2005
not indicated	2004- 2005
not indicated	2004- 2005
not indicated	2004- 2005
not indicated	2004- 2005
not indicated	2004- 2005
not indicated	2004- 2005
not indicated	2004- 2005
not indicated	2004- 2005
not indicated	2018
not indicated	2018
not indicated	2018

not indicated	2018
not indicated	2018
not indicated	2018
not indicated	2018
not indicated	2018
not indicated	2018
not indicated	2018
not indicated	2018
not indicated	2018
not indicated	2018
not indicated	2018
not indicated	2018
not indicated	2018
not indicated	2018
not indicated	2018
not indicated	2018
not indicated	2018
not indicated	2018
not indicated	2018
not indicated	2018
not indicated	2018
not indicated	2018
not indicated	2018
not indicated	2018
not indicated	2018
not indicated	2018
not indicated	2018
not indicated	2018
not indicated	2018
not indicated	2018
not indicated	2018
not indicated	2018
not indicated	2018
not indicated	2018
not indicated	2018
not indicated	2018
not indicated	2018
not indicated	2013-2014
not indicated	2013-2014
not indicated	2013-2014
not indicated	2013-2014
not indicated	2013-2014
not indicated	2013-2014
not indicated	2013-2014
not indicated	2013-2014
not indicated	2013-2014
not indicated	2013-2014
not indicated	2013-2014
not indicated	2013-2014
not indicated	2013-2014

not indicated	2013-2014
not indicated	2013-2014
not indicated	2013-2014
not indicated	2013-2014
not indicated	2013-2014
not indicated	2013-2014
not indicated	2013-2014
not indicated	2017- 2018
not indicated	2017- 2018
not indicated	2017- 2018
not indicated	2017- 2018
not indicated	2017- 2018
not indicated	2017- 2018
not indicated	2017- 2018
not indicated	2017- 2018
not indicated	2017- 2018
not indicated	2017- 2018
not indicated	2017- 2018
not indicated	2017- 2018
not indicated	2017- 2018
not indicated	2017- 2018
not indicated	2017- 2018
not indicated	2017- 2018
not indicated	2017- 2018
not indicated	2017- 2018
not indicated	2017- 2018
not indicated	2017- 2018
not indicated	2017- 2018
not indicated	2017- 2018
not indicated	2017- 2018
not indicated	2017- 2018
not indicated	2017- 2018
not indicated	2017- 2018
not indicated	2017- 2018
not indicated	not indicated
not indicated	not indicated
not indicated	not indicated
not indicated	not indicated
not indicated	not indicated
not indicated	not indicated
not indicated	not indicated
not indicated	not indicated
not indicated	not indicated
not indicated	not indicated
not indicated	not indicated
not indicated	not indicated
not indicated	not indicated
not indicated	not indicated
not indicated	not indicated
not indicated	not indicated

not indicated	not indicated
not indicated	not indicated
not indicated	not indicated
not indicated	not indicated
not indicated	not indicated
not indicated	not indicated
not indicated	not indicated
not indicated	not indicated
not indicated	not indicated
not indicated	not indicated
not indicated	not indicated
not indicated	not indicated
not indicated	not indicated
not indicated	not indicated
not indicated	not indicated
not indicated	not indicated
not indicated	not indicated
not indicated	not indicated
not indicated	not indicated
not indicated	not indicated
not indicated	not indicated
not indicated	not indicated
not indicated	not indicated
not indicated	not indicated
not indicated	2014-2015
not indicated	2014-2015
not indicated	2014-2015
not indicated	2014-2015
not indicated	2014-2015
not indicated	2014-2015
not indicated	2014-2015
not indicated	2014-2015
not indicated	2014-2015
not indicated	2014-2015
not indicated	2014-2015
not indicated	2014-2015
not indicated	2014-2015
not indicated	2014-2015
hydroponic	2004-2005
hydroponic	2004-2005
hydroponic	2004-2005
not indicated	2004-2005
organic	2006
organic	2006
organic	2006
organic	2006
organic	2006
organic	2006
organic	2006
organic	2006

organic	2006
organic	2006
organic	2006
organic	2006
organic	2006
organic	2006
organic	2006
not indicated	2002-2003
not indicated	2002-2003
not indicated	2002-2003
not indicated	2002-2003
not indicated	2002-2003
not indicated	2002-2003
not indicated	2002-2003
not indicated	2002-2003
not indicatedDO	2013
not indicatedDO	2013
not indicatedDO	2013
not indicatedDO	2013
not indicated	2012-2016
not indicated	2012-2016
not indicated	2012-2016
not indicated	2012-2016
not indicated	2012-2016
not indicated	2012-2016
not indicated	2012-2016
not indicated	2012-2016
not indicated	2012-2016
not indicated	2012-2016
not indicated	2012-2016
not indicated	2012-2016
not indicated	2012-2016
not indicated	2012-2016
not indicated	2012-2016
not indicated	2012-2016
not indicated	2012-2016
not indicated	2012-2016
not indicated	2012-2016
not indicated	2012-2016
not indicated	2012-2016
not indicated	2012-2016
not indicated	2012-2016
not indicated	2012-2016
not indicated	2012-2016
not indicated	2012-2016
not indicated	2012-2016
not indicated	2012-2016
not indicated	2012-2016
not indicated	2012-2016
not indicated	2012-2016

not indicated	2012-2016
not indicated	2012-2016
not indicated	2012-2016
not indicated	2012-2016
not indicated	2012-2016
not indicated	2012-2016
not indicated	2012-2016
not indicated	2012-2016
not indicated	2012-2016
not indicated	not indicated
not indicated	not indicated
not indicated	not indicated
not indicated	not indicated
not indicated	not indicated
not indicated	not indicated
not indicated	2012
not indicated	2012
not indicated	2012
not indicated	2012
not indicated	2012
not indicated	2012
not indicated	2012
not indicated	2012
not indicated	2012
not indicated	2012
not indicated	2014
not indicated	2014
not indicated	2014
not indicated	2014
not indicated	2014
not indicated	2014
not indicated	2014
not indicated	2014
not indicated	2014
not indicated	not indicated
not indicated	not indicated
not indicated	not indicated
not indicated	not indicated
not indicated	not indicated
not indicated	not indicated
not indicated	not indicated
not indicated	not indicated
not indicated	not indicated
not indicated	not indicated
not indicated	not indicated
not indicated	not indicated
not indicated	not indicated
not indicated	not indicated
not indicated	not indicated
not indicated	not indicated
not indicated	not indicated
not indicated	not indicated
not indicated	not indicated
not indicated	not indicated
not indicated	not indicated
not indicated	not indicated

not indicated	not indicated
not indicated	not indicated
not indicated	not indicated
not indicated	not indicated
not indicated	not indicated
not indicated	not indicated
not indicated	not indicated
not indicated	not indicated
not indicated	not indicated
not indicated	not indicated
not indicated	not indicated
not indicated	not indicated
not indicated	not indicated
not indicated	not indicated
not indicated	not indicated
not indicated	not indicated
not indicated	not indicated
not indicated	not indicated
not indicated	not indicated
not indicated	not indicated
not indicated	2017-2018
not indicated	2017-2018
not indicated	2017-2018
not indicated	2017-2018
not indicated	2017-2018
not indicated	2017-2018
not indicated	2017-2018
not indicated	2017-2018
not indicated	2017-2018
not indicated	2017-2018
not indicated	2017-2018
conventional	2018-2019
conventional	2018-2019
organic	2018-2019
organic	2018-2019
organic	2018-2019
conventional	2018-2019
conventional	2018-2019
conventional	2018-2019
conventional	2018-2019
conventional	2018-2019
conventional	2018-2019
conventional	2018-2019
conventional	2018-2019
conventional	2018-2019
conventional	2018-2019
organic	2018-2019
conventional	2018-2019
conventional	2018-2019
conventional	2018-2019
conventional	2018-2019

not indicated	2016-2017
not indicated	2016-2017
not indicated	2016-2017
not indicated	2016-2017
not indicated	2016-2017
not indicated	2016-2017
not indicated	2016-2017
not indicated	2016-2017
not indicated	2016-2017
not indicated	2016-2017
not indicated	2016-2017
not indicated	2016-2017
not indicated	2016-2017
not indicated	2016-2017
not indicated	2016-2017
not indicated	2016-2017
not indicated	2016-2017
not indicated	2016-2017
not indicated	2016-2017
not indicated	2016-2017
not indicated	2016-2017
not indicated	2016-2017
not indicated	2016-2017
not indicated	2016-2017
not indicated	2016-2017
not indicated	2016-2017
not indicated	2016-2017
not indicated	2016-2017
not indicated	2016-2017
not indicated	2006
not indicated	2006
not indicated	2006
not indicated	2006
not indicated	2006
not indicated	2006
not indicated	2006
not indicated	2006
not indicated	2006
not indicated	2006
not indicated	2006
not indicated	2006
not indicated	2006
not indicated	2006
not indicated	2006
not indicated	2006
not indicated	2006
not indicated	2006
not indicated	2006
not indicated	2006
not indicated	2006

not indicated	2006
not indicated	2006
not indicated	2006
not indicated	2006
not indicated	2006
not indicated	2006
not indicated	2006
not indicated	2006
not indicated	2006
not indicated	2006
not indicated	2006
not indicated	2006
not indicated	2006
not indicated	2006
not indicated	2006
not indicated	2006
not indicated	2006
not indicated	2006
not indicated	2006
not indicated	2006
not indicated	2006
not indicated	2006
not indicated	2006
not indicated	2006
not indicated	2006
not indicated	2006
not indicated	2006
not indicated	2006
not indicated	2006
not indicated	2006
not indicated	2006
not indicated	2006
not indicated	2006
not indicated	2006
not indicated	2006
not indicated	2006
not indicated	2006
not indicated	2006
not indicated	2006
not indicated	2006
not indicated	2006
not indicated	2006
not indicated	2006
not indicated	2006
not indicated	2006
not indicated	2006
not indicated	2006
not indicated	2006
not indicated	2006
not indicated	2006
not indicated	2006
not indicated	2006

not indicated	2006
not indicated	2006
not indicated	2006
not indicated	2006
not indicated	2006
not indicated	2006
not indicated	2006
not indicated	2006
not indicated	2006
not indicated	2006
not indicated	2006
not indicated	2006
not indicated	2006
not indicated	2006
not indicated	2006
not indicated	2006
not indicated	2006
not indicated	2012-2013
not indicated	2012-2013
not indicated	2010-2011
not indicated	2010-2011
not indicated	2010-2011
not indicated	2010-2011
not indicated	2010-2011
not indicated	2010-2011
not indicated	2010-2011
not indicated	2010-2011
not indicated	2010-2011
not indicated	2010-2011
not indicated	2010-2011
not indicated	2010-2011
not indicated	2010-2011
not indicated	2010-2011
not indicated	2010-2011
not indicated	2010-2011
not indicated	2010-2011
not indicated	2010-2011
not indicated	2010-2011
not indicated	2010-2011
not indicated	2010-2011
not indicated	2010-2011
not indicated	2010-2011
not indicated	2010-2011
not indicated	2010-2011
not indicated	2010-2011
not indicated	2010-2011
not indicated	2010-2011
not indicated	2010-2011
not indicated	2010-2011
not indicated	2010-2011
not indicated	2010-2011
not indicated	2004-2005

not indicated	2019
not indicated	2019
not indicated	2019
not indicated	2019
not indicated	2019
not indicated	2019
not indicated	2019
not indicated	2019
not indicated	2019
not indicated	2019
not indicated	2019
not indicated	2019
not indicated	2009-2010
not indicated	2009-2010
not indicated	2009-2010
not indicated	2012-2013
not indicated	2012-2013
not indicated	2012-2013
not indicated	2012-2013
not indicated	2012-2013
not indicated	2012-2013
not indicated	2012-2013
not indicated	2012-2013
not indicated	2012-2013
not indicated	2012-2013
not indicated	2012-2013
not indicated	2012-2013
not indicated	2012-2013
not indicated	2012-2013
not indicated	2012-2013
not indicated	2012-2013
not indicated	2012-2013
not indicated	2012-2013
not indicated	2012-2013
not indicated	2012-2013
not indicated	2012-2013
not indicated	2012-2013
not indicated	2012-2013
not indicated	2012-2013
not indicated	2012-2013
not indicated	2012-2013
not indicated	2012-2013
not indicated	2012-2013
not indicated	2012-2013
not indicated	2012-2013
hydroponic	2002-2003
hydroponic	2002-2003
hydroponic	2002-2003
hydroponic	2002-2003
not indicated	2002-2003
hydroponic	2002-2003
hydroponic	2002-2003
hydroponic	2002-2003

hydroponic	2002-2003
not indicated	2002-2003
not indicated	2002-2003
not indicated	2002-2003
not indicated	2002-2003
not indicated	2002-2003
not indicated	2002-2003
not indicated	2002-2003
not indicated	2002-2003
not indicated	2002-2003
not indicated	2002-2003
not indicated	2002-2003
not indicated	not indicated
not indicated	not indicated
not indicated	not indicated
not indicated	not indicated
not indicated	not indicated
not indicated	not indicated
not indicated	not indicated
not indicated	not indicated
not indicated	not indicated
not indicated	not indicated
not indicated	not indicated
not indicated	not indicated
not indicated	not indicated
not indicated	not indicated
not indicated	not indicated
not indicated	not indicated
not indicated	not indicated
not indicated	not indicated
not indicated	not indicated
not indicated	not indicated
not indicated	not indicated
not indicated	not indicated
not indicated	not indicated
not indicated	not indicated
not indicated	not indicated
not indicated	not indicated
not indicated	not indicated
not indicated	not indicated
not indicated	not indicated
not indicated	not indicated
not indicated	not indicated
not indicated	not indicated
not indicated	not indicated
not indicated	not indicated
not indicated	not indicated
not indicated	not indicated
not indicated	not indicated
not indicated	not indicated
not indicated	not indicated
not indicated	not indicated
not indicated	2019-2020

not indicated	2019-2020
not indicated	2019-2020
not indicated	2019-2020
not indicated	2019-2020
not indicated	2019-2020
not indicated	2019-2020
not indicated	2010-2011
not indicated	2010-2011
not indicated	2010-2011
not indicated	2010-2011
not indicated	2010-2011
not indicated	2010-2011
not indicated	2010-2011
not indicated	2010-2011
not indicated	2010-2011
not indicated	2010-2011
not indicated	2010-2011
not indicated	2010-2011
not indicated	2010-2011
not indicated	2010-2011
not indicated	2010-2011
not indicated	2010-2011
not indicated	2010-2011
not indicated	2010-2011
not indicated	2010-2011
not indicated	2010-2011
not indicated	2010-2011
not indicated	2010-2011
not indicated	2010-2011
not indicated	2010-2011
not indicated	2010-2011
not indicated	2010-2011
not indicated	2010-2011
not indicated	2010-2011
not indicated	2010-2011
not indicated	2010-2011
not indicated	2010-2011
not indicated	2010-2011
not indicated	2010-2011
not indicated	2010-2011
not indicated	2010-2011
not indicated	2010-2011
not indicated	2010-2011
not indicated	2010-2011
not indicated	2010-2011
not indicated	2010-2011
not indicated	2010-2011
not indicated	2010-2011
not indicated	2010-2011
not indicated	2010-2011
not indicated	2010-2011
not indicated	2010-2011
not indicated	2010-2011
not indicated	2010-2011

not indicated	2010-2011
not indicated	2010-2011
not indicated	2010-2011
not indicated	2010-2011
not indicated	2010-2011
not indicated	2010-2011
not indicated	2010-2011
not indicated	2010-2011
not indicated	2010-2011
not indicated	2010-2011
not indicated	2010-2011
not indicated	2010-2011
not indicated	2010-2011
not indicated	2010-2011
not indicated	2010-2011
not indicated	2010-2011
not indicated	2010-2011
not indicated	2010-2011
organic	2014-2016
organic	2014-2016
organic	2014-2016
organic	2014-2016
organic	2014-2016
organic	2014-2016
organic	2014-2016
organic	2014-2016
organic	2014-2016
organic	2014-2016
not indicated	2014-2015
not indicated	2014-2015
not indicated	2014-2015
not indicated	2014-2015
not indicated	2014-2015
not indicated	2014-2015
not indicated	2014- 2015
not indicated	2014- 2015
not indicated	2014- 2015
not indicated	2014-2015
not indicated	2014-2015
not indicated	2014- 2015
not indicated	2014- 2015
not indicated	2014- 2015
not indicated	2014- 2015
not indicated	2014- 2015
not indicated	2014- 2015
not indicated	2014- 2015
not indicated	2014- 2015
not indicated	2014- 2015
not indicated	2014-2015
not indicated	2014-2015
not indicated	2014-2015
not indicated	2014-2015
not indicated	2014-2015
not indicated	2014-2015
not indicated	2014-2015
not indicated	2014-2015
not indicated	2014-2015
not indicated	2014-2015

not indicated	not indicated
not indicated	not indicated
not indicated	not indicated
not indicated	not indicated
not indicated	not indicated
not indicated	not indicated
not indicated	not indicated
not indicated	not indicated
not indicated	not indicated
not indicated	not indicated
not indicated	not indicated
not indicated	not indicated
not indicated	not indicated
not indicated	not indicated
not indicated	not indicated
not indicated	not indicated
not indicated	not indicated
not indicated	not indicated
not indicated	not indicated
not indicated	not indicated
not indicated	not indicated
not indicated	not indicated
not indicated	not indicated
not indicated	not indicated
not indicated	not indicated
not indicated	not indicated
not indicated	not indicated
not indicated	not indicated
not indicated	not indicated
not indicated	not indicated
not indicated	not indicated
not indicated	not indicated
not indicated	not indicated
not indicated	not indicated
not indicated	not indicated
not indicated	not indicated
not indicated	not indicated
not indicated	not indicated
not indicated	not indicated
not indicated	not indicated
not indicated	not indicated
not indicated	not indicated
not indicated	not indicated
not indicated	not indicated
not indicated	not indicated
not indicated	not indicated
not indicated	not indicated
not indicated	not indicated
not indicated	not indicated
not indicated	not indicated
not indicated	not indicated
not indicated	not indicated
not indicated	not indicated
not indicated	not indicated
not indicated	2011
not indicated	2011
not indicated	2011

not indicated	2011
not indicated	2011
not indicated	2011
not indicated	2011
not indicated	2011
not indicated	2011
not indicated	2011
not indicated	2011
not indicated	2011
not indicated	2011
not indicated	2011
hydroponic	2010-2011
conventional	2010-2011
organic	2010-2011
hydroponic	2010-2011
conventional	2010-2011
organic	2010-2011
hydroponic	2010-2011
conventional	2010-2011
organic	2010-2011
hydroponic	2010-2011
conventional	2010-2011
organic	2010-2011
conventional	2010-2011
hydroponic	2010-2011
organic	2010-2011
conventional	2010-2011
hydroponic	2010-2011
organic	2010-2011
organic	2010-2011
hydroponic	2010-2011
conventional	2010-2011
hydroponic	2010-2011
conventional	2010-2011
organic	2010-2011
hydroponic	2010-2011
conventional	2010-2011
organic	2010-2011
conventional	2010-2011
hydroponic	2010-2011
organic	2010-2011
not indicated	2001-2002
not indicated	2001-2002
not indicated	2001-2002
not indicated	2001-2002
not indicated	2001-2002
not indicated	2001-2002
not indicated	2001-2002
not indicated	2001-2002
not indicated	2001-2002
not indicated	2001-2002
not indicated	2001-2002
not indicated	2001-2002
not indicated	2001-2002
not indicated	2001-2002
not indicated	2001-2002
not indicated	2001-2002
not indicated	2001-2002

not indicated	2001-2002
not indicated	2001-2002
not indicated	2001-2002
not indicated	2001-2002
not indicated	2001-2002
not indicated	2001-2002
not indicated	not indicated
not indicated	not indicated
not indicated	not indicated
not indicated	not indicated
not indicated	not indicated
not indicated	not indicated
not indicated	not indicated
not indicated	not indicated
not indicated	not indicated
not indicated	not indicated
not indicated	not indicated
not indicated	not indicated
not indicated	not indicated
not indicated	not indicated
not indicated	not indicated
not indicated	not indicated
not indicated	not indicated
not indicated	not indicated
not indicated	not indicated
not indicated	not indicated
not indicated	not indicated
not indicated	2012-2013
not indicated	2012-2013
not indicated	2012-2013
not indicated	2012-2013
not indicated	2012-2013
not indicated	2012-2013
not indicated	2012-2013
not indicated	2012-2013
not indicated	2012-2013
not indicated	2012-2013
not indicated	2012-2013
not indicated	2012-2013
not indicated	2012-2013
not indicated	2012-2013
not indicated	2012-2013
not indicated	2012-2013
not indicated	2012-2013
not indicated	2012-2013
not indicated	2012-2013
not indicated	2012-2013
not indicated	2012-2013
not indicated	2012-2013
not indicated	2012-2013
not indicated	2012-2013
not indicated	2012-2013
not indicated	2012-2013
not indicated	2012-2013
not indicated	2012-2013
not indicated	2012-2013
not indicated	2012-2013
not indicated	2012-2013

not indicated	2012-2013
not indicated	2012-2013
not indicated	2012-2013
not indicated	2012-2013
not indicated	2012-2013
not indicated	2012-2013
not indicated	2012-2013
not indicated	2012-2013
not indicated	2012-2013
not indicated	2012-2013
not indicated	2012-2013
not indicated	2012-2013
not indicated	2012-2013
not indicated	2012-2013
not indicated	2012-2013
not indicated	2012-2013
not indicated	2012-2013
not indicated	2012-2013
not indicated	2012-2013
not indicated	2012-2013
not indicated	2012-2013
not indicated	2012-2013
not indicated	2012-2013
not indicated	2012-2013
not indicated	2012-2013
not indicated	2012-2013
not indicated	2012-2013
not indicated	2012-2013
not indicated	2012-2013
not indicated	not indicated
not indicated	not indicated
not indicated	not indicated
not indicated	not indicated
not indicated	not indicated
not indicated	not indicated
not indicated	not indicated
not indicated	not indicated
not indicated	not indicated
not indicated	not indicated
not indicated	not indicated
not indicated	not indicated
not indicated	not indicated
not indicated	not indicated
not indicated	not indicated
not indicated	not indicated
not indicated	not indicated
not indicated	not indicated
not indicated	not indicated
not indicated	not indicated
not indicated	not indicated
not indicated	not indicated
not indicated	not indicated
not indicated	not indicated
not indicated	not indicated
not indicated	not indicated
not indicated	not indicated
not indicated	not indicated
not indicated	not indicated

not indicated	not indicated
not indicated	not indicated
not indicated	not indicated
not indicated	not indicated
not indicated	not indicated
not indicated	not indicated
not indicated	not indicated
not indicated	not indicated
not indicated	not indicated
not indicated	not indicated
not indicated	not indicated
not indicated	not indicated
not indicated	not indicated
not indicated	not indicated
not indicated	not indicated
not indicated	not indicated
not indicated	not indicated
not indicated	not indicated
not indicated	not indicated
not indicated	not indicated
not indicated	not indicated
not indicated	not indicated
not indicated	not indicated
not indicated	not indicated
not indicated	not indicated
fferent types of cultivatio	2018-2019
fferent types of cultivatio	2018-2019
fferent types of cultivatio	2018-2019
fferent types of cultivatio	2018-2019
fferent types of cultivatio	2018-2019
fferent types of cultivatio	2018-2019
fferent types of cultivatio	2018-2019
fferent types of cultivatio	2018-2019
fferent types of cultivatio	2018-2019
fferent types of cultivatio	2018-2019
fferent types of cultivatio	2018-2019
fferent types of cultivatio	2018-2019
fferent types of cultivatio	2018-2019
fferent types of cultivatio	2018-2019
fferent types of cultivatio	2018-2019
fferent types of cultivatio	2018-2019
fferent types of cultivatio	2018-2019
fferent types of cultivatio	2018-2019
fferent types of cultivatio	2018-2019
fferent types of cultivatio	2018-2019
fferent types of cultivatio	2018-2019
fferent types of cultivatio	2018-2019
fferent types of cultivatio	2018-2019
fferent types of cultivatio	2018-2019
fferent types of cultivatio	2018-2019
fferent types of cultivatio	2018-2019
fferent types of cultivatio	2018-2019
fferent types of cultivatio	2018-2019
fferent types of cultivatio	2018-2019
fferent types of cultivatio	2018-2019
not indicated	2018-2019
not indicated	2018-2019
not indicated	2018-2019

not indicated	2018-2019
not indicated	2018-2019
not indicated	2018-2019
not indicated	2018-2019
not indicated	2018-2019
not indicated	2018-2019
not indicated	2018-2019
not indicated	2018-2019
not indicated	2018-2019
fferent types of cultivatio	2018-2019
fferent types of cultivatio	2018-2019
fferent types of cultivatio	2018-2019
fferent types of cultivatio	2018-2019
fferent types of cultivatio	2018-2019
fferent types of cultivatio	2018-2019
fferent types of cultivatio	2018-2019
fferent types of cultivatio	2018-2019
fferent types of cultivatio	2018-2019
fferent types of cultivatio	2018-2019
fferent types of cultivatio	2018-2019
fferent types of cultivatio	2018-2019
fferent types of cultivatio	2018-2019
fferent types of cultivatio	2018-2019
fferent types of cultivatio	2018-2019
not indicated	2018-2019
not indicated	2018-2019
not indicated	2018-2019
not indicated	2018-2019
not indicated	2018-2019
not indicated	2018-2019
not indicated	2018-2019
not indicated	2018-2019
not indicated	2018-2019
not indicated	2018-2019
not indicated	2018-2019
not indicated	2018-2019
fferent types of cultivatio	2018-2019
fferent types of cultivatio	2018-2019
fferent types of cultivatio	2018-2019
fferent types of cultivatio	2018-2019
fferent types of cultivatio	2018-2019
fferent types of cultivatio	2018-2019
fferent types of cultivatio	2018-2019
fferent types of cultivatio	2018-2019
fferent types of cultivatio	2018-2019
fferent types of cultivatio	2018-2019
fferent types of cultivatio	2018-2019
fferent types of cultivatio	2018-2019
fferent types of cultivatio	2018-2019
fferent types of cultivatio	2018-2019
fferent types of cultivatio	2018-2019
fferent types of cultivatio	2018-2019
fferent types of cultivatio	2018-2019
fferent types of cultivatio	2018-2019
fferent types of cultivatio	2018-2019
fferent types of cultivatio	2018-2019
fferent types of cultivatio	2018-2019
fferent types of cultivatio	2018-2019

fferent types of cultivatio	2018-2019
fferent types of cultivatio	2018-2019
fferent types of cultivatio	2018-2019
fferent types of cultivatio	2018-2019
fferent types of cultivatio	2018-2019
fferent types of cultivatio	2018-2019
fferent types of cultivatio	2018-2019
fferent types of cultivatio	2018-2019
not indicated	2018-2019
not indicated	2018-2019
not indicated	2018-2019
not indicated	2018-2019
not indicated	2018-2019
not indicated	2018-2019
not indicated	2018-2019
not indicated	2018-2019
not indicated	2018-2019
not indicated	2018-2019
not indicated	2018-2019
not indicated	2018-2019
fferent types of cultivatio	2018-2019
fferent types of cultivatio	2018-2019
fferent types of cultivatio	2018-2019
fferent types of cultivatio	2018-2019
fferent types of cultivatio	2018-2019
fferent types of cultivatio	2018-2019
fferent types of cultivatio	2018-2019
fferent types of cultivatio	2018-2019
fferent types of cultivatio	2018-2019
fferent types of cultivatio	2018-2019
fferent types of cultivatio	2018-2019
fferent types of cultivatio	2018-2019
fferent types of cultivatio	2018-2019
fferent types of cultivatio	2018-2019
fferent types of cultivatio	2018-2019
not indicated	2018-2019
not indicated	2018-2019
not indicated	2018-2019
not indicated	2018-2019
not indicated	2018-2019
not indicated	2018-2019
not indicated	2018-2019
not indicated	2018-2019
not indicated	2018-2019
not indicated	2018-2019
not indicated	2018-2019
not indicated	2018-2019
fferent types of cultivatio	2018-2019
fferent types of cultivatio	2018-2019
fferent types of cultivatio	2018-2019
fferent types of cultivatio	2018-2019
fferent types of cultivatio	2018-2019
fferent types of cultivatio	2018-2019

fferent types of cultivatio	2018-2019
fferent types of cultivatio	2018-2019
fferent types of cultivatio	2018-2019
fferent types of cultivatio	2018-2019
fferent types of cultivatio	2018-2019
fferent types of cultivatio	2018-2019
fferent types of cultivatio	2018-2019
fferent types of cultivatio	2018-2019
fferent types of cultivatio	2018-2019
not indicated	2018-2019
not indicated	2018-2019
not indicated	2018-2019
not indicated	2018-2019
not indicated	2018-2019
not indicated	2018-2019
not indicated	2018-2019
not indicated	2018-2019
not indicated	2018-2019
not indicated	2018-2019
not indicated	2018-2019
not indicated	2018-2019
fferent types of cultivatio	2018-2019
fferent types of cultivatio	2018-2019
fferent types of cultivatio	2018-2019
fferent types of cultivatio	2018-2019
fferent types of cultivatio	2018-2019
fferent types of cultivatio	2018-2019
fferent types of cultivatio	2018-2019
fferent types of cultivatio	2018-2019
fferent types of cultivatio	2018-2019
fferent types of cultivatio	2018-2019
fferent types of cultivatio	2018-2019
fferent types of cultivatio	2018-2019
fferent types of cultivatio	2018-2019
fferent types of cultivatio	2018-2019
fferent types of cultivatio	2018-2019
not indicated	2018-2019
not indicated	2018-2019
not indicated	2018-2019
not indicated	2018-2019
not indicated	2018-2019
not indicated	2018-2019
not indicated	2018-2019
not indicated	2018-2019
not indicated	2018-2019
not indicated	2018-2019
not indicated	2018-2019
not indicated	2018-2019
fferent types of cultivatio	2018-2019
fferent types of cultivatio	2018-2019
fferent types of cultivatio	2018-2019
fferent types of cultivatio	2018-2019
fferent types of cultivatio	2018-2019

fferent types of cultivatio	2018-2019
fferent types of cultivatio	2018-2019
fferent types of cultivatio	2018-2019
fferent types of cultivatio	2018-2019
fferent types of cultivatio	2018-2019
fferent types of cultivatio	2018-2019
fferent types of cultivatio	2018-2019
fferent types of cultivatio	2018-2019
fferent types of cultivatio	2018-2019
fferent types of cultivatio	2018-2019
not indicated	2018-2019
not indicated	2018-2019
not indicated	2018-2019
not indicated	2018-2019
not indicated	2018-2019
not indicated	2018-2019
not indicated	2018-2019
not indicated	2018-2019
not indicated	2018-2019
not indicated	2018-2019
not indicated	2018-2019
not indicated	2018-2019
fferent types of cultivatio	2018-2019
fferent types of cultivatio	2018-2019
fferent types of cultivatio	2018-2019
fferent types of cultivatio	2018-2019
fferent types of cultivatio	2018-2019
fferent types of cultivatio	2018-2019
fferent types of cultivatio	2018-2019
fferent types of cultivatio	2018-2019
fferent types of cultivatio	2018-2019
fferent types of cultivatio	2018-2019
fferent types of cultivatio	2018-2019
fferent types of cultivatio	2018-2019
fferent types of cultivatio	2018-2019
fferent types of cultivatio	2018-2019
fferent types of cultivatio	2018-2019
not indicated	2018-2019
not indicated	2018-2019
not indicated	2018-2019
not indicated	2018-2019
not indicated	2018-2019
not indicated	2018-2019
not indicated	2018-2019
not indicated	2018-2019
not indicated	2018-2019
not indicated	2018-2019
not indicated	2018-2019
not indicated	2018-2019
fferent types of cultivatio	2018-2019
fferent types of cultivatio	2018-2019

fferent types of cultivatio	2018-2019
fferent types of cultivatio	2018-2019
fferent types of cultivatio	2018-2019
fferent types of cultivatio	2018-2019
fferent types of cultivatio	2018-2019
fferent types of cultivatio	2018-2019
fferent types of cultivatio	2018-2019
fferent types of cultivatio	2018-2019
fferent types of cultivatio	2018-2019
fferent types of cultivatio	2018-2019
fferent types of cultivatio	2018-2019
fferent types of cultivatio	2018-2019
fferent types of cultivatio	2018-2019
not indicated	2018-2019
not indicated	2018-2019
not indicated	2018-2019
not indicated	2018-2019
not indicated	2018-2019
not indicated	2018-2019
not indicated	2018-2019
not indicated	2018-2019
not indicated	2018-2019
not indicated	2018-2019
not indicated	2018-2019
not indicated	2018-2019
fferent types of cultivatio	2018-2019
fferent types of cultivatio	2018-2019
fferent types of cultivatio	2018-2019
fferent types of cultivatio	2018-2019
fferent types of cultivatio	2018-2019
fferent types of cultivatio	2018-2019
fferent types of cultivatio	2018-2019
fferent types of cultivatio	2018-2019
fferent types of cultivatio	2018-2019
fferent types of cultivatio	2018-2019
fferent types of cultivatio	2018-2019
fferent types of cultivatio	2018-2019
fferent types of cultivatio	2018-2019
fferent types of cultivatio	2018-2019
fferent types of cultivatio	2018-2019
not indicated	2018-2019
not indicated	2018-2019
not indicated	2018-2019
not indicated	2018-2019
not indicated	2018-2019
not indicated	2018-2019
not indicated	2018-2019
not indicated	2018-2019
not indicated	2018-2019
not indicated	2018-2019
not indicated	2018-2019

not indicated	2018-2019
not indicated	2011
not indicated	2011
not indicated	2011
not indicated	2011
not indicated	2011
not indicated	2011
not indicated	2011
not indicated	2011
not indicated	2011
not indicated	2011
not indicated	2011
not indicated	2011
not indicated	2011
not indicated	2011
not indicated	2011
not indicated	2011
not indicated	2011
not indicated	2011
not indicated	2011
not indicated	2011
not indicated	2011
not indicated	2011
not indicated	2011
not indicated	2011
not indicated	2011
not indicated	2011
not indicated	2011
not indicated	2011
not indicated	2011
not indicated	2011
not indicated	2009-2010
not indicated	2009-2010
not indicated	2009-2010
not indicated	2009-2010
not indicated	2009-2010
not indicated	2009-2010
not indicated	2002-2003
not indicated	2002-2003
not indicated	2002-2003
not indicated	2002-2003
not indicated	2002-2003
not indicated	2002-2003
not indicated	2002-2003
not indicated	not indicated
not indicated	not indicated
not indicated	not indicated
not indicated	2013
not indicated	2013
not indicated	2013

not indicated	2013
not indicated	2013
not indicated	2013
not indicated	2013
not indicated	2013
not indicated	2013
not indicated	2013
not indicated	2013
not indicated	2013
not indicated	not indicated
not indicated	not indicated
not indicated	not indicated
not indicated	not indicated
not indicated	not indicated
not indicated	not indicated
not indicated	not indicated
not indicated	not indicated
not indicated	not indicated
not indicated	2012
not indicated	2012
not indicated	2012
not indicated	2012
not indicated	2012
not indicated	2012
not indicated	2012
not indicated	2012
conventional	2008-2009
conventional	2008-2009
conventional	2008-2009
conventional	2008-2009
conventional	2008-2009
conventional	2008-2009
conventional	2008-2009
organic	2008-2009
organic	2008-2009
conventional	2008-2009
conventional	2008-2009
organic	2008-2009
organic	2008-2009
conventional	2008-2009
organic	2008-2009
organic	2008-2009
conventional	2008-2009
organic	2008-2009
conventional	2008-2009
organic	2008-2009
organic	2008-2009
conventional	2008-2009
organic	2008-2009
organic	2008-2009
conventional	2008-2009

conventional	2008-2009
conventional	2008-2009
conventional	2008-2009
organic	2008-2009
conventional	2008-2009
organic	2008-2009
organic	2008-2009
organic	2008-2009
organic	2008-2009
organic	2008-2009
conventional	2008-2009
conventional	2008-2009
organic	2008-2009
organic	2008-2009
organic	2008-2009
conventional	2008-2009
conventional	2008-2009
conventional	2008-2009
conventional	2008-2009
conventional	2008-2009
conventional	2008-2009
conventional	2008-2009
organic	2008-2009
organic	2008-2009
conventional	2008-2009
conventional	2008-2009
organic	2008-2009
conventional	2008-2009
organic	2008-2009
organic	2008-2009
conventional	2008-2009
organic	2008-2009
conventional	2008-2009
organic	2008-2009
organic	2008-2009
conventional	2008-2009
organic	2008-2009
conventional	2008-2009
conventional	2008-2009
conventional	2008-2009
organic	2008-2009
conventional	2008-2009
organic	2008-2009
organic	2008-2009
organic	2008-2009
organic	2008-2009
organic	2008-2009
organic	2008-2009
conventional	2008-2009
conventional	2008-2009
organic	2008-2009

organic	2008-2009
organic	2008-2009
organic	2008-2009
conventional	2008-2009
conventional	2008-2009
conventional	2008-2009
conventional	2008-2009
conventional	2008-2009
conventional	2008-2009
conventional	2008-2009
organic	2008-2009
organic	2008-2009
organic	2008-2009
conventional	2008-2009
conventional	2008-2009
organic	2008-2009
organic	2008-2009
conventional	2008-2009
organic	2008-2009
organic	2008-2009
conventional	2008-2009
organic	2008-2009
conventional	2008-2009
organic	2008-2009
organic	2008-2009
conventional	2008-2009
organic	2008-2009
organic	2008-2009
conventional	2008-2009
conventional	2008-2009
conventional	2008-2009
conventional	2008-2009
organic	2008-2009
conventional	2008-2009
organic	2008-2009
organic	2008-2009
organic	2008-2009
organic	2008-2009
organic	2008-2009
organic	2008-2009
organic	2008-2009
conventional	2008-2009
conventional	2008-2009
organic	2008-2009
not indicated	2003-2004
not indicated	2003-2004
not indicated	2003-2004
not indicated	2003-2004
not indicated	2003-2004
not indicated	2003-2004

not indicated	2003-2004
not indicated	2003-2004
not indicated	2003-2004
not indicated	2003-2004
not indicated	2003-2004
not indicated	2003-2004
not indicated	2003-2004
not indicated	2003-2004
not indicated	2003-2004
not indicated	2003-2004
not indicated	2003-2004
not indicated	2003-2004
not indicated	2003-2004
not indicated	2003-2004
not indicated	2003-2004
not indicated	2003-2004
not indicated	2003-2004
not indicated	2003-2004
not indicated	2003-2004
not indicated	2003-2004
not indicated	2003-2004
not indicated	not indicated
not indicated	not indicated
not indicated	not indicated
not indicated	not indicated
not indicated	not indicated
not indicated	not indicated
not indicated	not indicated
not indicated	not indicated
not indicated	not indicated
not indicated	not indicated
not indicated	not indicated
not indicated	not indicated
not indicated	not indicated
not indicated	not indicated
not indicated	not indicated
not indicated	not indicated
not indicated	not indicated
not indicated	not indicated
not indicated	not indicated
not indicated	not indicated
fferent types of cultivatio	2014-2015
fferent types of cultivatio	2014-2015
fferent types of cultivatio	2014-2015
fferent types of cultivatio	2014-2015
fferent types of cultivatio	2014-2015
fferent types of cultivatio	2014-2015
fferent types of cultivatio	2014-2015
fferent types of cultivatio	2014-2015
fferent types of cultivatio	2014-2015

fferent types of cultivatio	2014-2015
fferent types of cultivatio	2014-2015
fferent types of cultivatio	2014-2015
fferent types of cultivatio	2014-2015
fferent types of cultivatio	2014-2015
fferent types of cultivatio	2014-2015
fferent types of cultivatio	2014-2015
fferent types of cultivatio	2014-2015
fferent types of cultivatio	2014-2015
fferent types of cultivatio	2014-2015
fferent types of cultivatio	2014-2015
fferent types of cultivatio	2014-2015
fferent types of cultivatio	2014-2015
fferent types of cultivatio	2014-2015
fferent types of cultivatio	2014-2015
fferent types of cultivatio	2014-2015
fferent types of cultivatio	2014-2015
fferent types of cultivatio	2014-2015
not indicated	2006-2008
not indicated	2006-2008
not indicated	2006-2008
not indicated	2006-2008
not indicated	2006-2008
not indicated	2006-2008
not indicated	2006-2008
not indicated	2006-2008
organic	2011-2012
not indicated	2011-2012
not indicated	2011-2012
not indicated	2014-2016
not indicated	2014-2016
not indicated	2014-2016
not indicated	2014-2016
not indicated	2014-2016
not indicated	2014-2016
not indicated	2014-2016
not indicated	2014-2016
not indicated	2014-2016
not indicated	2014-2016
not indicated	2014-2016
not indicated	2014-2016
not indicated	2014-2016
not indicated	2014-2016
not indicated	2014-2016
not indicated	2014-2016
not indicated	2014-2016
not indicated	2014-2016
not indicated	2014-2016
not indicated	2014-2016
not indicated	2014-2016

not indicated	2014-2016
not indicated	2014-2016
not indicated	2014-2016
not indicated	2014-2016
not indicated	2014-2016
not indicated	2014-2016
not indicated	2014-2016
not indicated	2014-2016
not indicated	2014-2016
not indicated	2014-2016
not indicated	2014-2016
not indicated	2014-2016
not indicated	2014-2016
not indicated	2014-2016
not indicated	2014-2016
not indicated	2014-2016
not indicated	2014-2016
not indicated	2014-2016
not indicated	2014-2016
not indicated	2014-2016
not indicated	2014-2016
not indicated	2014-2016
not indicated	2014-2016
not indicated	2014-2016
not indicated	2014-2016
not indicated	2014-2016
not indicated	2014-2016
not indicated	2014-2016
not indicated	2014-2016
not indicated	2014-2016
not indicated	2014-2016
not indicated	2014-2016
not indicated	2014-2016
not indicated	2014-2016
not indicated	2014-2016
not indicated	2014-2016
not indicated	2014-2016
not indicated	2014-2016
not indicated	2014-2016
not indicated	2014-2016
not indicated	2014-2016
not indicated	2014-2016
not indicated	2013
not indicated	2013
not indicated	2013
not indicated	2013
not indicated	2013
not indicated	2013
not indicated	2013
not indicated	2013
not indicated	2013

not indicated	2013
not indicated	2013
not indicated	2013
not indicated	2013
not indicated	2013
not indicated	2013
not indicated	2013
not indicated	2013
not indicated	2013
not indicated	2013
not indicated	2013
not indicated	2013
not indicated	2013
not indicated	2013
not indicated	2013
not indicated	2013
not indicated	2013
not indicated	2013
not indicated	2013
not indicated	2013
not indicated	2013
not indicated	2013
not indicated	2013
not indicated	2013
not indicated	2013
not indicated	2013
not indicated	2013
not indicated	2013
not indicated	2013
not indicated	2013
not indicated	2013
not indicated	2013
not indicated	2013
not indicated	2013
not indicated	2013
not indicated	2013
not indicated	2013
not indicated	2013
not indicated	2013
not indicated	2013
not indicated	2013
not indicated	2013
not indicated	2013
not indicated	2013
not indicated	2013
not indicated	2013
not indicated	2013
not indicated	2013
not indicated	2013
not indicated	2013
not indicated	2013

not indicated	2013
not indicated	2013
not indicated	2013
not indicated	2013
not indicated	2013
not indicated	2013
not indicated	2013
not indicated	2013
not indicated	2013
not indicated	2013
not indicated	2013
not indicated	2013
not indicated	2013
not indicated	2013
not indicated	2013
not indicated	2013
not indicated	2013
not indicated	2013
not indicated	2013
not indicated	2013
not indicated	2013
not indicated	2013
not indicated	2013
not indicated	2013
not indicated	2013
not indicated	2013
not indicated	2013
not indicated	2013
not indicated	2013
not indicated	2013
not indicated	2001-2002
not indicated	2001-2002
not indicated	2001-2002
not indicated	2001-2002
not indicated	2001-2002
not indicated	2001-2002
not indicated	2001-2002
not indicated	2001-2002
not indicated	2001-2002
not indicated	2001-2002
not indicated	2017-2018
not indicated	2017-2018
not indicated	2017-2018
not indicated	2017-2018
not indicated	2017-2018
not indicated	2017-2018
not indicated	2017-2018
not indicated	2017-2018
not indicated	2017-2018
not indicated	2017-2018

not indicated	2017-2018
not indicated	2017-2018
not indicated	2017-2018
not indicated	2017-2018
not indicated	2017-2018
not indicated	2017-2018
not indicated	2017-2018
not indicated	2017-2018
not indicated	2017-2018
not indicated	2017-2018
not indicated	2017-2018
not indicated	2017-2018
not indicated	2017-2018
not indicated	2017-2018
not indicated	2017-2018
not indicated	2017-2018
not indicated	2017-2018
not indicated	2017-2018
not indicated	2017-2018
not indicated	2017-2018
not indicated	2017-2018
not indicated	2017-2018
not indicated	2017-2018
not indicated	2017-2018
not indicated	2017-2018
not indicated	2017-2018
not indicated	2017-2018
not indicated	2017-2018
not indicated	not indicated
not indicated	2017-2018
not indicated	2017-2018
not indicated	2017-2018
not indicated	2017-2018
not indicated	2017-2018
not indicated	2017-2018
not indicated	2017-2018
not indicated	2017-2018
not indicated	2017-2018
not indicated	2017-2018
not indicated	2017-2018
not indicated	2017-2018
not indicated	2017-2018
not indicated	2017-2018
not indicated	2017-2018
not indicated	2017-2018
not indicated	2017-2018
not indicated	2017-2018
not indicated	2017-2018
not indicated	2017-2018
not indicated	2017-2018

not indicated	2017-2018
not indicated	2017-2018
not indicated	2017-2018
not indicated	2017-2018
not indicated	2017-2018
not indicated	2017-2018
not indicated	2017-2018
not indicated	2017-2018
not indicated	2017-2018
not indicated	2017-2018
not indicated	2017-2018
not indicated	2017-2018
not indicated	2008
not indicated	2008
not indicated	2008
not indicated	2008
not indicated	2008
not indicated	2008
not indicated	2017
not indicated	2017
not indicated	2017
not indicated	2017
not indicated	2011
not indicated	2011
not indicated	2011
not indicated	2011
not indicated	2011
not indicated	2011
not indicated	2011
not indicated	2011
hydroponic	2015
conventional	2015
organic	2015
organic	2015
not indicated	2011-2012
not indicated	2011-2012
not indicated	2011-2012
not indicated	2011-2012
not indicated	2011-2012
not indicated	2011-2012
not indicated	2011-2012
not indicated	2011-2012
not indicated	2011-2012
not indicated	2011-2012
not indicated	2011-2012
not indicated	2011-2012
not indicated	2011-2012
not indicated	2011-2012
not indicated	2011-2012
not indicated	2011-2012

not indicated	2011-2012
not indicated	2011-2012
not indicated	2011-2012
not indicated	2011-2012
not indicated	2011-2012
not indicated	2011-2012
not indicated	2011-2012
not indicated	2011-2012
not indicated	2011-2012
not indicated	2011-2012
not indicated	2011-2012
not indicated	2011-2012
not indicated	2011-2012
not indicated	2011-2012
not indicated	2011-2012
not indicated	2011-2012
not indicated	2011-2012
not indicated	2011-2012
not indicated	2011-2012
not indicated	2011-2012
not indicated	2011-2012
not indicated	2011-2012
not indicated	2011-2012
not indicated	2011-2012
not indicated	2011-2012
not indicated	2011-2012
not indicated	2011-2012
not indicated	2011-2012
not indicated	2011-2012
not indicated	2011-2012
not indicated	2011-2012
not indicated	2011-2012
not indicated	2011-2012
not indicated	2011-2012
not indicated	2011-2012
not indicated	2011-2012
not indicated	2011-2012
not indicated	2011-2012
not indicated	2011-2012
not indicated	2011-2012
not indicated	2011-2012
not indicated	2011-2012
not indicated	2011-2012
not indicated	2011-2012
not indicated	2011-2012
not indicated	2011-2012
not indicated	2011-2012
not indicated	2011-2012
not indicated	2011-2012
not indicated	2011-2012

conventional	not indicated
not indicated	2006
not indicated	2006
not indicated	2006
not indicated	2006
not indicated	2006
not indicated	2006
not indicated	2006
not indicated	2006
not indicated	2006
not indicated	2006
not indicated	2006
not indicated	2006
not indicated	2006
not indicated	2006
not indicated	2006
not indicated	2006
not indicated	2006
not indicated	2006
conventional	2009-2010
conventional	2009-2010
conventional	2009-2010
hydroponic	2009-2010
conventional	2009-2010
hydroponic	2009-2010
conventional	2009-2010
conventional	2009-2010
conventional	2009-2010
conventional	2009-2010
hydroponic	2009-2010
conventional	2009-2010
conventional	2009-2010
hydroponic	2009-2010
conventional	2009-2010
conventional	2009-2010
conventional	2009-2010
conventional	2009-2010
hydroponic	2009-2010
conventional	2009-2010
conventional	2009-2010
conventional	2009-2010
conventional	2009-2010
hydroponic	2009-2010
conventional	2009-2010
conventional	2009-2010
conventional	2009-2010
conventional	2009-2010
hydroponic	2009-2010
conventional	2009-2010
conventional	2009-2010
hydroponic	2009-2010

conventional	2009-2010
conventional	2009-2010
hydroponic	2009-2010
conventional	2009-2010
conventional	2009-2010
conventional	2009-2010
conventional	2009-2010
hydroponic	2009-2010
conventional	2009-2010
conventional	2009-2010
hydroponic	2009-2010
conventional	2009-2010
conventional	2009-2010
conventional	2009-2010
hydroponic	2009-2010
conventional	2009-2010
conventional	2009-2010
conventional	2009-2010
hydroponic	2009-2010
conventional	2009-2010
not indicated	2002-2003
not indicated	2002-2003
not indicated	2002-2003
not indicated	2002-2003
not indicated	2002-2003
not indicated	2002-2003
not indicated	2002-2003
not indicated	2002-2003
not indicated	2002-2003
not indicated	2002-2003
not indicated	2002-2003
not indicated	2002-2003
not indicated	2002-2003
not indicated	2002-2003
not indicated	2002-2003
not indicated	2002-2003
not indicated	2002-2003
not indicated	2002-2003
not indicated	2002-2003
not indicated	2002-2003
not indicated	not indicated
not indicated	not indicated
not indicated	not indicated
not indicated	not indicated
not indicated	not indicated
not indicated	not indicated
not indicated	not indicated
not indicated	2011-2012
not indicated	2011-2012
not indicated	2011-2012

not indicated	2011-2012
not indicated	2011-2012
not indicated	2011-2012
not indicated	2011-2012
not indicated	2011-2012
not indicated	2011-2012
not indicated	2011-2012
not indicated	2011-2012
not indicated	2011-2012
not indicated	2011-2012
not indicated	2017-2018
not indicated	2017-2018
not indicated	2017-2018
not indicated	2017-2018
not indicated	2017-2018
not indicated	2017- 2018
not indicated	2017-2018
not indicated	2017-2018
not indicated	2017-2018
not indicated	2017-2018
not indicated	2017- 2018
not indicated	2017-2018
not indicated	2017-2018
not indicated	2017-2018
not indicated	2017-2018
not indicated	2017-2018
not indicated	2017-2018
not indicated	2017-2018
not indicated	2017-2018
not indicated	2017-2018
not indicated	2017-2018
not indicated	2001
not indicated	2001
not indicated	2009-2011
not indicated	2009-2011
not indicated	2009-2011
not indicated	2009-2011
not indicated	2009-2011
not indicated	2009-2011
not indicated	2009-2011
not indicated	2009-2011
not indicated	2009-2011
not indicated	2009-2011
not indicated	2009-2011
not indicated	2009-2011
not indicated	2009-2011
not indicated	2009-2011
not indicated	2009-2011
not indicated	2009-2011
not indicated	2009-2011

not indicated	2009-2011
not indicated	2009-2011
not indicated	2009-2011
not indicated	2009-2011
not indicated	2009-2011
not indicated	2009-2011
not indicated	2009-2011
not indicated	2009-2011
not indicated	2009-2011
not indicated	2009-2011
not indicated	2009-2011
not indicated	2009-2011
not indicated	2009-2011
not indicated	2009-2011
not indicated	2009-2011
not indicated	2009-2011
not indicated	2009-2011
not indicated	2009-2011
not indicated	2009-2011
not indicated	2009-2011
not indicated	2009-2011
not indicated	2020
not indicated	2020
not indicated	2020
not indicated	2020
not indicated	2020
organic	2014- 2016
organic	2014- 2016
organic	2014- 2016
organic	2014- 2016
organic	2014- 2016
organic	2014- 2016
organic	2014- 2016
organic	2014- 2016
organic	2014- 2016
organic	2014- 2016
organic	2014- 2016
conventional	2019
organic	2019
hydroponic	2019
organic	2014- 2016
not indicated	2016-2017
not indicated	2016-2017
not indicated	2016-2017
not indicated	2016-2017
not indicated	2016-2017
not indicated	2016-2017
not indicated	2016-2017
not indicated	2016-2017
not indicated	2016-2017

not indicated	2016-2017
not indicated	2016-2017
not indicated	2016-2017
not indicated	2016-2017
not indicated	2016-2017
not indicated	2016-2017
not indicated	2016-2017
not indicated	2016-2017
not indicated	2016-2017
not indicated	2016-2017
not indicated	2016-2017
not indicated	2016-2017
not indicated	2016-2017
not indicated	2016-2017
not indicated	2016-2017
not indicated	2016-2017
not indicated	2016-2017
not indicated	2016-2017
not indicated	2016-2017
not indicated	2016-2017
not indicated	2016-2017
not indicated	2016-2017
not indicated	2016-2017
not indicated	2016-2017
not indicated	2016-2017
not indicated	2016-2017
not indicated	2016-2017
not indicated	2016-2017
not indicated	2016-2017
not indicated	2016-2017
not indicated	2016-2017
not indicated	2016-2017
not indicated	2016-2017
not indicated	2016-2017
not indicated	2016-2017
not indicated	2016-2017
not indicated	2016-2017
not indicated	2016-2017
not indicated	2016-2017
not indicated	2016-2017
not indicated	2016-2017
not indicated	not indicated
not indicated	not indicated
not indicated	not indicated
not indicated	not indicated
not indicated	not indicated
not indicated	not indicated
not indicated	not indicated
not indicated	not indicated
not indicated	not indicated
not indicated	not indicated

not indicated	not indicated
not indicated	not indicated
not indicated	not indicated
not indicated	not indicated
not indicated	not indicated
not indicated	not indicated
not indicated	not indicated
not indicated	not indicated
not indicated	not indicated
not indicated	not indicated
not indicated	2017-2018
not indicated	2017-2018
not indicated	2017-2018
not indicated	2017-2018
not indicated	2017-2018
not indicated	2017-2018
not indicated	2017-2018
not indicated	2017-2018
fferent types of cultivatio	2015-1016
fferent types of cultivatio	2015-1016
organic	2015-1016
fferent types of cultivatio	2015-1016
organic	2015-1016
organic	2015-1016
organic	2015-1016
organic	2015-1016
not indicated	1999-2001
not indicated	1999-2001
not indicated	1999-2001
not indicated	1999-2001
not indicated	1999-2001
not indicated	1999-2001
not indicated	1999-2001
not indicated	1999-2001
not indicated	1999-2001
not indicated	1999-2001
not indicated	1999-2001
not indicated	1999-2001
not indicated	1999-2001
not indicated	1999-2001
not indicated	1999-2001
not indicated	1999-2001
not indicated	1999-2001
not indicated	1999-2001
not indicated	1999-2001
not indicated	1999-2001
not indicated	1999-2001
not indicated	1999-2001
not indicated	1999-2001
not indicated	1999-2001
not indicated	1999-2001
not indicated	1999-2001
not indicated	1999-2001
not indicated	1999-2001
not indicated	1999-2001
not indicated	1999-2001

not indicated	1999-2001
not indicated	1999-2001
not indicated	1999-2001
not indicated	1999-2001
not indicated	1999-2001
not indicated	2018
not indicated	2018
not indicated	2018
not indicated	2018
not indicated	2018
not indicated	2018
not indicated	2018
not indicated	2018
not indicated	2018
not indicated	2018
not indicated	2018
not indicated	2018
not indicated	2018
not indicated	2018
not indicated	2018
not indicated	2018
not indicated	2018
not indicated	2018
not indicated	2018
not indicated	2006-2007
not indicated	2006-2007
not indicated	2006-2007
not indicated	2006-2007
not indicated	2006-2007
not indicated	2006-2007
not indicated	not indicated
not indicated	not indicated
not indicated	not indicated
not indicated	not indicated
not indicated	not indicated
not indicated	not indicated
not indicated	not indicated
not indicated	not indicated
not indicated	not indicated
not indicated	not indicated
not indicated	not indicated
not indicated	not indicated
not indicated	not indicated
not indicated	not indicated
not indicated	not indicated
not indicated	not indicated
not indicated	not indicated
not indicated	not indicated
not indicated	not indicated
not indicated	not indicated
not indicated	not indicated
not indicated	not indicated
not indicated	not indicated
not indicated	not indicated

not indicated	not indicated
not indicated	not indicated
not indicated	not indicated
not indicated	not indicated
not indicated	not indicated
not indicated	not indicated
not indicated	not indicated
not indicated	not indicated
not indicated	not indicated
not indicated	not indicated
not indicated	not indicated
not indicated	not indicated
not indicated	not indicated
not indicated	not indicated
not indicated	not indicated
not indicated	not indicated
not indicated	not indicated
not indicated	not indicated
not indicated	not indicated
not indicated	not indicated
not indicated	not indicated
not indicated	not indicated
not indicated	not indicated
not indicated	not indicated
not indicated	not indicated
not indicated	not indicated
not indicated	not indicated
not indicated	not indicated
not indicated	not indicated
not indicated	not indicated
not indicated	not indicated
not indicated	not indicated
not indicated	not indicated
not indicated	not indicated
not indicated	not indicated
not indicated	not indicated
not indicated	not indicated
not indicated	not indicated
not indicated	not indicated
not indicated	not indicated
not indicated	not indicated
not indicated	not indicated
not indicated	not indicated
not indicated	not indicated
not indicated	not indicated
not indicated	not indicated
not indicated	not indicated
not indicated	not indicated
not indicated	not indicated
not indicated	not indicated
not indicated	not indicated

not indicated	not indicated
not indicated	not indicated
not indicated	not indicated
not indicated	not indicated
not indicated	not indicated
not indicated	not indicated
not indicated	not indicated
not indicated	2012
not indicated	2012
not indicated	2012
not indicated	2012
not indicated	2012
not indicated	2012
not indicated	2012
not indicated	2012
not indicated	2012
not indicated	2012
not indicated	2012
not indicated	2012
not indicated	2012
not indicated	2012
not indicated	2012
not indicated	2012
not indicated	2012
not indicated	2012
not indicated	2012
not indicated	2012
not indicated	2012
not indicated	2012
not indicated	2012
not indicated	2012
not indicated	2012
not indicated	2011
not indicated	2011
not indicated	2011
not indicated	2011
not indicated	2011
not indicated	2011
not indicated	2011
not indicated	2011
not indicated	2011
not indicated	2011
not indicated	2011
not indicated	2011
not indicated	2011
not indicated	2011
not indicated	2011
not indicated	2011
not indicated	2011
not indicated	2011
not indicated	2011
not indicated	2011
not indicated	2011

not indicated	2011
not indicated	2011
not indicated	2011
not indicated	2011
not indicated	2011
not indicated	2011
not indicated	2011
not indicated	2011
not indicated	2011
not indicated	2011
not indicated	2011
not indicated	2011
not indicated	2011
not indicated	2011
not indicated	2011
not indicated	2011
not indicated	2011
not indicated	2011
not indicated	2011
not indicated	2011
not indicated	2011
not indicated	2011
not indicated	2011
not indicated	2011
not indicated	2011
not indicated	2011
not indicated	2011
not indicated	2011
not indicated	2011
not indicated	2011
not indicated	2011
not indicated	2011
not indicated	2011
not indicated	2011
not indicated	2011
not indicated	2011
not indicated	2011
not indicated	2011
not indicated	2011
not indicated	2011
not indicated	2011
not indicated	2011
not indicated	2011
not indicated	2011
not indicated	2011
not indicated	2011
not indicated	2011
not indicated	2011
not indicated	2011
not indicated	2011
not indicated	2011

not indicated	2011
not indicated	2011
not indicated	2011
not indicated	2011
not indicated	2011
not indicated	2011
not indicated	2011
not indicated	2011
not indicated	2011
not indicated	2011
not indicated	2011
not indicated	2011
not indicated	2011
not indicated	2011
not indicated	2011
not indicated	2011
not indicated	2011
not indicated	2011
not indicated	2011
not indicated	2011
not indicated	2011
not indicated	2011
not indicated	2011
not indicated	2011
not indicated	2011
not indicated	2011
not indicated	2011
not indicated	2011
not indicated	2011
not indicated	2011
not indicated	2011
not indicated	2011
not indicated	2011
not indicated	2011
not indicated	2011
not indicated	2011
not indicated	2011
not indicated	2011
not indicated	2011
not indicated	2011
not indicated	2011
not indicated	2011
not indicated	2011
not indicated	2011
not indicated	2011
not indicated	2011
not indicated	2011
not indicated	2011
not indicated	2008
not indicated	2008
not indicated	2008
not indicated	2008

not indicated	2008
not indicated	2008
not indicated	2008
not indicated	2008
not indicated	2008
not indicated	2008
not indicated	2008
not indicated	2008
not indicated	2008
not indicated	2008
not indicated	2008
not indicated	2008
not indicated	2008
not indicated	2008
not indicated	2008
not indicated	2008
not indicated	2008
not indicated	2008
not indicated	2008
not indicated	2008
not indicated	2008
not indicated	2008
not indicated	2008
not indicated	2008
not indicated	2008
not indicated	2008
not indicated	2008
not indicated	2008
not indicated	2008
not indicated	2008
not indicated	2008
not indicated	2008
not indicated	2008
not indicated	2008
not indicated	2008
not indicated	2008
not indicated	2008
not indicated	2008
not indicated	2008
not indicated	2008
not indicated	2008
not indicated	2008
not indicated	2008
not indicated	2008
not indicated	2008
not indicated	2008
not indicated	2008
not indicated	2008
not indicated	2008
not indicated	2008
not indicated	2008

not indicated	2008
not indicated	2008
not indicated	2008
not indicated	2008
not indicated	2008
not indicated	2008
not indicated	2008
not indicated	2008
not indicated	2008
not indicated	2008
not indicated	2008
not indicated	2008
not indicated	2008
not indicated	2008
not indicated	2008
not indicated	2008
not indicated	2008
not indicated	2008
not indicated	2008
not indicated	2008
not indicated	2008
not indicated	2008
not indicated	2008
not indicated	2008
not indicated	2008
not indicated	2008
not indicated	2008
not indicated	2008
not indicated	2008
not indicated	2008
not indicated	2008
not indicated	2008
not indicated	2008
not indicated	2008
not indicated	2008
not indicated	2011
not indicated	2011
not indicated	2011
not indicated	2011
not indicated	2011
not indicated	2011
not indicated	2011
not indicated	2011
not indicated	2011
not indicated	2011
not indicated	2011
not indicated	2011
not indicated	2014-2015
not indicated	2014-2015
not indicated	2014-2015
not indicated	2014-2015
not indicated	2014-2015
not indicated	2014-2015
not indicated	2014-2015
not indicated	2014-2015

not indicated	2014-2015
not indicated	2014-2015
not indicated	1997-1998
not indicated	1997-1998
not indicated	1997-1998
not indicated	1997-1998
not indicated	1997-1998
not indicated	1997-1998
not indicated	1997-1998
not indicated	1997-1998
not indicated	1997-1998
not indicated	1997-1998
not indicated	1997-1998
not indicated	1997-1998
not indicated	1997-1998
not indicated	1997-1998
not indicated	1997-1998
not indicated	1997-1998
not indicated	1997-1998
not indicated	1997-1998
not indicated	1997-1998
not indicated	1997-1998
not indicated	1997-1998
not indicated	1997-1998
not indicated	1997-1998
not indicated	1997-1998
not indicated	1997-1998
not indicated	1997-1998
not indicated	1997-1998
not indicated	1997-1998
not indicated	1997-1998
not indicated	1997-1998
not indicated	1997-1998
not indicated	1997-1998
not indicated	2012
not indicated	2012
not indicated	2012
not indicated	2012
not indicated	2012
not indicated	2012
not indicated	2012
not indicated	2012
not indicated	2012
not indicated	2012
not indicated	2012
not indicated	2012
not indicated	2012
not indicated	2012
not indicated	2012
not indicated	2012
not indicated	2012

not indicated	2012
not indicated	2012
not indicated	2012
not indicated	2012
not indicated	2012
not indicated	2012
not indicated	2012
not indicated	2012
not indicated	2012
not indicated	2012
not indicated	2012
not indicated	2012
not indicated	2012
not indicated	2012
not indicated	2012
not indicated	2012
not indicated	2012
not indicated	2012
not indicated	2012
not indicated	2012
not indicated	2012
not indicated	2012
not indicated	2012
not indicated	2012
not indicated	2012
not indicated	2012
not indicated	2012
not indicated	2012
not indicated	2012
not indicated	2012
not indicated	2012
not indicated	2012
not indicated	2012
not indicated	2012
not indicated	2012
not indicated	2012
not indicated	2012
not indicated	2012
not indicated	2012
not indicated	2012
not indicated	2012
not indicated	2012
not indicated	2012
not indicated	2012
not indicated	2012
not indicated	2012
not indicated	2012
not indicated	2012
not indicated	2012
not indicated	2012
not indicated	2012
not indicated	2012
not indicated	2012
not indicated	2012

not indicated	2012
not indicated	2012
not indicated	2012
not indicated	2012
not indicated	2012
not indicated	2012
not indicated	2012
not indicated	2012
not indicated	2012
not indicated	2012
not indicated	2012
not indicated	2012
not indicated	2012
not indicated	2012
not indicated	2012
not indicated	2012
not indicated	2012
not indicated	2012
not indicated	2012
not indicated	2012
not indicated	2012
not indicated	2012
not indicated	2012
not indicated	2012
not indicated	2012
not indicated	2012
not indicated	2012
not indicated	2012
not indicated	2012
not indicated	2012
not indicated	2012
not indicated	2012
not indicated	2012
not indicated	2012
not indicated	2012
not indicated	2012
not indicated	2012
not indicated	2012
not indicated	2012
not indicated	2003-2004
not indicated	2003-2004
not indicated	2003-2004
not indicated	2003-2004
not indicated	2003-2004
not indicated	2003-2004
not indicated	2003-2004
not indicated	2003-2004
not indicated	2003-2004
not indicated	2003-2004
not indicated	2003-2004

not indicated	2003-2004
not indicated	2003-2004
not indicated	2003-2004
not indicated	2003-2004
not indicated	2003-2004
not indicated	2003-2004
not indicated	2003-2004
not indicated	2003-2004
not indicated	2003-2004
not indicated	2003-2004
not indicated	2003-2004
not indicated	2003-2004
not indicated	2003-2004
not indicated	2003-2004
not indicated	2003-2004
not indicated	2003-2004
not indicated	2003-2004
not indicated	2003-2004
not indicated	2003-2004
not indicated	2003-2004
not indicated	2003-2004
not indicated	2003-2004
not indicated	2003-2004
not indicated	2003-2004
not indicated	2003-2004
not indicated	ISA
not indicated	ISA
not indicated	ISA
not indicated	ISA
not indicated	ISA
not indicated	ISA
not indicated	2016
not indicated	2016
not indicated	2016
not indicated	2016
not indicated	2016
not indicated	2016
not indicated	2016
not indicated	2016
not indicated	2016
not indicated	2016
not indicated	2016
not indicated	2016
not indicated	2016
not indicated	2016
not indicated	2016
not indicated	2015-2016
not indicated	2015-2016
not indicated	2015-2016
not indicated	2015-2016
not indicated	2015-2016
not indicated	2015-2016

organic	2008-2009
conventional	2008-2009
organic	2008-2009
conventional	2008-2009
organic	2008-2009
organic	2008-2009
organic	2008-2009
conventional	2008-2009
conventional	2008-2009
organic	2008-2009
organic	2008-2009
conventional	2008-2009
organic	2008-2009
conventional	2008-2009
conventional	2008-2009
conventional	2008-2009
organic	2008-2009
organic	2008-2009
organic	2008-2009
conventional	2008-2009
conventional	2008-2009
organic	2008-2009
organic	2008-2009
organic	2008-2009
organic	2008-2009
conventional	2008-2009
organic	2008-2009
organic	2008-2009
organic	2008-2009
conventional	2008-2009
conventional	2008-2009
organic	2008-2009
organic	2008-2009
organic	2008-2009
organic	2008-2009
conventional	2008-2009
conventional	2008-2009
organic	2008-2009
conventional	2008-2009
conventional	2008-2009
organic	2008-2009
not indicated	2009
hydroponic	2009
not indicated	not indicated
not indicated	not indicated
not indicated	not indicated
not indicated	not indicated
not indicated	not indicated
not indicated	not indicated
not indicated	not indicated

not indicated	not indicated
not indicated	not indicated
not indicated	2013-2014
not indicated	2013-2014
not indicated	2013-2014
not indicated	2013-2014
not indicated	2013-2014
not indicated	2013-2014
not indicated	2013-2014
not indicated	2004-2005
not indicated	2004-2005
not indicated	2004-2005
not indicated	2004-2005
not indicated	2004-2005
not indicated	2004-2005
not indicated	2004-2005
not indicated	2004-2005
not indicated	2004-2005
not indicated	2004-2005
not indicated	2014-2016
not indicated	2014-2016
not indicated	2012-2016
not indicated	2012-2016
not indicated	2012-2016
not indicated	2012-2016
not indicated	2012-2016
not indicated	2012-2016
not indicated	2012-2016
not indicated	2012-2016
not indicated	2012-2016
not indicated	2012-2016
not indicated	2012-2016
not indicated	2012-2016
not indicated	2012-2016
not indicated	2012-2016
not indicated	2012-2016
not indicated	2012-2016
not indicated	2017
not indicated	2017
not indicated	2017
not indicated	2017
not indicated	2017
not indicated	2017
not indicated	2017
not indicated	2017
not indicated	2017
not indicated	2017
not indicated	2017
not indicated	2017
not indicated	2017
not indicated	2017
not indicated	2017

not indicated	2017
not indicated	2017
not indicated	2017
not indicated	2017
not indicated	2017
not indicated	2017
not indicated	2017
not indicated	2017
not indicated	2017
not indicated	2017
not indicated	2017
not indicated	2017
not indicated	2017
not indicated	2017
not indicated	2017
not indicated	2017
not indicated	2017
not indicated	2017
not indicated	2017
not indicated	2017
not indicated	2017
not indicated	2017
not indicated	2017
not indicated	2017
not indicated	2017
not indicated	2017
not indicated	2017
not indicated	2017
not indicated	2017
not indicated	2017
not indicated	2017
not indicated	2017
not indicated	2017
not indicated	2017
not indicated	2017
not indicated	2017
not indicated	2017
not indicated	2017
not indicated	2017
not indicated	2017
not indicated	2017
not indicated	2017
not indicated	2017
not indicated	2017
not indicated	2017
not indicated	2017
not indicated	2017
not indicated	2017
not indicated	2017
not indicated	2017

not indicated	2017
not indicated	2017
not indicated	2017
not indicated	2017
not indicated	2017
not indicated	2016
not indicated	2016
not indicated	2016
not indicated	2016
not indicated	2016
not indicated	2016
not indicated	2016
not indicated	2016
not indicated	2016
not indicated	2016
not indicated	2016
not indicated	2016
not indicated	2016
not indicated	2016
not indicated	2016
not indicated	2016
not indicated	2016
not indicated	2016
not indicated	2016
not indicated	2016
not indicated	2016
not indicated	2016
not indicated	2016
not indicated	2016
not indicated	2016
not indicated	2016
not indicated	2016
not indicated	2016
not indicated	2016
not indicated	2016
not indicated	2016
not indicated	2016
not indicated	2016
not indicated	2016
not indicated	2016
not indicated	2016
not indicated	2016
not indicated	2016
not indicated	2016
not indicated	2016
not indicated	2016
not indicated	2016
not indicated	2016
not indicated	2016
not indicated	2016

not indicated	2016
not indicated	2016
not indicated	2016
not indicated	2016
not indicated	2016
not indicated	2016
not indicated	2016
not indicated	2015-2017
not indicated	2015 - 2017
not indicated	2015-2017
not indicated	2015 - 2017
not indicated	2015 - 2017
not indicated	2015 - 2017
not indicated	2015 - 2017
not indicated	2015 - 2017
not indicated	2015 - 2017
not indicated	2015-2017
not indicated	2015 - 2017
not indicated	2015 - 2017
not indicated	2015-2017
not indicated	2015-2017
not indicated	2015 - 2017
not indicated	2015 - 2017
not indicated	2015 - 2017
not indicated	2015 - 2017
not indicated	2015 - 2017
not indicated	2014
not indicated	2014
not indicated	2014
not indicated	2014
not indicated	2014
not indicated	2014
not indicated	2014
not indicated	2014
not indicated	2014
not indicated	2014
not indicated	2014
not indicated	2014
not indicated	2014
not indicated	2014
not indicated	2014
not indicated	2014
not indicated	2014
not indicated	2014
not indicated	2014
not indicated	2014
not indicated	2014
not indicated	2014
not indicated	2014
not indicated	2014

not indicated	2014
not indicated	2014
not indicated	2014
not indicated	2014
not indicated	2014
not indicated	2014
not indicated	2014
not indicated	2014
not indicated	2014
not indicated	2014
not indicated	2014
not indicated	2014
not indicated	2014
not indicated	2014
not indicated	2014
not indicated	2014
not indicated	2014
not indicated	2014
not indicated	2014
not indicated	2014
not indicated	2014
not indicated	2014
not indicated	2014
not indicated	2014
not indicated	2014
not indicated	2014
not indicated	2013
not indicated	2013
not indicated	2013
not indicated	2013
not indicated	2013
not indicated	2013
not indicated	2013
not indicated	2013
not indicated	2013
not indicated	2013
