# Supplementary figures and images for: Parasitic contamination in vegetables for human consumption: a systematic review and meta-analysis
Source: Rev Bras Parasitol Vet. 2024 Sep 6;33(3):e002824. doi: 10.1590/S1984-29612024040 (PMC11452068; doi:10.1590/S1984-29612024040)

Figure S1: Fixed and Random Effects Models applied to systematic review studies.

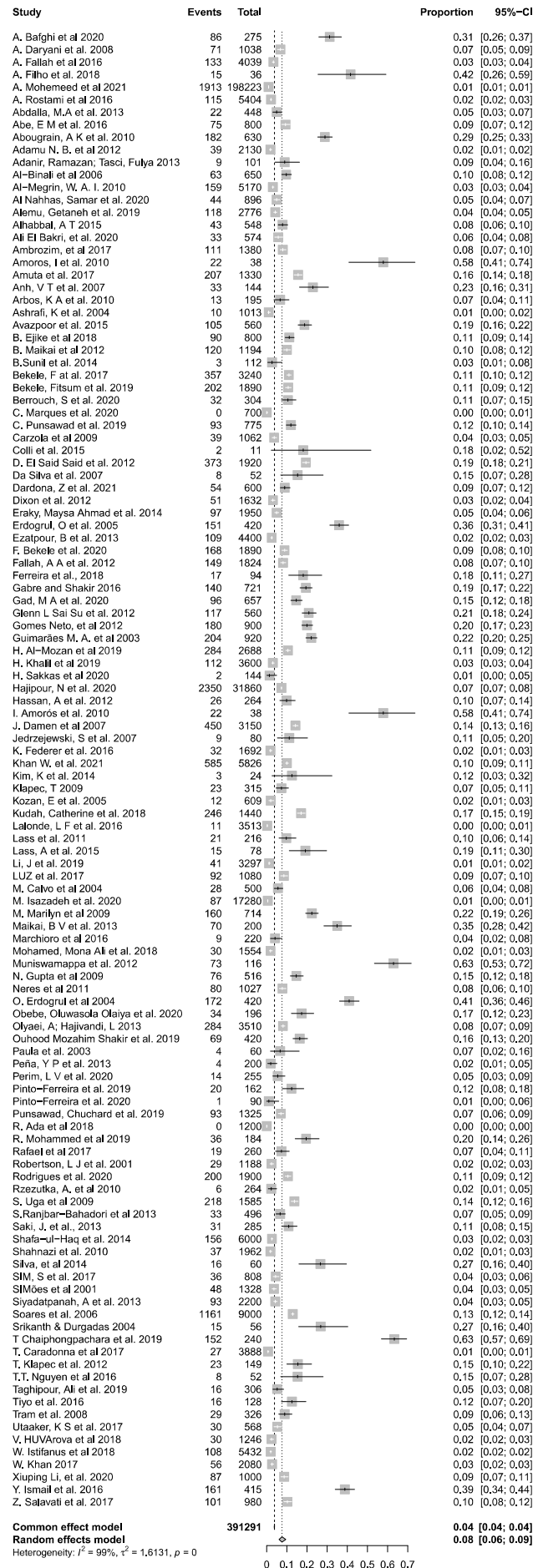

Supplement: Figure S1 [file rbpv-33-3-e002824-Suppl-FigS01.pdf]
