# Supplementary material for: Parasitic contamination in vegetables for human consumption: a systematic review and meta-analysis
Source: Rev Bras Parasitol Vet. 2024 Sep 6;33(3):e002824. doi: 10.1590/S1984-29612024040 (PMC11452068; doi:10.1590/S1984-29612024040)
Supplement: References S1 [file rbpv-33-3-e002824-Suppl-Ref.pdf]

References used in the systematic review and meta-analysis

A Gad MA, Ashour AA, Soliman MI, Saleh ER, Al-Herrawy AZ, Nigm AH. Prevalence of enteric protozoa on fresh vegetables and their irrigation water in Giza, *Egypt. Egypt J Aquat Biol Fish* 2020; 24(2): 451-468. https://doi.org/10.21608/ejabf.2020.85738

Abdalla MA, Salim AM, Hassanin SH. The prevalence of intestinal parasitic diseases in human and its association with the presence of infection stages in vegetables, in El Khorma Province, Saudi Arabia. *Life Sci J* 2013; 10(4): 3107-3113. https://doi.org/10.21315/tlsr2017.28.1.2

Abe EM, Ajah L, Ayuba SO, Mogaji H, Ekpo UF. Geohelminths contamination of fruits and vegetables sold in Lafia markets. *Ann Res Rev Biol* 2016; 11(2): 1-8. https://doi.org/10.9734/ARRB/2016/25566

Ada RT, Obadiah HI, Akuma N, Odaudu E, Omudu EA. Detection of parasite ova and egg in commonly consumed vegetables in Makurdi, Nigeria. *Niger J Parasitol* 2018; 39(1): 79-83. https://doi.org/10.4314/njpar.v39i1.14

Adamu NB, Adamu JY, Mohammed D. Prevalence of helminth parasites found on vegetables sold in Maiduguri, Northeastern Nigeria. *Food Cont* 2012; 25(1): 23-26. https://doi.org/10.1016/j.foodcont.2011.10.016

Adanir R, Tasci F. Prevalence of helminth eggs in raw vegetables consumed in Burdur, Turkey. *Food Cont* 2013; 31(2): 482-484. https://doi.org/10.1016/j.foodcont.2012.10.032

Al Nahhas S, Aboualchamat G. Investigation of parasitic contamination of salad vegetables sold by street vendors in city markets in Damascus, Syria. *Food Waterborne Parasitol* 2020; 21: e00090. https://doi.org/10.1016/j.fawpar.2020.e00090

Al-Binali AM, Bello CS, El-Shewy K, Abdulla SE. The prevalence of parasites in commonly used leafy vegetables in South Western, Saudi Arabia. *Saudi Med J* 2006; 27(5): 613-616. https://pubmed.ncbi.nlm.nih.gov/16680247/

Alemu G, Mama M, Misker D, Haftu D. Parasitic contamination of vegetables marketed in Arba Minch town, southern Ethiopia. *BMC Infect Dis* 2019; 19: 1-7. https://doi.org/10.1186/s12879-019-4020-5

Alhabbal AT. The prevalence of parasitic contamination on common cold vegetables in Alqalamoun Region. *Int J Pharm Sci Rev Res* 2015; 30(1): 94-97. https://api.semanticscholar.org/CorpusID:19832417

Al-Mozan HDK, Dakhil KM. Prevalence of parasites in fresh vegetables from two regions of Thi-Qar Province, Iraq. *J Pure Appl Microbiol* 2019; 13(2): 1103-1110. https://dx.doi.org/10.22207/JPAM.13.2.49

Amorós I, Alonso JL, Cuesta G. Cryptosporidium oocysts and Giardia cysts on salad products irrigated with contaminated water. *J Food Prot* 2010; 73(6): 1138-1140. https://doi.org/10.4315/0362-028x-73_6_1138

Amuta E, Obisike V, Acham N. Prevalence of helminth eggs on raw vegetables and fruits sold in selected markets in Makurdi, Benue State, Nigeria. *Ann Res Rev Biol* 2017; 19(4): 1-6. https://doi.org/ 10.9734/ARRB/2017/35806

Anh VT, Tram NT, Klank LT, Cam PD, Dalsgaard A. Faecal and protozoan parasite contamination of water spinach (Ipomoea aquatica) cultivated in urban wastewater in Phnom Penh, Cambodia. *Trop Med Int Health* 2007; 12: 73-81. https://doi.org/ 10.1111/j.1365-3156.2007.01944.x

Arbos KA, Freitas RJS, Stertz SC, Carvalho LA. Segurança alimentar de hortaliças orgânicas: aspectos sanitários e nutricionais. *Food Sci Technol* 2010; 30: 215-220. https://doi.org/10.1590/S0101-20612010000500033

Avazpoor M, Yousefipoor MT, Dusty M, Mehdipour M, Seifipour F, Gholami Z. Determination of the level of parasitic infection (Cryptosporidium and Giardia) of the vegetables marketed in Ilam city. *Environ Health Eng Manage J* 2015; 2(1): 37-40. https://ssrn.com/abstract=2610016

Aydenizoz M, Gokpinar S, Gazyagci AN. Investigation of parasitological contamination in leafy vegetables in Kırıkkale of Turkey. *Anim Health Prod Hyg* 2017; 6(1): 463-467. https://www.researchgate.net/publication/322518952_Investigation_of_parasitological_contamination_in_leafy_vegetables_in_Kirikkale_of_Turkey

Bafghi AF, Mirzaei F, Yavari MR, Siyadatpanah A, Mitsuwan W, Nissapatorn V, Hosseini SA. Prevalence and risk factors associated with Cryptosporidium infection in raw vegetables in Yazd District, Iran. *World's Vet J* 2020; 10(3): 260-266. https://dx.doi.org/10.36380/scil.2020.wvj34

Bekele F, Shumbej T. Fruit and vegetable contamination with medically important helminths and protozoans in Tarcha town, Dawuro zone, South West Ethiopia. *Res Rep Trop Med* 2019; 19-23. https://doi.org/10.2147/RRTM.S205250

Bekele F, Shumbej T, Dendir A, Mesfin D, Solomon A, Jemal A, Alemayheu M. Contamination rate of commonly consumed fresh vegetables and fruits with parasites of medically importance in Wolkite and Butajira Towns of Gurage Zone, Southern Ethiopia. *Int J Public Health* 2020; 9(3): 211-215. https://doi.org/10.11591/ijphs.v9i3.20395

Bekele F, Tefera T, Biresaw G, Yohannes T. Parasitic contamination of raw vegetables and fruits collected from selected local markets in Arba Minch town, Southern Ethiopia. *Infect Dis Poverty* 2017; 6: 1-7. https://doi.org/ 10.1186/s40249-016-0226-6

Berrouch S, Escotte-Binet S, Amraouza Y, Flori P, Aubert D, Villena I, Hafid J. Cryptosporidium spp., Giardia duodenalis and Toxoplasma gondii detection in fresh vegetables consumed in Marrakech, Morocco. *Afr Health Sci* 2020; 20(4): 1669-1678. https://doi.org/10.1016/j.jfp.2023.100112

Calvo M, Carazo M, Arias ML, Chaves C, Monge R, Chinchilla M. Prevalence of Cyclospora sp., Cryptosporidium sp., microsporidia and fecal coliform determination in fresh fruit and vegetables consumed in Costa Rica. *Arch Latinoam Nutr* 2004; 54(4):

428-432. http://ve.scielo.org/scielo.php?script=sci_arttext&pid=S0004-06222004000400009&lng=es&nrm=iso

Caradonna T, Marangi M, Del Chierico F, Ferrari N, Reddel S, Bracaglia G, Giangaspero A. Detection and prevalence of protozoan parasites in ready-to-eat packaged salads on sale in Italy. *Food Microbiol* 2017; 67: 67-75. https://doi.org/10.1016/j.fm.2017.06.006

Cazorla D, Morales P, Chirinos M, Acosta ME. Parasitological evaluation of vegetables commercially traded in Coro, Falcón state, Venezuela. *Bol Mal Salud Amb* 2009; 49(1): 117-125. https://ve.scielo.org/scielo.php?script=sci_arttext&pid=S1690-46482009000100008&lng=es&nrm=iso

Chaiphongpachara T. Detection of Anisakis spp. and residual formaldehyde in Indian mackerel and splendid squid from a fish market in Samut Songkhram Province, Thailand. *Tropical Biomed* 2019; 36(1): 53-59. https://api.semanticscholar.org/CorpusID:231954479

Colli CM, Bezagio RC, Nishi L, Bignotto TS, Ferreira EC, Falavigna-Guilherme AL, Gomes ML. Identical assemblage of Giardia duodenalis in humans, animals and vegetables in an urban area in southern Brazil indicates a relationship among them. *PLoS One* 2015; 10(3): e0118065. https://doi.org/10.1371/journal.pone.0118065

da Silva Terto WD, de Oliveira RG, de Lima MM. Avaliação parasitológica em alfaces (Lactuca sativa L.) comercializadas em Serra Talhada, Pernambuco, Brasil. *Vigt Sanit Debate* 2014; 2(3): 51-57. https://doi.org/ 10.3395/vd.v2i3.220

Damen JG, Banwat EB, Egah DZ, Allanana JA. Parasitic contamination of vegetables in Jos, Nigeria. *Ann Afr Med* 2007; 6(3): 115-118. https://doi.org/ 10.1371/journal.pone.0118065

Dardona Z, Al Hindi A, Hafidi M, Boumezzough A, Boussaa S. Occurrence of Toxoplasma gondii on raw leafy vegetables in Gaza, Palestine. *J Food Prot* 2021; 84(2): 255-261. https://doi.org/ 10.4315/JFP-20-160

Daryani A, Ettehad GH, Sharif M, Ghorbani L, Ziaei H. Prevalence of intestinal parasites in vegetables consumed in Ardabil, Iran. *Food Cont* 2008; 19(8): 790-794. https://doi.org/10.1016/j.foodcont.2007.08.004

Dixon B, Parrington L, Cook A, Pollari F, Farber J. Detection of Cyclospora, Cryptosporidium, and Giardia in ready-to-eat packaged leafy greens in Ontario, Canada. *J Food Prot* 2013; 76(2): 307-313. https://doi.org/10.4315/0362-028X.JFP-12-282

Ejike BU, Ejike EN, Ogbonna CR, Onu EO. Geohelminth contamination of vegetables sold in some markets in Aba North south-east, Nigeria. *Niger J Parasitol* 2018; 39(2): 194-198. https://doi.org/ 10.4314/njpar.v39i2.14

El Bakri A, Hussein NM, Ibrahim ZA, Hasan H, AbuOdeh R. Intestinal parasite detection in assorted vegetables in the United Arab Emirates. *Oman Med J* 2020; 35(3): e128. https://doi.org/ 10.5001/omj.2020.46

Eraky MA, Rashed SM, Nasr MES, El-Hamshary AMS, Salah El-Ghannam A. Parasitic contamination of commonly consumed fresh leafy vegetables in Benha, *Egypt. J Parasitol Res* 2014. https://doi.org/ 10.1155/2014/613960

Erdogˇrul Ö, Şener H. The contamination of various fruit and vegetable with Enterobius vermicularis, Ascaris eggs, Entamoeba histolyca cysts and Giardia cysts. *Food Cont* 2005; 16(6): 557-560. https://doi.org/10.1016/j.foodcont.2004.06.016

Ezatpour B, Chegeni AS, Abdollahpour F, Aazami M, Alirezaei M. Prevalence of parasitic contamination of raw vegetables in Khorramabad, Iran. Food Control 2013; 34(1): 92-95. https://www.researchgate.net/publication/236875243_Prevalence_of_parasitic_contamination_of_raw_vegetables_in_Khorramabad_Iran

Fallah AA, Pirali-Kheirabadi K, Shirvani F, Saei-Dehkordi SS. Prevalence of parasitic contamination in vegetables used for raw consumption in Shahrekord, Iran: influence of season and washing procedure. *Food Cont* 2012; 25(2): 617-620. https://doi.org/ 10.1016/j.foodcont.2011.12.004

Federer K, Armua-Fernandez MT, Gori F, Hoby S, Wenker C, Deplazes P. Detection of taeniid (Taenia spp., Echinococcus spp.) eggs contaminating vegetables and fruits sold in European markets and the risk for metacestode infections in captive primates. *Int J Parasitol Parasites Wildl* 2016; 5(3): 249-253. https://doi.org/10.1016/j.ijppaw.2016.07.002

Ferreira FP, Caldart ET, Freire RL, Mitsuka-Breganó R, Freitas FMD, Miura AC, Navarro IT. The effect of water source and soil supplementation on parasite contamination in organic vegetable gardens. *Rev Bras Parasitol Vet* 2018; 27: 327-337. https://doi.org/10.1590/S1984-296120180050

Ferreira FP, Freire RL, Caldart ET, Paschoal ATP, Arias GB, Ladeia WA, Navarro IT. Organic horticulture: a current demand, whose proper management is the only guarantee of safe food. *Biotemas* 2019; 32(3): 43-50. https://doi.org/10.5007/2175-7925.2019v32n3p43

Filho AFM, de Azevedo CAV, de Queiroz Almeida Azevedo MR, Fernandes JD, Correa EB, dos Santos SA. Microbiological and parasitological contamination of hydroponic grown curly lettuce under different optimized nutrient solutions. *Aust J Crop Sci* 2018; 12(3): 400-406. https://doi.org/ 10.21475/ajcs.18.12.03.pne797

Gabre RM, Shakir A. Prevalence of some human enteroparasites in commonly consumed raw vegetables in Tabuk, Saudi Arabia. *J Food Prot* 2016; 79(4): 655-658. https://doi.org/ 10.4315/0362-028X.JFP-15-485

Guimarães AM, Alves EGL, Figueiredo HCP, Costa GMD, Rodrigues LDS. Freqüência de enteroparasitas em amostras de alface (Lactuca sativa) comercializadas em Lavras, Minas Gerais. *Rev Soc Bras Med Trop* 2003; 36: 621-623. https://doi.org/10.1590/S0037-86822003000500014

Gupta N, Khan DK, Santra SC. Prevalence of intestinal helminth eggs on vegetables grown in wastewater-irrigated areas of Titagarh, West Bengal, India. *Food Cont* 2009; 20(10): 942-945. https://doi.org/10.1016/j.foodcont.2009.02.003

Hajipour N, Soltani M, Ketzis J, Hassanzadeh P. Zoonotic parasitic organisms on vegetables: impact of production system characteristics on presence, prevalence on vegetables in northwestern Iran and washing methods for removal. *Food Microbiol* 2021; 95: 103704. https://doi.org/10.1016/j.fm.2020.103704

Hassan A, Farouk H, Abdul-Ghani R. Parasitological contamination of freshly eaten vegetables collected from local markets in Alexandria, Egypt: A preliminary study. *Food Cont* 2012; 26(2): 500-503. https://doi.org/ 10.1016/j.foodcont.2012.01.033

Hong S, Kim K, Yoon S, Park WY, Sim S, Yu JR. Detection of Cryptosporidium parvum in environmental soil and vegetables. J Korean Med Sci 2014; 29(10): 1367. https://doi.org/ 10.3346/jkms.2014.29.10.1367

Huvarova V, Kralik P, Vasickova P, Kubankova M, Verbikova V, Slany M, Moravkova M. Tracing of selected viral, bacterial, and parasitic agents on vegetables and herbs originating from farms and markets. *J Food Sci* 2018; 83(12): 3044-3053. https://doi.org/ 10.1111/1750-3841.14385

Isazadeh M, Mirzaii-Dizgah I, Shaddel M, Homayouni MM. The prevalence of parasitic contamination of fresh vegetables in Tehran, Iran. *Turkiye Parazitol Derg* 2020; 44(3): 143-8. https://doi.org/ 10.4274/tpd.galenos.2020.6469

Ismail Y. Prevalence of parasitic contamination in salad vegetables collected from supermarkets and street vendors in Amman and Baqa'a–Jordan. *Pol J Microbiol* 2016; 65(2): 201-207. https://pubmed.ncbi.nlm.nih.gov/28517923/

Istifanus WA, Panda SM. Parasitic agents in fresh fruits and vegetables sold in open markets in Bauchi, Nigeria. *J Food Qual Hazards Control* 2018; 5(3): 84-88. https://doi.org/ 10.29252/jfqhc.5.3.84

Jedrzejewski S, Graczyk TK, Slodkowicz-Kowalska A, Tamang L, Majewska AC. Quantitative assessment of contamination of fresh food produce of various retail types by human-virulent microsporidian spores. *Appl Environ Microbiol* 2007; 73(12): 4071-4073. https://doi.org/ 10.1128/AEM.00477-07

Khalil HI. Prevalence of enteric parasites in raw leafy vegetables in Baghdad City, Iraq. *Indian J Public Health Res Dev* 2019; 10: 446-450. https://www.researchgate.net/

Khan W, Khatoon N, Arshad S, Mohammed OB, Ullah S, Ullah I, Mahmoud AH. Evaluation of vegetables grown in dry mountainous regions for soil-transmitted helminths contamination. *Braz J Biol* 2021; 82: e238953. https://doi.org/10.1590/1519-6984.238953

Kłapeć T. Contamination of soil with geohelminth eggs on vegetable organic farms in the Lublin voivodeship, Poland. *Wiad Parazytol* 2009; 55(4): 405-409. https://pubmed.ncbi.nlm.nih.gov/20209816/

Kłapeć T, Borecka A. Contamination of vegetables, fruits and soil with geohelminth eggs on organic farms in Poland. *Ann Agric Environ Med* 2012; 19(3). https://pubmed.ncbi.nlm.nih.gov/23020033/

Kozan E, Gonenc B, Sarimehmetoglu O, Aycicek H. Prevalence of helminth eggs on raw vegetables used for salads. *Food Cont* 2005; 16(3): 239-242. https://doi.org/10.1016/j.foodcont.2004.02.005

Kudah C, Sovoe S, Baiden F. Parasitic contamination of commonly consumed vegetables in two markets in Ghana. *Ghana Med J* 2018; 52(2): 88-93. https://doi.org/10.4314/gmj.v52i2.5

Lalonde LF, Gajadhar AA. Detection of Cyclospora cayetanensis, Cryptosporidium spp., and Toxoplasma gondii on imported leafy green vegetables in Canadian survey. *Food Waterborne Parasitol* 2016; 2: 8-14. https://doi.org/10.1016/j.fawpar.2016.01.001

Lass A, Pietkiewicz H, Szostakowska B, Myjak P. The first detection of Toxoplasma gondii DNA in environmental fruits and vegetable samples. *Eur J Clin Microbiol Infect Dis* 2012; 31: 1101-1108. https://doi.org/ 10.1007/s10096-011-1414-8

Lass A, Szostakowska B, Myjak P, Korzeniewski K. The first detection of Echinococcus multilocularis DNA in environmental fruit, vegetable, and mushroom samples using nested PCR. *Parasitol Res* 2015; 114: 4023-4029. https://doi.org/ 10.1007/s00436-015-4630-9

Li J, Shi K, Sun F, Li T, Wang R, Zhang S, et al. Identification of human pathogenic Enterocytozoon bieneusi, Cyclospora cayetanensis, and Cryptosporidium parvum on the surfaces of vegetables and fruits in Henan, China. *Int J Food Microbiol* 2019; 307: 108292. https://doi.org/ 10.1016/j.ijfoodmicro.2019.108292

Li X, Zhang X, Jian Y, Wang G, Ma L, Schou C, et al. Detection of Cryptosporidium oocysts and Giardia cysts in vegetables from street markets from the Qinghai Tibetan Plateau Area in China. *Parasitol Res* 2020; 119: 1847-1855. https://doi.org/10.1007/s00436-020-06661-z

Luz JGG, Barbosa MV, Carvalho AGD, Resende SD, Dias JVL, Martins HR. Contamination by intestinal parasites in vegetables marketed in an area of Jequitinhonha Valley, Minas Gerais, Brazil. *Rev Nutr* 2017; 30: 127-136. https://doi.org/10.1590/1678-98652017000100012

Maikai BV, Baba-Onoja EBT, Elisha IA. Contamination of raw vegetables with Cryptosporidium oocysts in markets within Zaria metropolis, Kaduna State, Nigeria. *Food Cont* 2013; 31(1): 45-48. https://doi.org/ https://doi.org/10.1016/j.foodcont.2012.09.032

Malison MT, Su GS. Prevalence of intestinal parasites in selected vegetables at major public markets in Metro Manila, Philippines. *Asian Pac J Trop Med* 2009; 2(6): 37-39. https://oaji.net/articles/2016/3555-1469591714.pdf

Marchioro AA, Tiyo BT, Colli CM, de Souza CZ, Garcia JL, Gomes ML, et al. First detection of Toxoplasma gondii DNA in the fresh leafs of vegetables in South America. *Vector Borne Zoonotic Dis* 2016; 16(9): 624-626. https:// 10.1089/vbz.2015.1937

Marques CS, Sousa S, Castro A, da Costa JMC. Detection of Toxoplasma gondii oocysts in fresh vegetables and berry fruits. *Parasit Vectors* 2020; 13: 1-12. https://doi.org/ 10.1186/s13071-020-04040-2

Mendonça Ambrozim F, Pezzin J, Barreto Teresa Gradella D, Andrade de Souza MA. Enteroparasites in vegetables marketed in an ancient Brazilian city. *Rev Salud Publica* 2017; 19: 635-640. https://doi.org/10.15446/rsap.v19n5.57141

Mohamed MA, Siddig EE, Elaagip AH, Edris AMM, Nasr AA. Parasitic contamination of fresh vegetables sold at central markets in Khartoum state, Sudan. *Ann Clin Microbiol Antimicrob* 2016; 15: 1-7. https://doi.org/ 10.1186/s12941-016-0133-5

Mohammed RG, Hussein Kadhim HA, Ali JF. Diagnostic Study on Intestinal Parasites Isolated from Raw Consumed Vegetables in Misan City/Iraq. *Indian J Public Health Res Dev* 2019; 10(8). https://doi.org/10.5958/0976-5506.2019.02064.3

Mohemeed AA, Alrawi ZAA, Mahdi NK, Salih TA. Immunological and molecular investigation of the level of parasite contamination of some vegetables sold in the local markets of Ramadi City–Iraq. *Ann Romanian Soc Cell Biol* 2021; 5964-5973. https://doi.org/ 10.5539/gjhs.v8n10p178

Montanher CC, de Camargo Coradin D, Fontoura-da-Silva SE. Avaliação parasitológica em alfaces (Lactuca sativa) comercializadas em restaurantes self-service por quilo, da cidade de Curitiba, Paraná, Brasil. *Estud Biol* 2007; 29(66). https://doi.org/ 10.7213/reb.v29i66.22749

Muniswamappa K, Rao SD, Venkatramana K. Prevalence of Strongyloides stercoralis contaminating Coriandrum obtained from vendors in Karimnagar. *Ann Trop Med Public Health* 2012; 5(4). https://doi.org/ 10.4103/1755-6783.102030

Neres AC, Nascimento AH, Lemos KRM, Ribeiro EL, Leitão VO, Pacheco JBP, et al. Enteroparasitos em amostras de alface (Lactuva sativa var. crispa), no município de Anápolis, Goiás, Brasil. *Biosci J* 2011; 336-341. https://seer.ufu.br/index.php/biosciencejournal/article/view/7613/7240

Neto NJG, Pessoa RML, Queiroga IMBN, Magnani M, de Sousa Freitas FI, de Souza EL, et al. Bacterial counts and the occurrence of parasites in lettuce (Lactuca sativa) from different cropping systems in Brazil. *Food Cont* 2012; 28(1): 47-51. https://doi.org/ 10.1016/j.foodcont.2012.04.033

Nguyen TT, Traub R, Pham PD, Nguyen HV, Nguyen KC, Phung CD, et al. Prevalence and molecular characterization of Cryptosporidum spp. and Giardia spp. in environmental samples in Hanam province, Vietnam. *Food Waterborne Parasitol* 2016; 3: 13-20. https://doi.org/ 10.1016/j.fawpar.2016.03.003

Obebe OO, Aluko OO, Falohun OO, Akinlabi KB, Onyiche TE. Parasitic contamination and public health risk of commonly consumed vegetables in Ibadan-Nigeria. *Pan Afr Med J* 2020; 36(1). https://doi.org/ 10.11604/pamj.2020.36.126.19364

Olyaei A, Hajivandi L. Parasitological contamination of markets and farms in vegetables consumed in southern Iran. *Global Veterinaria* 2013;10(3) :327-331.https://doi.org/ 10.5829/idosi.gv.2013.10.3.71185

Paula P, Rodrigues PSDS, Tórtora JCDO, Uchôa CMA, Farage S. Contaminação microbiológica e parasitológica em alfaces (Lactuca sativa) de restaurantes self-service, de Niterói, RJ. *Rev Soc Bras Med Trop* 2003; 36: 535-537. https://doi.org/10.1590/S0037-86822003000400019

Perim LV, Custódio NCC, Lima VDCV, da Igreja JASL, Alves DDSMM, Storchilo HR, et al. Occurrence of parasites in salads in restaurants in Aparecida de Goiânia, Goiás, Brazil. *Rev Patol Trop/J Trop Pathol* 2020; 49(3). https://doi.org/10.5216/rpt.v49i3.62681

Puig Peña Y, Leyva Castillo V, Suárez A, Carrera Vara J, Molejón PL, Muñoz Y, et al. Calidad microbiológica de las hortalizas y factores asociados a la contaminación en áreas de cultivo en La Habana. *Rev Haban Cienc Méd* 2014; 13(1): 111-119. http://scielo.sld.cu/scielo.php?script=sci_arttext&pid=S1729-519X2014000100013

Punsawad C, Phasuk N, Thongtup K, Nagavirochana S, Viriyavejakul P. Prevalence of parasitic contamination of raw vegetables in Nakhon Si Thammarat province, southern Thailand. *BMC Public Health* 2019; 19: 1-7. https:// doi.org/ 10.1186/s12889-018-6358-9

Rafael K, Marchioro AA, Colli CM, Tiyo BT, Evangelista FF, Bezagio RC, et al. Genotyping of Giardia duodenalis in vegetables cultivated with organic and chemical fertilizer from street markets and community vegetable gardens in a region of Southern Brazil. *Trans R Soc Trop Med Hyg* 2017; 111(12): 540-545. https://doi.org/ 10.1093/trstmh/try013

Ranjbar-Bahadori SH, Mostoophi A, Shemshadi B. Study on Cryptosporidium contamination in vegetable farms around Tehran. *Trop Biomed* 2013; 30(2): 193-8. https://pubmed.ncbi.nlm.nih.gov/23959484/

Robertson LJ, Gjerde B. Occurrence of parasites on fruits and vegetables in Norway. *J Food Prot* 2001; 64(11): 1793-1798. https://doi.org/ 10.4315/0362-028x-64.11.1793

Rostami A, Ebrahimi M, Mehravar S, Omrani VF, Fallahi S, Behniafar H. Contamination of commonly consumed raw vegetables with soil transmitted helminth eggs in Mazandaran province, northern Iran. *Int J Food Microbiol* 2016; 225: 54-58. https://doi.org/ 10.1016/j.ijfoodmicro.2016.03.013

Ruiz BA, Victor D, de Moraes Alexandrino JHB, Nascimento RF, Rodrigues T, Silva JS. Análise parasitológica em alfaces (Lactuca sativa var. crispa) comercializadas em restaurantes. *An Semin Cient* UNIFACIG (5). https://pensaracademico.unifacig.edu.br/index

Rzeżutka A, Nichols RAB, Connelly L, Kaupke A, Kozyra I, Cook N, et al. Cryptosporidium oocysts on fresh produce from areas of high livestock production in Poland. *Int J Food Microbiol* 2010; 139(1-2): 96-101. https://doi.org/ 10.1016/j.ijfoodmicro.2010.01.027

Said DES. Detection of parasites in commonly consumed raw vegetables. Alexandria J Med 2012; 48(4): 345-352. https://doi.org/10.1016/j.ajme.2012.05.005

Saki J, Asadpoori R, Khademvatan S. Prevalence of intestinal parasites in vegetables consumed in Ahvaz, South West of Iran. *J Med Sci* 2013;13(06): 488-492 https://doi.org/10.3923/jms.2013.488.492

Sakkas H, Economou V, Bozidis P, Gousia P, Papadopoulou C, Karanis P. Detection of Cryptosporidium and Giardia in foods of plant origin in North-Western Greece. *J Water Health* 2020; 18(4): 574-578. https://doi.org/ 10.2166/wh.2020.037

Sakkas H, Economou V, Bozidis P, Gousia P, Papadopoulou C, Karanis P. Detection of Cryptosporidium and Giardia in foods of plant origin in North-Western Greece. *J Water Health* 2020; 18(4): 574-578. https://doi.org/ 10.2166/wh.2020.037

Salavati Z, Chalehchaleh AA, Rezaei F. Parasitic infections in raw vegetables of Kermanshah, Western Iran and their relation with season and washing procedures. *J Food Qual Hazards Control* 2017; 4(2): 37-41. https://magiran.com/p1715100

Shafa-ul-Haq SUH, Azhar Maqbool AM, Khan UJ, Ghazalah Yasmin GY, Razia Sultana RS. Parasitic contamination of vegetables eaten raw in Lahore. *Pakistan J Zool* 2014;46(5):13031309https://www.researchgate.net/publication/266740328_Parasitic_Contamination_of_Vegetables_Eaten_Raw_in_Lahore

Shahnazi M, Jafari-Sabet M. Prevalence of parasitic contamination of raw vegetables in villages of Qazvin Province, Iran. *Foodborne Pathog Dis* 2010; 7(9): 1025-1030. https://doi.org/ 0.1089/fpd.2009.0477

Shakir OM, Abdulla KK, Mustafa AA, Mustafa MA. Investigation of the presence of parasites that contaminate some fruits and vegetables in the Samarra City in Iraq. *Plant Arch* 2019; 19: 1184-1190.

Sherchand JB, Cross JH. Parasitic epidemiological studies of Cyclospora cayetanensis in Nepal. *Southeast Asian J Trop Med Public Health* 2004; 35: 1-8. https://pubmed.ncbi.nlm.nih.gov/10695790/

Sim S, Won J, Kim JW, Kim K, Park WY, Yu JR. Simultaneous molecular detection of Cryptosporidium and Cyclospora from raw vegetables in Korea. *Korean J Parasitol* 2017; 55(2): 137. https://doi.org/ 10.3347/kjp.2017.55.2.137

Simões M, Pisani B, Marques EGL, Prandi MAG, Martini MH, Chiarini PFT, et al. Hygienic-sanitary conditions of vegetables and irrigation water from kitchen gardens in the municipality of Campinas, SP. *Braz J Microbiol* 2001; 32: 331-333. https://doi.org/ 10.1590/S1517-83822001000400015

Siyadatpanah A, Tabatabaei F, Emami ZA, Spotin A, Fallah OV, Assadi M, et al. Parasitic contamination of raw vegetables in Amol, North of Iran. *Arch Clin Infect Dis* 2013 April; 8(2): e15983. https://doi.org/ 10.5812/archcid.15983

Soares B, Cantos GA. Qualidade parasitológica e condições higiênico-sanitárias de hortaliças comercializadas na cidade de Florianópolis, Santa Catarina, Brasil. *Rev Bras Epidemiol* 2005; 8: 377-384. https://doi.org/10.1590/S1415-790X2005000400006

Srikanth R, Naik D. Prevalence of Giardiasis due to wastewater reuse for agriculture in the suburbs of Asmara City, Eritrea. *Int J Environ Health Res* 2004; 14(1): 43-52. https://doi.org/ 10.1080/09603120310001633912

Su GLS, Mariano CMR, Matti NSA, Ramos GB. Assessing parasitic infestation of vegetables in selected markets in Metro Manila, Philippines. *Asian Pacific J Trop Dis* 2012;2(1):51-54. https://doi.org/ 10.1016/S2222-1808(12)60012-7

Sunil B, Thomas DR, Latha C, Shameem H. Assessment of parasitic contamination of raw vegetables in Mannuthy, Kerala state, India. *Vet World* 2014;7(4). https://doi.org/ 10.14202/vetworld.2014.253-256

Taghipour A, Javanmard E, Haghighi A, Mirjalali H, Zali MR. The occurrence of Cryptosporidium sp., and eggs of soil-transmitted helminths in market vegetables in the north of Iran. *Gastroenterol Hepatol Bed Bench* 2019;12(4):364. https://doi.org/10.22037/ghfbb.v12i4.1689

Tiyo R, de Souza CZ, Piovesani AFA, Tiyo BT, Colli CM, Marchioro AA, et al. Predominance of Giardia duodenalis assemblage AII in fresh leafy vegetables from a market in southern Brazil. *J Food Prot* 2016;79(6):1036-9. https://doi.org/ 10.4315/0362-028X.JFP-15-306

Tram NT, Hoang LM, Cam PD, Chung PT, Fyfe MW, Isaac-Renton JL, et al. Cyclospora spp. in herbs and water samples collected from markets and farms in Hanoi, Vietnam. *Trop Med Int Health*. 2008;13(11):1415-20. https://doi.org/ 10.1111/j.1365-3156.2008.02158.x

Uga S, Hoa NT, Noda S, Moji K, Cong L, Aoki Y, et al. Parasite egg contamination of vegetables from a suburban market in Hanoi, Vietnam. Nepal Medical College journal: NMCJ. 2009;11(2):75-8. https://doi/ 10.2149/tmh.2010-02

Utaaker KS, Kumar A, Joshi H, Chaudhary S, Robertson LJ. Checking the detail in retail: occurrence of Cryptosporidium and Giardia on vegetables sold across different counters in Chandigarh, India. *Int J Food Microbiol*. 2017;263:1-8. https://doi.org/ 10.1016/j.ijfoodmicro.2017.09.020

Al-Megrin WA. Prevalence of intestinal parasites in leafy vegetables in Riyadh, Saudi Arabia. *Int J Trop Med*. 2010;5(2):20-3. https://doi.org/ 10.3923/ijtmed.2010.20.23

Wali Khan WK, Ghazal Mumtaz GM, Saima Bibi SB, Salma Afzal SA. Parasitic contamination of fresh vegetables sold at Upper and Lower Dir districts, Khyber Pakhtunkhwa, Pakistan. *Pakistan J. Zoo* 2017; 9(3):1115-1118 https://doi.org/ 10.17582/journal.pjz/2017.49.3.sc3
